# Fucoidan Alleviates Renal Fibrosis in Diabetic Kidney Disease *via* Inhibition of NLRP3 Inflammasome-Mediated Podocyte Pyroptosis

**DOI:** 10.3389/fphar.2022.790937

**Published:** 2022-03-18

**Authors:** Mei-Zi Wang, Jie Wang, Dong-Wei Cao, Yue Tu, Bu-Hui Liu, Can-Can Yuan, Huan Li, Qi-Jun Fang, Jia-Xin Chen, Yan Fu, Bing-Ying Wan, Zi-Yue Wan, Yi-Gang Wan, Guo-Wen Wu

**Affiliations:** ^1^ Department of Traditional Chinese Medicine, Nanjing Drum Tower Hospital Clinical College of Nanjing University of Chinese Medicine, Nanjing, China; ^2^ Institute of Chinese Medicine, Nanjing University, Nanjing, China; ^3^ Department of Traditional Chinese Medicine, Nanjing Drum Tower Hospital, The Affiliated Hospital of Nanjing University Medical School, Nanjing, China; ^4^ Department of Nephrology, Nanjing Drum Tower Hospital, The Affiliated Hospital of Nanjing University Medical School, Nanjing, China; ^5^ Department of Traditional Chinese Medicine Health Preservation, Acupuncture, Moxibustion and Massage College, Health Preservation and Rehabilitation College, Nanjing University of Chinese Medicine, Nanjing, China; ^6^ Graduate School of Social Sciences, Faculty of Social Sciences, Hitotsubashi University, Tokyo, Japan; ^7^ Jilin Province Huinan Chonglong Bio-Pharmacy Co., Ltd., Huinan, China

**Keywords:** fucoidan, diabetic kidney disease, renal fibrosis, podocyte pyroptosis, NLRP3 inflammasome

## Abstract

**Background:** Fucoidan (FPS) has been widely used to treat renal fibrosis (RF) in patients with diabetic kidney disease (DKD); however, the precise therapeutic mechanisms remain unclear. Recently, research focusing on inflammation-derived podocyte pyroptosis in DKD has attracted increasing attention. This phenomenon is mediated by the activation of the nucleotide-binding oligomerization domain (Nod)-like receptor family pyrin domain-containing 3 (NLRP3) inflammasome, leading to RF during DKD progression. Therefore, we designed a series of experiments to investigate the ameliorative effects of FPS on RF in DKD and the mechanisms that are responsible for its effect on NLRP3 inflammasome-mediated podocyte pyroptosis in the diabetic kidney.

**Methods:** The modified DKD rat models were subjected to uninephrectomy, intraperitoneal injection of streptozotocin, and a high-fat diet. Following induction of renal injury, the animals received either FPS, rapamycin (RAP), or a vehicle for 4 weeks. For *in vitro* research, we exposed murine podocytes to high glucose and MCC950, an NLRP3 inflammasome inhibitor, with or without FPS or RAP. Changes in the parameters related to RF and inflammatory podocyte injury were analyzed *in vivo*. Changes in podocyte pyroptosis, NLRP3 inflammasome activation, and activation of the adenosine monophosphate-activated protein kinase (AMPK)/mammalian target of rapamycin complex 1 (mTORC1)/NLRP3 signaling axis involved in these changes were analyzed *in vivo* and *in vitro*.

**Results:** FPS and RAP ameliorated RF and inflammatory podocyte injury in the DKD model rats. Moreover, FPS and RAP attenuated podocyte pyroptosis, inhibited NLRP3 inflammasome activation, and regulated the AMPK/mTORC1/NLRP3 signaling axis *in vivo* and *in vitro*. Notably, our data showed that the regulative effects of FPS, both *in vivo* and *in vitro*, on the key signaling molecules, such as p-AMPK and p-raptor, in the AMPK/mTORC1/NLRP3 signaling axis were superior to those of RAP, but similar to those of metformin, an AMPK agonist, *in vitro*.

**Conclusion:** We confirmed that FPS, similar to RAP, can alleviate RF in DKD by inhibiting NLRP3 inflammasome-mediated podocyte pyroptosis via regulation of the AMPK/mTORC1/NLRP3 signaling axis in the diabetic kidney. Our findings provide an in-depth understanding of the pathogenesis of RF, which will aid in identifying precise targets that can be used for DKD treatment.

## Introduction

An increasing body of evidence in both clinical and experimental animal models has shown that inflammation is an important contributor to renal fibrosis (RF) in the progression of diabetic kidney disease (DKD) (Cooper and Warren, 2019). Inflammatory podocyte injury is the key pathogenic factor that triggers and sustains RF, which is closely associated with renal dysfunction and renal failure (Dai et al., 2017). Therefore, precision therapies that protect against podocyte inflammation are considered to have great significance in treatment of RF in DKD. Inflammation-derived podocyte pyroptosis is a newly discovered cell death pathway that plays a critical role in podocyte injury in DKD (Lin et al., 2020). Pyroptosis is mediated by the nucleotide-binding oligomerization domain (Nod)-like receptor family pyrin domain-containing 3 (NLRP3) inflammasome activation, caspase activation, cell membrane pore formation characterized by gasdermin D (GSDMD), and release of interleukin (IL)-1β and IL-18. NLRP3 inflammasome activation plays a central role in pyroptosis (He et al., 2015; Shi et al., 2015; Liu et al., 2016). It has been demonstrated that podocytes, as a group of renal residential cells, express all the necessary components of NLRP3 inflammasome, which is activated and contributes to inflammatory damage induced by high glucose (HG) (Xiong et al., 2020). In addition, Birnbaum et al. (2018) report a combination of sodium-dependent glucose transporters-2 inhibitor and dipeptidyl peptidase-4 (DPP-4) inhibitors can delay DKD progression, and that their therapeutic actions are closely related to the inhibition of NLRP3 inflammasome activation. Therefore, it is possible to alleviate inflammatory podocyte injury in glomeruli of kidneys affected by DKD by targeting NLRP3 inflammasome activation.

NLRP3 inflammasome can be activated by diverse stimuli and involves multiple signaling pathways, including the reactive oxygen species/thioredoxin-interacting protein, nuclear factor (NF)-κB, nuclear factor erythroid-related factor 2, long non-coding RNA, and mitogen-activated protein kinases (Paik et al., 2021). In addition, some studies demonstrated the NLRP3 inflammasome’s crucial role in pyroptosis initiation and pro-inflammatory cytokine production in DKD (Xiong et al., 2021). For instance, NLRP3 deficiency in diabetic mice significantly blocks Caspases-1 mediated IL-1β secretion and protects against renal injury *in vivo* (Yu et al., 2020). A strong upregulation of pyroptosis-related proteins, including NLRP3 and Caspase-1, in diabetic tissue has been observed (Lin et al., 2020). Additional research showed that many signaling mechanisms of NLRP3 inflammasome activation are involved in diabetic complications. Zhang et al. (2017) found that autophagy can downregulate NLRP3 inflammasome via mammalian target of rapamycin (mTOR) signaling. Accordingly, Yang et al. (2019) reported that NLRP3 inflammasome can be inhibited by metformin and rapamycin (RAP, an mTOR inhibitor) by targeting the adenosine monophosphate-activated protein kinase (AMPK)/mTOR complex 1 (mTORC1)-dependent effects in diabetic cardiomyopathy. Thus, we suggest that regulating the AMPK/mTORC1-related signaling axis is of great importance in the inhibition of NLRP3 inflammasome activation.

Fucoidan (FPS) is a class of fucose-rich sulfated carbohydrates found in brown marine algae and echinoderms, and it was recently identified in *Laminaria japonica*, a traditional Chinese herbal medicine (van Weelden et al., 2019). Previous studies have indicated that FPS has an attractive array of bioactivities and potential applications, including anti-inflammation and anti-cancer activities as well as the inhibition of the immune response and of pathogens (Fitton et al., 2015). Over the last few years, research into FPS has continued to gained pace and suggests potential therapeutic or beneficial roles in DKD (Wang et al., 2019). Chen et al. (2015) reported that low molecular weight FPS ameliorates DKD by inhibiting epithelial-mesenchymal transition (EMT) and RF. Despite this, the pharmacological mechanistic link between anti-RF actions and protection against podocyte inflammation correlated with diabetic kidney remains poorly understood.

In this study, we used a modified DKD rat model and murine podocytes to assess the ameliorative effects of FPS on RF and inflammatory podocyte injury *in vivo* and *in vitro* and to clarify the anti-RF mechanisms of FPS via targeting NLRP3 inflammasome-mediated podocyte pyroptosis in the diabetic kidney. These results may provide a precise therapeutic strategy for RF in patients with DKD.

## Materials and Methods

### Animals, Drug, and Reagents

All experiments were performed using male Sprague-Dawley rats weighing 200–220 g, purchased from the Experimental Animal Centre of Nanjing University (Nanjing, China) (License No: SCXK [Shanghai] 2012-0006). All rats were housed (six rats/cage) at 22 ± 3°C and 50 ± 10% humidity using a 12 h light/dark cycle, fed a specific pathogen-free grade standard rat chow (catalogue number 1010008) from Xietong Pharmaceutical Bio-engineering Co., Ltd. (Nanjing, China), and provided tap water ad libitum in the Experimental Animal Centre of Nanjing Drum Tower Hospital, the Affiliated Hospital of Nanjing University Medical School. The animal ethics committee of Nanjing University Medical School approved the surgical procedures and protocols. FPS (C_6_H_10_O_7_S, CAS: 9072-19-9) was obtained from Jilin Province Huinan Chonglong Bio-Pharmacy Co., Ltd. (Huinan, China), and dissolved in 10% dimethyl sulfoxide at a concentration of 1 g/L. RAP and lipopolysaccharide (LPS) were purchased from Gene Operations (Shanghai, China). Novolin N was obtained from Novo Nordisk Company (Tianjin, China). STZ was purchased from Sigma-Aldrich (St. Louis, MO, United States). The total protein extraction kit and bicinchoninic acid protein assay kit were provided by Key-Gentec (Nanjing, China). Antibodies against fibronectin (FN), collagen type I (collagen I), transforming growth factor-β1 (TGF-β1), Smad2/3, IL-6, toll-like receptor 4 (TLR4), phosphorylated AMPK (p-AMPK), raptor, phosphorylated raptor (p-raptor), mTORC1, phosphorylated mTORC1 (p-mTORC1), CD2-associated protein (CD2AP), and glyceraldehydes-3-phosphate dehydrogenase (GAPDH) were obtained from Cell Signalling Technology (Danvers, MA, United States). Antibodies against nephrin, podocin, neph1(KIRREL), NLRP3, C-terminal caspase recruitment domain (ASC), pro IL-18, pro IL-1β, IL-1β, IL-18, and β-actin were obtained from Abcam (Cambridge, United Kingdom). Antibodies against Caspase-1, pro-Caspase-1, and cleaved-Caspase-1 were obtained from Absin (Shanghai, China). Antibodies against GSDMD and GSDMD-N were obtained from Proteintech (Wuhan, China).

### Animal Experimental Design

Twenty-six rats were divided into four groups using a random number table, with five, seven, seven, and seven rats in the sham-operated group, the DKD model group, the FPS-treated group, and the RAP-treated group, respectively. In particular, the left kidney was exposed during surgery for the rats in the sham-operated group. The rats were then given distilled water and a standard diet for 18 weeks. In contrast, the rats in the other three groups were given a 40% high-fat diet containing 19.8% fat, 22.3% crude protein, and 44.6% carbohydrates, for 4 weeks. The rats in these three groups were then subjected to left nephrectomy and received two intraperitoneal injections (3 days apart) of STZ at a dosage of 35 mg/kg. This process lasted for 10 weeks, finally establishing a modified rat model of DKD, as described in detail in our previous studies (Mao et al., 2015; Wu et al., 2017; Wu et al., 2018; Han et al., 2019). Once the DKD rat models had been established, we used gastric gavage to administer appropriate daily treatments: FPS was administered to rats in the FPS-treated group (abbreviated as the FPS group), while the rats of the sham-operated group, the DKD model group, and the RAP-treated group (abbreviated as the Sham, the Vehicle, and the RAP groups) were treated with 2 ml of distilled water (vehicle) or RAP, respectively. After 4 weeks of treatment with the different interventions, all rats were anesthetized and sacrificed by cardiac puncture. Blood, urine, liver, and kidneys were collected for the detection of various indicators. The *in vivo* experimental process is shown in Supplementary Figure S1. In the clinic, 600 mg/day of FPS is normally used to treat a 60 kg patient with CKD. Based on the standard animal conversion formula, the effective amount of FPS in a rat weighing 200 g was determined to be 120 mg/kg/day. The RAP dose used in this experiment (1 mg/kg/day) was used previously by Wu et al. (2018). Two rats died in the Vehicle, FPS, and RAP groups, respectively, due to severe diabetes and its associated complications during the experiment. Therefore, at the end of the experiment, only five rats were included in each group.

### Biochemical Parameters

Body weight (BW) and blood glucose (BG) were tested before modeling and every 2 weeks thereafter. The left kidneys of rats in the experimental groups were removed and weighed after cardiac puncture. After 4 weeks of drug intervention, the rats were anesthetized, and blood samples (5 ml) were drawn from the heart. A range of biochemical parameters were tested, including serum alanine transaminase (ALT), serum aspartate transaminase (AST), serum creatinine (Scr), and blood urea nitrogen (BUN) levels. Prior to sacrifice, we collected urinary samples from the four groups and used these samples to detect 24 h urinary albumin (UAlb) levels. Chromatometry was used to determine these parameters, as previously described (Liu et al., 2019).

### Foot Process Form and Glomerular Basement Membrane Thickness

Tissue samples from renal cortex for electron microscopy (EM) assessment were fixed in 2.5% glutaraldehyde in 0.1 mol/L phosphate buffer (PB) for several days at 4°C. After washing in PB and post-fixing in 1% osmium tetroxide (OsO_4_) for 2 h, the fixed material was dehydrated through an ethanol propylene oxide series and embedded in Araldite M. Ultrathin sections were prepared and stained with uranyl acetate and lead citrate, and foot process form and GBM thickness were observed and photographed under a JEM-1011 transmission electron microscope (JEOL, Tokyo, Japan). Five glomeruli were randomly selected from each section. According to the method described by Haas (2009), GBM thickness was directly measured and calculated using Image-Pro Plus (IPP) 6.0 software (Media Cybernetic). The results were confirmed by a professional pathologist.

### Immunohistochemistry and Histological Assay

Kidney samples were fixed in 4% paraformaldehyde and embedded in paraffin. Sections of 3 μm thickness were cut perpendicularly to the long axis of the kidney for immunohistochemistry (IHC) and morphometric analyses. For IHC analysis, the paraffin-embedded kidney sections were deparaffinized in xylene, hydrated in graded alcohol and water, and subsequently placed in 3% hydrogen peroxide (H_2_O_2_) to eliminate endogenous peroxidase activity. Then, the sections were blocked with normal goat serum, followed by incubation with anti-podocin, anti-CD2AP, anti-FN, anti-collagen I, anti-NLRP3, anti-ASC, and anti-Caspase-1 antibodies overnight at 4°C, and then with goat anti-rabbit IgG for 30 min at 37°C. The sections were stained with diaminobenzidine, counterstained with hematoxylin, dehydrated with gradient ethanol, purified with xylene, and fixed with neutral balsam. The proportion (%) of positively stained glomerular area across five fields of view was analyzed using IPP. For histological analysis, the sections were stained with periodic acid-Schiff (PAS) staining, Masson’s trichrome staining, and hematoxylin-eosin (H&E) staining. The kidney and liver sections were examined using light microscopy (LM). Five sections (PAS staining) from each group were randomly selected to calculate glomerular cell population (GCP) per glomerulus by IPP. Analogously, five sections (Masson staining) from each group were randomly selected to calculate the area of extracellular matrix (ECM) and the area of collagen per glomerulus by IPP. Liver sections were stained with H&E. These results were confirmed by a professional pathologist.

### Immunofluorescence *in Vivo* Assay

Paraffin-embedded kidney sections were used to assess the co-localization of zonula occludens-1 (ZO-1) and GSDMD-N. First, the sections were subjected to microwave-based antigen retrieval using ethylene diamine tetraacetic acid antigen retrieval solution (pH 8.0), followed by tyramide signal amplification (TSA). Briefly, the following steps were performed: 1) incubation of sections with anti-GSDMD-N (1:200; Abcam; EPR20829-408) at 4°C overnight, 2) incubation with horseradish peroxidase (HRP) (1:500; Servicebio)-conjugated secondary antibody for 50 min at room temperature, 3) reaction with CY3-TSA (Servicebio, China) for 10 min in the dark, 4) removal of nonspecific binding antibodies by microwave treatment, 5) incubation with anti-ZO-1 (1:200; Servicebio) at 4°C overnight, 6) incubation with HRP (1:500; Servicebio)-conjugated secondary antibody for 50 min in the dark, 7) reaction with fluorescein isothiocyanate-TSA (Servicebio; China) for 10 min in the dark, and 8) staining with diamidine phenyl indole solution for 10 min. The images were captured by fluorescence microscopy using excitation wavelengths of 330–380 nm (blue), 510–560 nm (red) and 515–555 nm (green). The proportion (%) of positively stained glomerular area across three fields of view was analyzed using IPP. The results were confirmed by a professional pathologist.

### Western Blotting *in Vivo* Analysis

Western blotting (WB) analysis was performed as previously described (Wang et al., 2021). The protein expression levels of FN, collagen I, TGF-β1, Smad2/3, podocin, CD2AP, nephrin, neph1, IL-6, TLR4, GSDMD, GSDMD-N, NLRP3, ASC, pro-Caspase-1, cleaved-Caspase-1, p-AMPK, p-raptor, raptor, p-mTORC1, mTORC1, GAPDH, and β-actin were examined, respectively. WB analysis was then performed on kidney samples from each group. We used the protocol described in our previous publications (Tu et al., 2020; Liu et al., 2021b).

### Cell Culture Treatment

An immortalized mouse podocyte cell-5 line (MPC-5 cells) was kindly provided by Dr. Jian Yao (University of Yamanashi, Chuo, Japan) and cultured as described previously (Liu et al., 2021b). In brief, MPC-5 cells were cultured at a permissive temperature (33°C) in 5% carbon dioxide (CO_2_) in Dulbecco’s modified Eagle’s medium (DMEM) containing 10% fetal bovine serum (FBS; Gibco, Grand Island, NY, United States) and recombinant interferon (IFN)-γ (10 U/ml, Cambridge, United Kingdom). Following passage, MPC-5 cells were cultured at 37°C in 5% CO_2_ for 14 days in DMEM without IFN-γ to induce differentiation. The cells were then exposed to HG (30 mmol/L D-glucose) and MCC950 (10 μmol/L, an NLRP3 inhibitor), with or without FPS, at a dose of 20 μg/ml or RAP at a dose of 20 nmol/L for 24 h. For this experiment, the doses of FPS, RAP, and MCC950 were described by Liu et al. (2021a), Wu et al. (2018), and Liu et al. (2021b), respectively.

### Cell Viability Assessment

The viability of MPC-5 cells was assessed using the cell counting kit (CCK-8) (Beyotime, Shanghai, China). The cells were seeded into 96-well plates, with three replicate wells for each group, at a density of 1 × 10^4^ cells per well, in 100 μl medium. After the cells were incubated for the indicated time period, 10 μl of CCK-8 solution was added to each well, followed by incubation for 2 h. The optical density was determined at an absorbance of 450 nm, and cell viability was calculated.

### Immunofluorescence *in Vitro* Assay

First differentiated podocytes grown on glass coverslips were fixed with paraformaldehyde (4% in PBS) and permeabilised with Triton ×-100 (0.25%). Then the cells were incubated with primary antibody directed against GSDMD-N (1:200) at 4°C overnight. Next, the coverslips were incubated with Alexa Fluor 488 goat anti-rabbit IgG (1:200) as a secondary antibody for 1 h at room temperature. Finally, the coverslips were mounted with Fluoroshield™ and 4′,6-diamidine-2′-phenylindole dihydrochloride (DAPI; F6057, Sigma-Aldrich, blue fluorescent dye) for 10 min in the dark. The images were acquired using an epifluorescence inverted microscope (IX81, Olympus, Tokyo, Japan) equipped with a cell imaging software (Soft Imaging System GmbH, Munster, Germany). The results were confirmed by a professional pathologist.

### WB *In Vitro* Analysis

MPC-5 cells were treated with appropriate treatments for 24 h. Following treatment, the cell lysates were separated by gel electrophoresis and blotted with antibodies against GSDMD, GSDMD-N, NLRP3, ASC, pro-Caspase-1, cleaved-Caspase-1, pro IL-18, IL-18, pro IL-1β, IL-1β, p-AMPK, p-raptor, raptor, p-mTORC1, mTORC1, GAPDH and β-actin, respectively. HRP-conjugated anti-rabbit IgG antibody was used as the secondary antibody. WB analysis was then performed on the samples of cells from each group. We used the protocol described in our previous publications (Tu et al., 2020; Liu et al., 2021b).

### Statistical Analysis

The WB assessment was repeated at least three independent times, and the individual data were subjected to densitometric analysis. The data are expressed as the mean ± standard deviation (SD). Statistical analysis was performed by one-way analysis of variance (ANOVA) on normally distributed data, with least significant difference (LSD) post-hoc test, or non-parametric Kruskal–Wallis test if not. A *p*-value < 0.05 or <0.01 indicated a statistically significant difference.

## Results

### FPS and RAP Ameliorate Renal Fibrosis *In Vivo*

**FIGURE 1:**
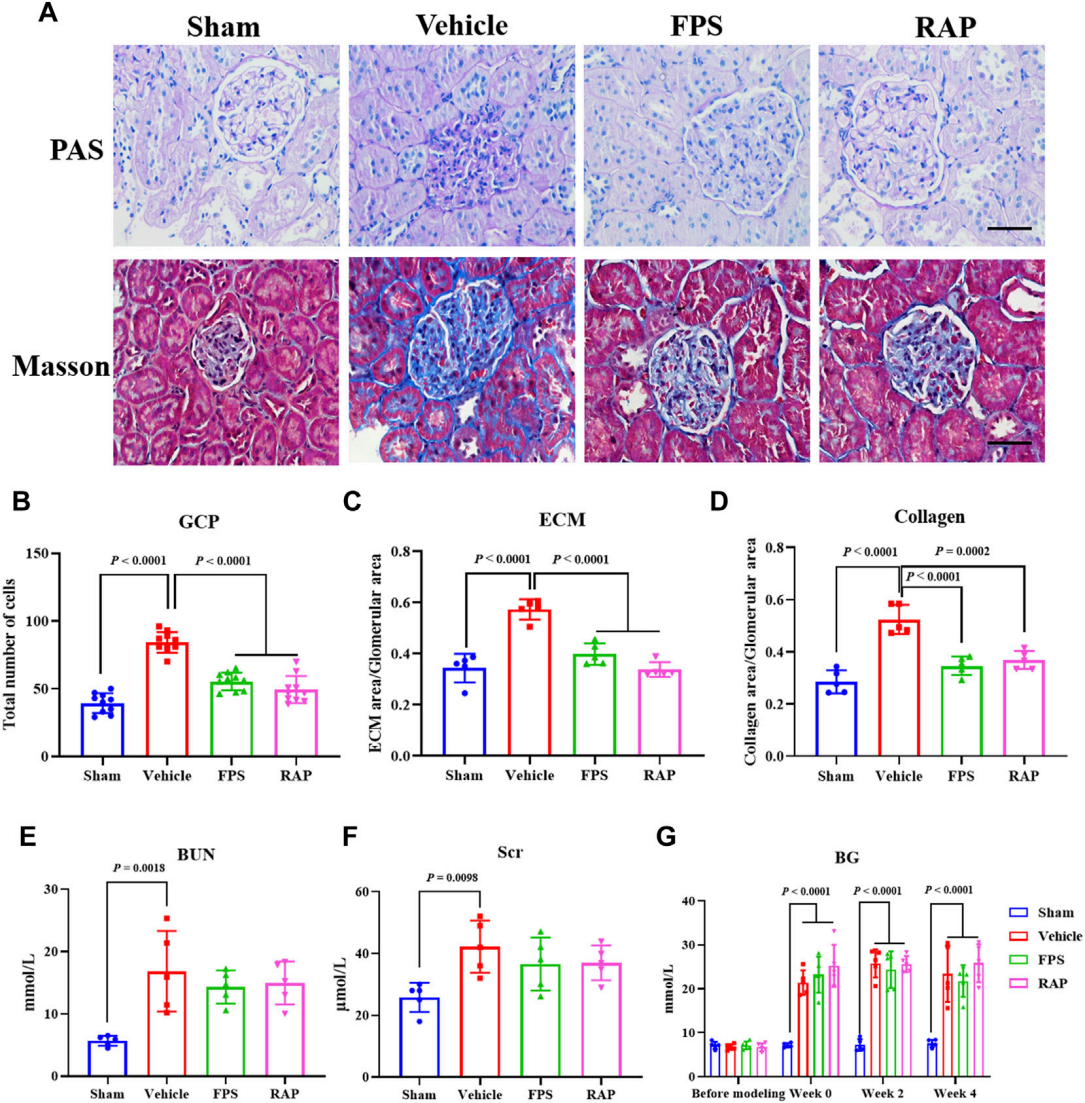
Effects of FPS and RAP on GS and renal function *in vivo*. **(A)** The staining of PAS and Masson stains in the diabetic kidneys (×400). Scale bar = 50 μm. **(B)** The number of GCP. **(C)** The rate of ECM area/glomerular area. **(D)** The rate of collagen area/glomerular area. **(E,F)** The levels of Scr and BUN. **(G)** The dynamic changes in BG after drug intervention during 4 weeks. The data are expressed as the mean ± SD.

As is widely known, the pathomorphological changes of RF in DKD are mainly characterized by glomerular sclerosis (GS) (Alicic et al., 2017). Thus, we first studied the effects of FPS and RAP on GS in these DKD model rats. In Figure 1A, we found that, compared with the Sham group rats, after modeling, the obvious pathological changes in the Vehicle group rats, including glomerular mild hypertrophy, capillary loop area reduction, glomerular cell proliferation, ECM expansion, and collagen deposition were detected. After treatment with FPS or RAP, the degree of pathological changes in GS in these DKD model rats, including GCP, and the rates of ECM and collagen area to glomerular area were improved significantly when compared to the Vehicle group rats (Figures 1B–D). In addition, after treatment with FPS or RAP, the decreasing tendency of BUN and Scr in the FPS and RAP group rats was also observed at different degrees when compared to the Vehicle group rats (Figures 1E,F). Here, notably, in Figure 1G, we found that FPS and RAP did not affect hyperglycemia in these modified DKD model rats.

**FIGURE 2:**
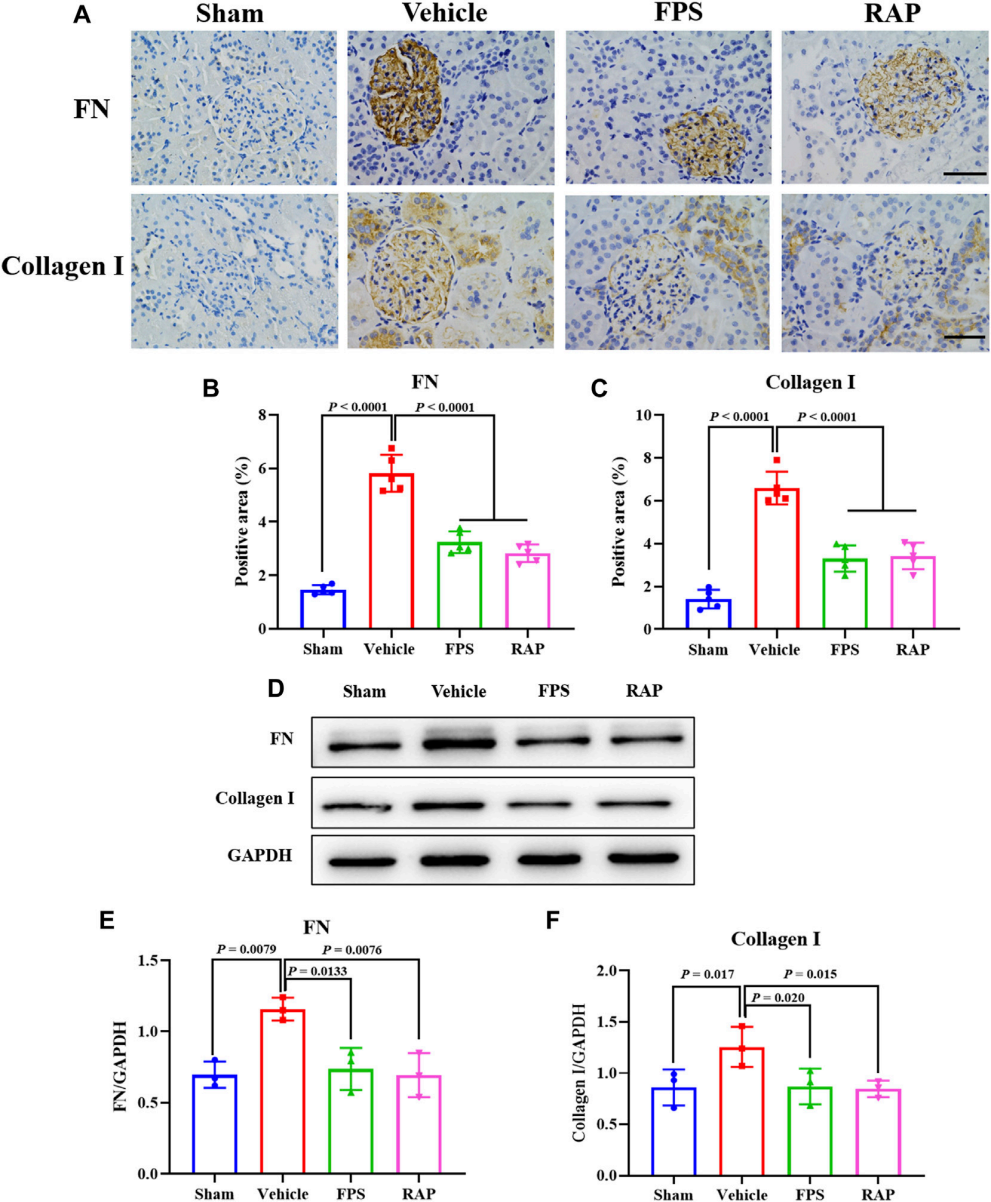
Effects of FPS and RAP on the expression levels of FN and collagen I *in vivo*. **(A)** The immunostaining of FN and collagen I in glomeruli (×400). Scale bar = 50 μm. **(B,C)** The positive area of FN and collagen I in glomeruli. **(D)** The WB analysis of FN and collagen I in the diabetic kidneys. **(E,F)** The rate of FN and collagen I to GAPDH, respectively. The data are expressed as the mean ± SD.

We then examined the effects of FPS and RAP on the expression levels of FN and collagen I as the markers of RF in the kidneys of these DKD model rats by PAS staining and WB analysis. In Figures 2A,D, we found that, compared with the Sham group rats, after modeling, the altered immunostaining extent of FN and collagen I in glomeruli and the higher protein expression levels of FN and collagen I in the kidneys of these DKD model rats were detected, respectively, and significantly improved in the FPS and RAP group rats after treatment with FPS or RAP when compared to the Vehicle group rats (Figures 2B,C,E,F).

**FIGURE 3:**
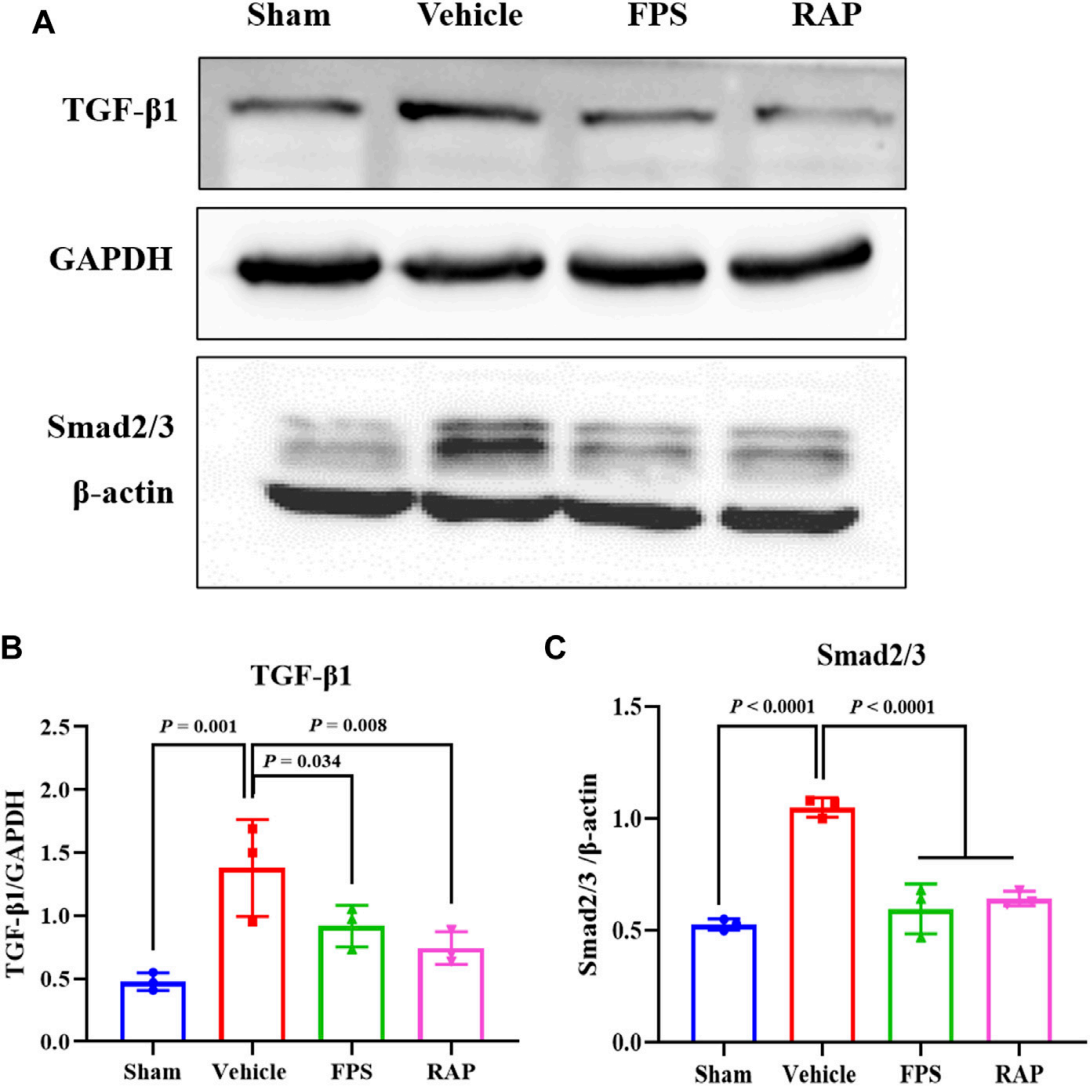
Effects of FPS and RAP on the TGF-β1/Smad2/3 pathway *in vivo*. **(A)** The WB analysis of TGF-β1 and Smad2/3 I in the diabetic kidneys. **(B,C)** The rate of TGF-β1 and Smad2/3 to GAPDH or β-actin, respectively. The data are expressed as the mean ± SD.

Further, using WB analysis, we investigated the effects of FPS and RAP on the TGF-β1/Smad2/3 pathway as a given signaling mechanism inducing RF in the kidneys of these DKD model rats. In Figure 3A, after modeling, the increased protein expression levels of TGF-β1 and Smad2/3 in the kidneys of these DKD model rats were appeared, respectively, and significantly decreased in the FPS and RAP group rats when compared to the Vehicle group rats (Figures 3B,C).

In brief, the above results showed that FPS and RAP significantly ameliorated RF in the DKD model rats.

### FPS and RAP Alleviate Inflammatory Podocyte Injury *In Vivo*

**FIGURE 4:**
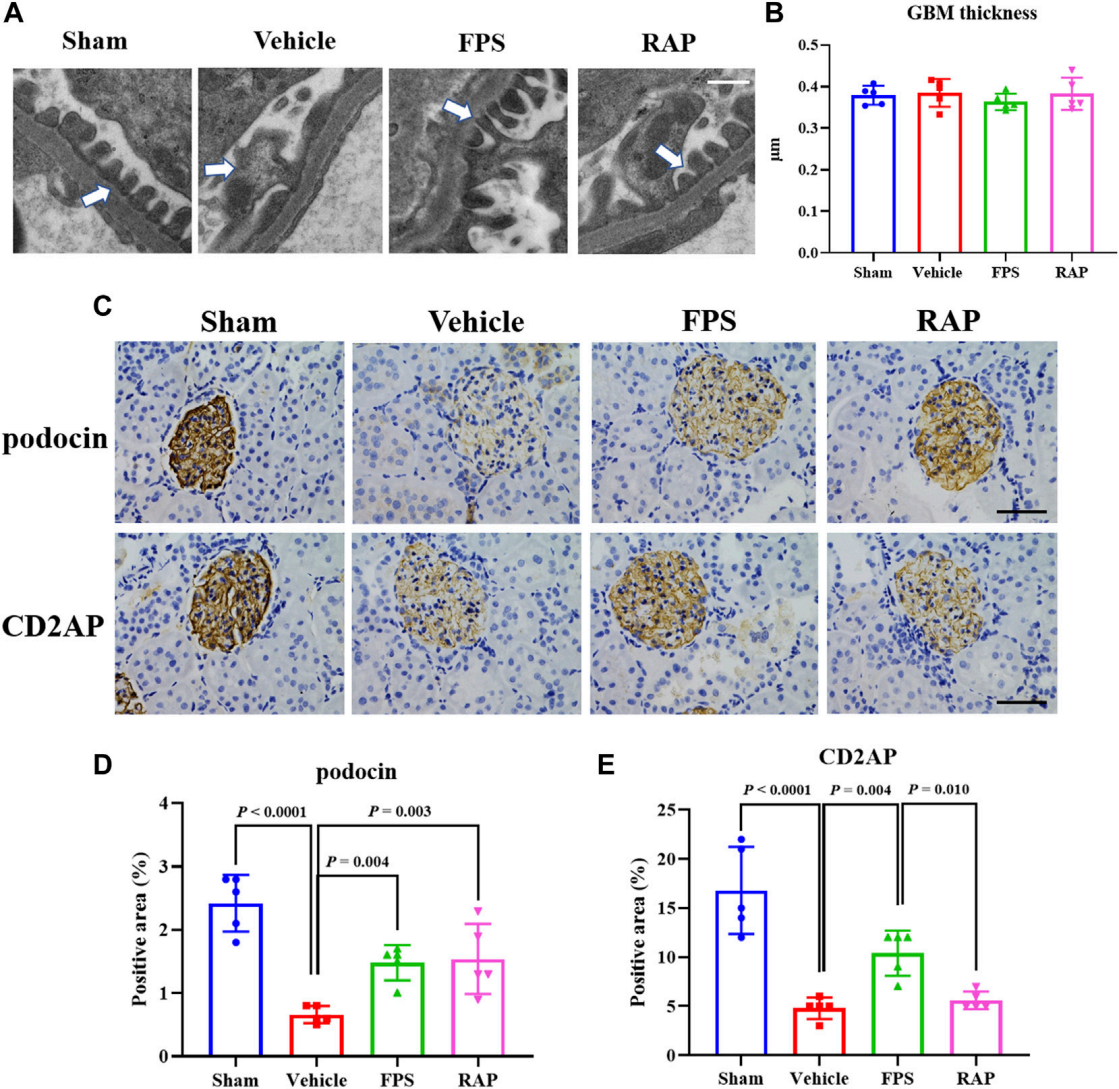
Effects of FPS and RAP on foot process form, GBM thickness, and the expression characteristics of podocin and CD2AP *in vivo*. **(A)** The ultra-microstructure of foot process form and GBM (×11,500). Scale bar = 500 nm. **(B)** The GBM thickness. **(C)** The immunostaining of podocyte and CD2AP in glomeruli (×400). Scale bar = 50 μm. **(D,E)** The positive area of podocin and CD2AP in glomeruli. The data are expressed as the mean ± SD.

During the progression of DKD, inflammatory podocyte injury is an important mechanism by which hyperglycemia contributes to GS (Alicic et al., 2017). We first investigated the effects of FPS and RAP on foot process form and GBM thickness by EM in these DKD model rats. In Figure 4A, we found that, compared with the Sham group rats, widened, shortened, and fused foot processes were clearly evident in the Vehicle group rats. After treatment with FPS or RAP, foot process effacement in these DKD model rats was significantly improved in the FPS and RAP group rats when compared to the Vehicle group rats. However, no significant difference in GBM thickness was detected among the four groups (Figure 4B).

**FIGURE 5:**
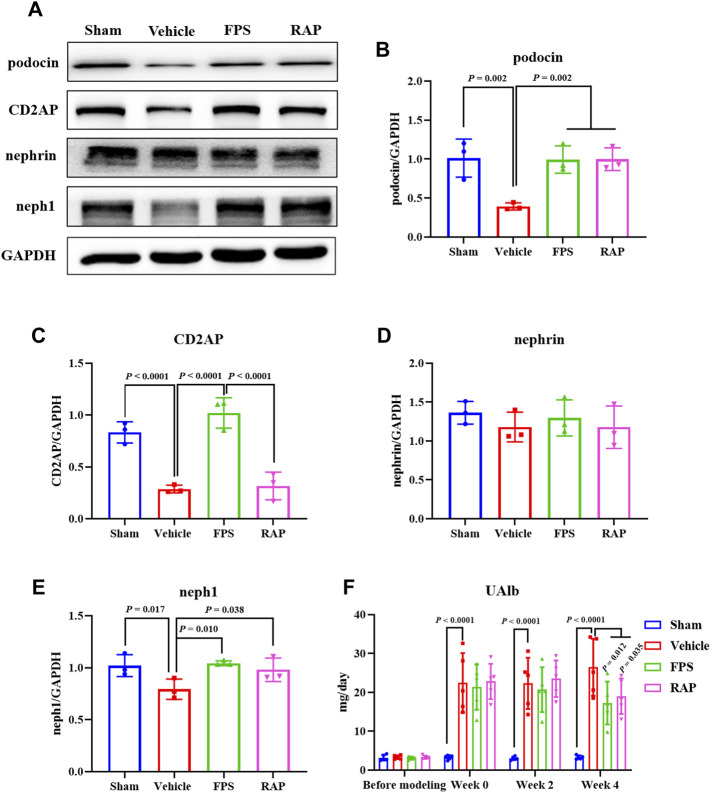
Effects of FPS and RAP on the expression levels of podocin, CD2AP, nephrin, and neph1 *in vivo*, as well as UAlb in the DKD model rats. **(A)** The WB analysis of podocin, CD2AP, nephrin, and neph1 in the diabetic kidneys. **(B–E)** The rate of podocin, CD2AP, neph1, and nephrin to GAPDH, respectively. **(F)** The dynamic change of UAlb after drug intervention during 4 weeks. The data are expressed as the mean ± SD.

**FIGURE 6:**
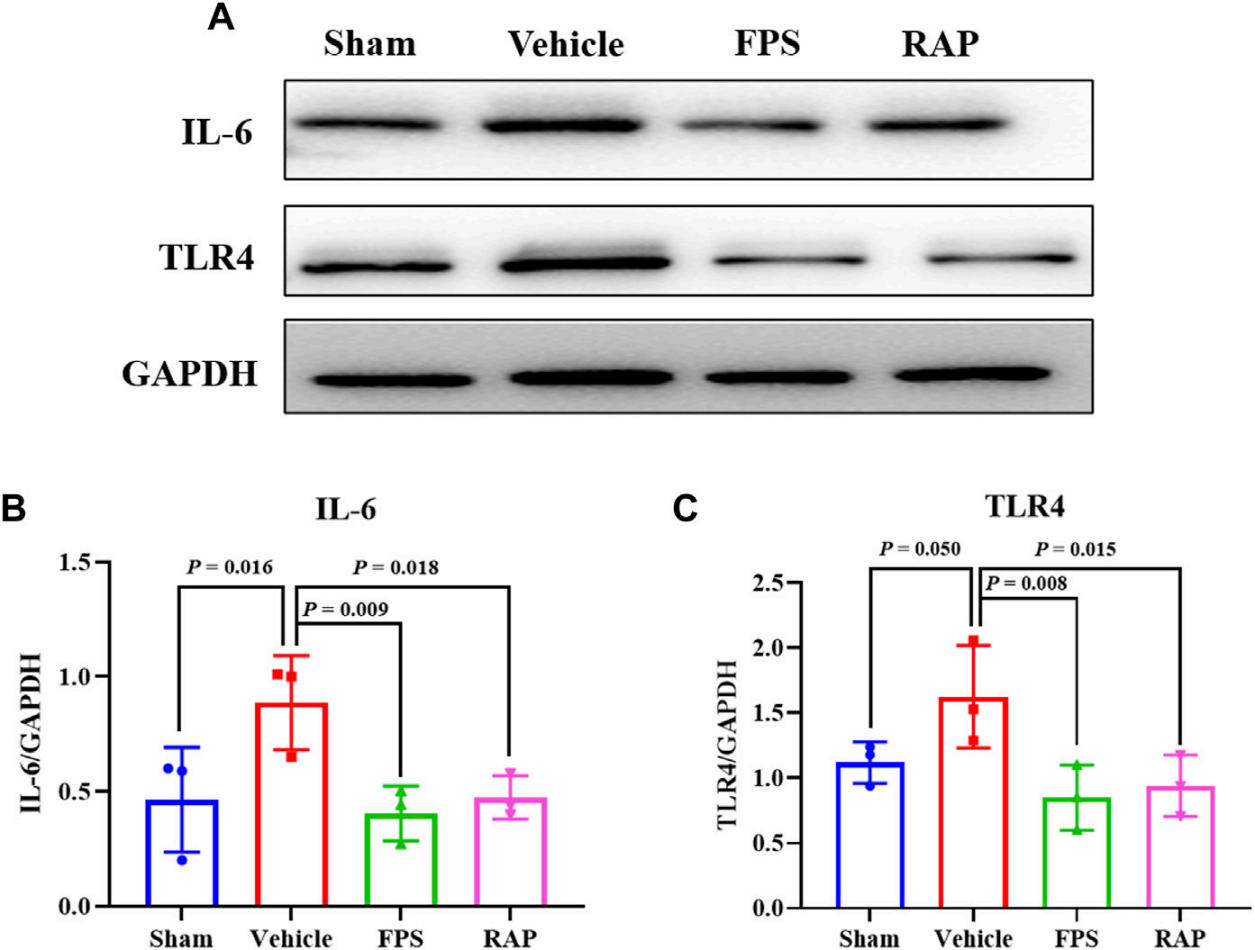
Effects of FPS and RAP on the expression levels of IL-6 and TLR4 *in vivo*. **(A)** The WB analysis of IL-6 and TLR4 in the diabetic kidneys. **(B,C)** The rate of IL-6 and TLR4 to GAPDH, respectively. The data are expressed as the mean ± SD.

We then examined the effects of FPS and RAP on the expression levels of the injurious and inflammatory markers in podocytes, including podocin, CD2AP, nephrin, and neph1, as well as IL-6 and TLR4 in glomeruli and in the kidneys of these DKD model rats by IHC staining and WB analysis. In Figures 4C, 5A, 6A, we found that, compared with the Sham group rats, after modeling, the changed immunostaining extent of podocin and CD2AP in glomeruli, and the altered protein expression levels of podocin, CD2AP, neph1, IL-6, and TLR4 in the kidneys of these DKD model rats were detected, respectively, and significantly improved in the FPS and RAP group rats after treatment with FPS or RAP when compared to the Vehicle group rats. Notably, the expression characteristic of CD2AP in glomeruli and the protein expression level of CD2AP in the kidneys of the FPS group rats were better than those of the RAP group rats (Figures 4E, 5C). However, no significant difference in the protein expression level of nephrin was found among the four groups (Figure 5D). In addition, after treatment with FPS or RAP for 4 weeks, the increased level of UAlb in these DKD model rats was significantly decreased in the FPS and RAP group rats when compared to the Vehicle group rats (Figure 5F).

Taken together, the above results showed that FPS and RAP could significantly reduce inflammatory podocyte injury in the DKD model rats.

### FPS and RAP Attenuate Podocyte Pyroptosis *In Vivo* and *In Vitro*

**FIGURE 7:**
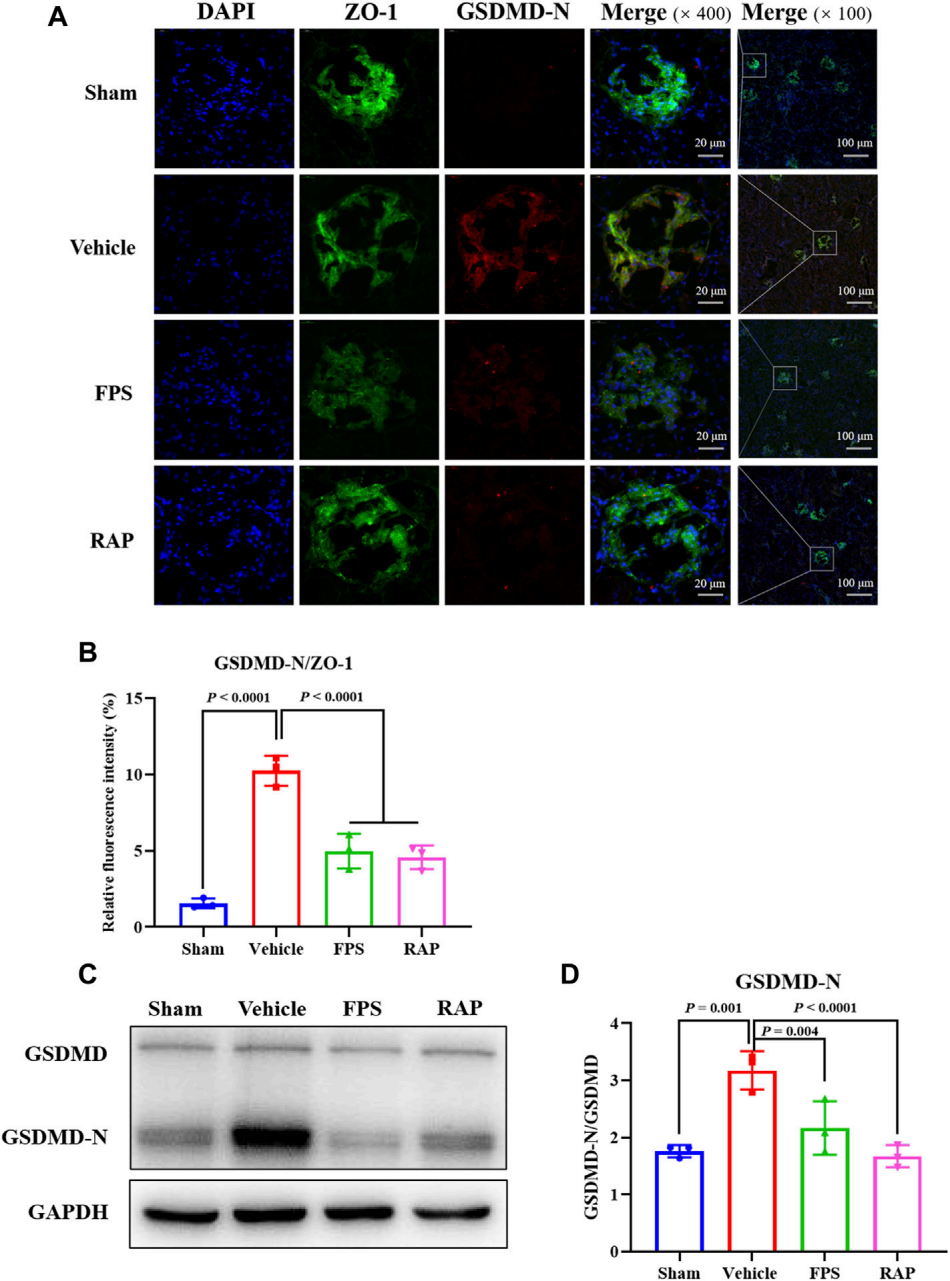
Effects of FPS and RAP on podocyte pyroptosis *in vivo*. **(A)** The immunofluorescent co-localization of ZO-1 and GSDMD-N in glomeruli. Nuclei counterstained with DAPI. Scale bar = 20 and 100 μm. **(B)** The relative fluorescence intensity of GSDMD-N/ZO-1. **(C)** The WB analysis of GSDMD and GSDMD-N in the diabetic kidneys. **(D)** The rate of GSDMD-N to GSDMD. The data are expressed as the mean ± SD.

GSDMD is a universal substrate for inflammatory caspases that play a specific role in inflammatory caspase-initiated pyroptosis (Humphries et al., 2020). As a downstream effector of multiple inflammasomes, GSDMD is hydrolyzed by activated inflammatory caspases, and the released N-terminus of GSDMD (GSDMD-N) translocates to the cell membrane (Xie et al., 2019; Zhu et al., 2020). On the other hand, ZO-1 is a specific scaffolding marker of podocytes (Rincon-Choles et al., 2006). Therefore, we used immunofluorescence co-localization and semi-quantitative analysis to observe whether pyroptosis could occur in podocytes treated with FPS and RAP in glomeruli. In Figure 7A, GSDMD-N mainly co-localized with ZO-1 in glomeruli, indicating that pyroptosis occurred in podocytes. Compared with the Sham group rats, the relative fluorescence intensity of GSDMD-N in podocytes in the Vehicle group rats was significantly stronger. After treatment with FPS or RAP, the increased fluorescence intensity of GSDMD-N in glomeruli of these DKD model rats was significantly decreased in the FPS and RAP group rats, respectively (Figure 7B). On the contrary, the fluorescence intensity of ZO-1 at the same position in glomeruli of the Vehicle, FPS, and RAP group rats appeared the inverse changes, respectively. Accordingly, as shown in Figure 7C, we also found that, after treatment with FPS or RAP, the increased protein expression level of GSDMD-N in the kidneys of these DKD model rats was significantly decreased in the FPS and RAP group rats compared to the Vehicle group rats (Figure 7D). Here, there was no significant changes in the protein expression level of GSDMD in the kidneys among the four groups.

**FIGURE 8:**
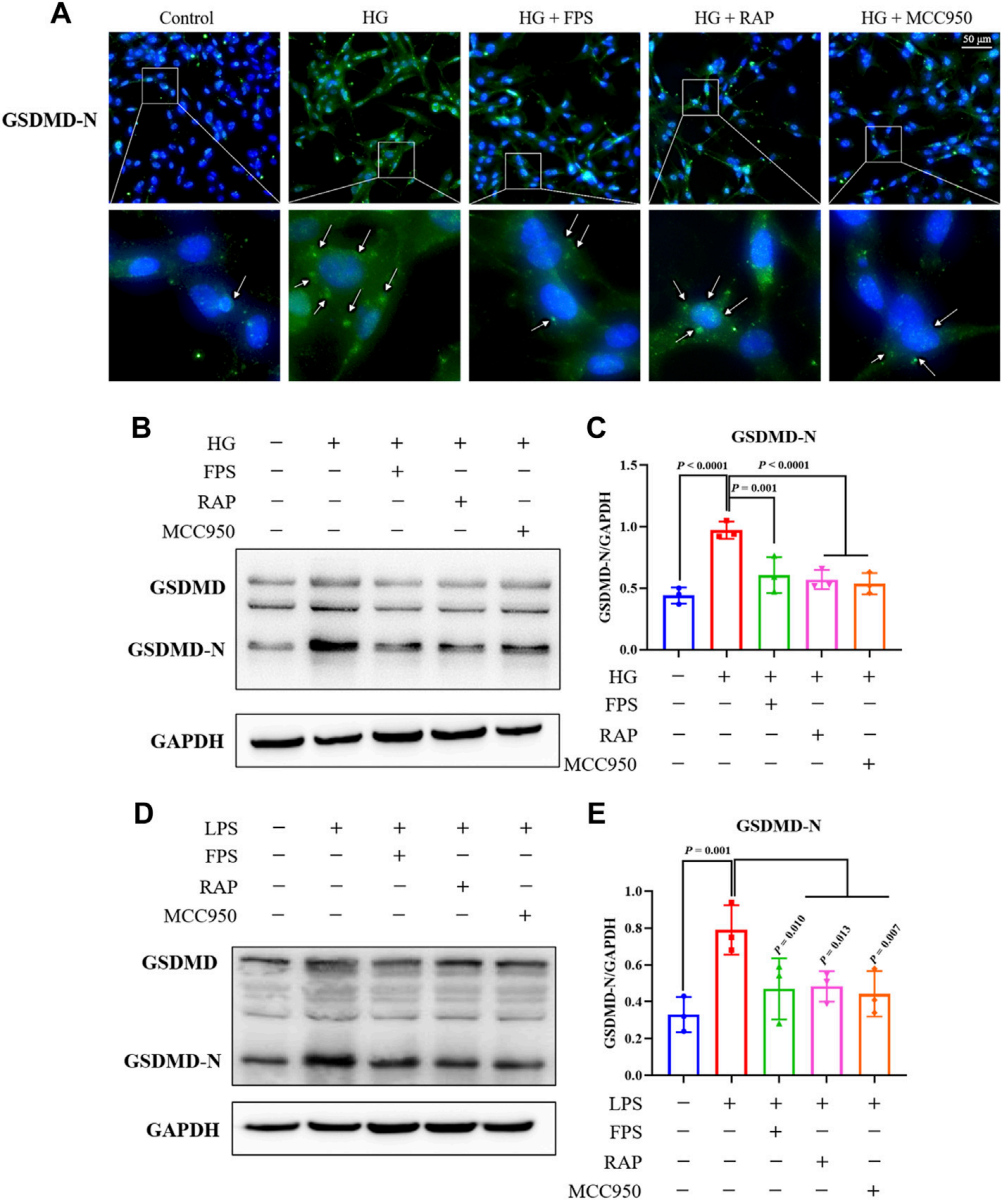
Effects of FPS and RAP on podocyte pyroptosis *in vitro*. **(A)** Immunofluorescent labeling with GSDMD-N. Nuclei counterstained with DAPI. In the locally enlarged lower panel, the arrow shows that GSDMD-N relocated to the cell membrane (×400). Scale bar = 50 μm. **(B)** The WB analysis of GSDMD and GSDMD-N in MPC-5 cells exposed to HG with or without FPS or RAP for 24 h; **(C)** The rate of GSDMD-N to GSDMD. **(D)** The WB analysis of GSDMD and GSDMD-N in MPC-5 cells exposed to LPS with or without FPS or RAP or MCC950 for 24 h; **(E)** The rate of GSDMD-N to GSDMD. The data are expressed as the mean ± SD.

To investigate whether FPS or RAP could reduce podocyte pyroptosis *in vitro*, we examined immunofluorescence staining of GSDMD-N and the protein expression levels of GSDMD and GSDMD-N in podocytes exposed to HG with or without FPS or RAP for 24 h. Prior to the formal cellular experiments, we determined the cytotoxicity of FPS or RAP on podocytes using a CCK-8 kit. As shown in Supplementary Figure S2, we found that the cellular viability of podocytes was significantly reduced when treated with higher concentrations of FPS (25 μg/ml) and RAP (25 nmol/L) when compared to a 20 μg/ml dose of FPS and a 20 nmol/L dose of RAP. According to these results, we selected the safe and effective doses for both FPS (20 μg/ml) and RAP (20 nmol/L). Figure 8A shows that GSDMD-N staining positive podocytes were tagged with the most powerful fluorescence at the cell membrane of the HG-treated alone group when compared to the other groups including the HG + FPS-treated group, the HG + RAP-treated group, and the HG + MCC950 (an NLRP3 inhibitor)-treated group. In addition, the protein expression level of GSDMD-N was significantly higher in the podocytes exposed to HG than in the control cells (Figure 8B). FPS or RAP treatment significantly ameliorated these changes in the podocytes exposed to HG compared to HG treatment alone. Moreover, we also found an improvement in the protein expression level of GSDMD-N in the podocytes exposed to HG and MCC950 when compared to those treated with HG alone (Figure 8C). Here, the inhibitory effects of FPS and MCC950 on the protein expression levels of GSDMD-N in podocytes were very similar. Further, to confirm the effects *in vitro* of FPS or RAP on podocyte pyroptosis under a state of inflammation, we also tested the protein expression levels of GSDMD and GSDMD-N in the podocytes exposed to LPS, an inflammatory inducer, with or without FPS or RAP for 24 h. Interestingly, when compared with the podocytes exposed to HG, we got similar results (Figures 8D,E).

In short, the above results showed that FPS and RAP could significantly attenuate podocyte pyroptosis *in vivo* and *in vitro*.

### FPS and RAP Inhibit NLRP3 Inflammasome Activation *In Vivo* and *In Vitro*

**FIGURE 9:**
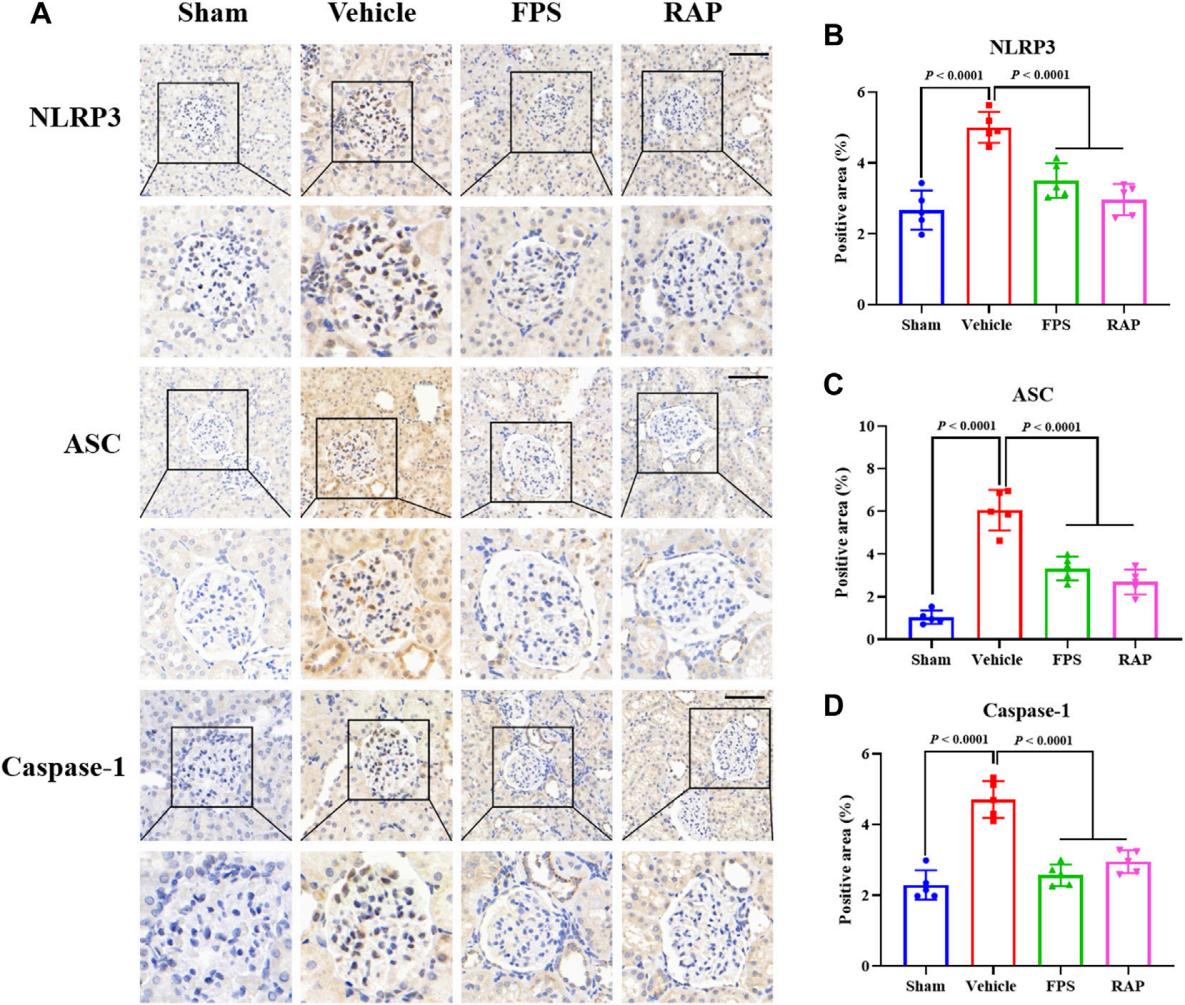
Effects of FPS and RAP on the expression characteristic of NLRP3 inflammasome *in vivo*. **(A)** The immunostaining of NLRP3, ASC, and Caspase-1 in glomeruli (×400). Scale bar = 50 μm. **(B–D)** The positive area of NLRP3, ASC, and Caspase-1, respectively. The data are expressed as the mean ± SD.

**FIGURE 10:**
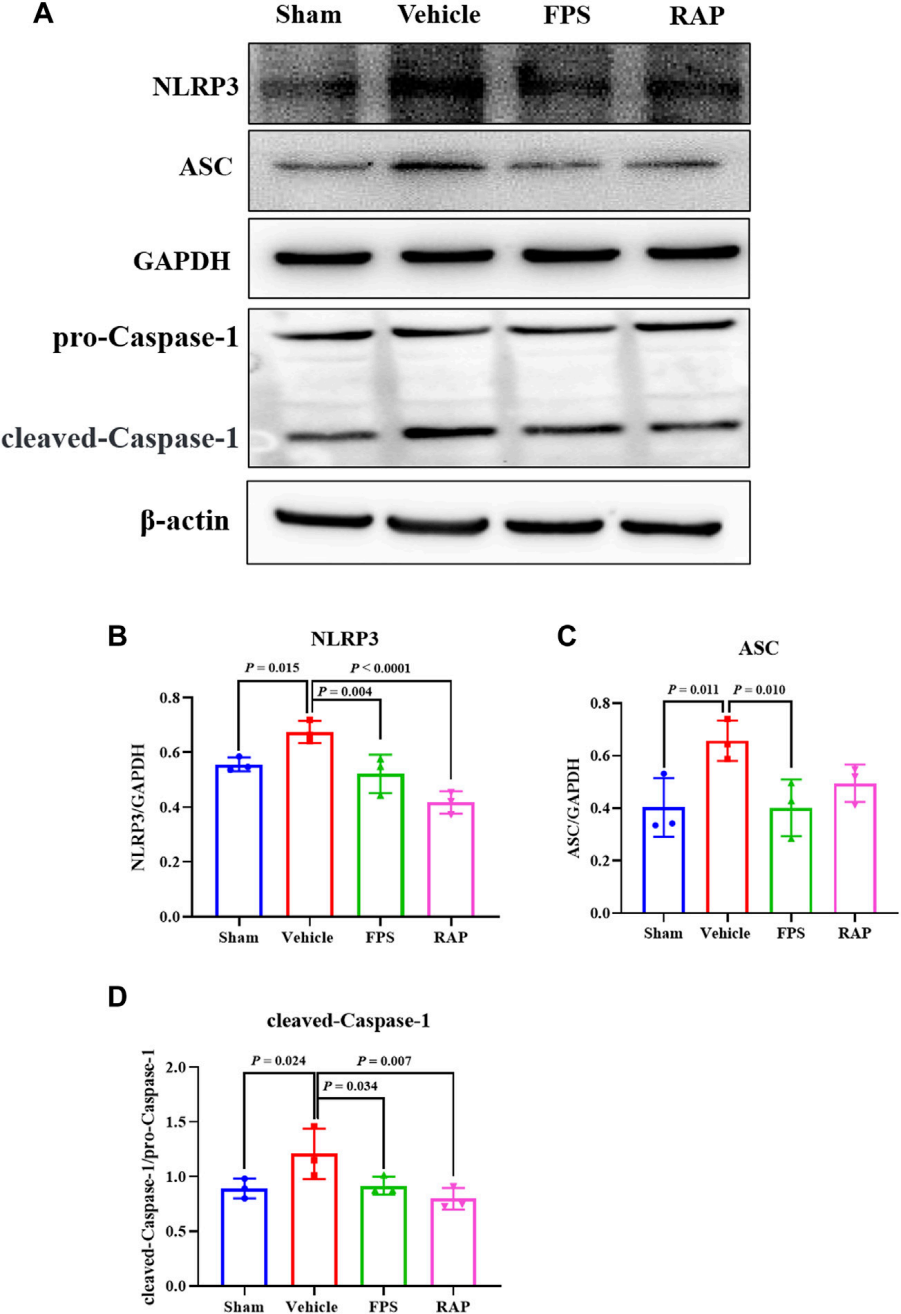
Effects of FPS and RAP on the activation of NLRP3 inflammasome *in vivo*. **(A)** The WB analysis of NLRP3, ASC, pro-Caspase-1, and cleaved-Caspase-1 in the diabetic kidneys. **(B–D)** The rate of NLRP3, ASC, and cleaved-Caspase-1 to GAPDH or pro-Caspase-1, respectively. The data are expressed as the mean ± SD.

NLRP3 inflammasome activation is an important factor that acts upstream of pyroptosis (Xiong et al., 2020). Thus, we evaluated the expression of NLRP3, ASC, and Caspase-1 as the markers of NLRP3 inflammasome activation in glomeruli and in the kidneys of these DKD model rats by IHC staining and WB analysis. In Figure 9A, IHC staining showed that, after treatment with FPS or RAP, the immunostaining of NLRP3, ASC, and Caspase-1 in glomeruli in these DKD model rats were significantly decreased in the FPS and RAP group rats when compared to the Vehicle group rats (Figures 9B–D). In addition, in Figure 10A, WB analysis showed that changes in the protein expression levels of NLRP3, ASC, and Caspase-1 in the kidneys of the Vehicle, FPS, and RAP group rats were consistent with the above-mentioned results of IHC staining (Figures 10B–D).

**FIGURE 11:**
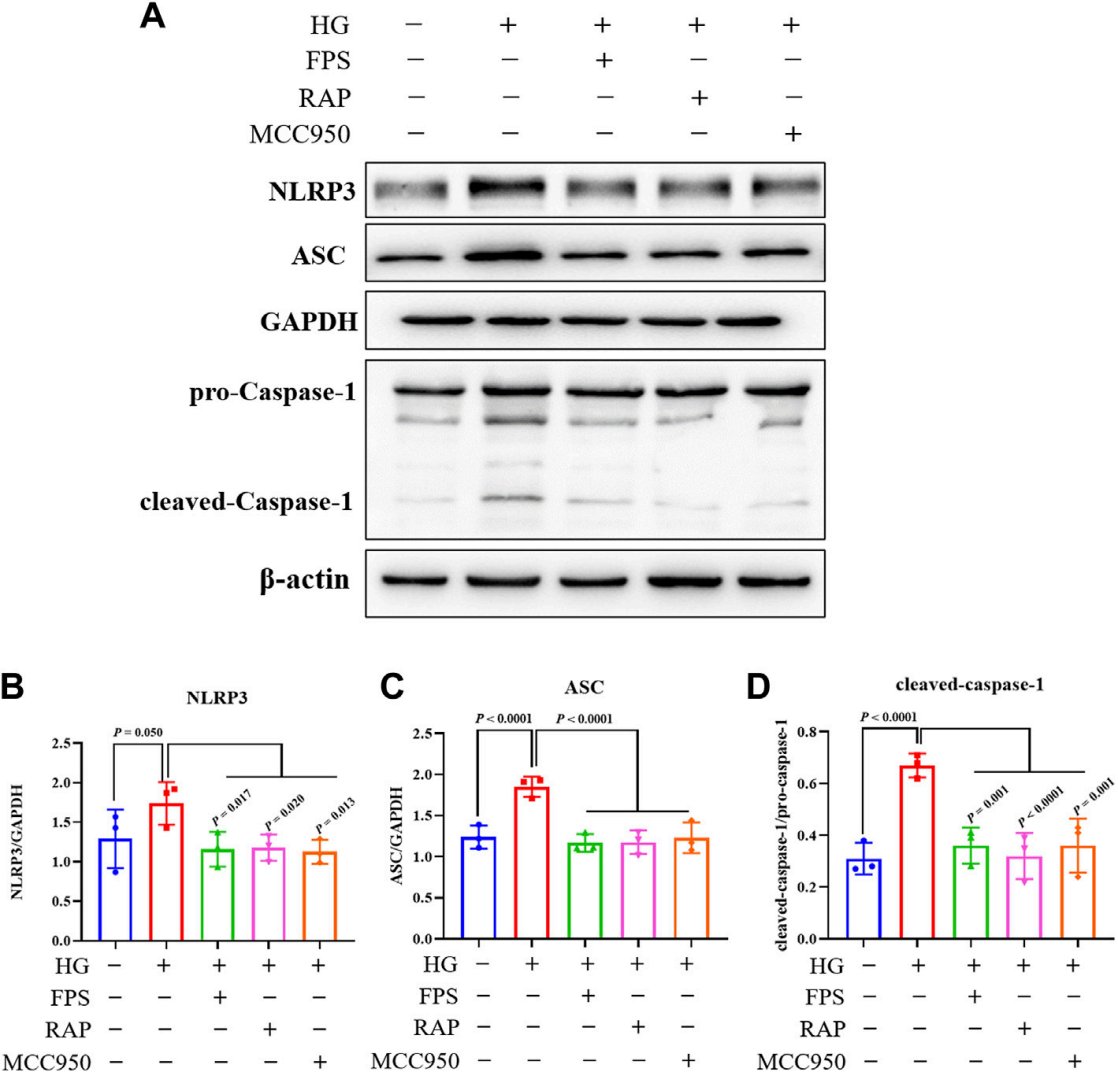
Effects of FPS and RAP on the activation of NLRP3 inflammasome *in vitro*. **(A)** The WB analysis of NLRP3, ASC, pro-Caspase-1, and cleaved-Caspase-1 in MPC-5 cells exposed to HG with or without FPS or RAP or MCC950 for 24 h. **(B–D)** The rate of NLRP3, ASC, and cleaved Caspase-1 to GAPDH or pro-Caspase-1, respectively. The data are expressed as the mean ± SD.

**FIGURE 12:**
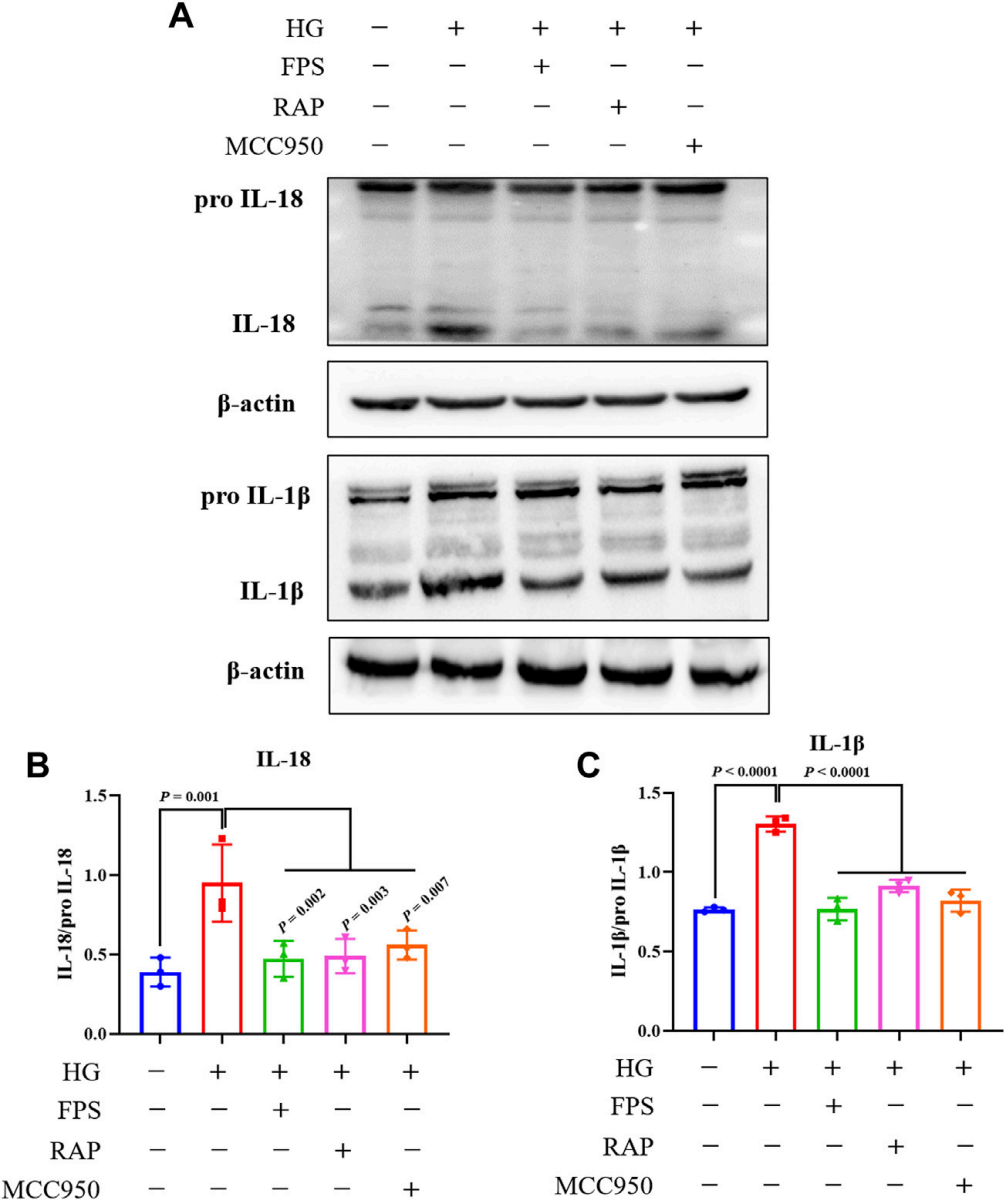
Effects of FPS and RAP on the expression levels of IL-18 and IL-1β *in vitro*. **(A)** The WB analysis of pro IL-18, pro IL-18, pro IL-1β, and IL-1β in MPC-5 cells exposed to HG with or without FPS or RAP or MCC950 for 24 h **(B,C)** The rate of IL-18 and IL-1β to pro IL-18 or pro IL-1β, respectively. The data are expressed as the mean ± SD.

To confirm whether FPS or RAP could inhibit NLRP3 inflammasome activation *in vitro*, we tested the protein expression levels of NLRP3, ASC, Caspase-1, IL-18, and IL-1β in the podocytes exposed to HG with or without FPS or RAP for 24 h, compared to MCC950. Figures 11A, 12A show that, compared with the control cells, the podocytes exposed to HG exhibited significantly higher protein expression levels of NLRP3, ASC, cleaved-Caspase-1, IL-18, and IL-1β. After treatment with FPS or RAP, the podocytes exposed to HG showed a significant reduction in the protein expression levels of NLRP3, ASC, cleaved-Caspase-1, IL-18, and IL-1β when compared to HG treatment alone. In addition, we found a decrease in NLRP3, ASC, cleaved-Caspase-1, IL-18, and IL-1β protein expression levels in the podocytes exposed to HG and MCC950. Here, the inhibitory effects of FPS on NLRP3 inflammasome activation in the podocytes exposed to HG were similar to those in the podocytes treated with MCC950 and HG (Figures 11B–D, 12B‒C). Notably, there was no significant changes in the protein expression levels of pro-Caspase-1, pro IL-1β, and pro IL-18 *in vivo* and *in vitro* under the same treatment.

Overall, the above results showed that FPS and RAP could significantly inhibit NLRP3 inflammasome activation *in vivo* and *in vitro*.

### FPS and RAP Regulate the AMPK/mTORC1/NLRP3 Signaling Axis *In Vivo* and *In Vitro*

**FIGURE 13:**
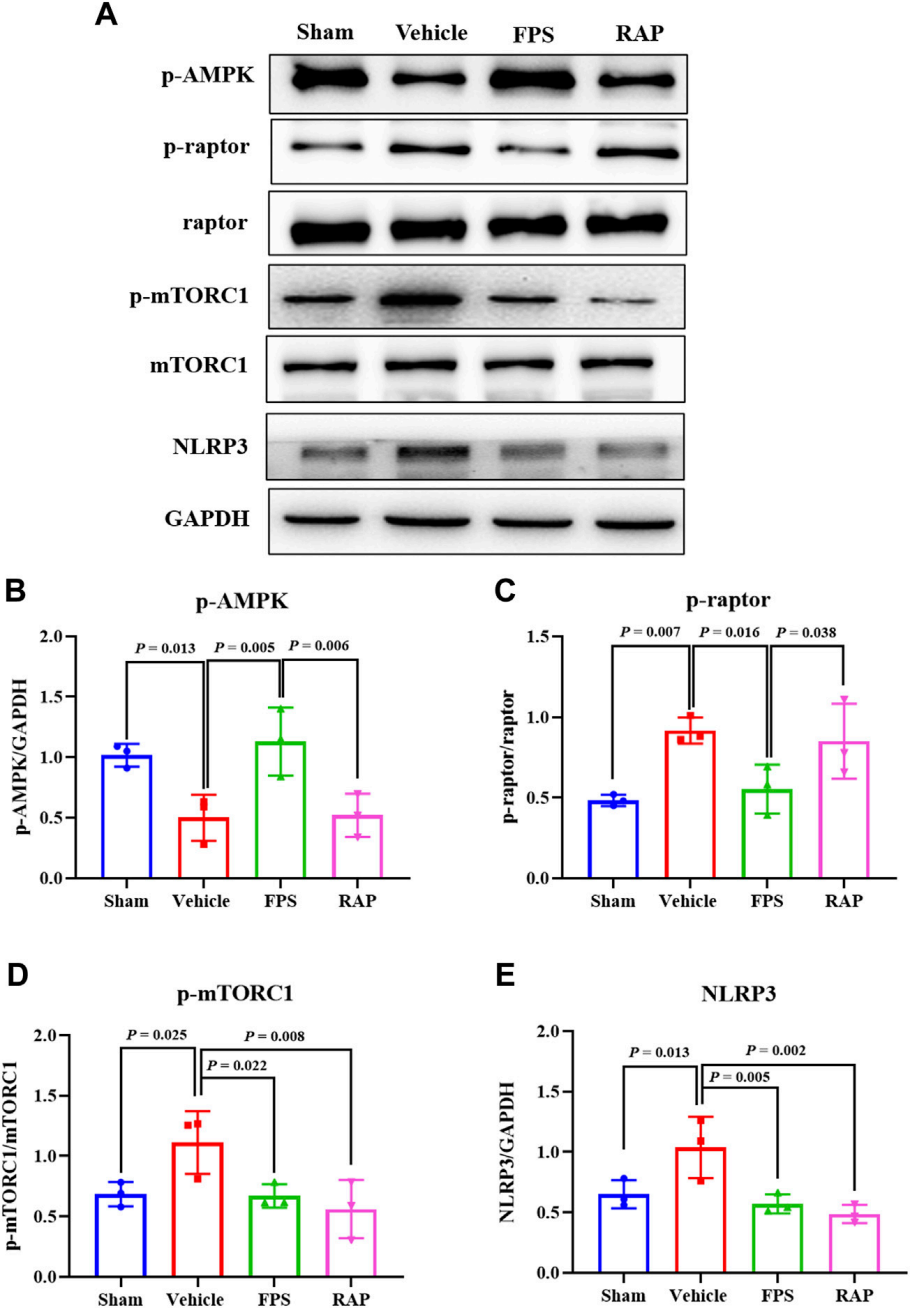
Effects of FPS and RAP on the AMPK/mTORC1/NLRP3 signaling axis *in vivo*. **(A)** The WB analysis of the key signaling molecules in the AMPK/mTORC1/NLRP3 signaling axis in the diabetic kidneys. **(B–E)** The rate of p-AMPK, p-raptor, p-mTORC1, and NLRP3 to GAPDH or raptor or mTORC1, respectively. The data are expressed as the mean ± SD.

The AMPK/mTORC1/NLRP3 signaling axis is an important regulatory mechanism in NLRP3 inflammasome activation (Bullón et al., 2016; Qiu et al., 2016). Thus, we used WB analysis to investigate the protein expression levels of the key signaling molecules of the AMPK/mTORC1/NLRP3 signaling axis in the kidneys of these DKD model rats. Figure 13A shows that the protein expression levels of p-AMPK, p-raptor, p-mTORC1, and NLRP3 in the kidneys of the Vehicle group rats were significantly different from those in the Sham group rats. After treatment with FPS or RAP, changes in the protein expression levels of the above-mentioned key signaling molecules in the kidneys of these DKD model rats were significantly improved in the FPS or RAP group rats when compared to the Vehicle group rats (Figures 13B–E). Notably, the regulative effects of FPS on the protein expression levels of p-AMPK and p-raptor in the kidneys of the FPS group rats were better than those of the RAP group rats (Figures 13B,C).

**FIGURE 14:**
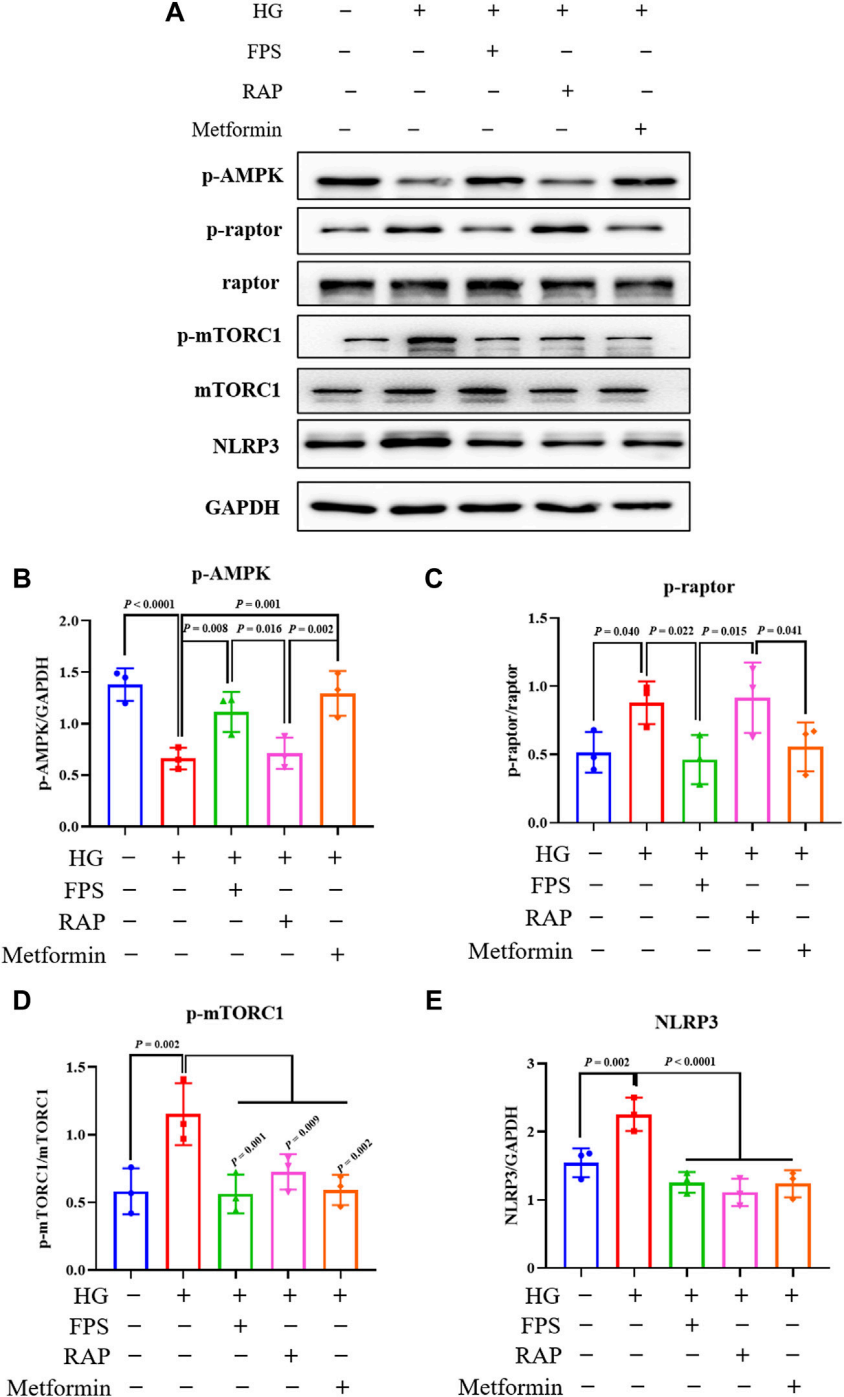
Effects of FPS and RAP on the AMPK/mTORC1/NLRP3 signaling axis *in vitro*. **(A)** The WB analysis of the key signaling molecules in the AMPK/mTORC1/NLRP3 signaling axis in MPC-5 cells exposed to HG with or without FPS or RAP or metformin for 24 h **(B–E)** The rate of p-AMPK, p-raptor, p-mTORC1, and NLRP3 to GAPDH or raptor or mTORC1, respectively. The data are expressed as the mean ± SD.

To determine whether FPS or RAP could regulate the AMPK/mTORC1/NLRP3 signaling axis *in vitro*, we tested the protein expression levels of the key signaling molecules in the AMPK/mTORC1/NLRP3 signaling axis in the podocytes exposed to HG with or without FPS or RAP for 24 h, compared to metformin (an AMPK agonist). Figure 14A shows that the protein expression levels of p-AMPK, p-raptor, p-mTORC1, and NLRP3 in the podocytes exposed to HG were significantly different from those in the control cells. In comparison with HG treatment alone, the protein expression levels of p-AMPK, p-raptor, p-mTORC1, and NLRP3 in the podocytes exposed to FPS or RAP were all significantly reversed. In addition, we also found an increase in p-AMPK protein expression level and a decrease in p-raptor, p-mTORC1, and NLRP3 protein expression levels in the podocytes exposed to HG and metformin. Here, notably, the regulative effects of FPS on the protein expression levels of p-AMPK and p-raptor in the podocytes exposed to HG were better than those treated with HG and RAP, and similar to those treated with HG and metformin (Figures 14B,C).

In a nutshell, the above results showed that FPS and RAP could significantly regulate the AMPK/mTORC1/NLRP3 signaling axis *in vivo* and *in vitro.*

## Discussion

In the progression of DKD, RF is one of the main underlying causes of end-stage kidney disease. Podocyte injury is involved in almost all pathological changes in diabetes mellitus (DM)-related glomerular diseases, namely GS (Alicic et al., 2017; Lu et al., 2019). A series of pathological changes in podocyte injury in DKD such as podocyte hypertrophy, EMT, detachment, apoptosis, and autophagy has been conformed, respectively (Zhang et al., 2020). In recent years, emerging evidence has shown that podocyte pyroptosis plays a significant role in the pathological mechanisms of inflammation-derived podocyte injury (Lin et al., 2020). Therefore, in this study, we first attempted to establish a modified DKD rat model accompanied by GS and inflammatory podocyte injury. Our results showed that these modified DKD model rats not only exhibited stable hyperglycemia and abnormal levels of UAlb, Scr, and BUN, but also had typical GS characteristics and podocyte injurious features, especially widened, shortened, and fused foot processes. Furthermore, significant changes in the expression of podocyte injurious markers, such as podocin, CD2AP, nephrin, and nephr1, as well as inflammatory markers, including IL-6 and TLR4, in the kidneys of the DKD model rats were detected. Therefore, we considered that these modified rat models of DKD induced by uninephrectomy, STZ intraperitoneal injection, and a high-fat diet should be helpful in understanding the mechanisms underlying inflammatory podocyte injury-related RF and to identify novel therapeutic targets for RF in DKD.

Previous studies have shown that FPS, as a natural anti-inflammatory phytomedicine, protects the kidney from dysfunction and fibrogenesis by inhibiting the TGF-β pathway and has the potential to slow down the progression of STZ-induced DKD (Chen et al., 2015). Accordingly, in this study, using the modified DKD model rats, we found that FPS and RAP not only reduced glomerular cell proliferation and ECM deposition, but also improved the expression levels of podocin, CD2AP, nephrin, nephr1, IL-6, and TLR4 in diabetic kidneys. In addition, FPS and RAP could inhibit the TGF-β1/Smad2/3 signaling pathway in the kidneys of the DKD model rats. Thus, we concluded that FPS, similar to RAP, can alleviate RF and inflammatory podocyte damage *in vivo*, which are the main injurious characteristics of the kidneys in DKD.

Pyroptosis is a newly discovered type of programmed cell death. The essence of pyroptosis is the activation of NLRP3 inflammasome, which mediates GSDMD and rapidly causes cell membrane rupture and cell content release, leading to an inflammatory response (Galluzzi et al., 2012; Galluzzi et al., 2018). In the pyroptosis pathway, GSDMD is cleaved by inflammatory caspases at an aspartate site within the linking loop, thereby generating the GSDMD-C terminal and the active group GSDMD-N terminal (Xie et al., 2019; Zhu et al., 2020). In the progression of DKD, GSDMD-N acts as a perforating protein, forming pores in proper renal cells, gradually triggering cell death, releasing pro-inflammatory factors, such as IL-1β and IL-18, and aggravating RF, including GS and renal tubulointerstitial fibrosis (Gu et al., 2019). In this study, we first examined a range of pyroptosis markers and inflammasome component proteins in diabetic kidneys to investigate whether pyroptosis could occur in podocytes treated with FPS and RAP *in vivo*. Immunofluorescence co-localization and semi-quantitative analysis showed that, after treatment with FPS or RAP, the relative fluorescence intensity of GSDMD-N in glomeruli and the increased protein expression level of GSDMD-N in the kidneys of the DKD model rats were significantly decreased. Furthermore, in the *in vitro* study, we also found that FPS or RAP treatment significantly reduced the number of GSDMD-N staining positive podocytes and ameliorated the higher protein expression level of GSDMD-N in podocytes exposed to HG, and that the inhibitory effects of FPS and MCC950 (an NLRP3 inflammasome inhibitor) on GSDMD-N *in vitro* were very similar. Therefore, we considered that podocyte pyroptosis occurs in glomeruli of the modified DKD rat model, and FPS, similar to RAP, can impart anti-podocyte pyroptosis effects *in vivo* and *in vitro*.

It is well known that a variety of exogenous stimuli can promote NLRP3 inflammasome activation. Two canonical steps were considered to occur. First, microbial molecules or endogenous factors promote the expression of NLRP3, pro IL-1β, and pro IL-18 via the NF-κB signaling pathway (Bauernfeind et al., 2009). Second, these stimuli induce oligomerization and activation of NLRP3 and recruitment of the adapter protein ASC and pro-Caspase-1, the latter of which undergoes autoproteolytic cleavage into Caspase-1 to activate pro IL-1β and pro IL-18 to produce the active cytokines (Martinon et al., 2002; Lamkanfi and Dixit, 2014; Man and Kanneganti, 2015). It has been reported that NLRP3 inflammasome not only mediates inflammatory response but is also related to pyroptosis in RF during DKD progression (Zhang and Wang, 2020). In this study, IHC staining and WB analysis showed that the increased immunostaining of NLRP3, ASC, and Caspase-1 in glomeruli and the higher protein expression levels of NLRP3, ASC, and cleaved-Caspase-1 in the kidneys of the DKD model rats were significantly decreased after treatment with FPS or RAP. Likewise, in the *in vitro* study, we also found that, after treatment with FPS or RAP, the podocytes exposed to HG showed a significant reduction in the protein expression levels of NLRP3, ASC, cleased-Caspase-1, IL-18, and IL-1β. In addition, the inhibitory effects of FPS on NLRP3 inflammasome activation in the podocytes exposed to HG were similar to those in the podocytes treated with MCC950 and HG. Hence, we concluded that NLRP3 inflammasome in the diabetic kidneys of the modified DKD rat model is activated, and FPS, similar to RAP, can inhibit NLRP3 inflammasome activation *in vivo* and *in vitro*.

Previous research has shown that mTOR is widely distributed in renal cortex and medulla of patients with DKD, in which mTORC1 activation plays a key regulatory role in podocyte injury (Lu et al., 2011). In addition, AMPK, an important metabolic stress protein kinase, can be activated by stimulation of numerous hormones, adipokines, and cytokines in DKD (Sanders et al., 2007). Under HG conditions, the phosphorylation level of AMPK decreases and its activation is inhibited. Upstream AMPK inhibits mTORC1 activation and induces autophagy through phosphorus acidification of raptor-related regulatory proteins (Kim et al., 2011). Ding et al. (2010) reported that resveratrol, an AMPK activator, reduces podocyte injury by restoring AMPK activation in STZ-induced diabetic rats. Jin et al. (2017) also reported that berberine reduces HG-induced podocyte damage by enhancing AMPK activation. In this study, our *in vivo* and *in vitro* data clearly indicated that the protein expression levels of p-AMPK, p-raptor, p-mTORC1, and NLRP3 in the kidneys of the DKD model rats and in the cultured podocytes exposed to HG both revealed significant changes. After treatment with FPS or RAP, changes in the protein expression levels of the above-mentioned key signaling molecules *in vivo* and *in vitro* were significantly improved. More importantly, we found that the regulative effects of FPS on p-AMPK and p-raptor *in vivo* and *in vitro* were better than those of RAP. Consequently, we concluded that FPS, in contrast to RAP, can regulate the AMPK/mTORC1/NLRP3 signaling axis *in vivo* and *in vitro.*

Finally, it is important to discuss certain additional points. First, FPS as a natural anti-inflammatory phytomedicine alleviated RF in DKD without affecting hyperglycemia. Second, to exclude the side effects of FPS on the liver, we observed the histological characteristics of liver tissue and biochemical indices of liver function in the DKD model rats after treatment with FPS for 4 weeks. As shown in Supplementary Figure S3, we did not find that FPS has any obvious side effects on the liver. Third, it is important to identify the specific targets of FPS that possess the ability to attenuate podocyte pyroptosis. Although FPS was shown to inhibit NLRP3 inflammasome activation and regulate the AMPK/mTORC1/NLRP3 signaling axis *in vivo* and *in vitro*, we were unable to identify the specific molecules involved in the pyroptosis pathway in this study. Future studies should be devoted to the *in vitro* experiments, further clarifying the precise targets of treating podocyte pyroptosis in DKD.

## Conclusion

**FIGURE 15:**
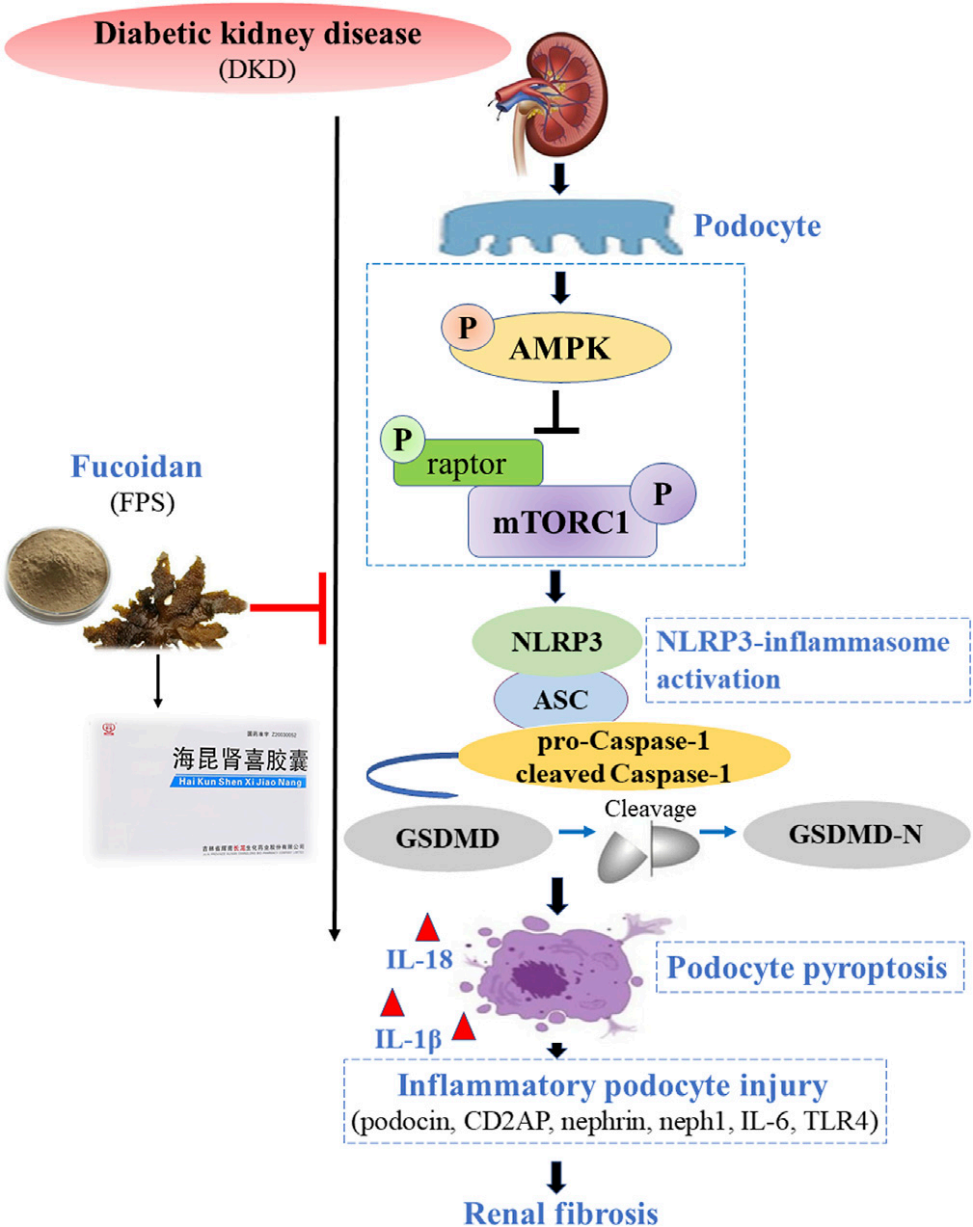
Overview of the effects of FPS on RF in DKD via inhibition of NLRP3 inflammasome-mediated podocyte pyroptosis.

In this study, we confirmed that FPS can alleviate RF in DKD in a manner similar to RAP by inhibiting NLRP3 inflammasome-mediated podocyte pyroptosis via regulation of the AMPK/mTORC1/NLRP3 signaling axis in the diabetic kidney (Figure 15). Our findings provide an in-depth understanding of the pathogenesis of RF, which will aid in identifying precise targets that can be used for DKD treatment.

## Data Availability

The original contributions presented in the study are included in the article/Supplementary Material, further inquiries can be directed to the corresponding authors.

## Glossary

ALT: alanine transaminase
AMPK: adenosine monophosphate-activated protein kinase
ASC: C-terminal caspase recruitment domain
AST: aspartate transaminase
BG: blood glucose
BUN: blood urea nitrogen
BW: body weight
collagen I: collagen type I
DKD: diabetic kidney disease
ECM: extracelluar matrix
FN: fibronectin
FPS: Fucoidan
GBM: glomerular basement membrane
GCP: glomerular celluar population
GS: glomerular sclerosis
GSDMD: gasdermin D
GSDMD-N: Nterminus of GSDMD
GAPDH: glyceraldehyde-3-phosphate dehydrogenase
HG: high glucose
HRP: horse radish peroxidase
IHC: immunohistochemistry
IL: interleukin
IPP: Image-Pro Plus
LPS: lipopolysaccharide
mTOR: mammalian target of rapamycin
mTORC1: mTOR complex 1
NLRP3: nucleotide-binding oligomerization domain (Nod)-like receptor family pyrin domain-containing 3
p-AMPK: phosphorylated AMPK
PAS: Periodic acid-Schiff
p-mTOR: phosphorylated mTOR
p-raptor: phosphorylated raptor
RAP: Rapamycin
RF: renal fibrosis
Scr: serum creatinine
STZ: Streptozotocin
TGF-β1: transforming growth factor-β1
TSA: tyramide signal amplification
UAlb: 24 h urinary albumin
WB: Western blotting
