# Supplementary figures and images for: Fucoidan Alleviates Renal Fibrosis in Diabetic Kidney Disease via Inhibition of NLRP3 Inflammasome-Mediated Podocyte Pyroptosis (part 1 of 3)
Source: Front Pharmacol. 2022 Mar 18;13:790937. doi: 10.3389/fphar.2022.790937 (PMC8972405; doi:10.3389/fphar.2022.790937)

Smad2/3

Original  
image 1,2

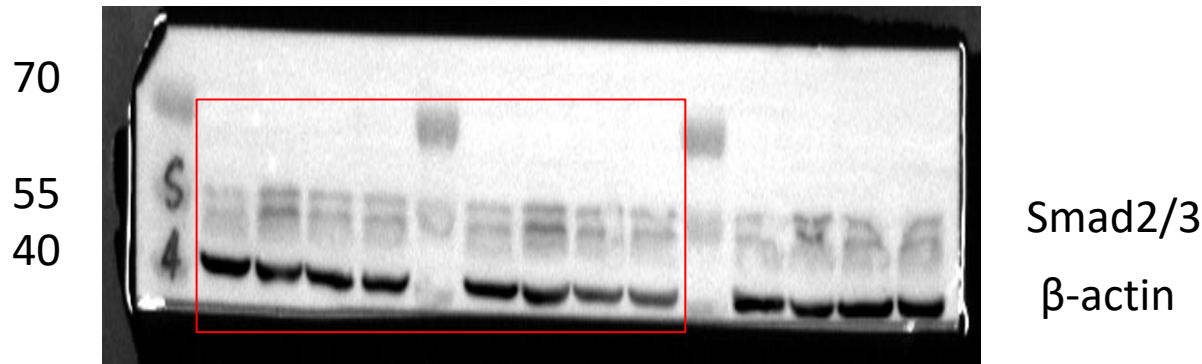

Original  
image 3

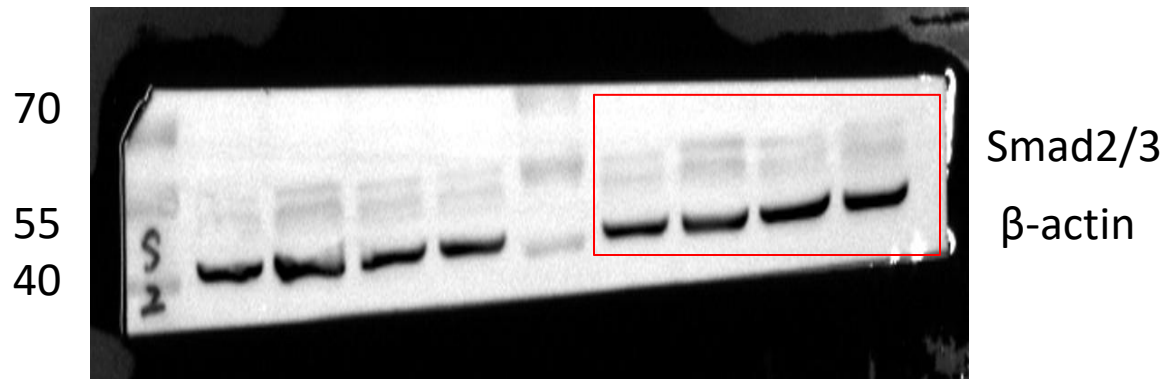

TGF-β1

Original  
image 1

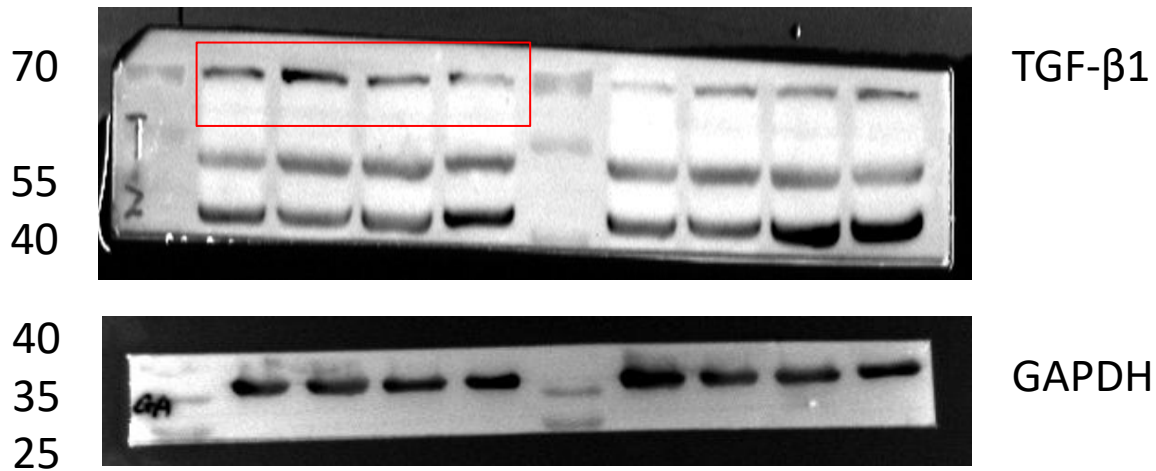

Original  
image 2,3

70

55

40

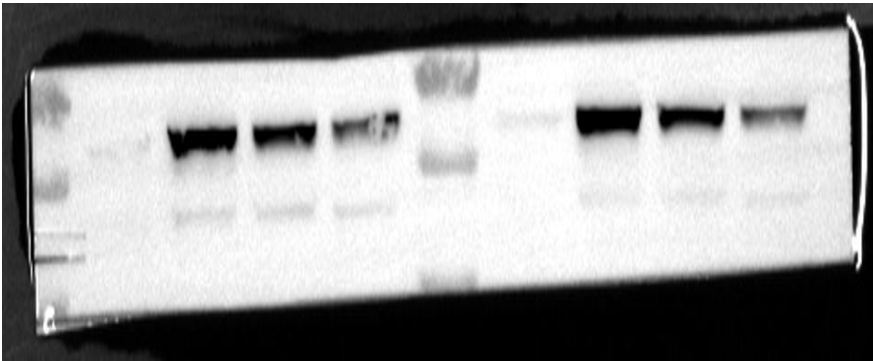

TGF-β1

40

35

25

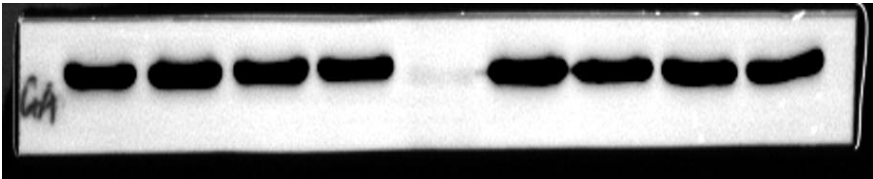

GAPDH

Supplement: Supplementary file 1 [file DataSheet3.ZIP › Original data of Figure 3/Figure 3A-Smad2-3 beta-actin TGF-beta-1 GAPDH-original image-1-3.pdf]

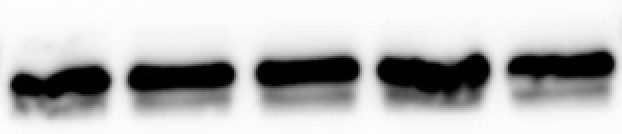

Supplement: Supplementary file 2 [file DataSheet14.ZIP › Original data of Figure 14/Figure 14A-GAPDH-1.jpg]

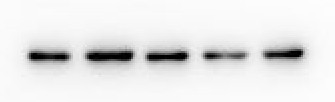

Supplement: Supplementary file 2 [file DataSheet14.ZIP › Original data of Figure 14/Figure 14A-GAPDH-2.jpg]

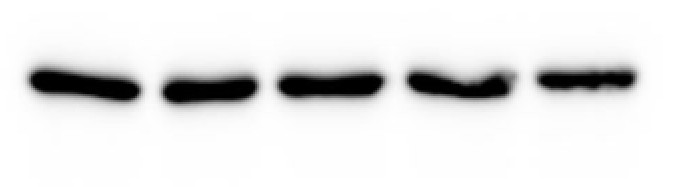

Supplement: Supplementary file 2 [file DataSheet14.ZIP › Original data of Figure 14/Figure 14A-GAPDH-3.jpg]

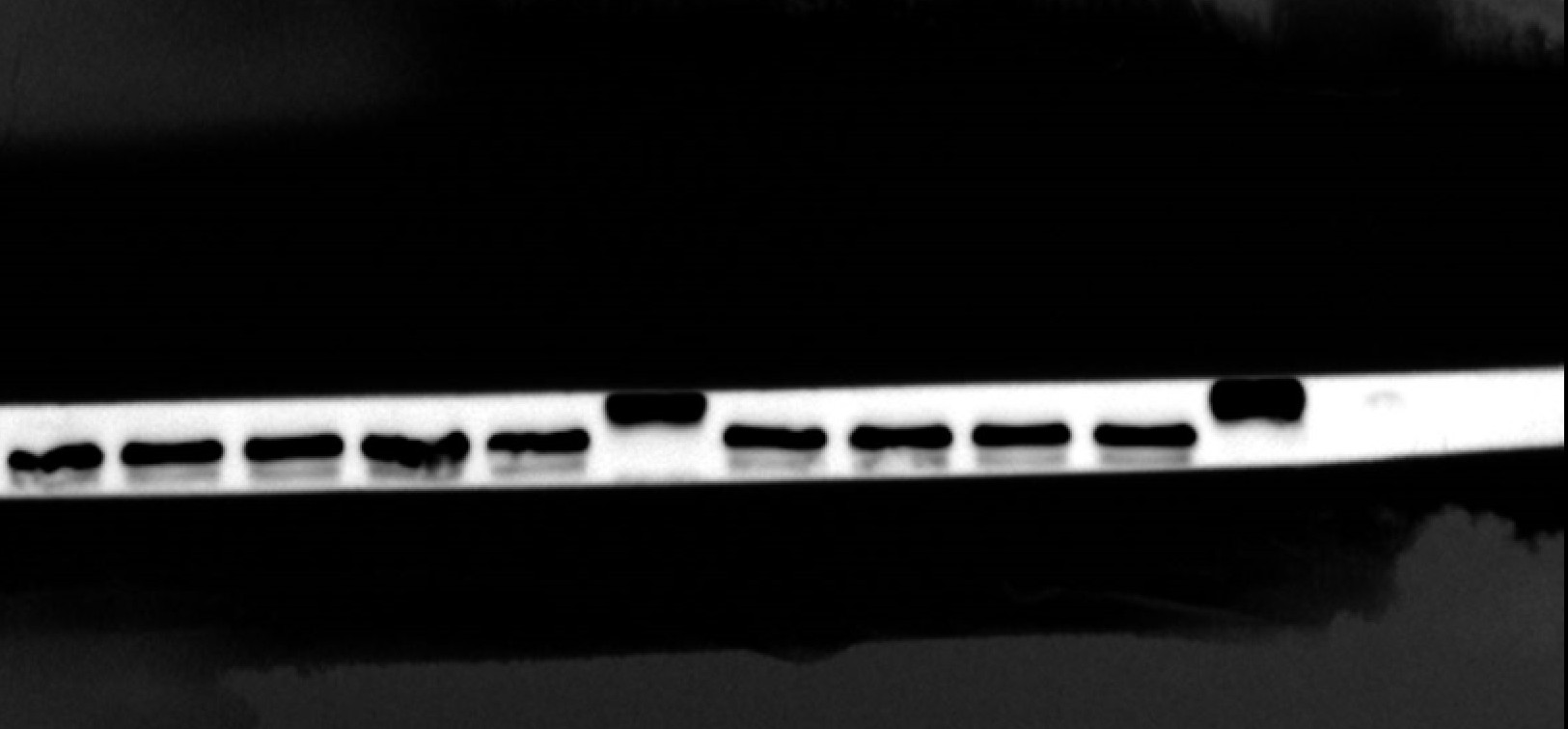

Supplement: Supplementary file 2 [file DataSheet14.ZIP › Original data of Figure 14/Figure 14A-GAPDH-original image-1.jpg]

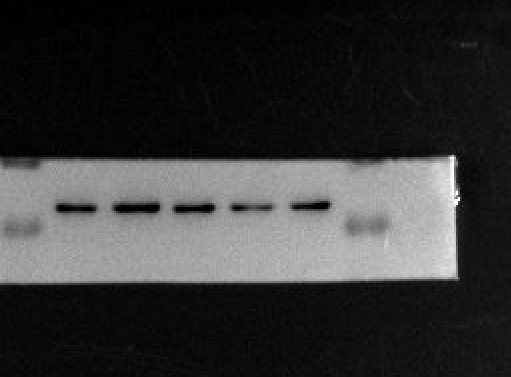

Supplement: Supplementary file 2 [file DataSheet14.ZIP › Original data of Figure 14/Figure 14A-GAPDH-original image-2.jpg]

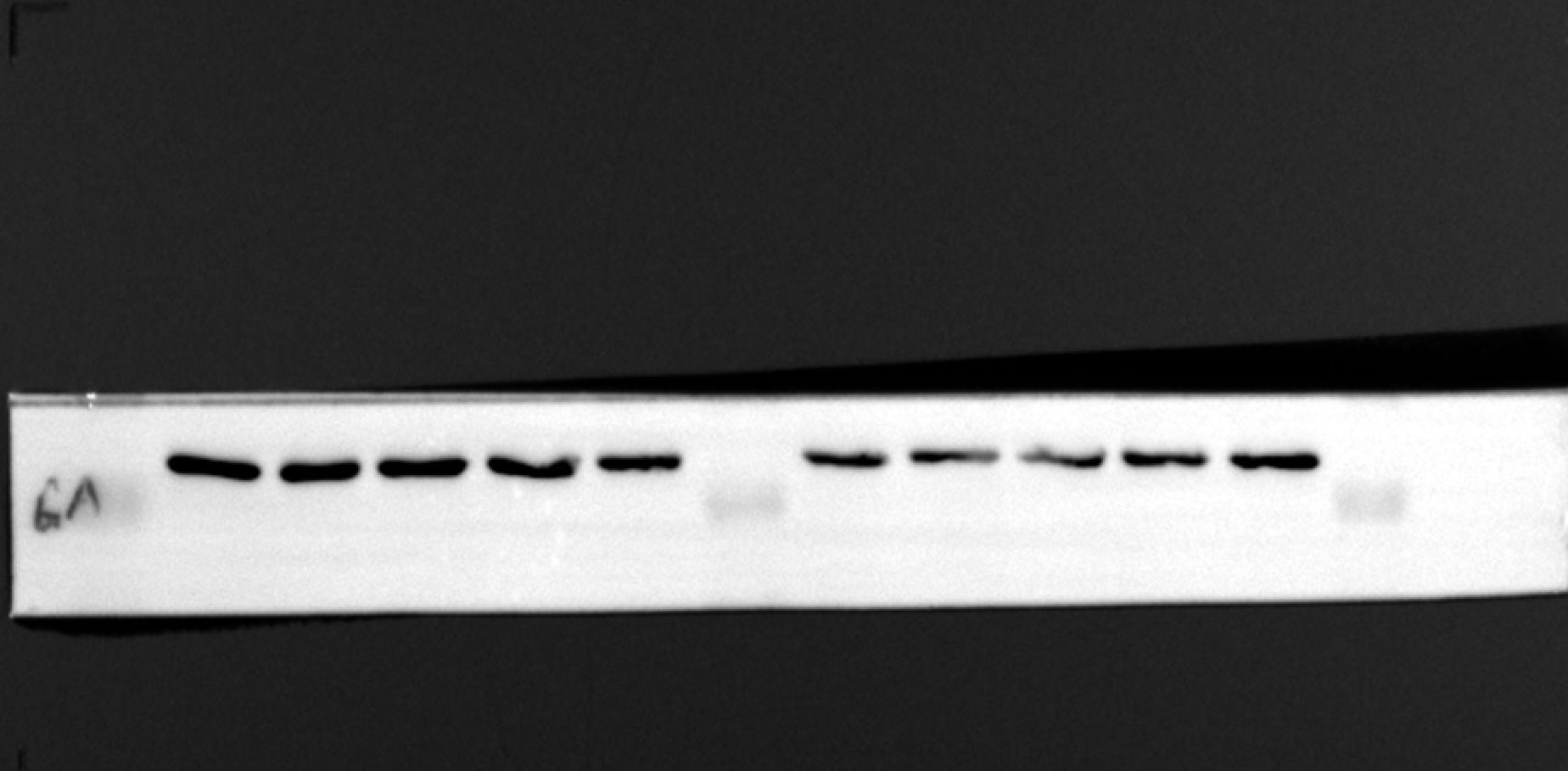

Supplement: Supplementary file 2 [file DataSheet14.ZIP › Original data of Figure 14/Figure 14A-GAPDH-original image-3.jpg]

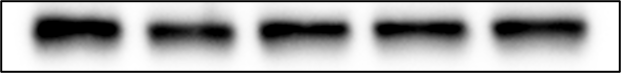

Supplement: Supplementary file 2 [file DataSheet14.ZIP › Original data of Figure 14/Figure 14A-mTORC1-1.tif]

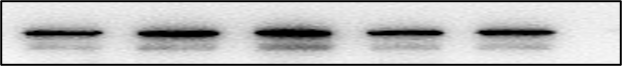

Supplement: Supplementary file 2 [file DataSheet14.ZIP › Original data of Figure 14/Figure 14A-mTORC1-2.tif]

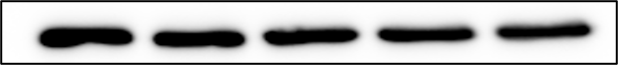

Supplement: Supplementary file 2 [file DataSheet14.ZIP › Original data of Figure 14/Figure 14A-mTORC1-3.tif]

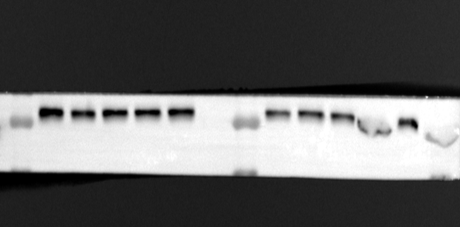

Supplement: Supplementary file 2 [file DataSheet14.ZIP › Original data of Figure 14/Figure 14A-mTORC1-original image-1.tif]

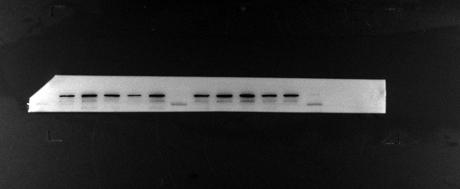

Supplement: Supplementary file 2 [file DataSheet14.ZIP › Original data of Figure 14/Figure 14A-mTORC1-original image-2.tif]

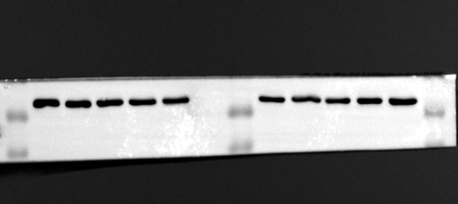

Supplement: Supplementary file 2 [file DataSheet14.ZIP › Original data of Figure 14/Figure 14A-mTORC1-original image-3.tif]

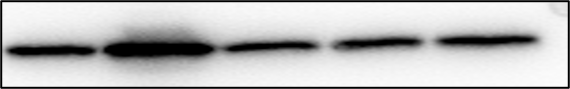

Supplement: Supplementary file 2 [file DataSheet14.ZIP › Original data of Figure 14/Figure 14A-NLRP3-1.tif]

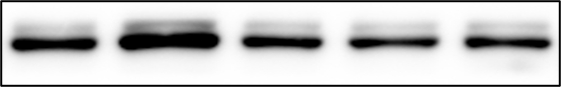

Supplement: Supplementary file 2 [file DataSheet14.ZIP › Original data of Figure 14/Figure 14A-NLRP3-2.tif]

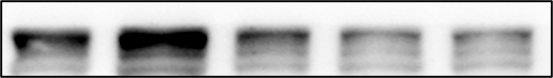

Supplement: Supplementary file 2 [file DataSheet14.ZIP › Original data of Figure 14/Figure 14A-NLRP3-3.tif]

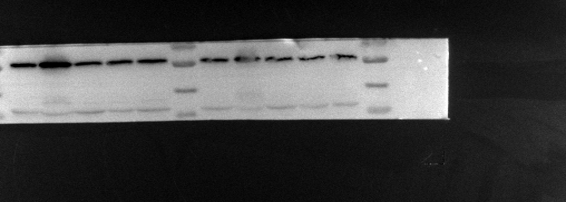

Supplement: Supplementary file 2 [file DataSheet14.ZIP › Original data of Figure 14/Figure 14A-NLRP3-original image-1.tif]

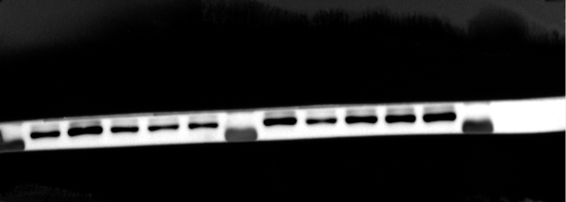

Supplement: Supplementary file 2 [file DataSheet14.ZIP › Original data of Figure 14/Figure 14A-NLRP3-original image-2.tif]

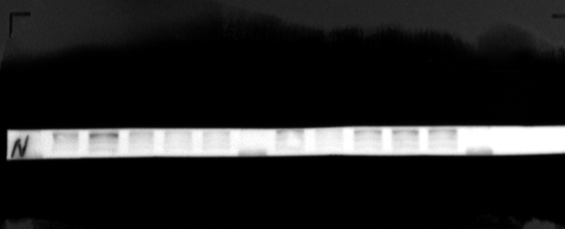

Supplement: Supplementary file 2 [file DataSheet14.ZIP › Original data of Figure 14/Figure 14A-NLRP3-original image-3.tif]

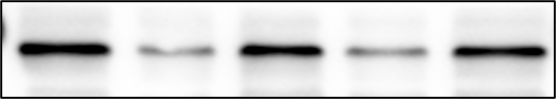

Supplement: Supplementary file 2 [file DataSheet14.ZIP › Original data of Figure 14/Figure 14A-p-AMPK-1.tif]

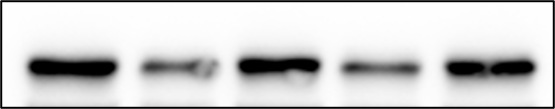

Supplement: Supplementary file 2 [file DataSheet14.ZIP › Original data of Figure 14/Figure 14A-p-AMPK-2.tif]

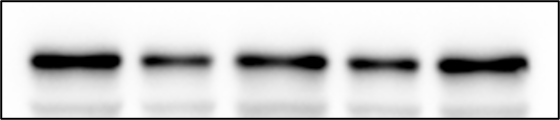

Supplement: Supplementary file 2 [file DataSheet14.ZIP › Original data of Figure 14/Figure 14A-p-AMPK-3.tif]

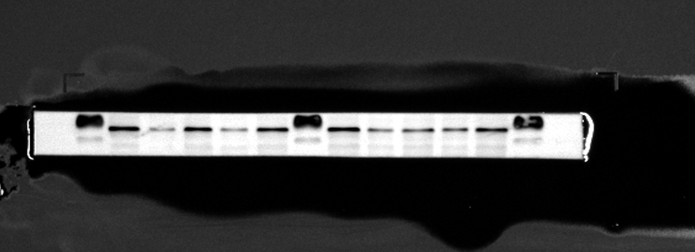

Supplement: Supplementary file 2 [file DataSheet14.ZIP › Original data of Figure 14/Figure 14A-p-AMPK-original image-1.tif]

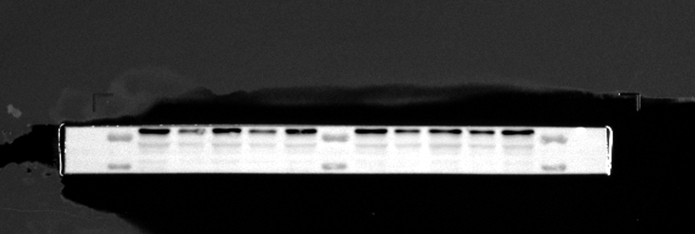

Supplement: Supplementary file 2 [file DataSheet14.ZIP › Original data of Figure 14/Figure 14A-p-AMPK-original image-2.tif]

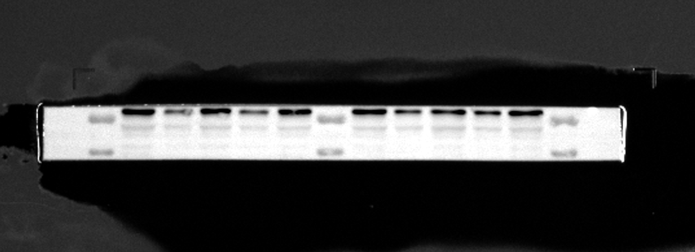

Supplement: Supplementary file 2 [file DataSheet14.ZIP › Original data of Figure 14/Figure 14A-p-AMPK-original image-3.tif]

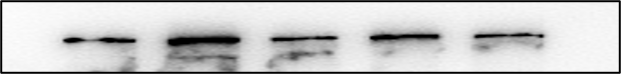

Supplement: Supplementary file 2 [file DataSheet14.ZIP › Original data of Figure 14/Figure 14A-p-mTORC1-1.tif]

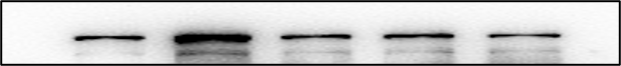

Supplement: Supplementary file 2 [file DataSheet14.ZIP › Original data of Figure 14/Figure 14A-p-mTORC1-2.tif]

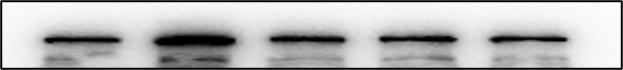

Supplement: Supplementary file 2 [file DataSheet14.ZIP › Original data of Figure 14/Figure 14A-p-mTORC1-3.tif]

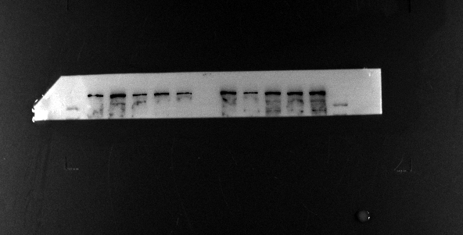

Supplement: Supplementary file 2 [file DataSheet14.ZIP › Original data of Figure 14/Figure 14A-p-mTORC1-original image-1.tif]

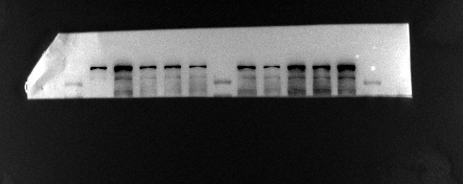

Supplement: Supplementary file 2 [file DataSheet14.ZIP › Original data of Figure 14/Figure 14A-p-mTORC1-original image-2.tif]

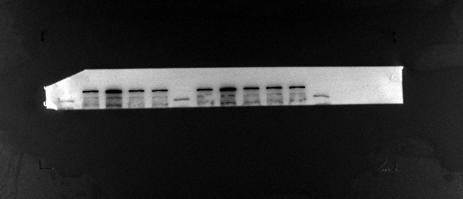

Supplement: Supplementary file 2 [file DataSheet14.ZIP › Original data of Figure 14/Figure 14A-p-mTORC1-original image-3.tif]

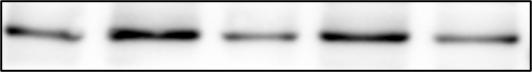

Supplement: Supplementary file 2 [file DataSheet14.ZIP › Original data of Figure 14/Figure 14A-p-raptor-1.tif]

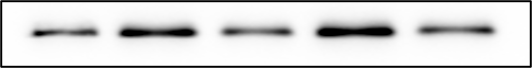

Supplement: Supplementary file 2 [file DataSheet14.ZIP › Original data of Figure 14/Figure 14A-p-raptor-2.tif]

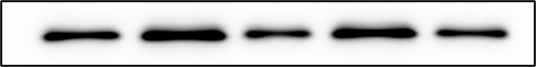

Supplement: Supplementary file 2 [file DataSheet14.ZIP › Original data of Figure 14/Figure 14A-p-raptor-3.tif]

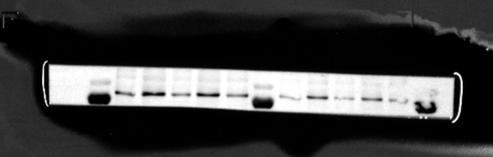

Supplement: Supplementary file 2 [file DataSheet14.ZIP › Original data of Figure 14/Figure 14A-p-raptor-original image-1.tif]

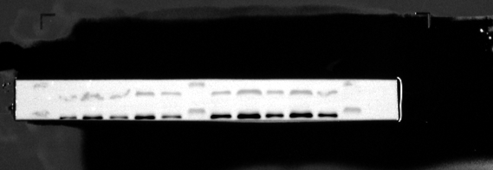

Supplement: Supplementary file 2 [file DataSheet14.ZIP › Original data of Figure 14/Figure 14A-p-raptor-original image-2.tif]

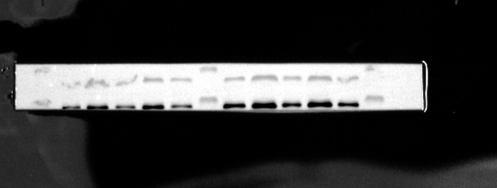

Supplement: Supplementary file 2 [file DataSheet14.ZIP › Original data of Figure 14/Figure 14A-p-raptor-original image-3.tif]

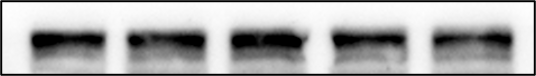

Supplement: Supplementary file 2 [file DataSheet14.ZIP › Original data of Figure 14/Figure 14A-raptor-1.tif]

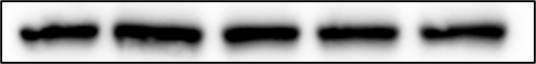

Supplement: Supplementary file 2 [file DataSheet14.ZIP › Original data of Figure 14/Figure 14A-raptor-2.tif]

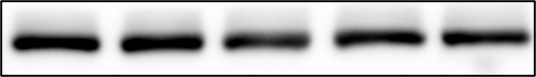

Supplement: Supplementary file 2 [file DataSheet14.ZIP › Original data of Figure 14/Figure 14A-raptor-3.tif]

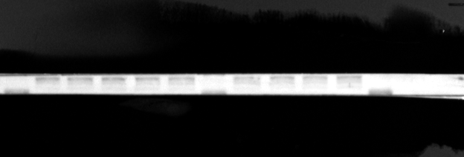

Supplement: Supplementary file 2 [file DataSheet14.ZIP › Original data of Figure 14/Figure 14A-raptor-original image-1.tif]

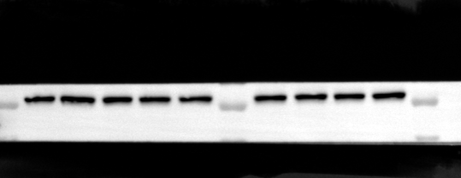

Supplement: Supplementary file 2 [file DataSheet14.ZIP › Original data of Figure 14/Figure 14A-raptor-original image-2.tif]

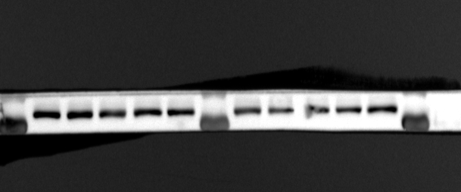

Supplement: Supplementary file 2 [file DataSheet14.ZIP › Original data of Figure 14/Figure 14A-raptor-original image-3.tif]

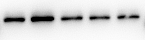

Supplement: Supplementary file 3 [file DataSheet11.ZIP › Original data of Figure 11/Figure 11A-ASC-1.tif]

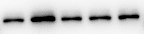

Supplement: Supplementary file 3 [file DataSheet11.ZIP › Original data of Figure 11/Figure 11A-ASC-2.tif]

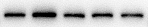

Supplement: Supplementary file 3 [file DataSheet11.ZIP › Original data of Figure 11/Figure 11A-ASC-3.tif]

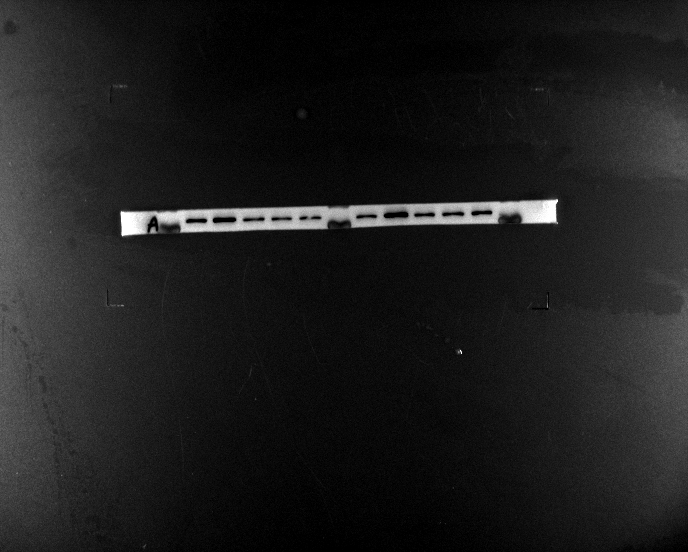

Supplement: Supplementary file 3 [file DataSheet11.ZIP › Original data of Figure 11/Figure 11A-ASC-original image-1.tif]

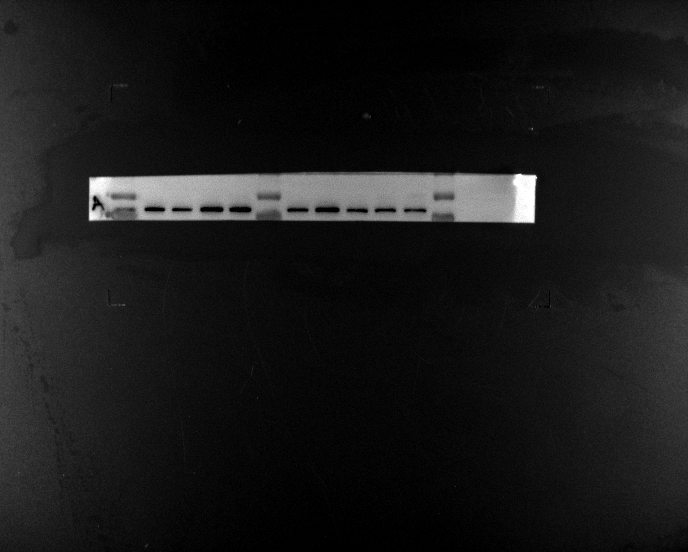

Supplement: Supplementary file 3 [file DataSheet11.ZIP › Original data of Figure 11/Figure 11A-ASC-original image-3.tif]

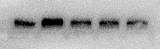

Supplement: Supplementary file 3 [file DataSheet11.ZIP › Original data of Figure 11/Figure 11A-NLRP3-1.tif]

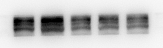

Supplement: Supplementary file 3 [file DataSheet11.ZIP › Original data of Figure 11/Figure 11A-NLRP3-2.tif]

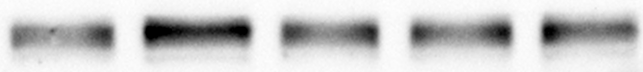

Supplement: Supplementary file 3 [file DataSheet11.ZIP › Original data of Figure 11/Figure 11A-NLRP3-3.tif]

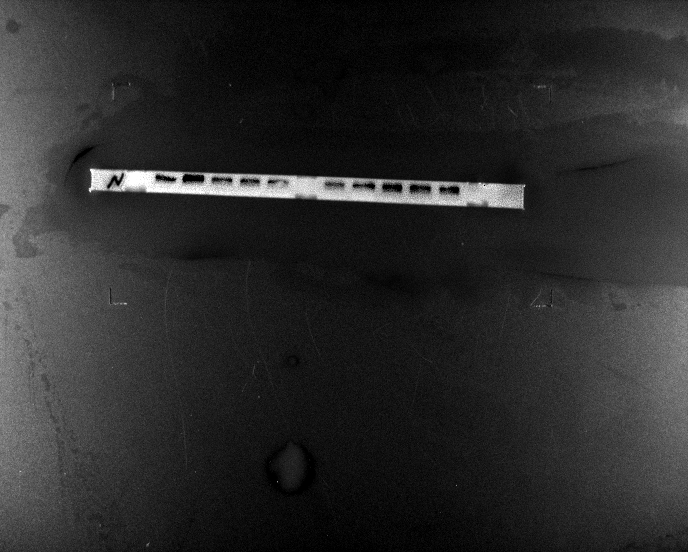

Supplement: Supplementary file 3 [file DataSheet11.ZIP › Original data of Figure 11/Figure 11A-NLRP3-original image-1.tif]

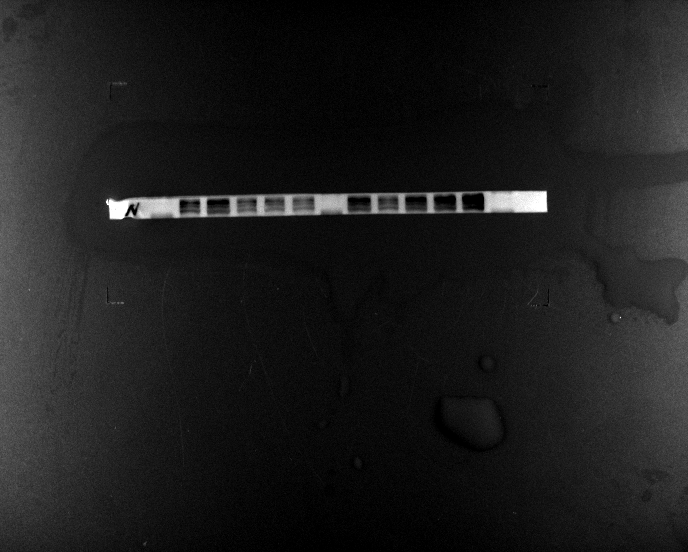

Supplement: Supplementary file 3 [file DataSheet11.ZIP › Original data of Figure 11/Figure 11A-NLRP3-original image-2.tif]

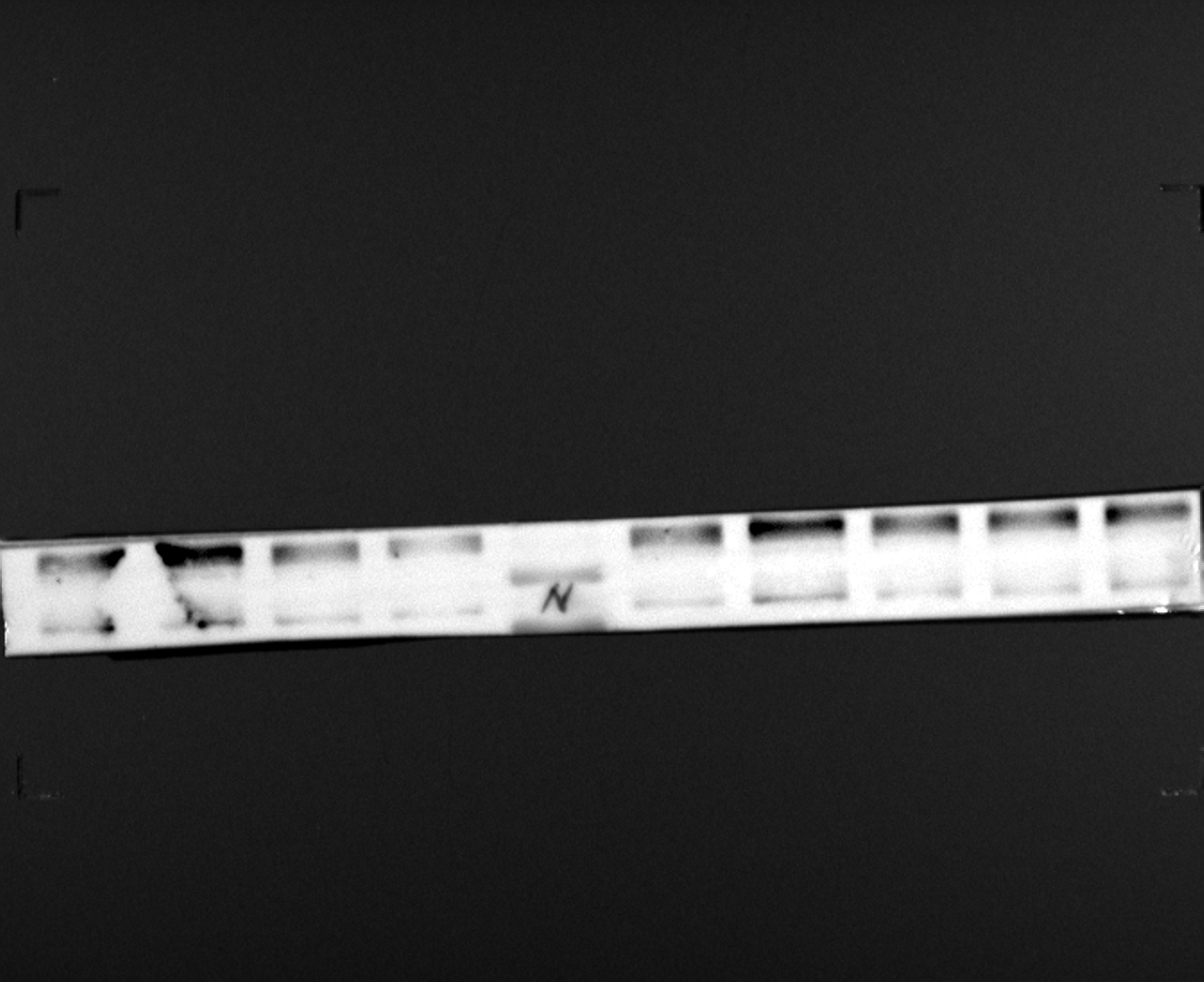

Supplement: Supplementary file 3 [file DataSheet11.ZIP › Original data of Figure 11/Figure 11A-NLRP3-original image-3.Tif]

Caspase-1

Original  
image 1

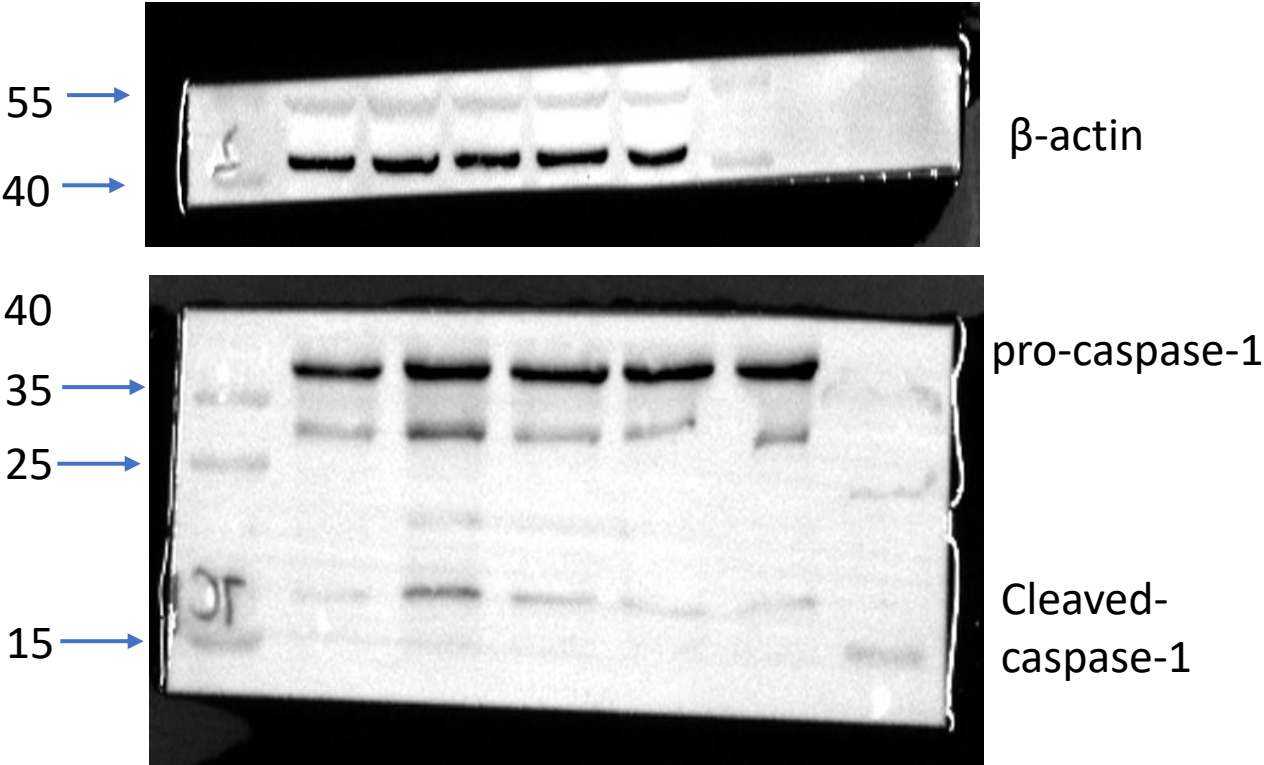

Original  
image 2

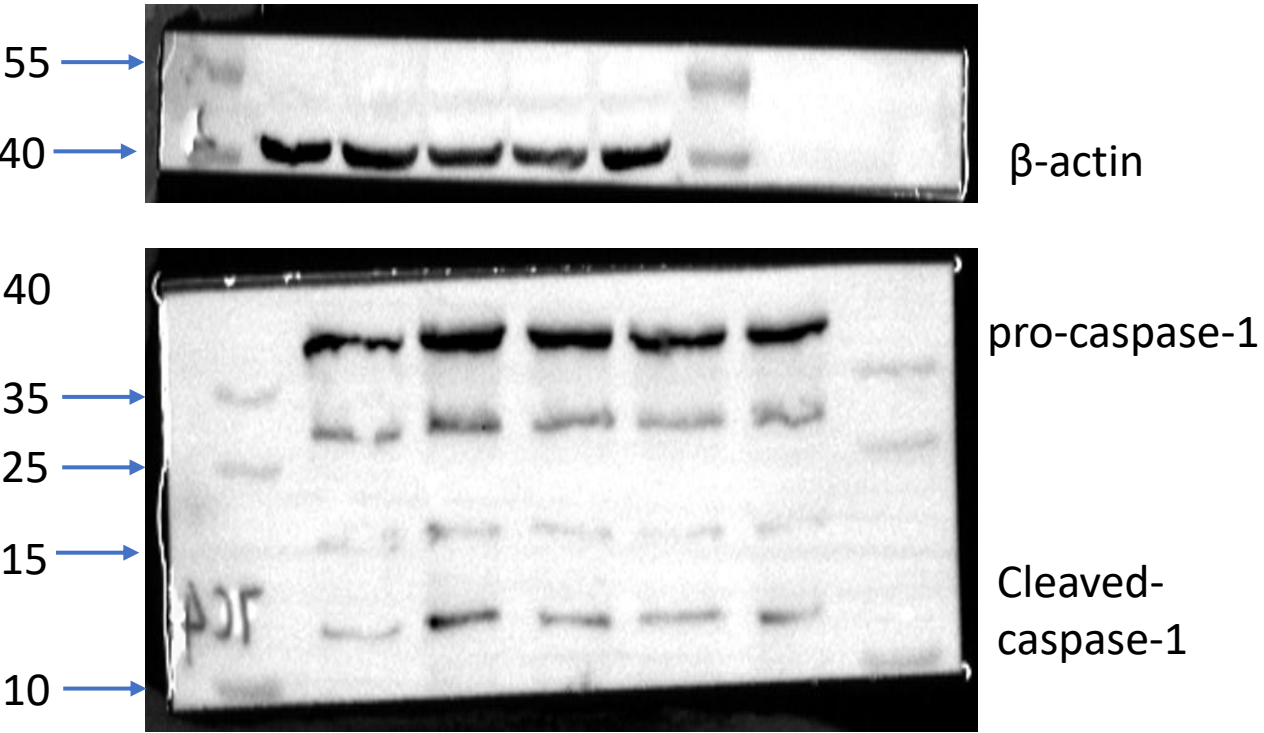

Original  
image 3

55 →  
40 →

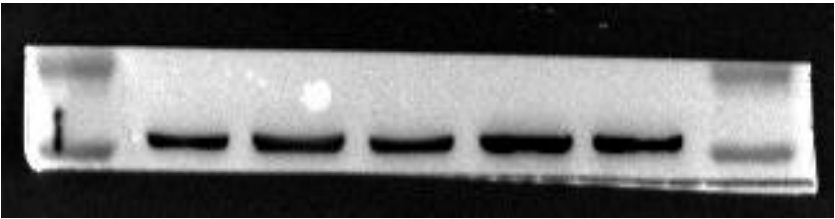

β-actin

40  
35 →  
25 →  
15 →

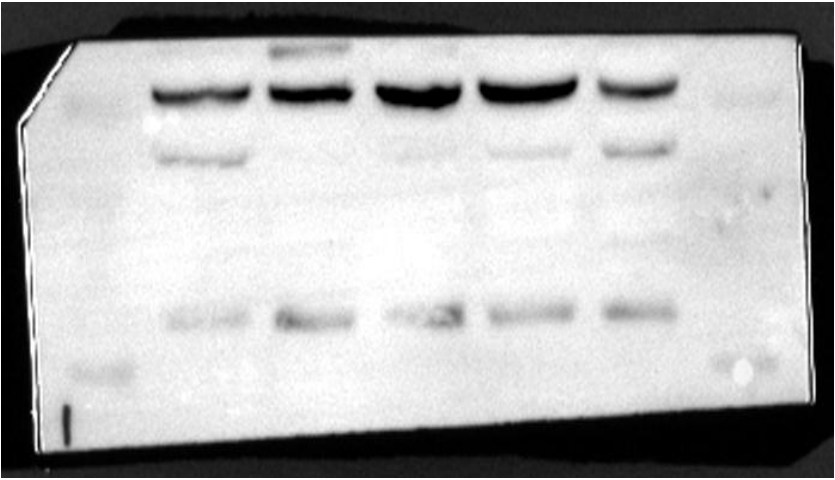

pro-caspase-1

Cleaved-  
caspase-1

Supplement: Supplementary file 3 [file DataSheet11.ZIP › Original data of Figure 11/Figure 11A-pro-Caspase-1, cleaved-Caspase-1 and β-actin-original image-1-3.pdf]

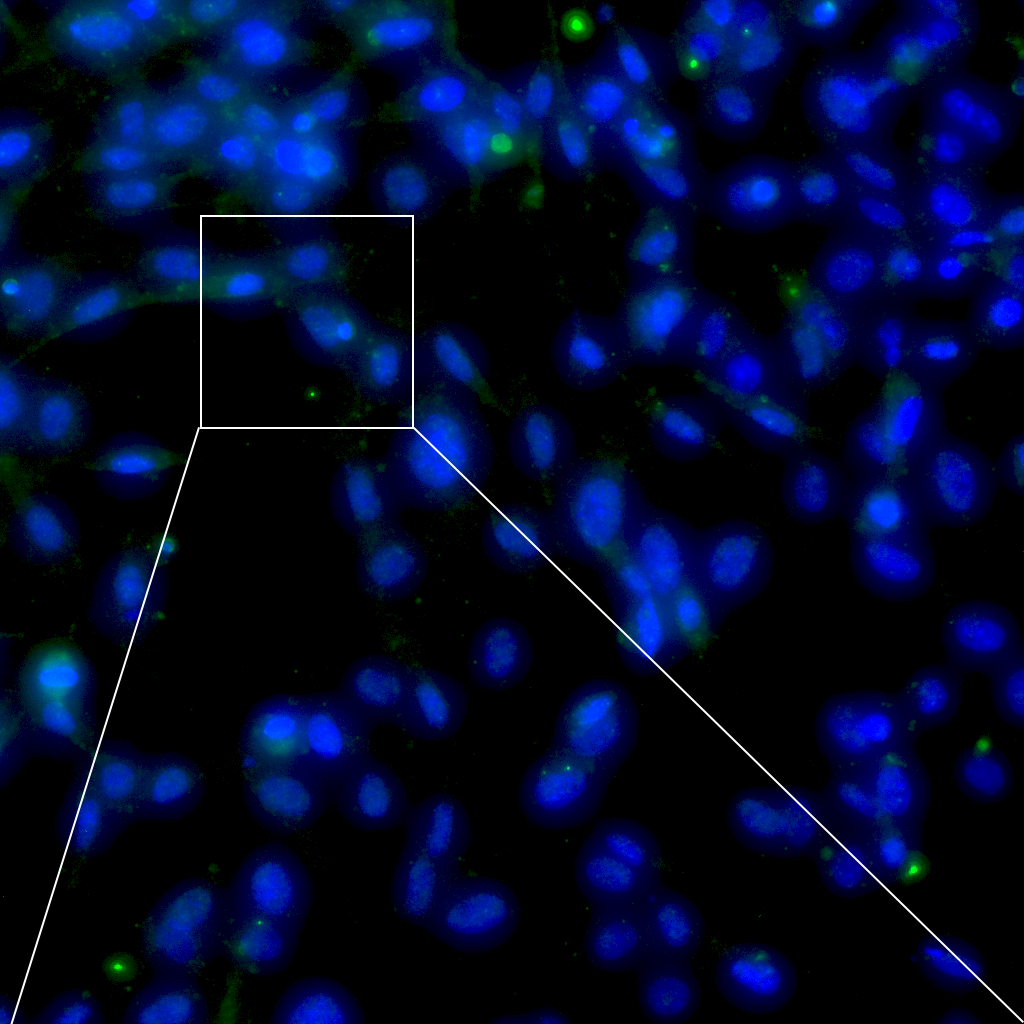

Supplement: Supplementary file 4 [file DataSheet8.ZIP › Original data of Figure 8/Figure 8A-Control-original image.tif]

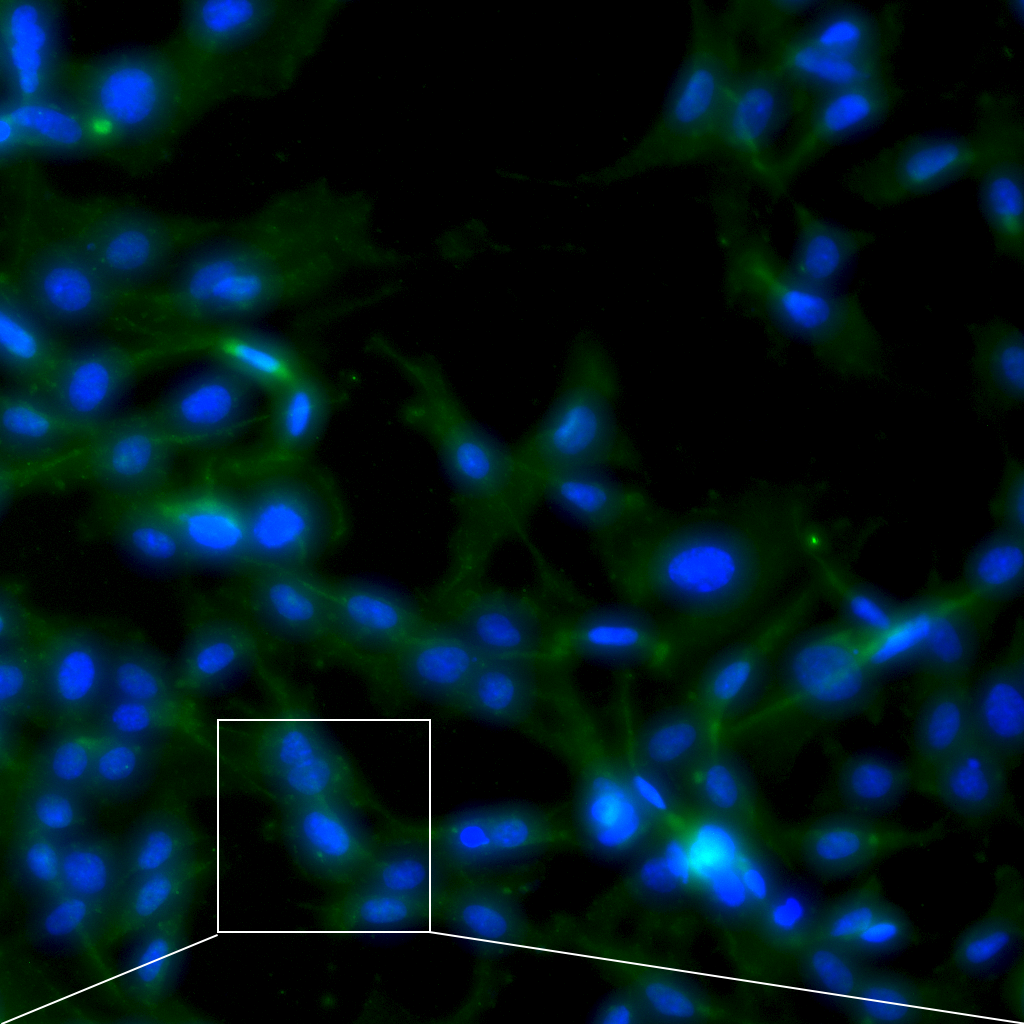

Supplement: Supplementary file 4 [file DataSheet8.ZIP › Original data of Figure 8/Figure 8A-HG+FPS-original image.tif]

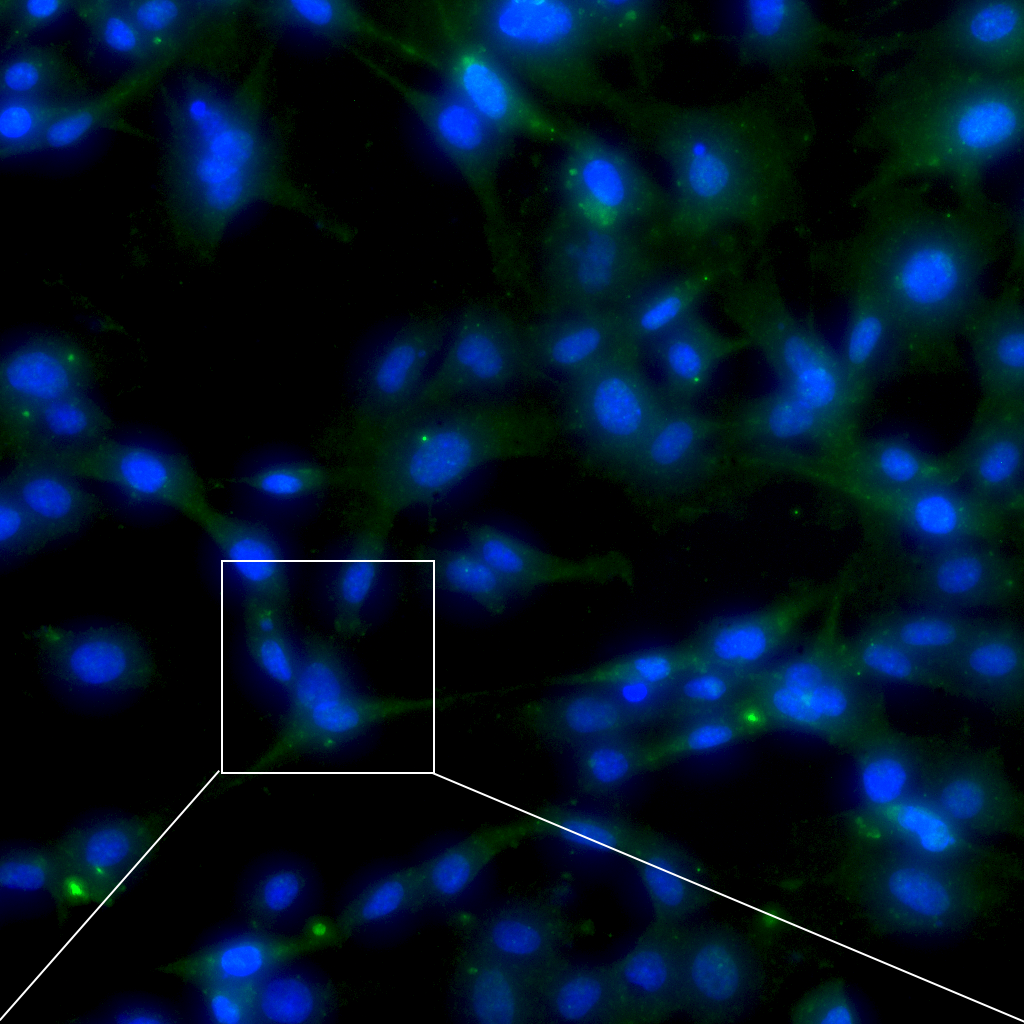

Supplement: Supplementary file 4 [file DataSheet8.ZIP › Original data of Figure 8/Figure 8A-HG+MCC950-original image.tif]

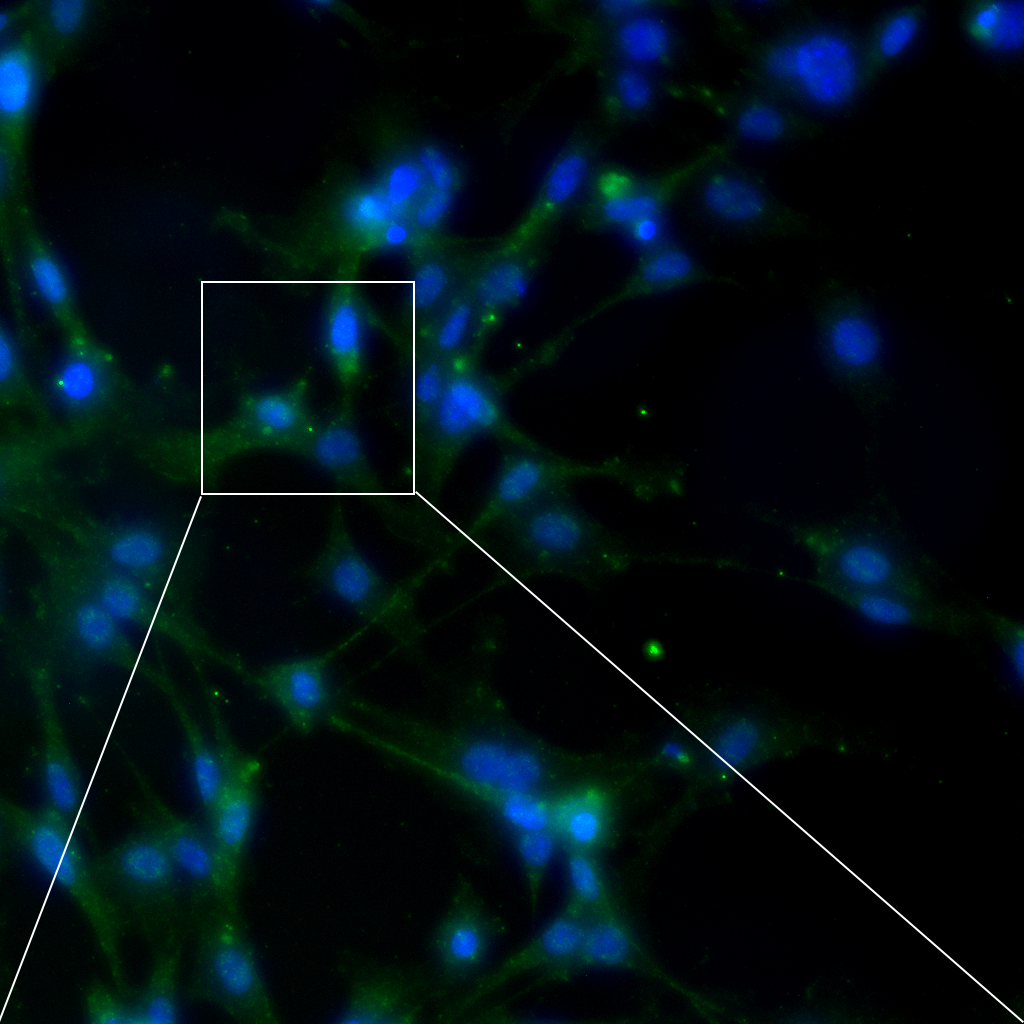

Supplement: Supplementary file 4 [file DataSheet8.ZIP › Original data of Figure 8/Figure 8A-HG+RAP-original image.tif]

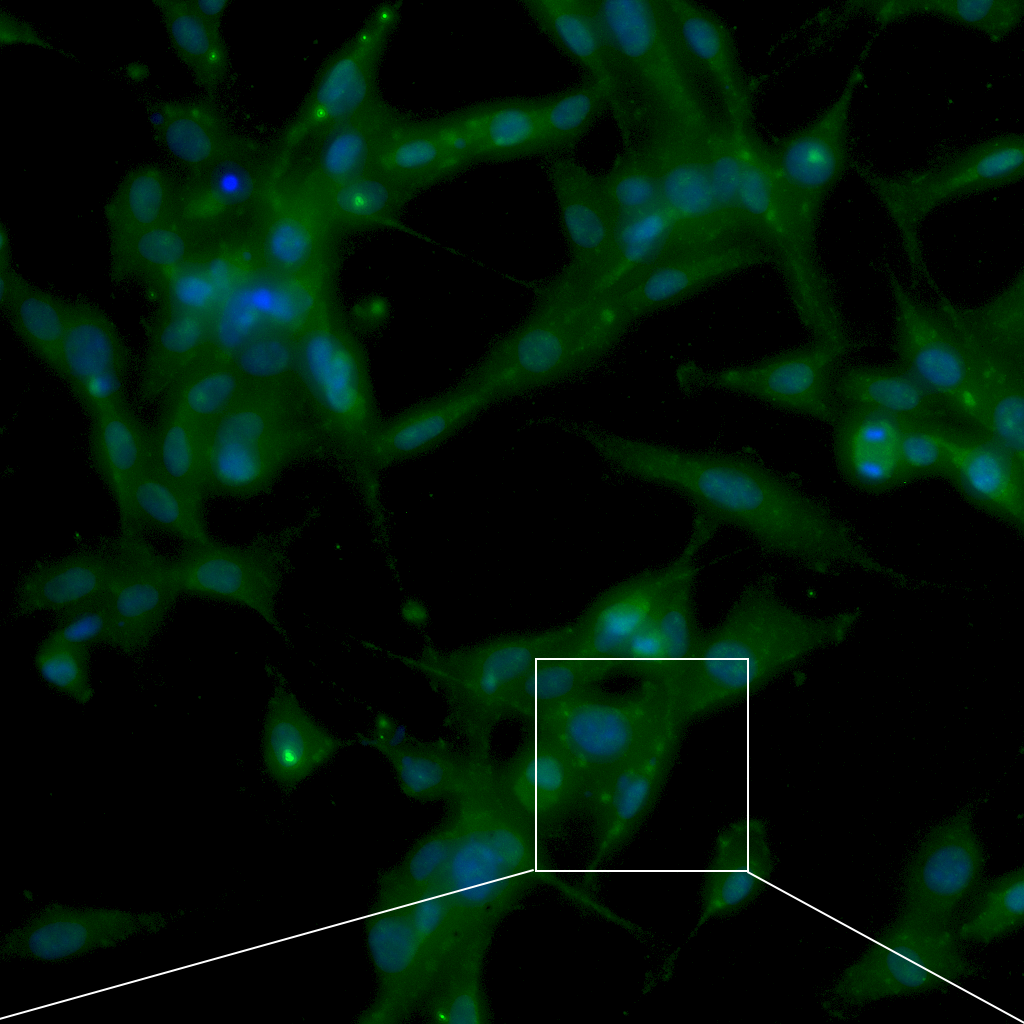

Supplement: Supplementary file 4 [file DataSheet8.ZIP › Original data of Figure 8/Figure 8A-HG-original image.tif]

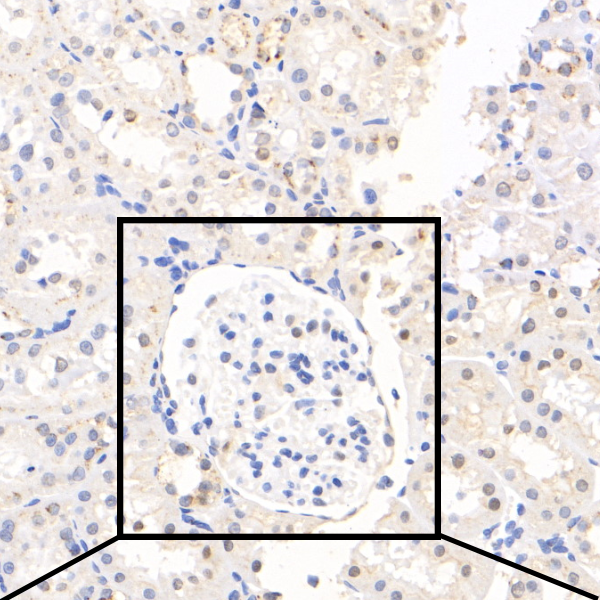

Supplement: Supplementary file 5 [file DataSheet9.ZIP › Original data of Figure 9/Figure 9A-ASC(FPS).tif]

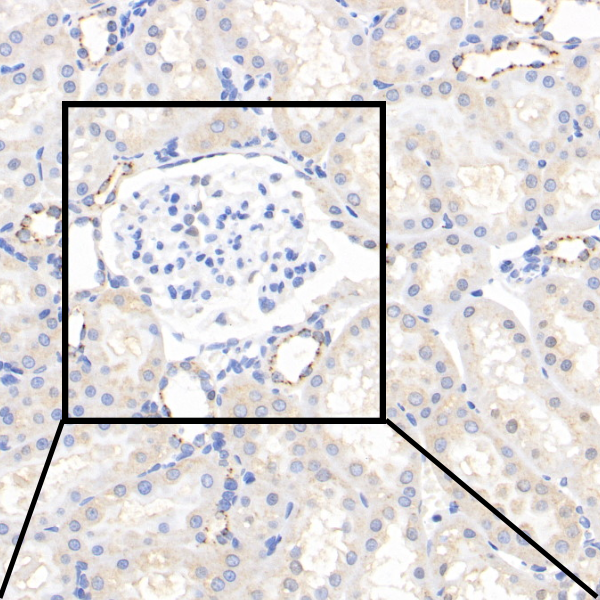

Supplement: Supplementary file 5 [file DataSheet9.ZIP › Original data of Figure 9/Figure 9A-ASC(RAP).tif]

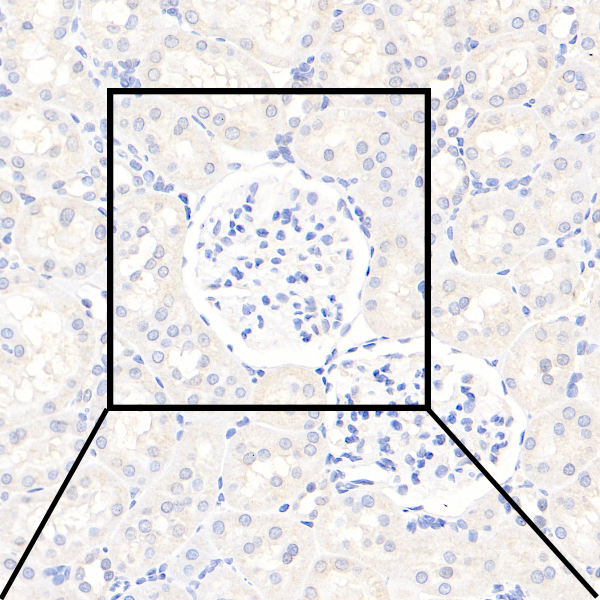

Supplement: Supplementary file 5 [file DataSheet9.ZIP › Original data of Figure 9/Figure 9A-ASC(Sham).tif]

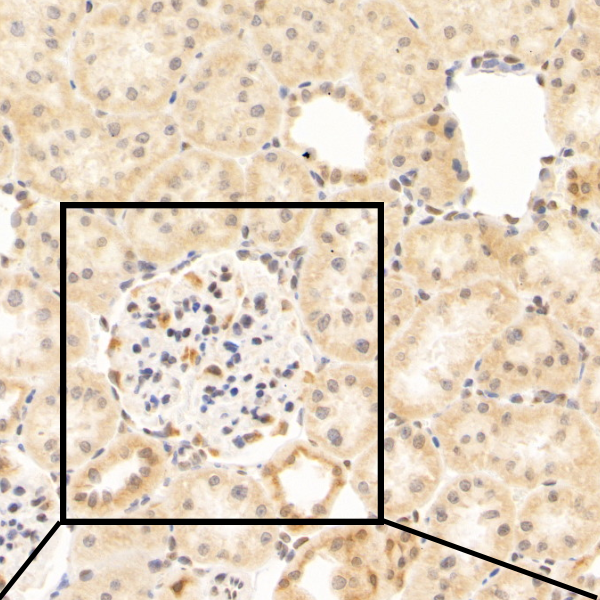

Supplement: Supplementary file 5 [file DataSheet9.ZIP › Original data of Figure 9/Figure 9A-ASC(Vehicle).tif]

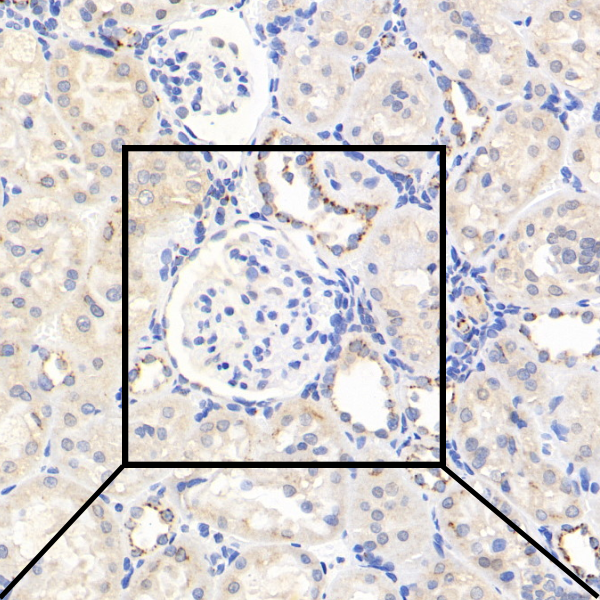

Supplement: Supplementary file 5 [file DataSheet9.ZIP › Original data of Figure 9/Figure 9A-Caspase-1(FPS).tif]

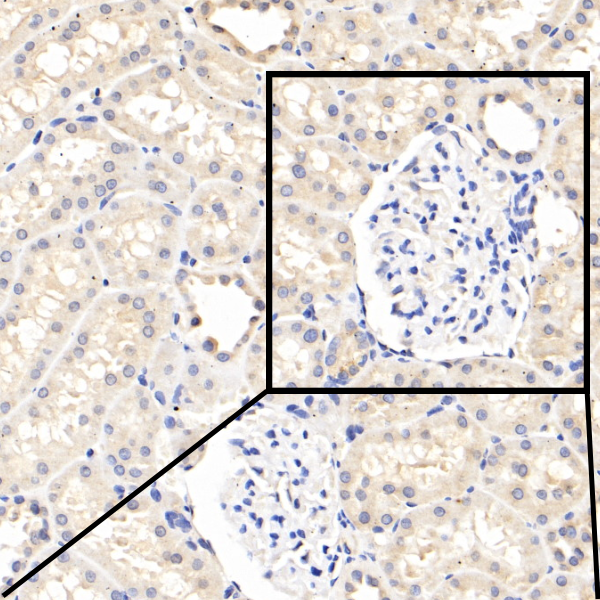

Supplement: Supplementary file 5 [file DataSheet9.ZIP › Original data of Figure 9/Figure 9A-Caspase-1(RAP).tif]

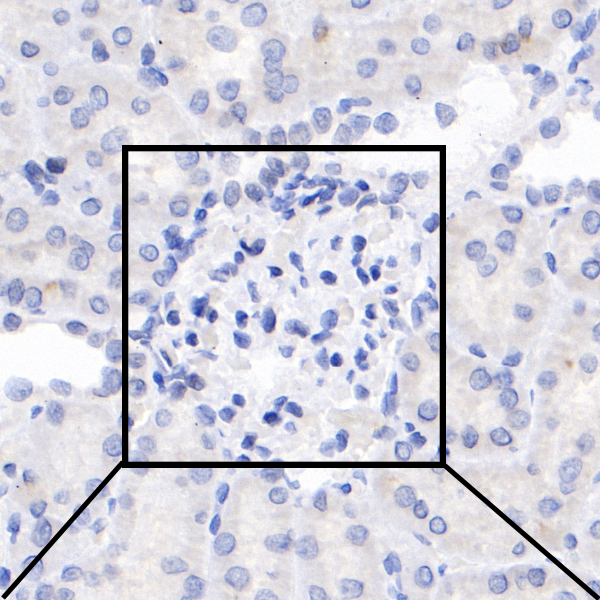

Supplement: Supplementary file 5 [file DataSheet9.ZIP › Original data of Figure 9/Figure 9A-Caspase-1(Sham).tif]

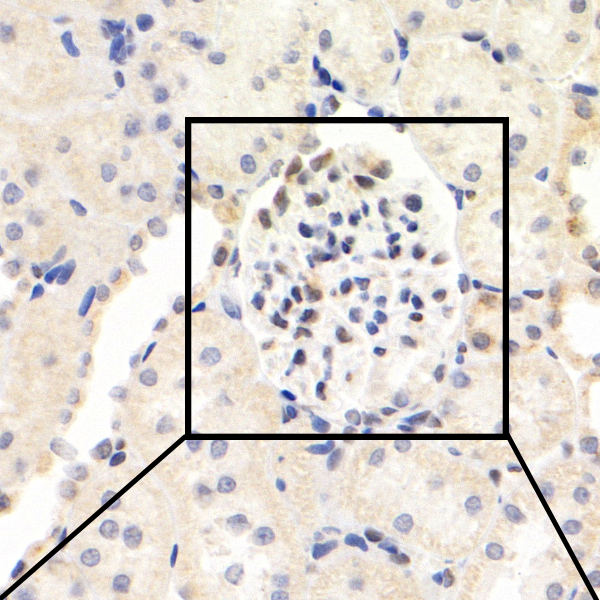

Supplement: Supplementary file 5 [file DataSheet9.ZIP › Original data of Figure 9/Figure 9A-Caspase-1(Vehicle).tif]

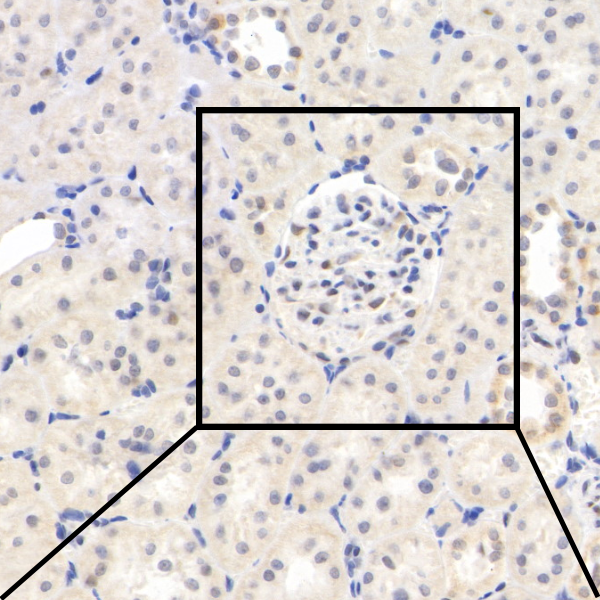

Supplement: Supplementary file 5 [file DataSheet9.ZIP › Original data of Figure 9/Figure 9A-NLRP3(FPS).tif]

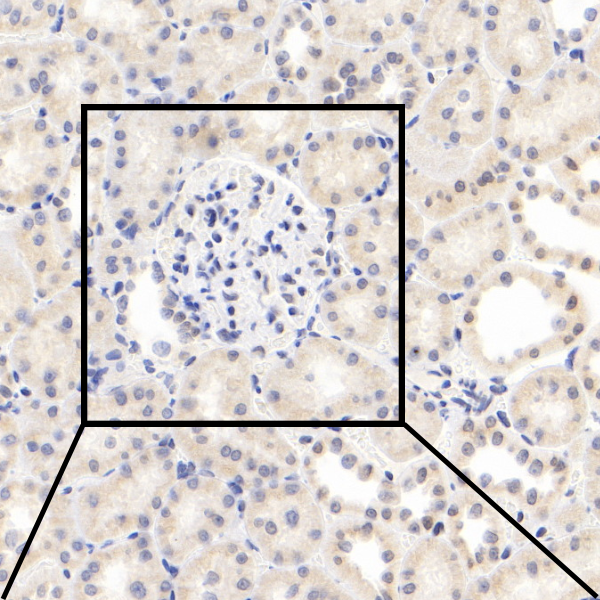

Supplement: Supplementary file 5 [file DataSheet9.ZIP › Original data of Figure 9/Figure 9A-NLRP3(RAP).tif]

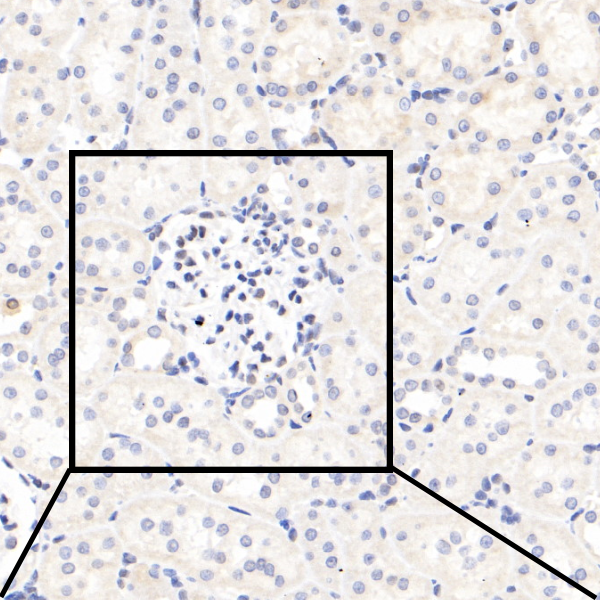

Supplement: Supplementary file 5 [file DataSheet9.ZIP › Original data of Figure 9/Figure 9A-NLRP3(Sham).tif]

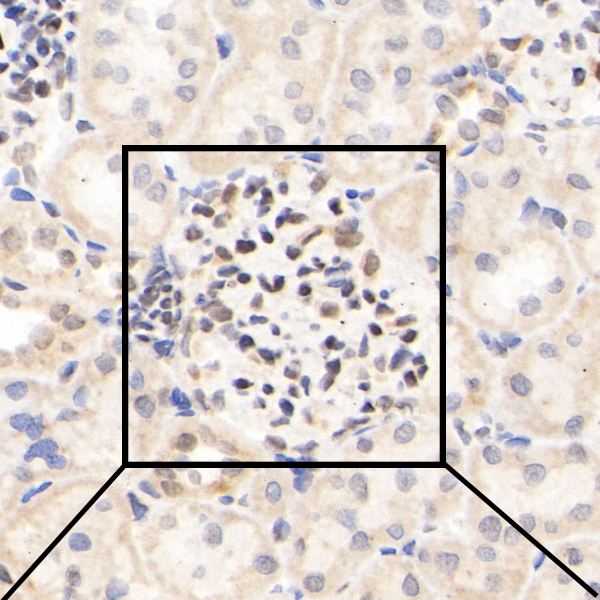

Supplement: Supplementary file 5 [file DataSheet9.ZIP › Original data of Figure 9/Figure 9A-NLRP3(Vehicle).tif]

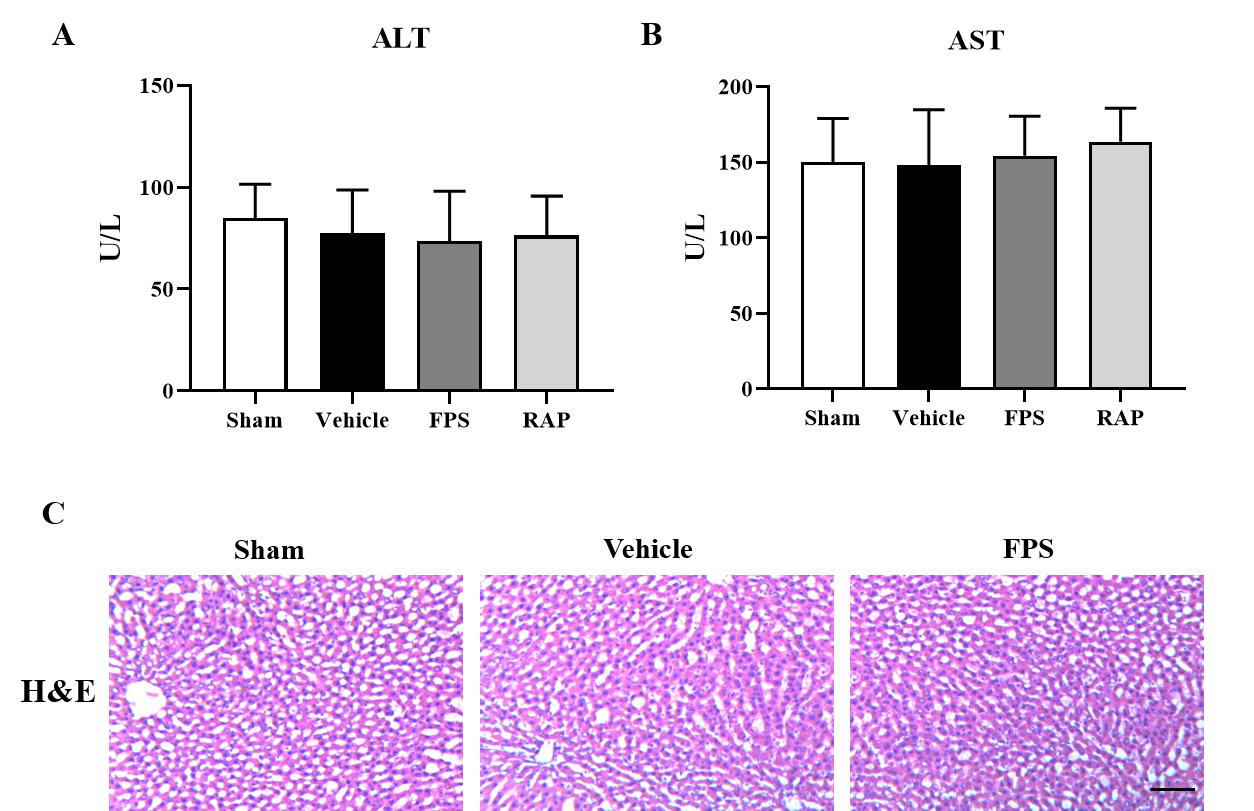

Supplement: Supplementary file 6 [file Image3.TIF]

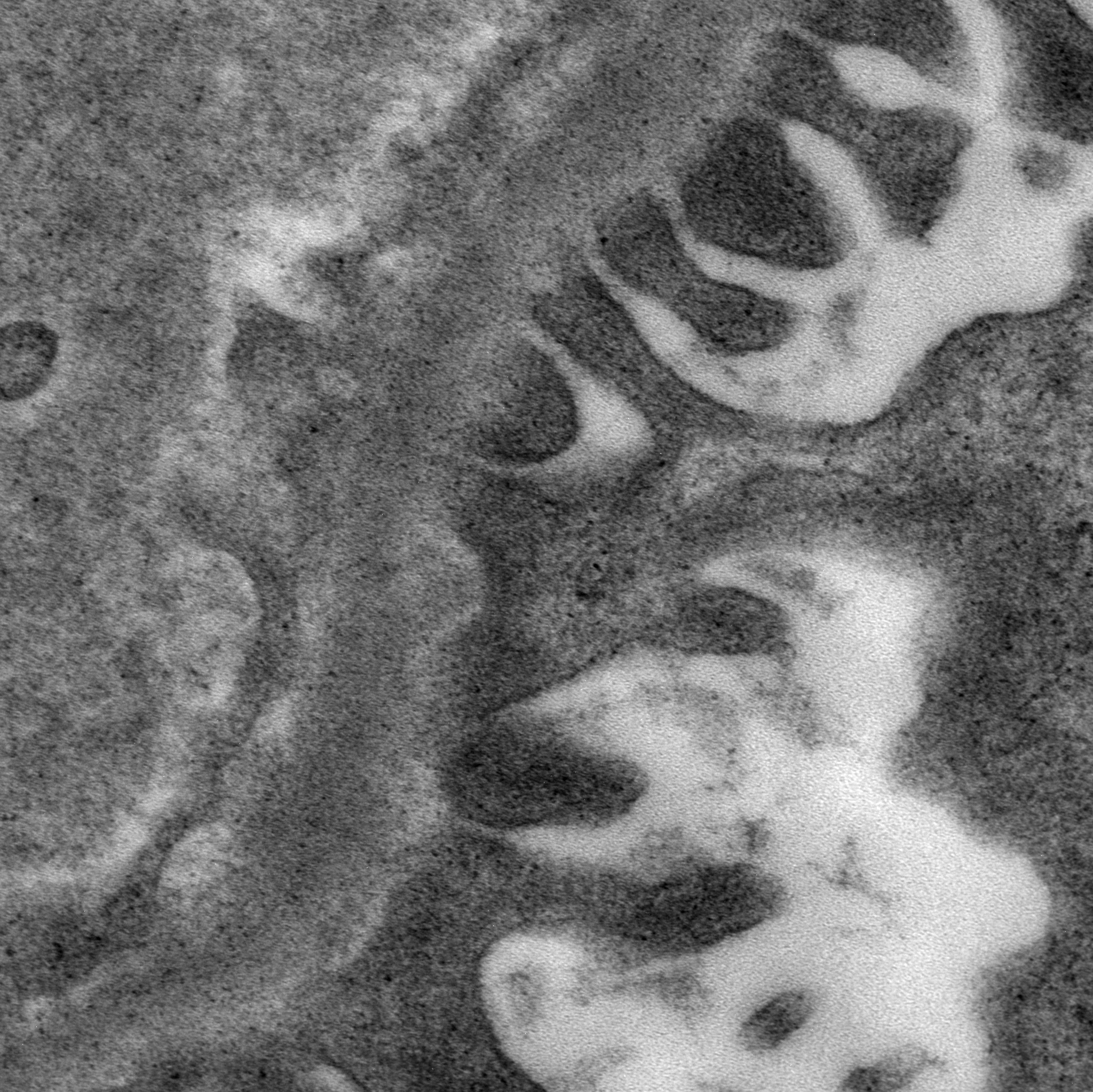

Supplement: Supplementary file 7 [file DataSheet4.ZIP › Original data of Figure 4/Figure 4A (FPS).tif]

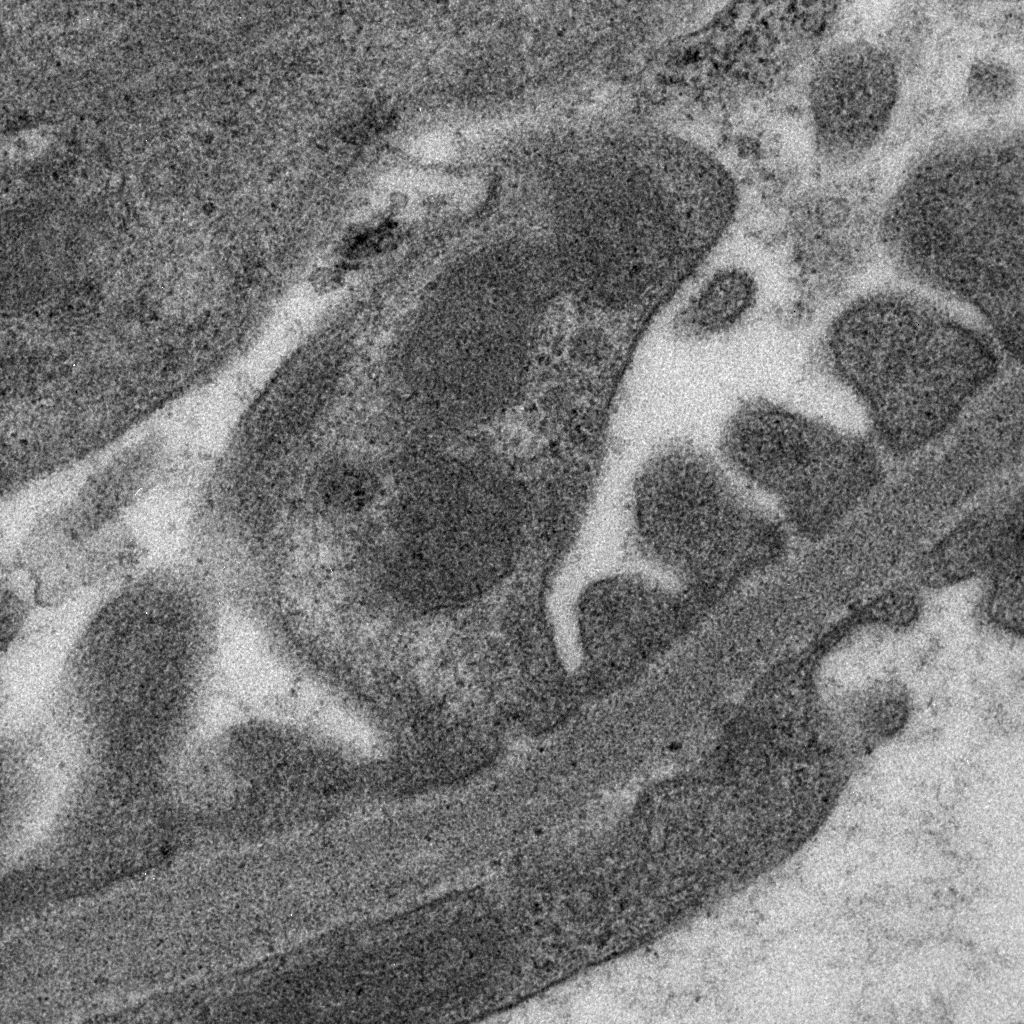

Supplement: Supplementary file 7 [file DataSheet4.ZIP › Original data of Figure 4/Figure 4A (RAP).tif]

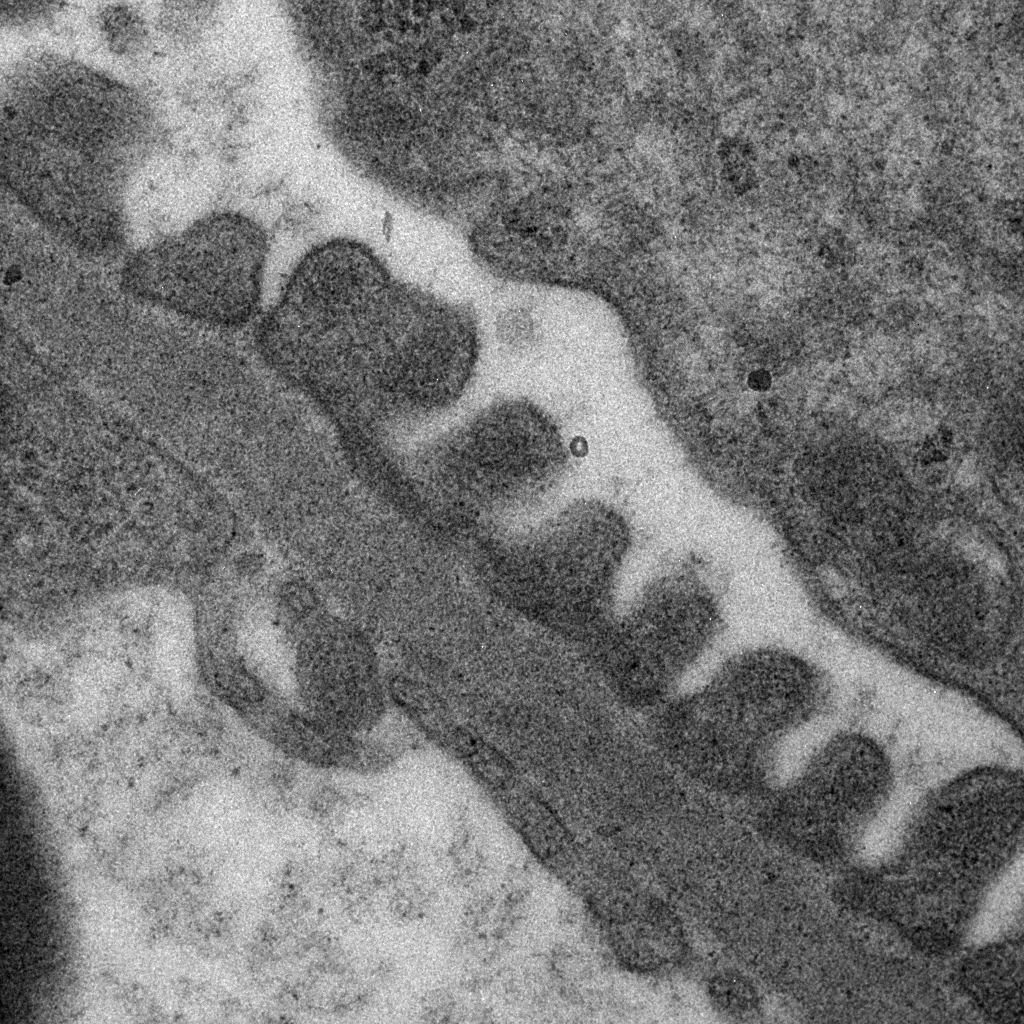

Supplement: Supplementary file 7 [file DataSheet4.ZIP › Original data of Figure 4/Figure 4A (Sham).tif]

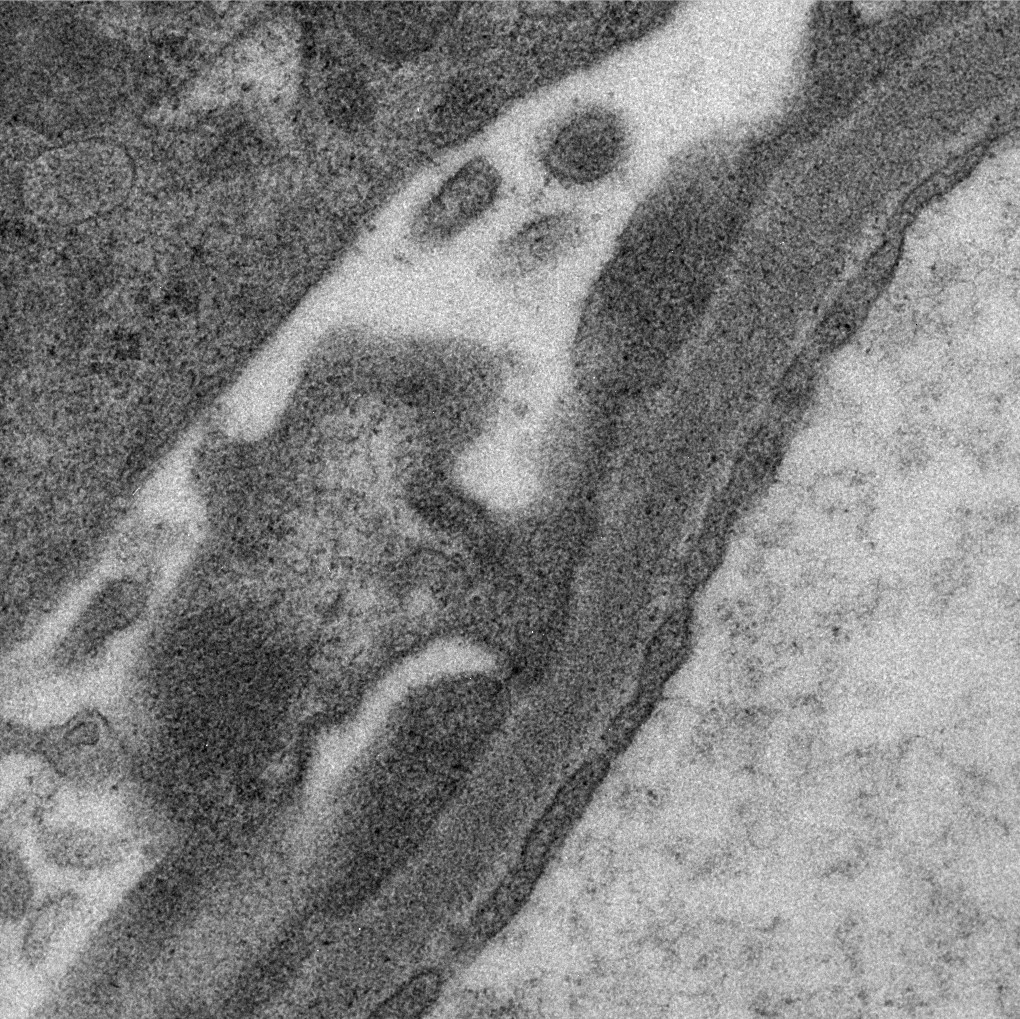

Supplement: Supplementary file 7 [file DataSheet4.ZIP › Original data of Figure 4/Figure 4A (Vehicle).jpg]

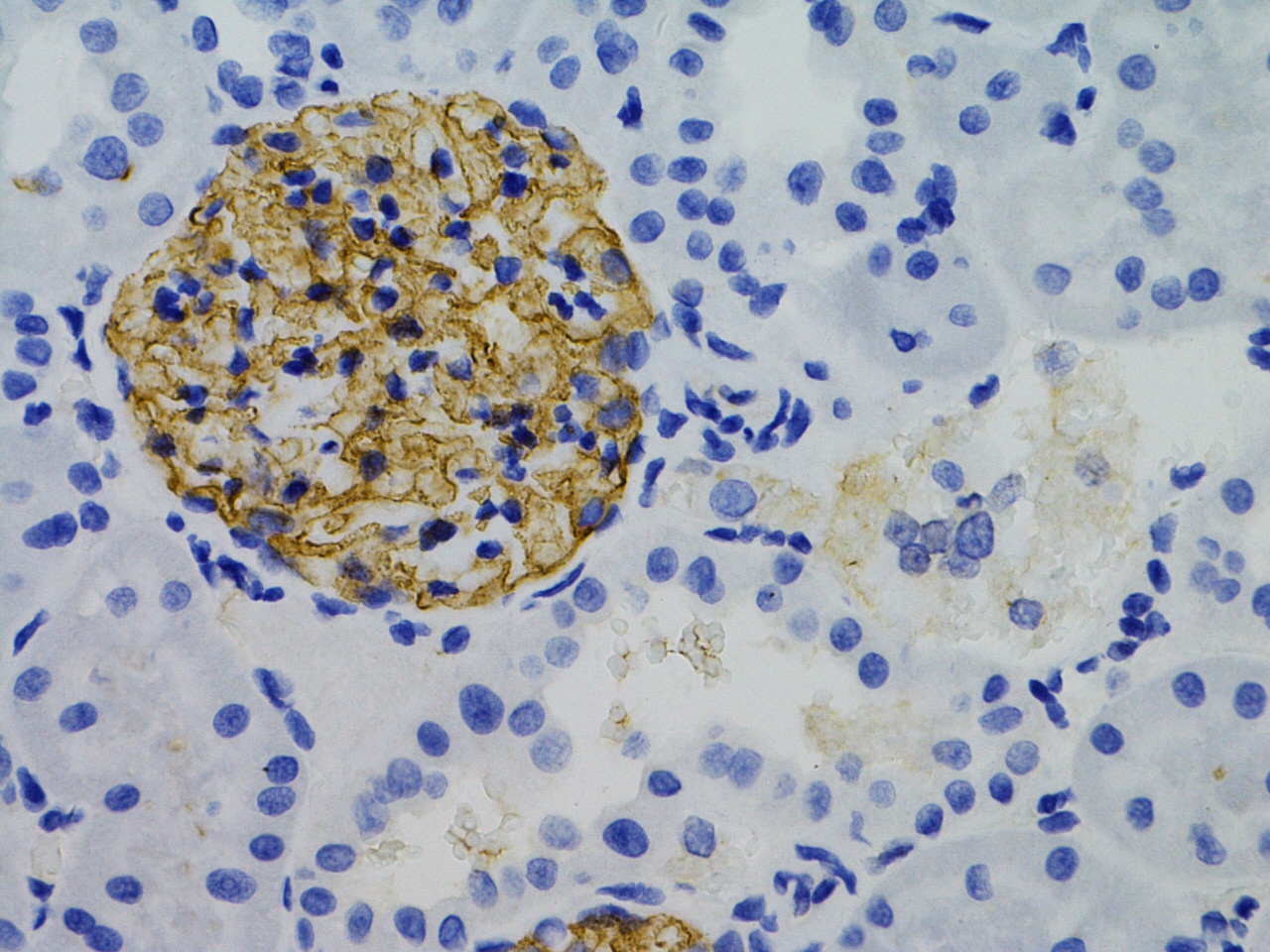

Supplement: Supplementary file 7 [file DataSheet4.ZIP › Original data of Figure 4/Figure 4C-CD2AP (FPS).jpg]

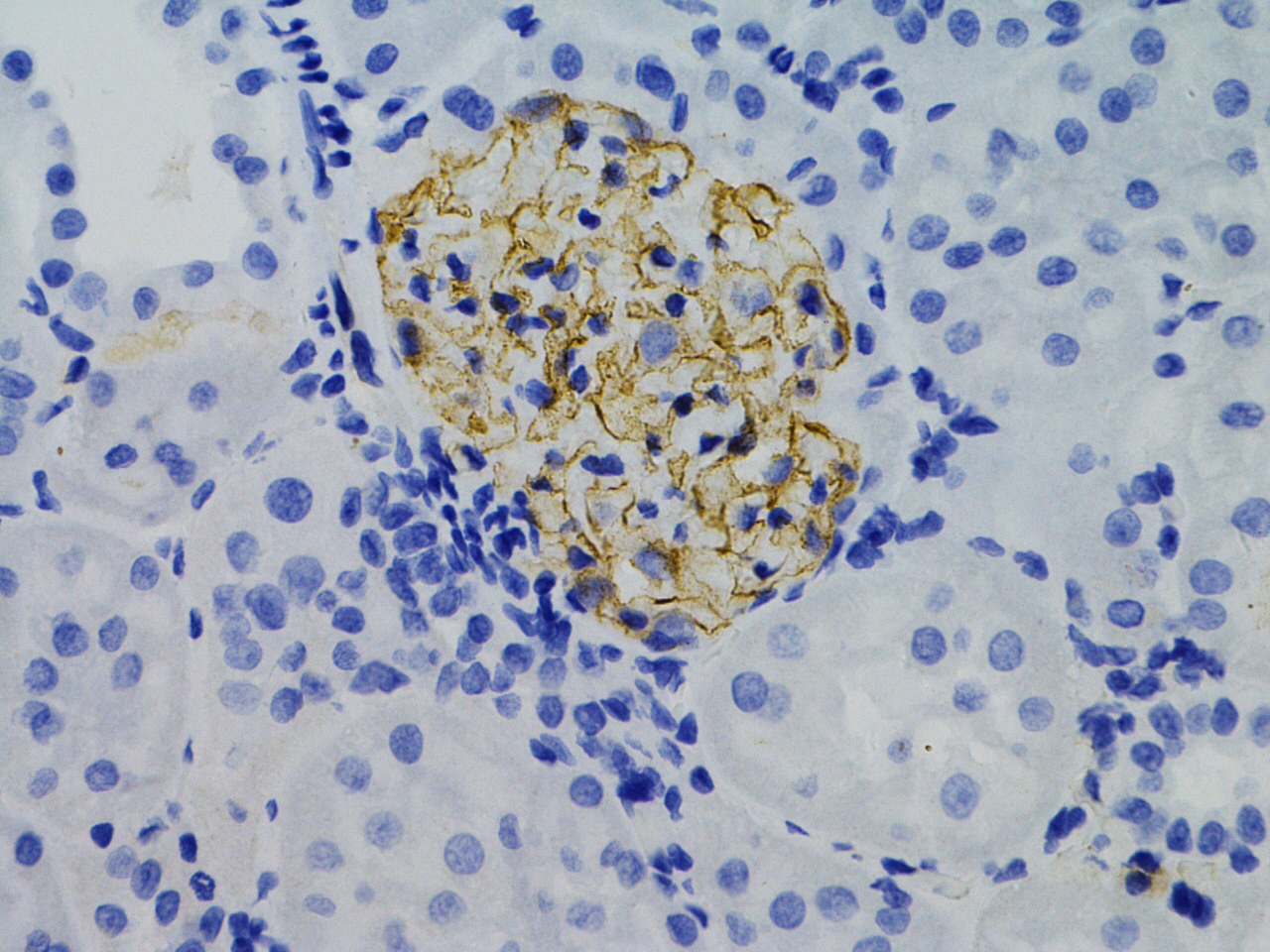

Supplement: Supplementary file 7 [file DataSheet4.ZIP › Original data of Figure 4/Figure 4C-CD2AP (RAP).jpg]

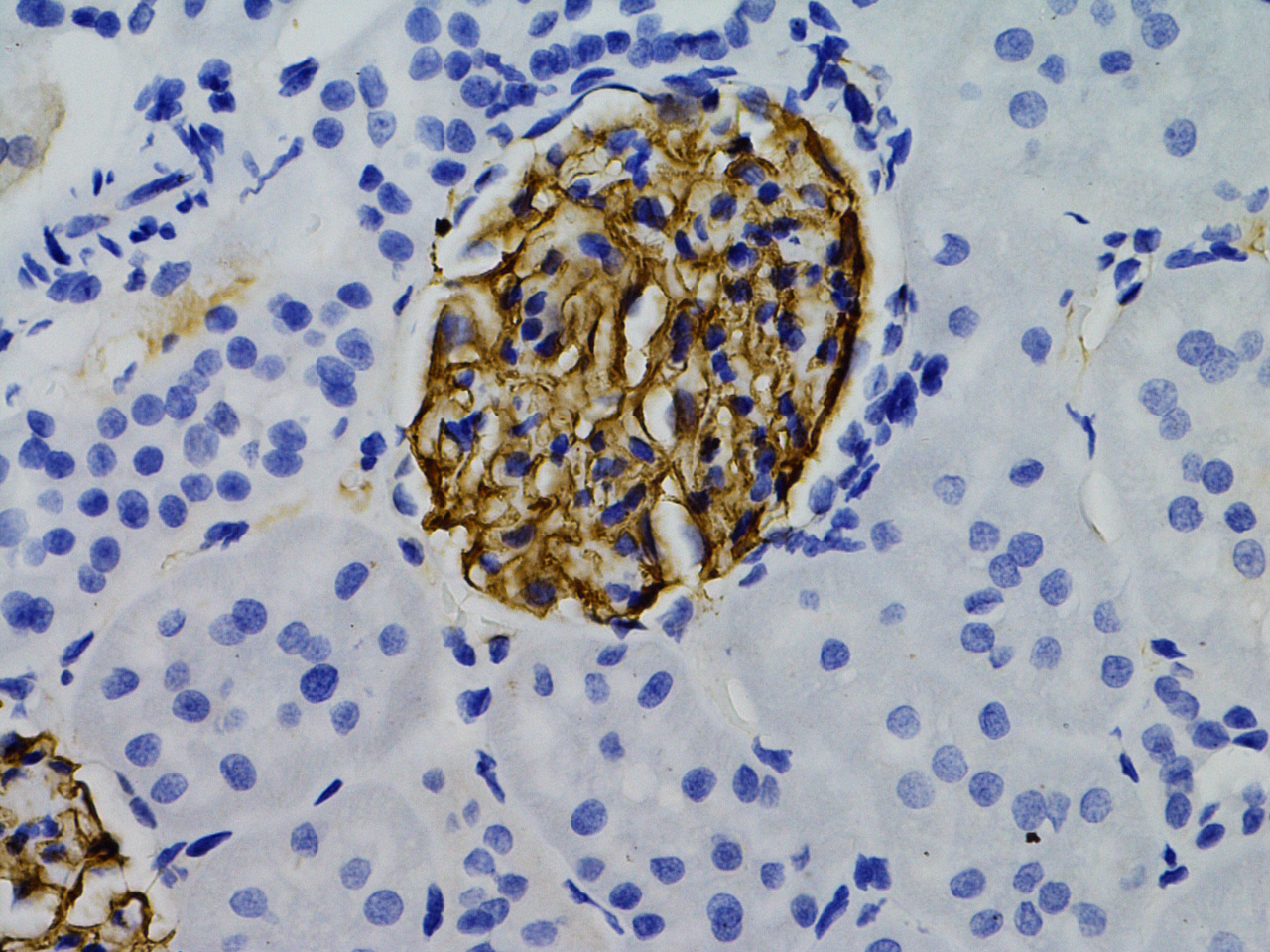

Supplement: Supplementary file 7 [file DataSheet4.ZIP › Original data of Figure 4/Figure 4C-CD2AP (Sham).jpg]

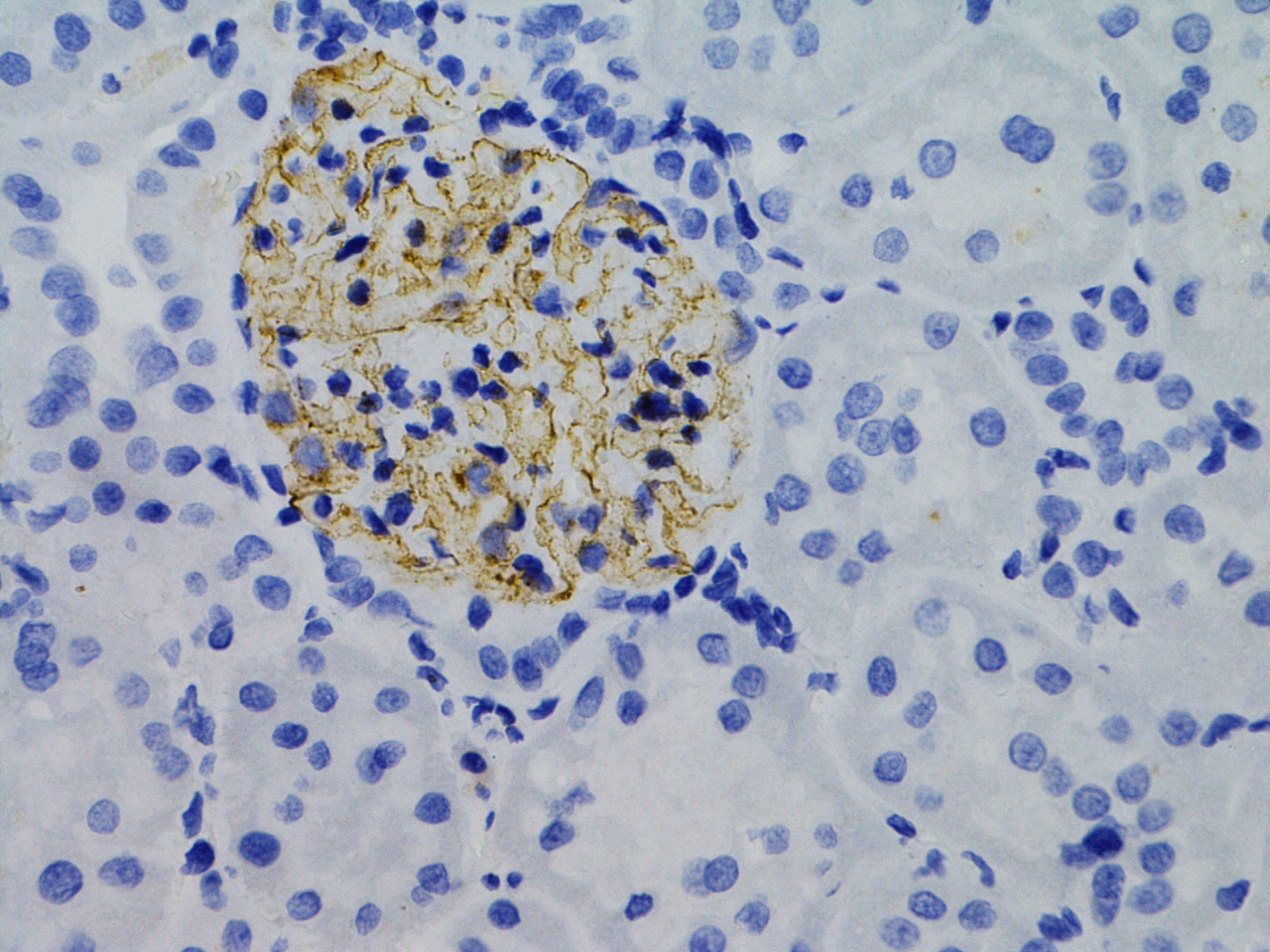

Supplement: Supplementary file 7 [file DataSheet4.ZIP › Original data of Figure 4/Figure 4C-CD2AP (Vehicle).jpg]

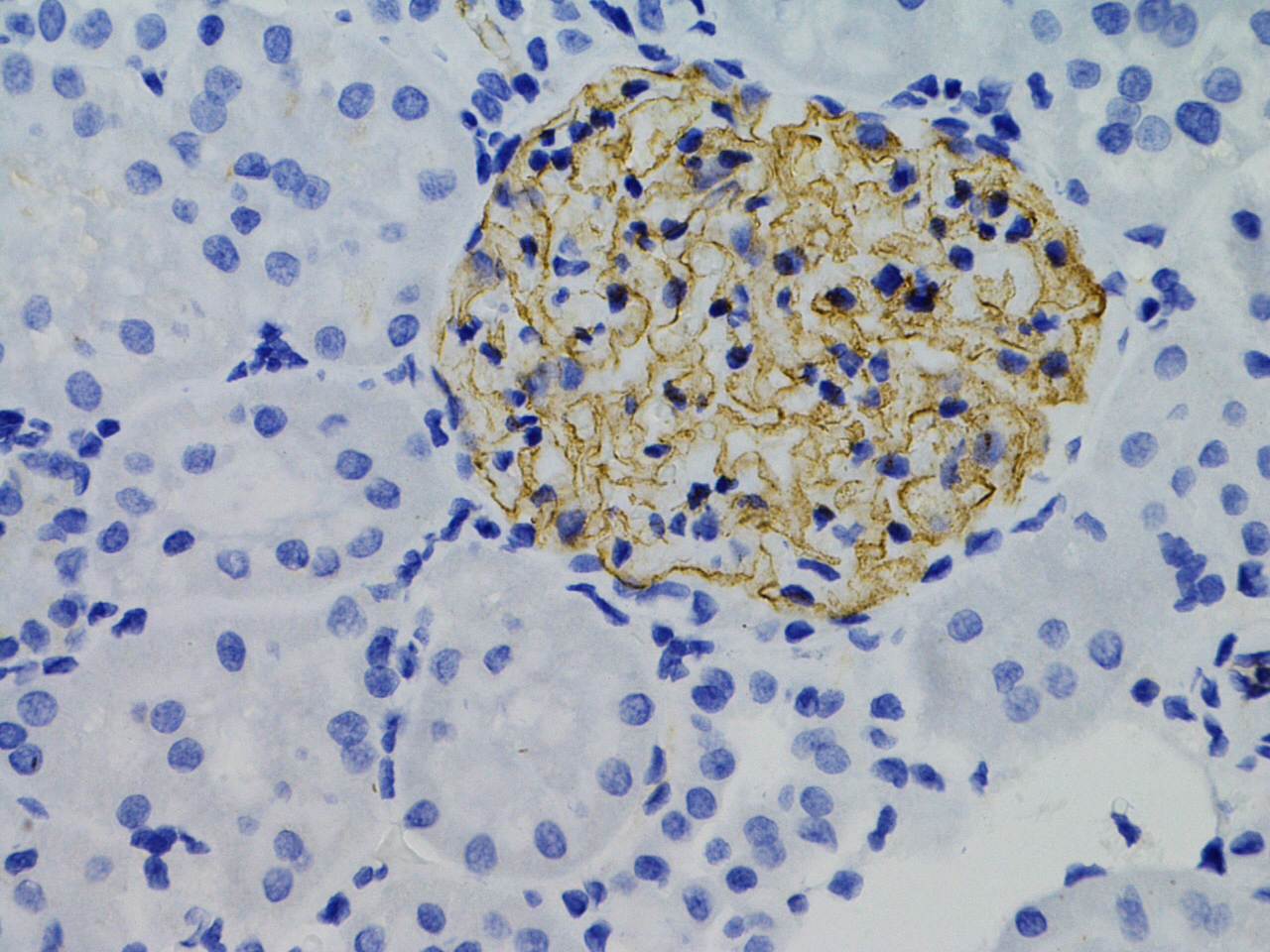

Supplement: Supplementary file 7 [file DataSheet4.ZIP › Original data of Figure 4/Figure 4C-podocin (FPS).jpg]

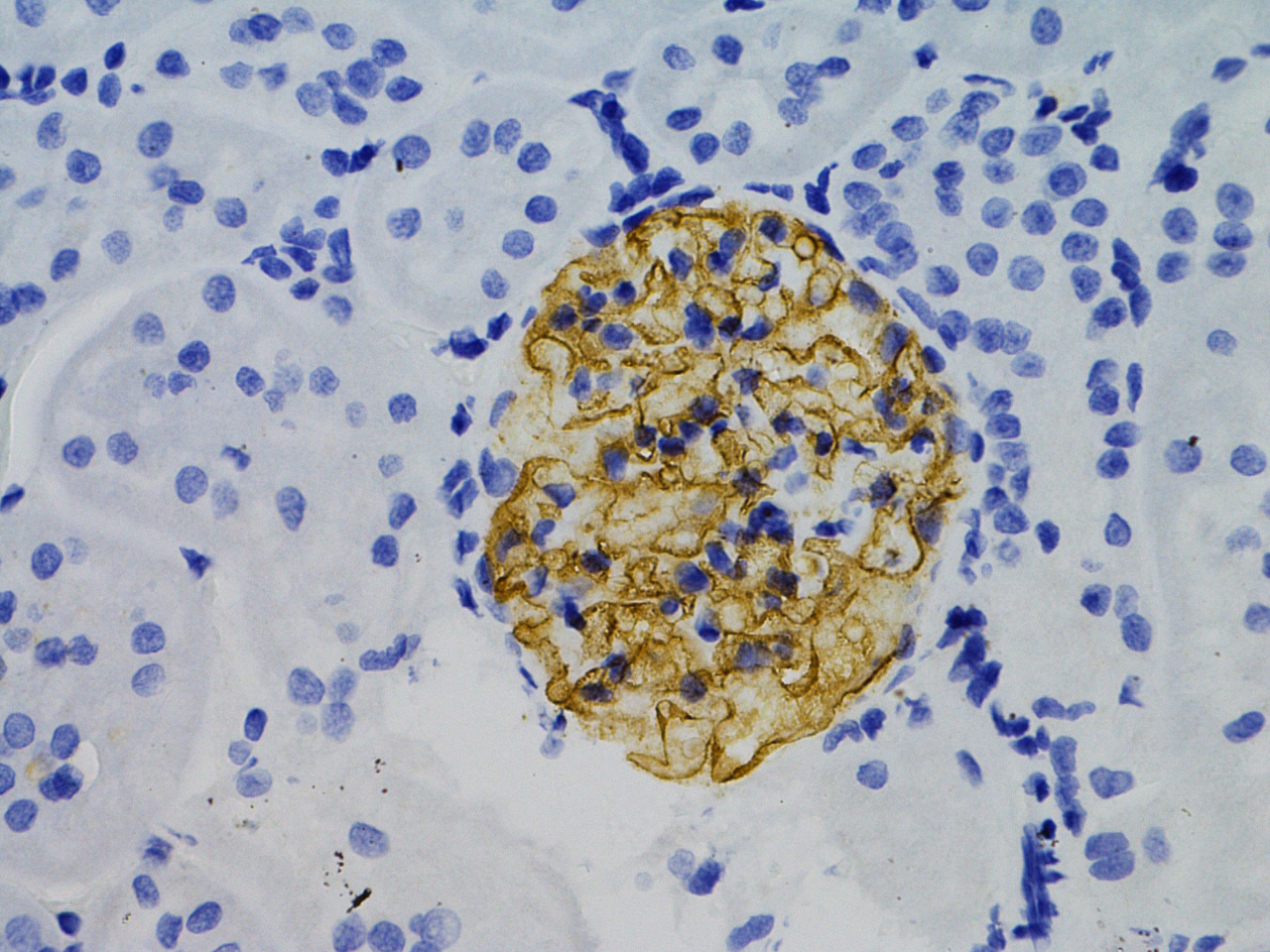

Supplement: Supplementary file 7 [file DataSheet4.ZIP › Original data of Figure 4/Figure 4C-podocin (RAP).jpg]

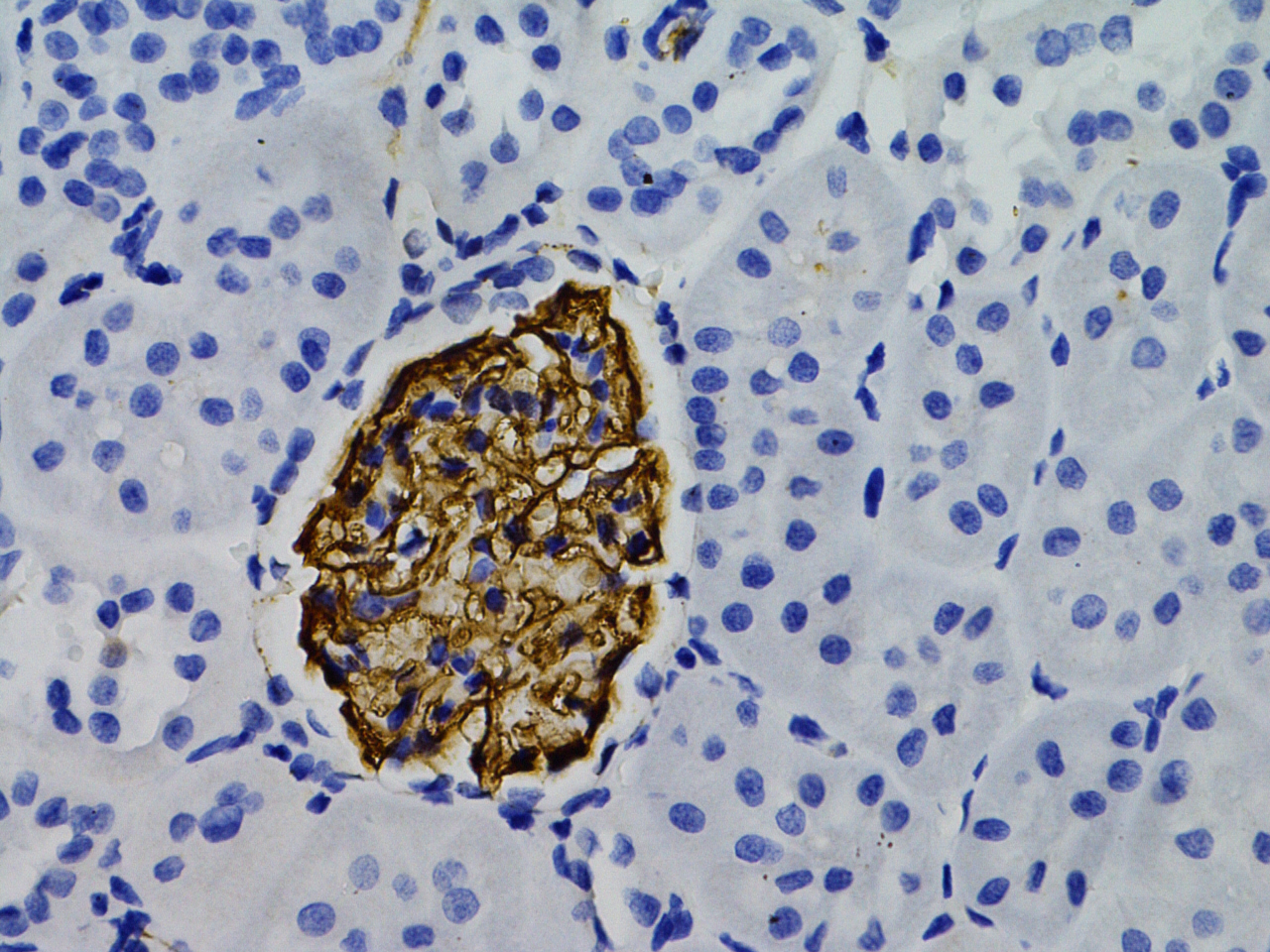

Supplement: Supplementary file 7 [file DataSheet4.ZIP › Original data of Figure 4/Figure 4C-podocin (Sham).jpg]

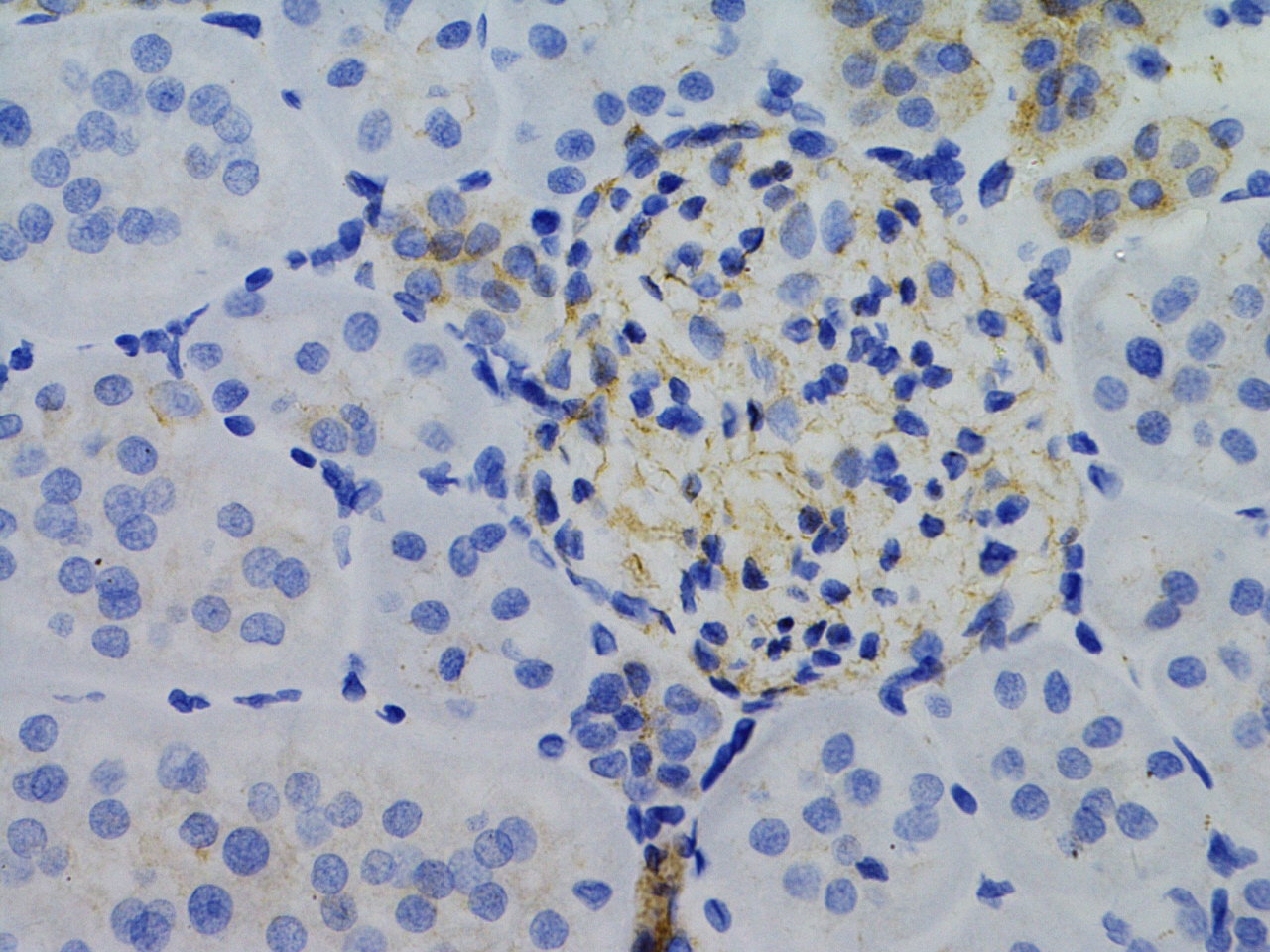

Supplement: Supplementary file 7 [file DataSheet4.ZIP › Original data of Figure 4/Figure 4C-podocin (Vehicle).jpg]

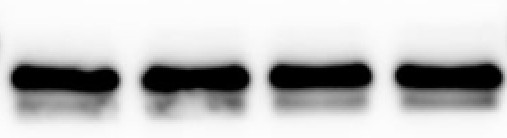

Supplement: Supplementary file 8 [file DataSheet13.ZIP › Original data of Figure 13/Figure 13A-GAPDH-1.jpg]

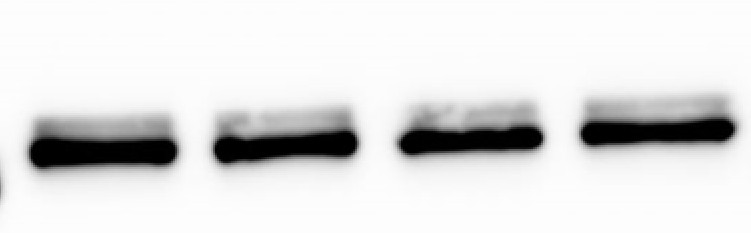

Supplement: Supplementary file 8 [file DataSheet13.ZIP › Original data of Figure 13/Figure 13A-GAPDH-2.jpg]

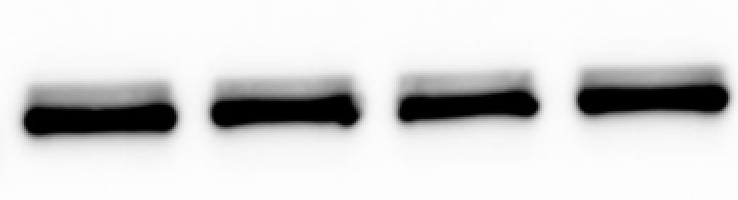

Supplement: Supplementary file 8 [file DataSheet13.ZIP › Original data of Figure 13/Figure 13A-GAPDH-3.jpg]

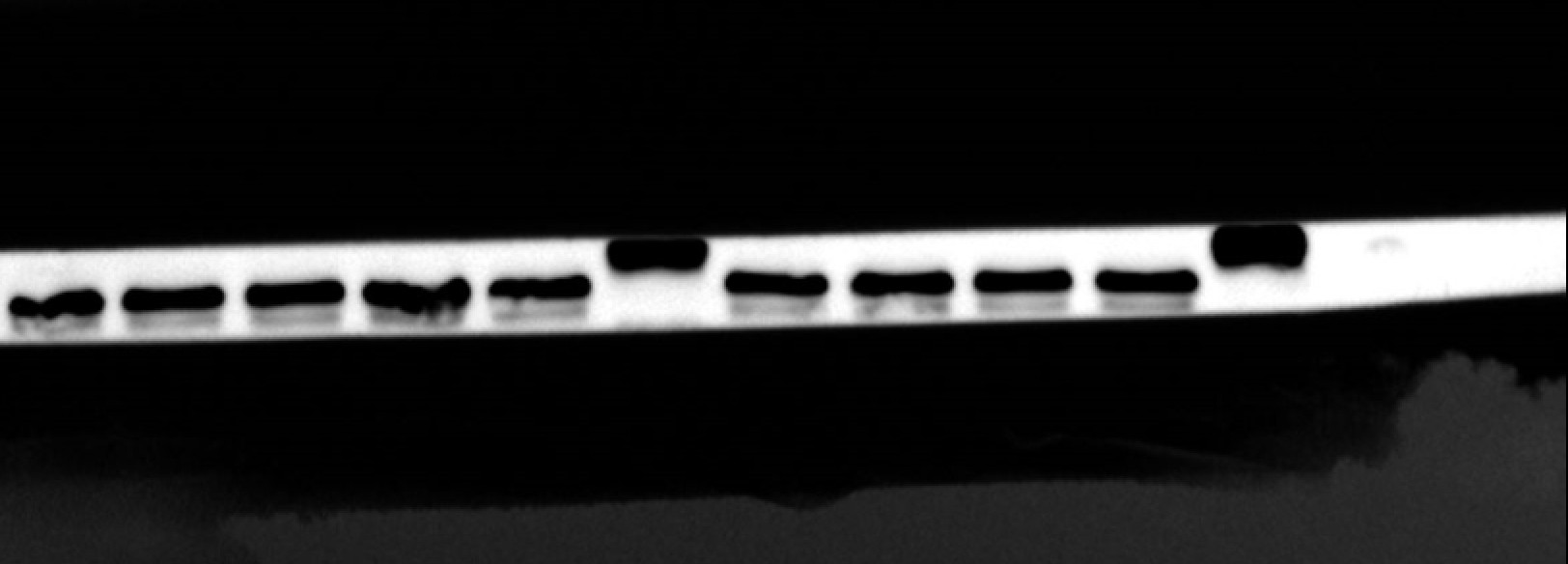

Supplement: Supplementary file 8 [file DataSheet13.ZIP › Original data of Figure 13/Figure 13A-GAPDH-original image-1.jpg]

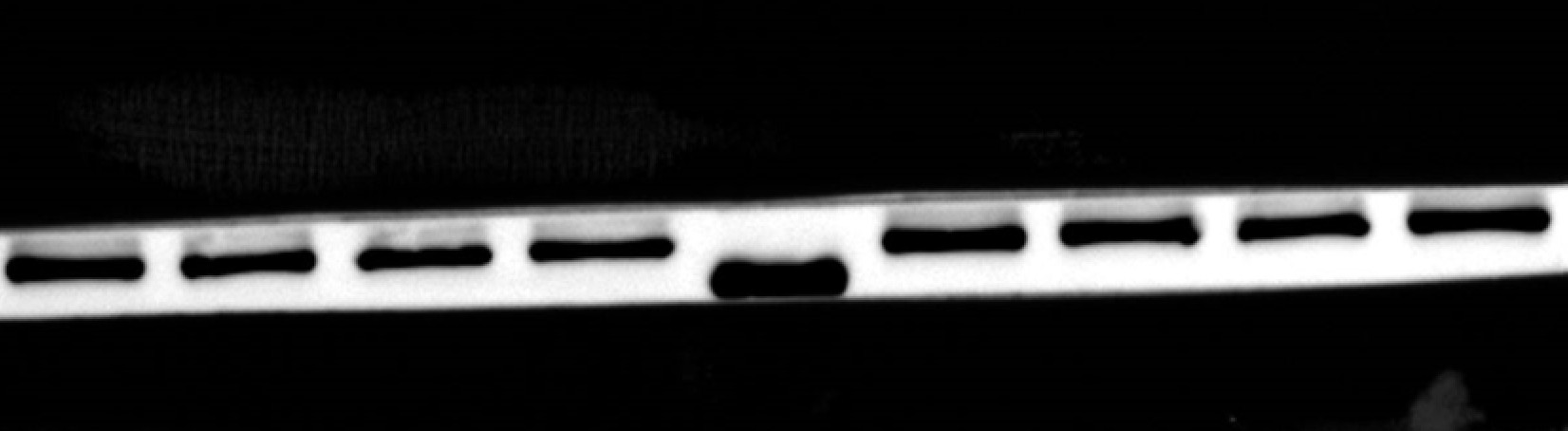

Supplement: Supplementary file 8 [file DataSheet13.ZIP › Original data of Figure 13/Figure 13A-GAPDH-original image-2.jpg]

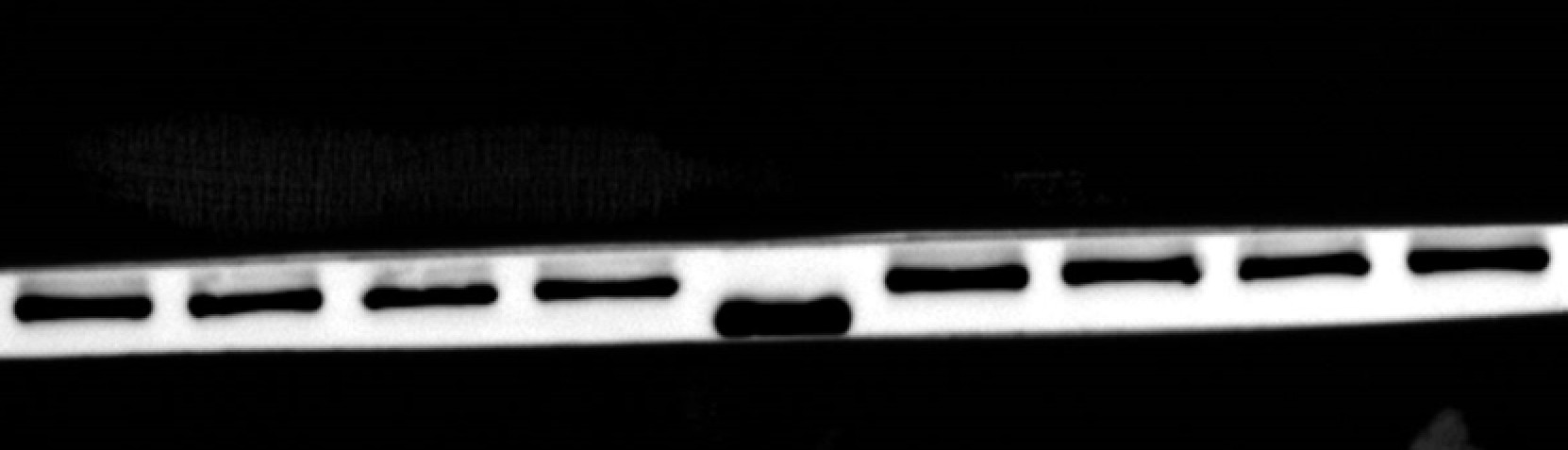

Supplement: Supplementary file 8 [file DataSheet13.ZIP › Original data of Figure 13/Figure 13A-GAPDH-original image-3.jpg]

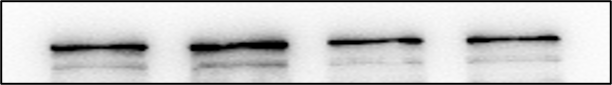

Supplement: Supplementary file 8 [file DataSheet13.ZIP › Original data of Figure 13/Figure 13A-mTORC1-1.tif]

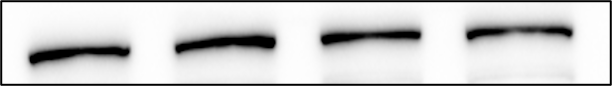

Supplement: Supplementary file 8 [file DataSheet13.ZIP › Original data of Figure 13/Figure 13A-mTORC1-2.tif]

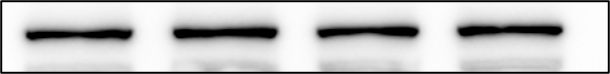

Supplement: Supplementary file 8 [file DataSheet13.ZIP › Original data of Figure 13/Figure 13A-mTORC1-3.tif]

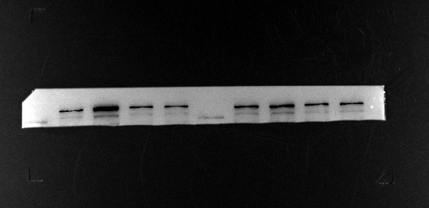

Supplement: Supplementary file 8 [file DataSheet13.ZIP › Original data of Figure 13/Figure 13A-mTORC1-original image-1.tif]

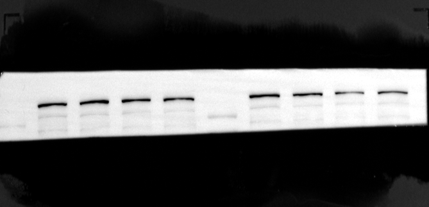

Supplement: Supplementary file 8 [file DataSheet13.ZIP › Original data of Figure 13/Figure 13A-mTORC1-original image-2.tif]

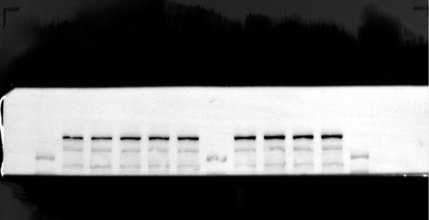

Supplement: Supplementary file 8 [file DataSheet13.ZIP › Original data of Figure 13/Figure 13A-mTORC1-original image-3.tif]

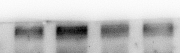

Supplement: Supplementary file 8 [file DataSheet13.ZIP › Original data of Figure 13/Figure 13A-NLRP3-1.tif]

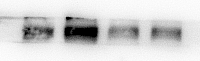

Supplement: Supplementary file 8 [file DataSheet13.ZIP › Original data of Figure 13/Figure 13A-NLRP3-2.tif]

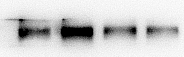

Supplement: Supplementary file 8 [file DataSheet13.ZIP › Original data of Figure 13/Figure 13A-NLRP3-3.tif]
