# Supplementary material for: Fucoidan Alleviates Renal Fibrosis in Diabetic Kidney Disease via Inhibition of NLRP3 Inflammasome-Mediated Podocyte Pyroptosis
Source: Front Pharmacol. 2022 Mar 18;13:790937. doi: 10.3389/fphar.2022.790937 (PMC8972405; doi:10.3389/fphar.2022.790937)

GSDMD HG

Original image 1

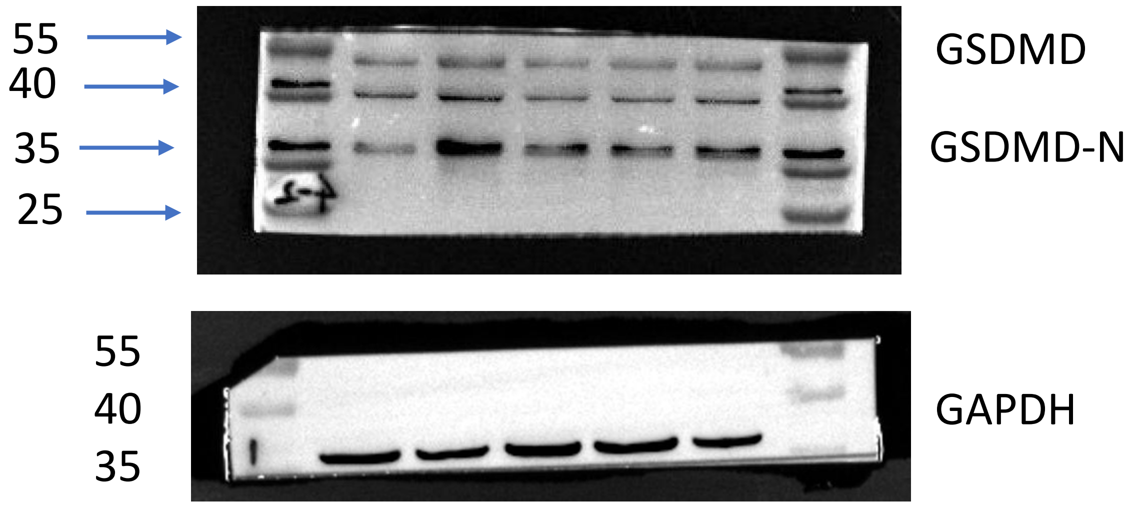

Original image 2

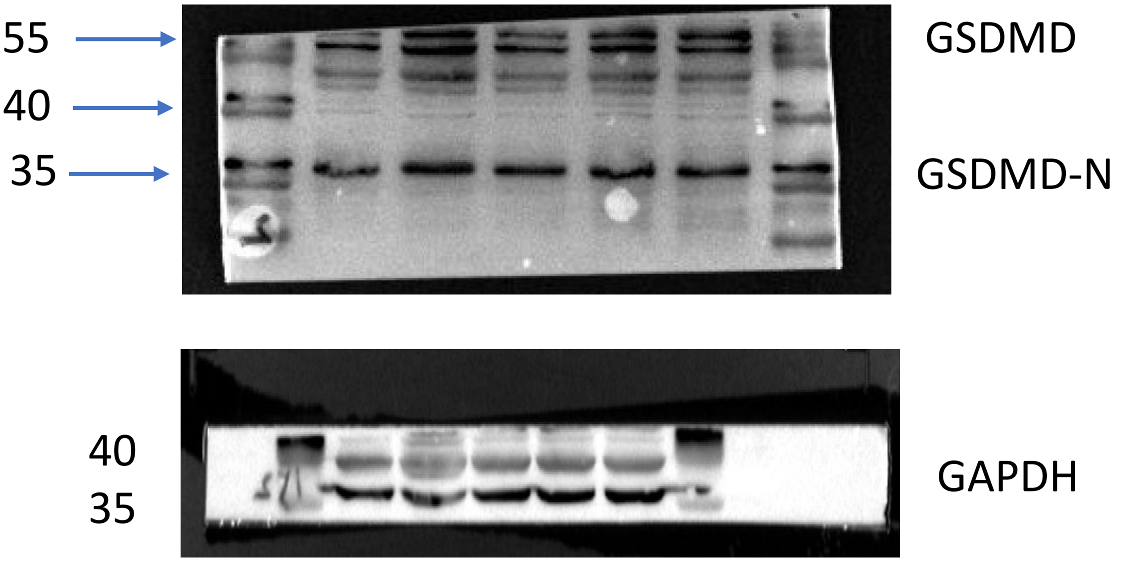

Original image 3

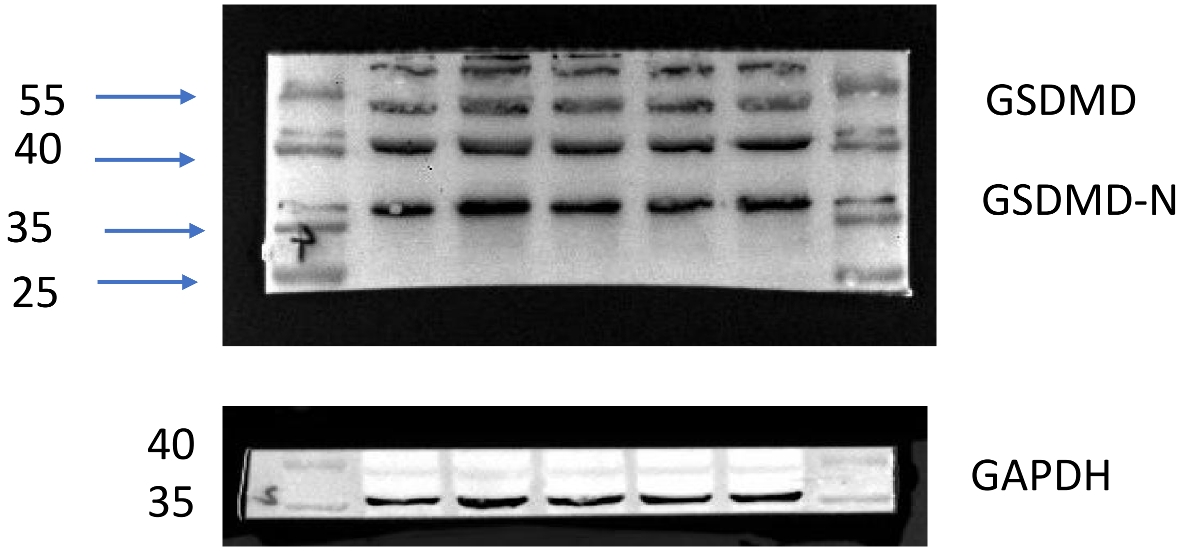

GSDMD LPS

Original image 1

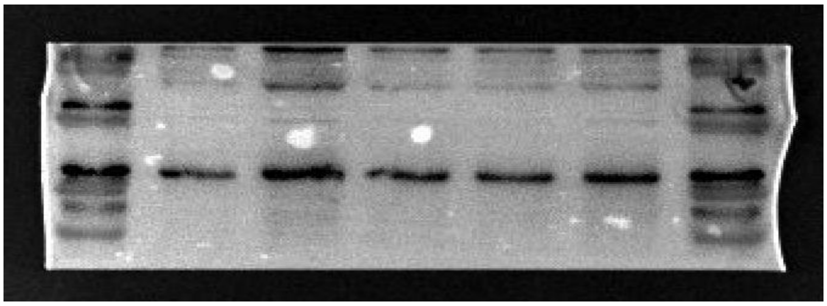

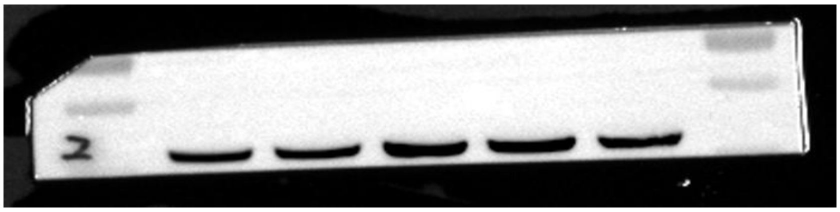

Original image 2

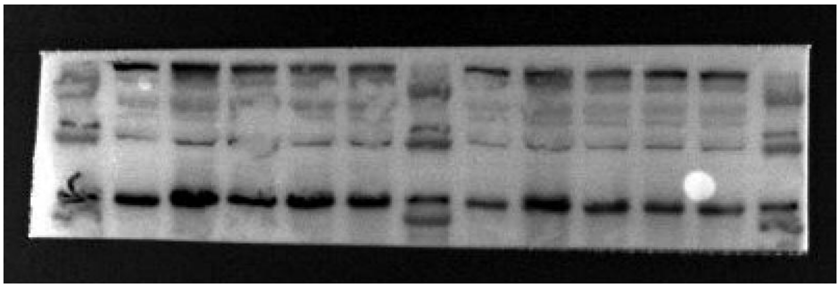

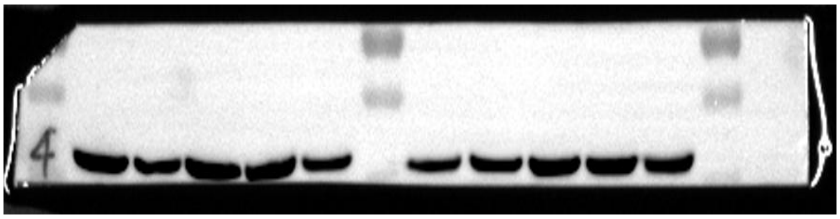

Original image 3

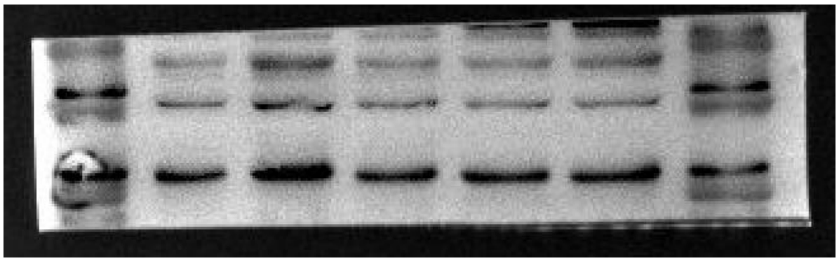

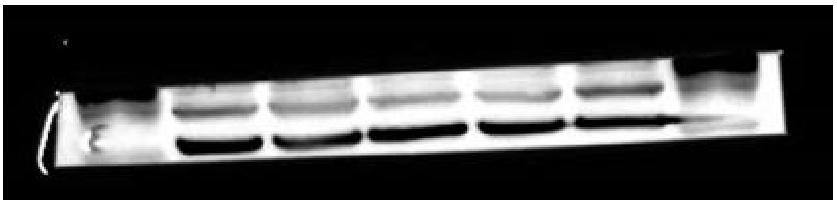

Supplement: Supplementary file 4 [file DataSheet8.ZIP › Original data of Figure 8/Figure 8B and D-GSDMD and GSDMD-N-original image-1-3.pdf]
