# Supplementary figures and images for: Fucoidan Alleviates Renal Fibrosis in Diabetic Kidney Disease via Inhibition of NLRP3 Inflammasome-Mediated Podocyte Pyroptosis (part 2 of 3)
Source: Front Pharmacol. 2022 Mar 18;13:790937. doi: 10.3389/fphar.2022.790937 (PMC8972405; doi:10.3389/fphar.2022.790937)

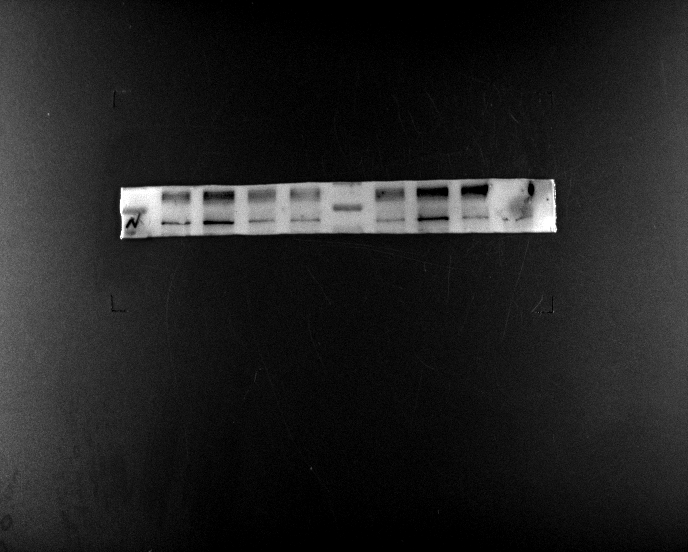

Supplement: Supplementary file 8 [file DataSheet13.ZIP › Original data of Figure 13/Figure 13A-NLRP3-original image-1.tif]

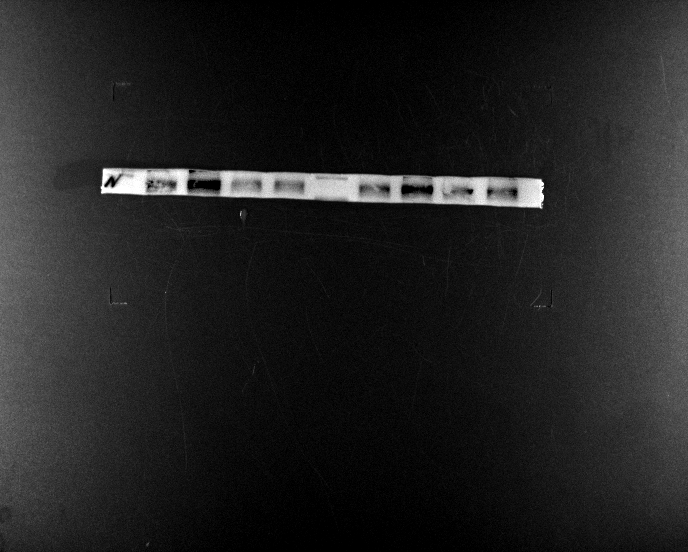

Supplement: Supplementary file 8 [file DataSheet13.ZIP › Original data of Figure 13/Figure 13A-NLRP3-original image-2.tif]

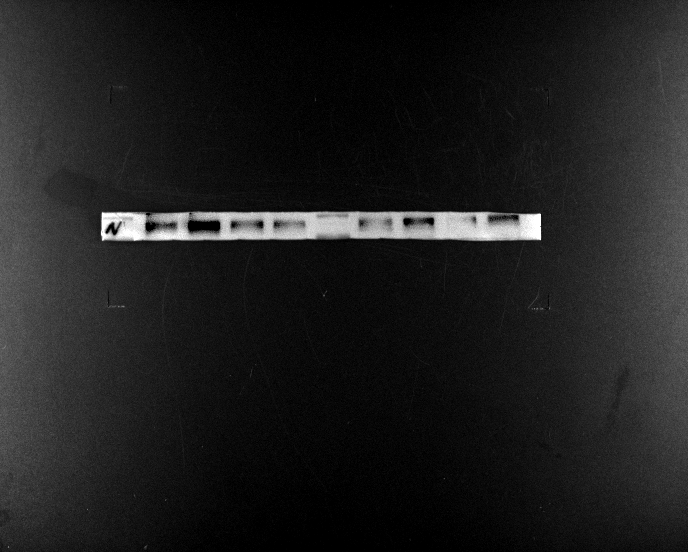

Supplement: Supplementary file 8 [file DataSheet13.ZIP › Original data of Figure 13/Figure 13A-NLRP3-original image-3.tif]

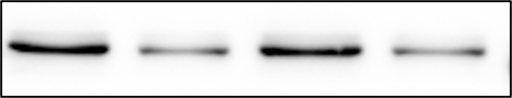

Supplement: Supplementary file 8 [file DataSheet13.ZIP › Original data of Figure 13/Figure 13A-p-AMPK-1.tif]

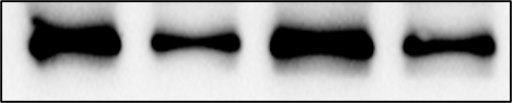

Supplement: Supplementary file 8 [file DataSheet13.ZIP › Original data of Figure 13/Figure 13A-p-AMPK-2.tif]

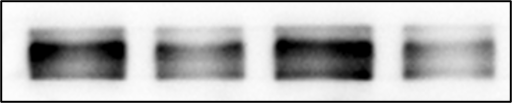

Supplement: Supplementary file 8 [file DataSheet13.ZIP › Original data of Figure 13/Figure 13A-p-AMPK-3.tif]

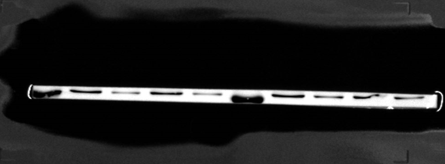

Supplement: Supplementary file 8 [file DataSheet13.ZIP › Original data of Figure 13/Figure 13A-p-AMPK-original image-1.tif]

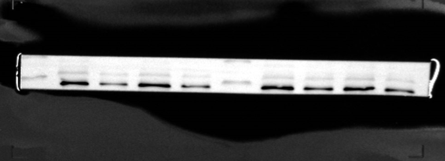

Supplement: Supplementary file 8 [file DataSheet13.ZIP › Original data of Figure 13/Figure 13A-p-AMPK-original image-2.tif]

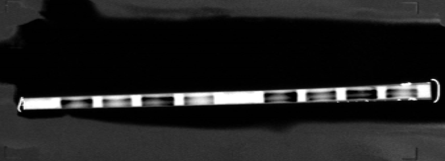

Supplement: Supplementary file 8 [file DataSheet13.ZIP › Original data of Figure 13/Figure 13A-p-AMPK-original image-3.tif]

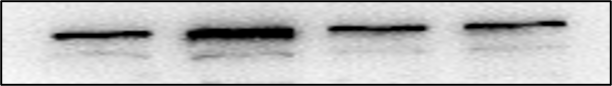

Supplement: Supplementary file 8 [file DataSheet13.ZIP › Original data of Figure 13/Figure 13A-p-mTORC1-1.tif]

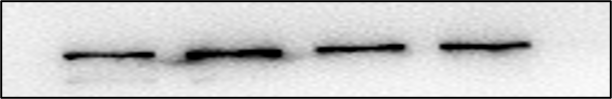

Supplement: Supplementary file 8 [file DataSheet13.ZIP › Original data of Figure 13/Figure 13A-p-mTORC1-2.tif]

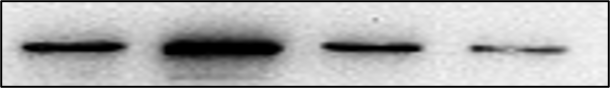

Supplement: Supplementary file 8 [file DataSheet13.ZIP › Original data of Figure 13/Figure 13A-p-mTORC1-3.tif]

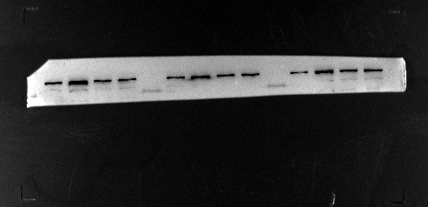

Supplement: Supplementary file 8 [file DataSheet13.ZIP › Original data of Figure 13/Figure 13A-p-mTORC1-original image-1.tif]

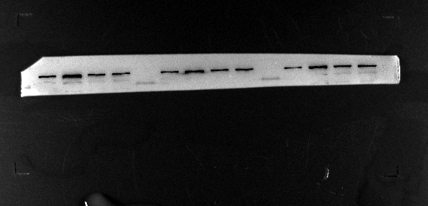

Supplement: Supplementary file 8 [file DataSheet13.ZIP › Original data of Figure 13/Figure 13A-p-mTORC1-original image-2.tif]

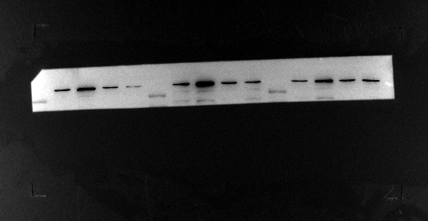

Supplement: Supplementary file 8 [file DataSheet13.ZIP › Original data of Figure 13/Figure 13A-p-mTORC1-original image-3.tif]

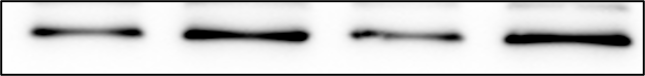

Supplement: Supplementary file 8 [file DataSheet13.ZIP › Original data of Figure 13/Figure 13A-p-raptor-1.tif]

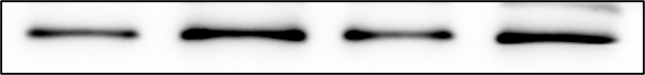

Supplement: Supplementary file 8 [file DataSheet13.ZIP › Original data of Figure 13/Figure 13A-p-raptor-2.tif]

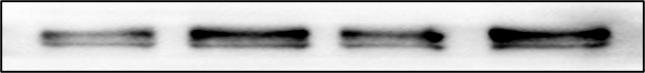

Supplement: Supplementary file 8 [file DataSheet13.ZIP › Original data of Figure 13/Figure 13A-p-raptor-3.tif]

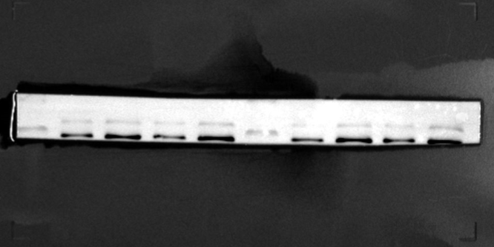

Supplement: Supplementary file 8 [file DataSheet13.ZIP › Original data of Figure 13/Figure 13A-p-raptor-original image-1.tif]

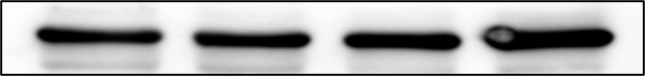

Supplement: Supplementary file 8 [file DataSheet13.ZIP › Original data of Figure 13/Figure 13A-raptor-1.tif]

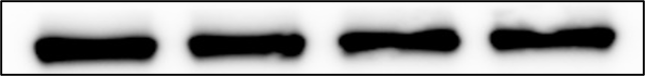

Supplement: Supplementary file 8 [file DataSheet13.ZIP › Original data of Figure 13/Figure 13A-raptor-2.tif]

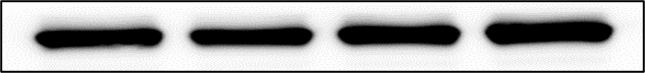

Supplement: Supplementary file 8 [file DataSheet13.ZIP › Original data of Figure 13/Figure 13A-raptor-3.tif]

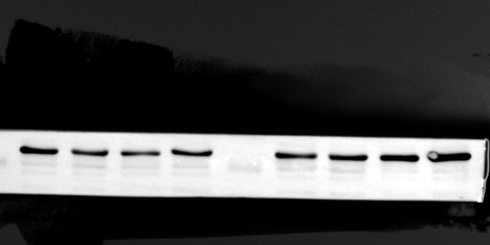

Supplement: Supplementary file 8 [file DataSheet13.ZIP › Original data of Figure 13/Figure 13A-raptor-original image-1.tif]

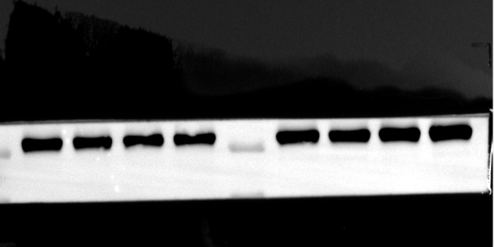

Supplement: Supplementary file 8 [file DataSheet13.ZIP › Original data of Figure 13/Figure 13A-raptor-original image-2.tif]

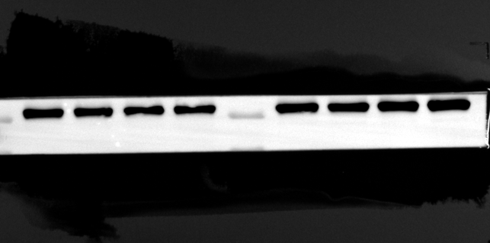

Supplement: Supplementary file 8 [file DataSheet13.ZIP › Original data of Figure 13/Figure 13A-raptor-original image-3.tif]

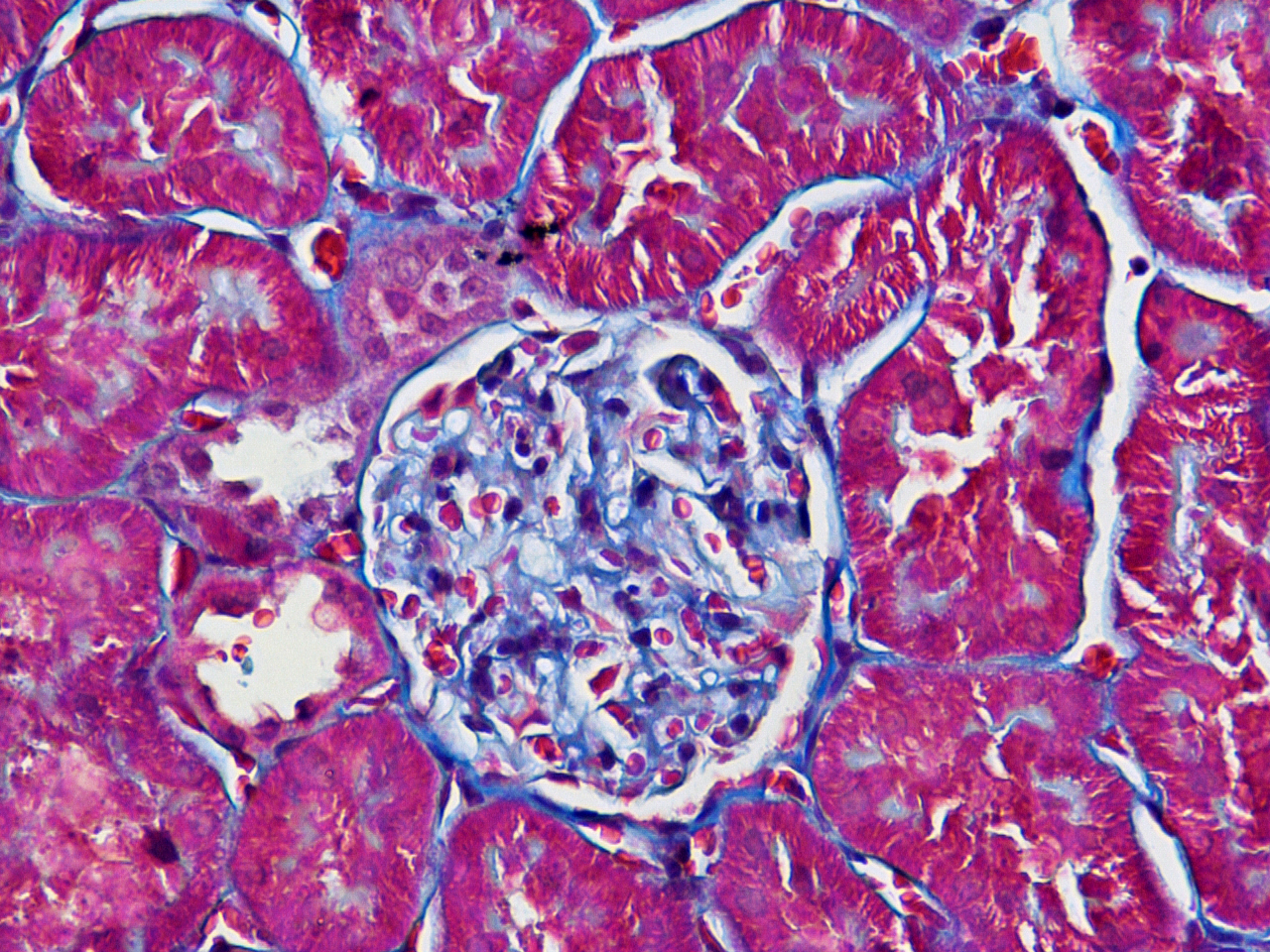

Supplement: Supplementary file 9 [file DataSheet1.ZIP › Original data of Figure 1/Figure 1A-Masson (FPS).jpg]

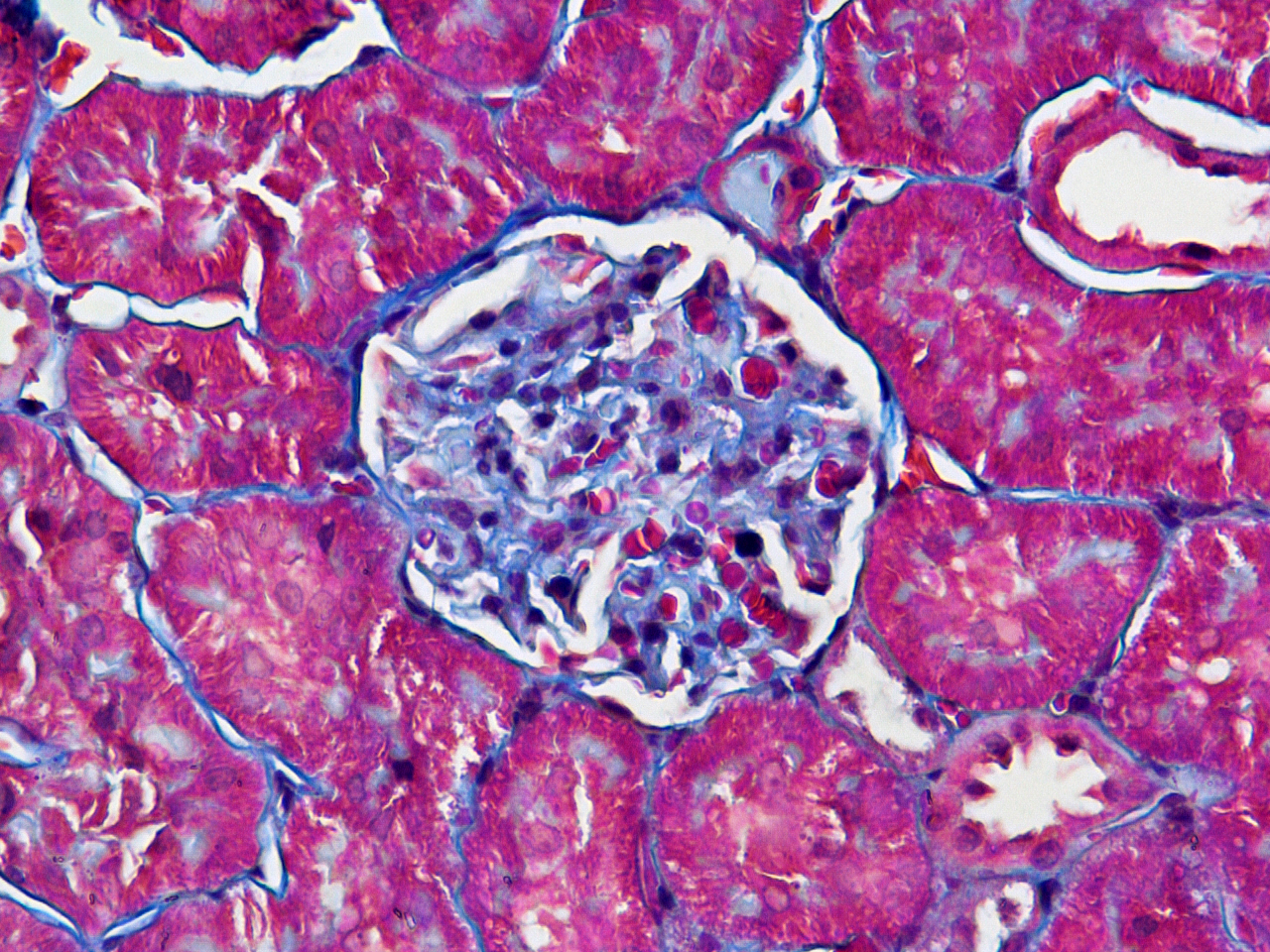

Supplement: Supplementary file 9 [file DataSheet1.ZIP › Original data of Figure 1/Figure 1A-Masson (RAP).jpg]

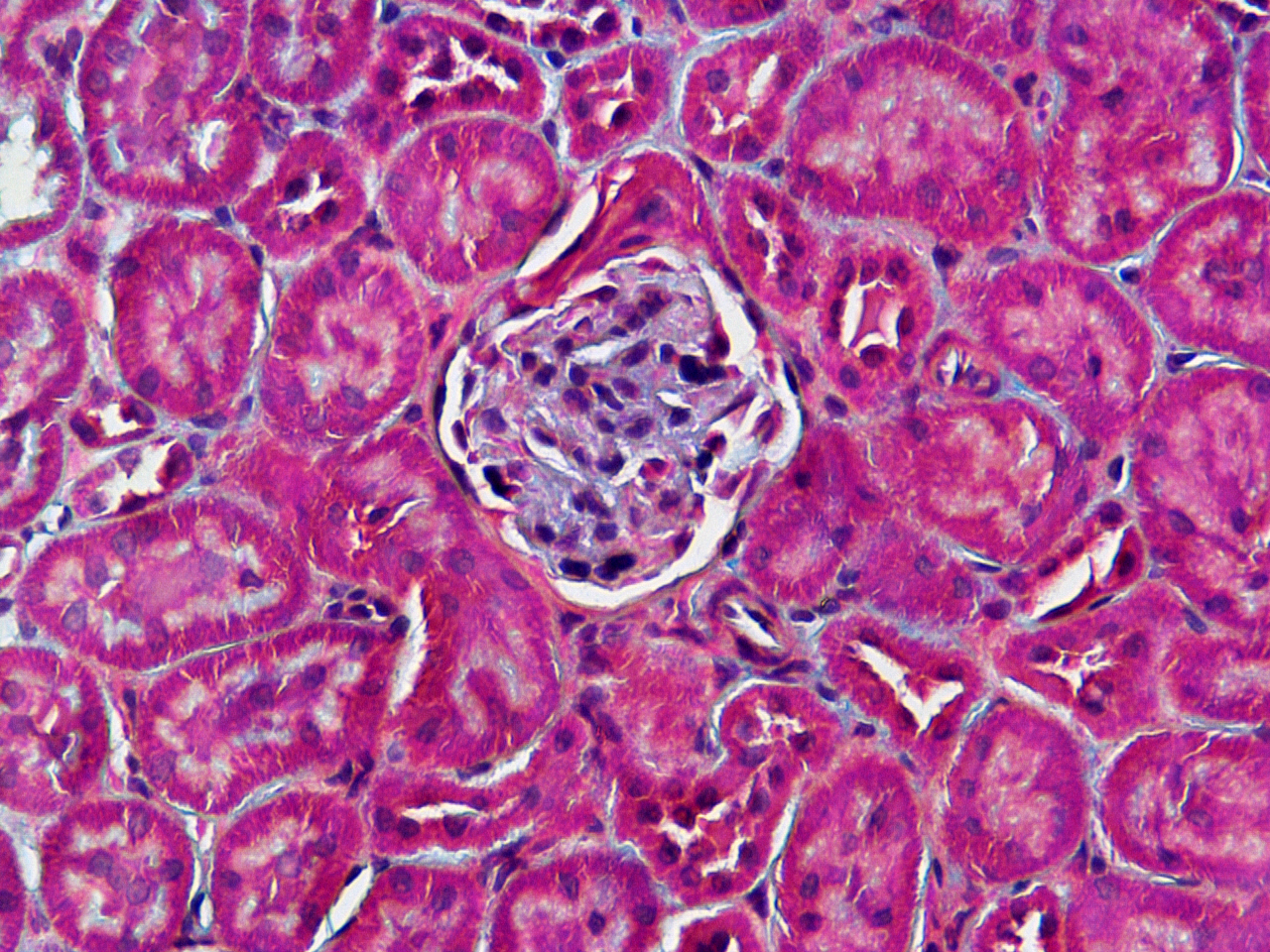

Supplement: Supplementary file 9 [file DataSheet1.ZIP › Original data of Figure 1/Figure 1A-Masson (Sham).jpg]

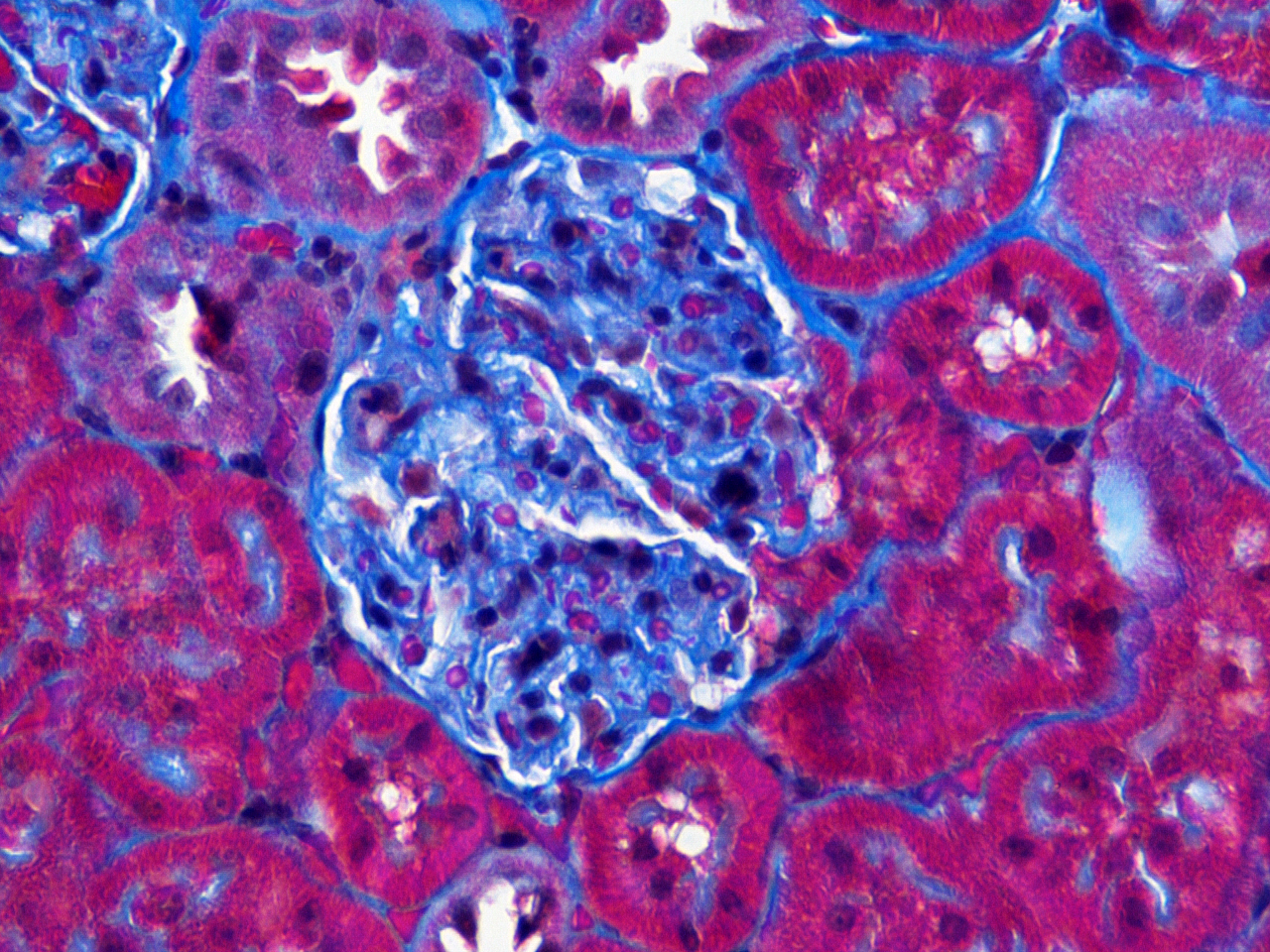

Supplement: Supplementary file 9 [file DataSheet1.ZIP › Original data of Figure 1/Figure 1A-Masson (Vehicle).jpg]

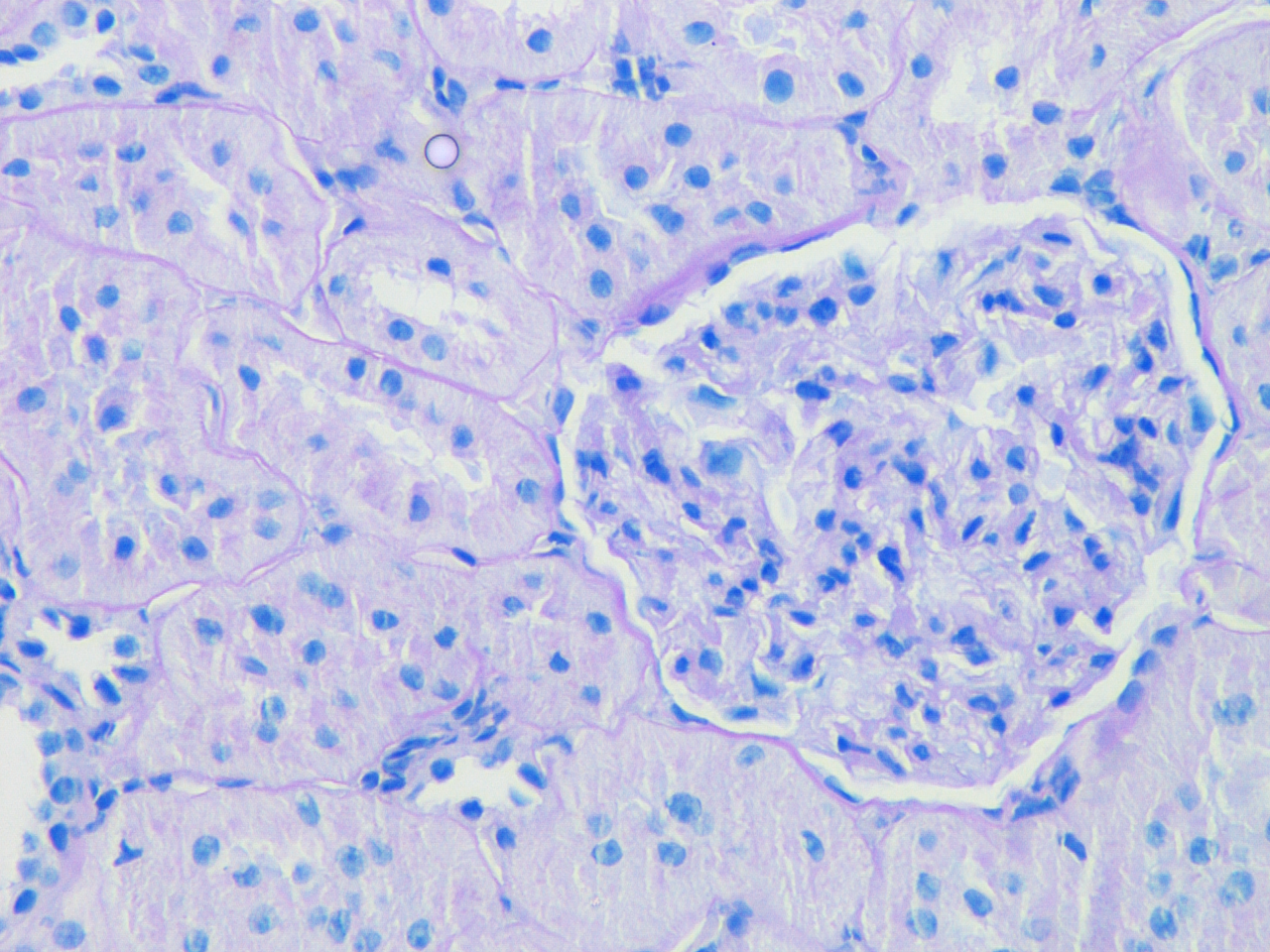

Supplement: Supplementary file 9 [file DataSheet1.ZIP › Original data of Figure 1/Figure 1A-PAS (FPS).tif]

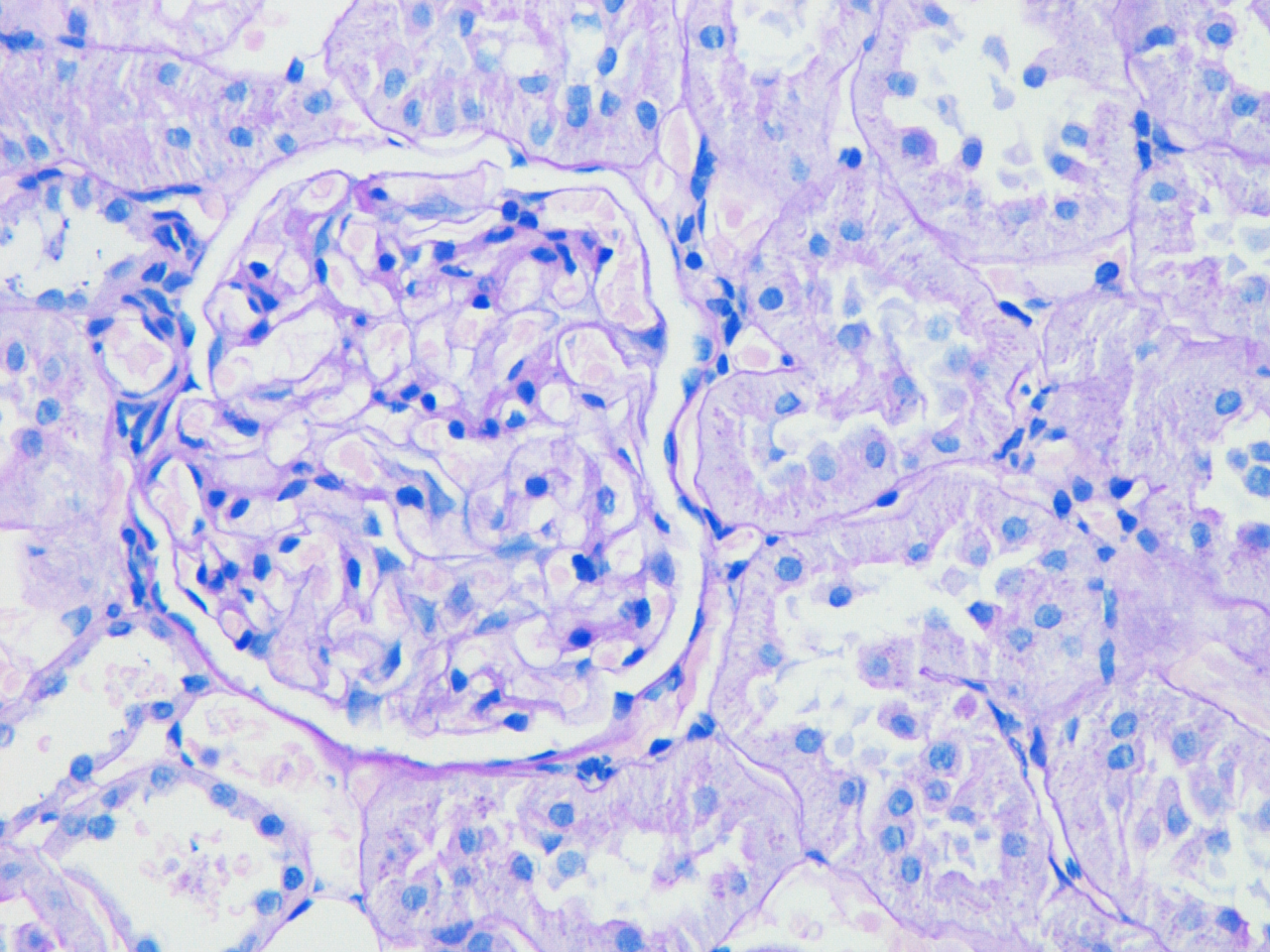

Supplement: Supplementary file 9 [file DataSheet1.ZIP › Original data of Figure 1/Figure 1A-PAS (RAP).tif]

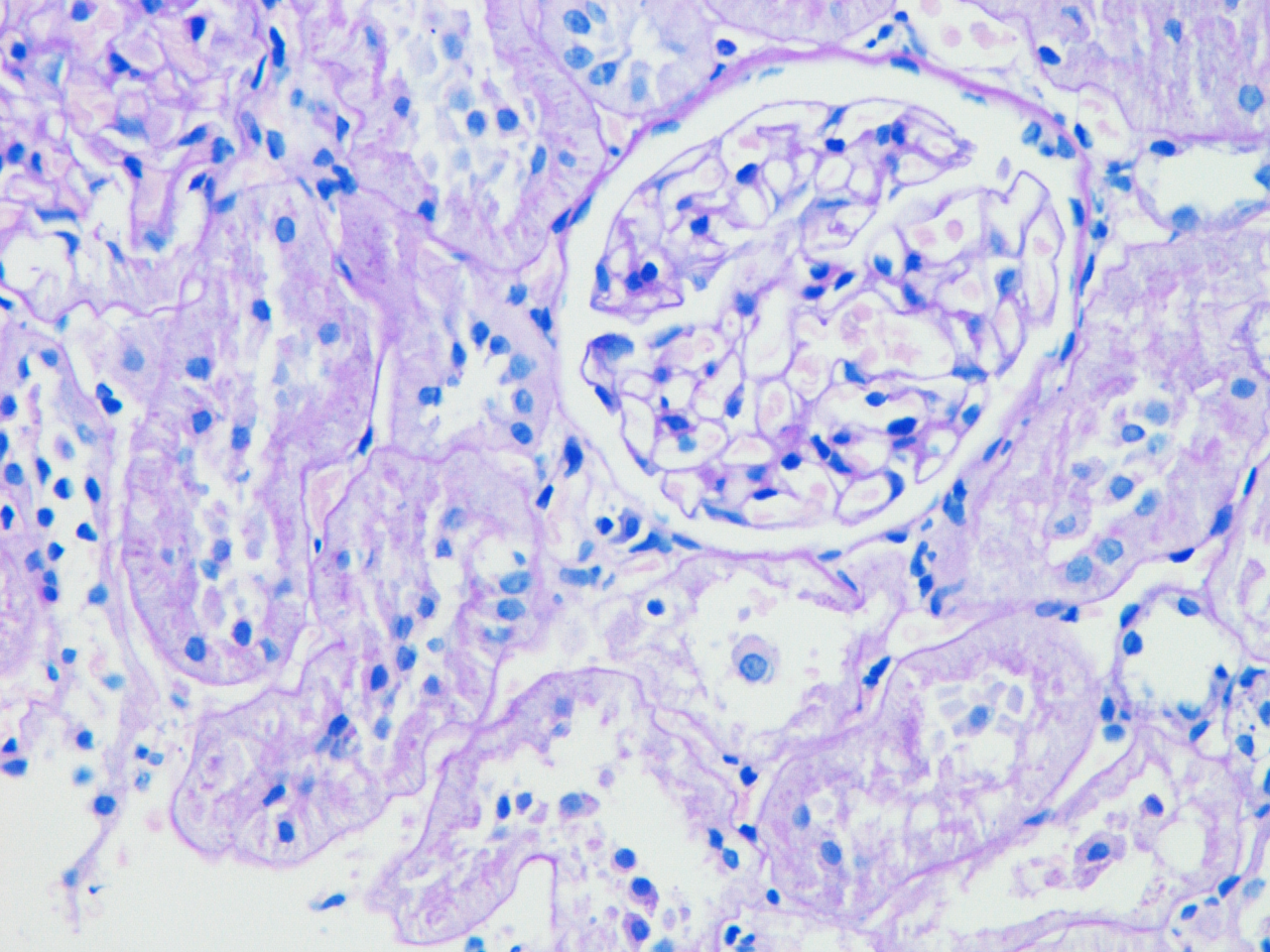

Supplement: Supplementary file 9 [file DataSheet1.ZIP › Original data of Figure 1/Figure 1A-PAS (Sham).tif]

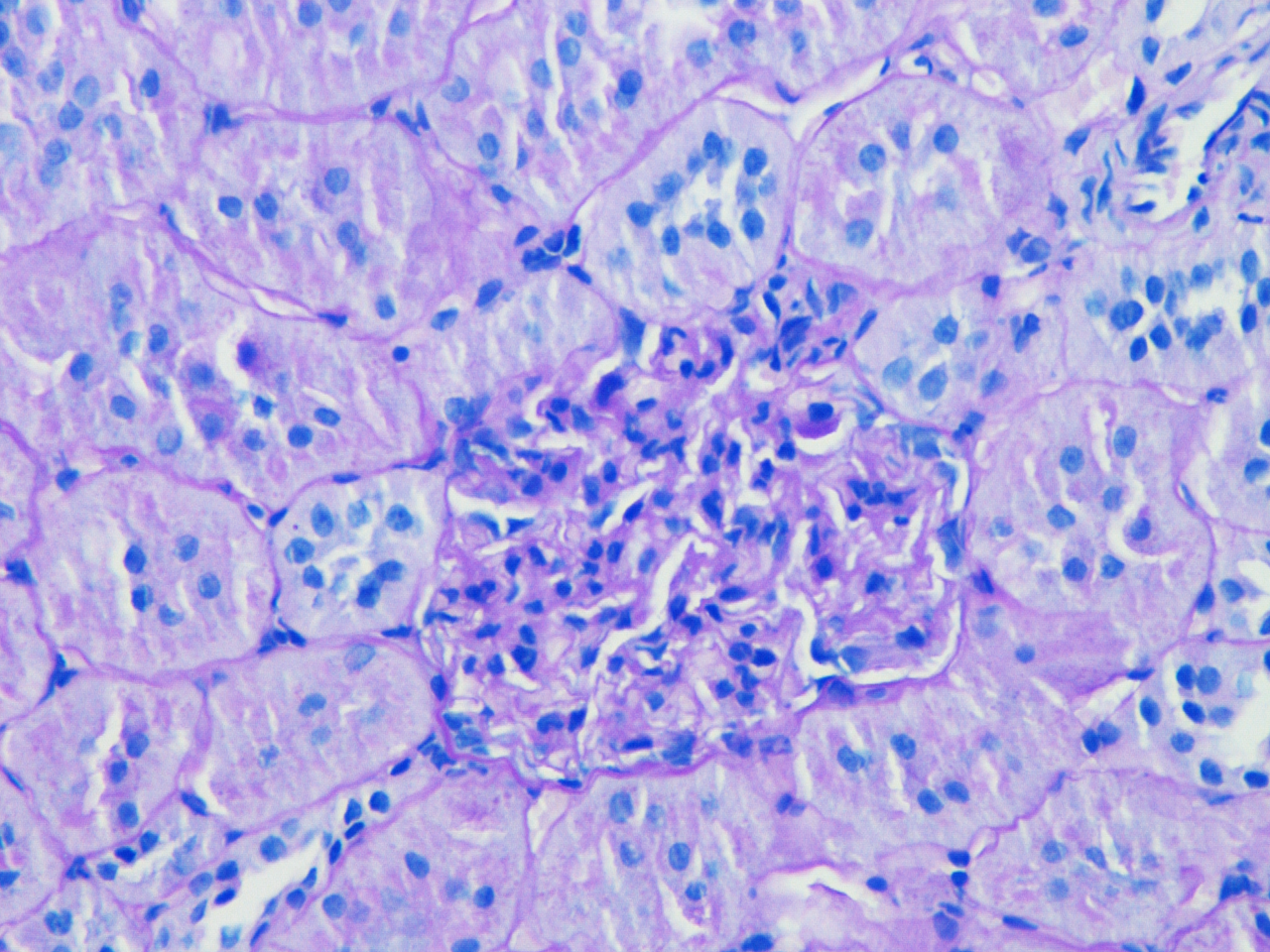

Supplement: Supplementary file 9 [file DataSheet1.ZIP › Original data of Figure 1/Figure 1A-PAS (Vehicle).tif]

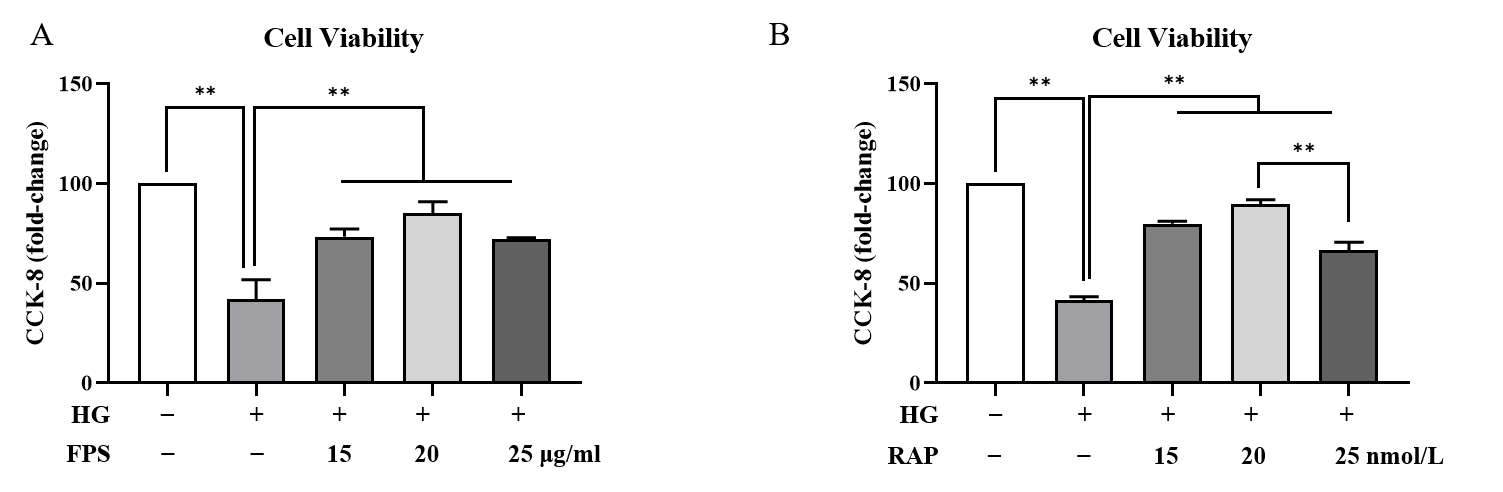

Supplement: Supplementary file 10 [file Image2.TIF]

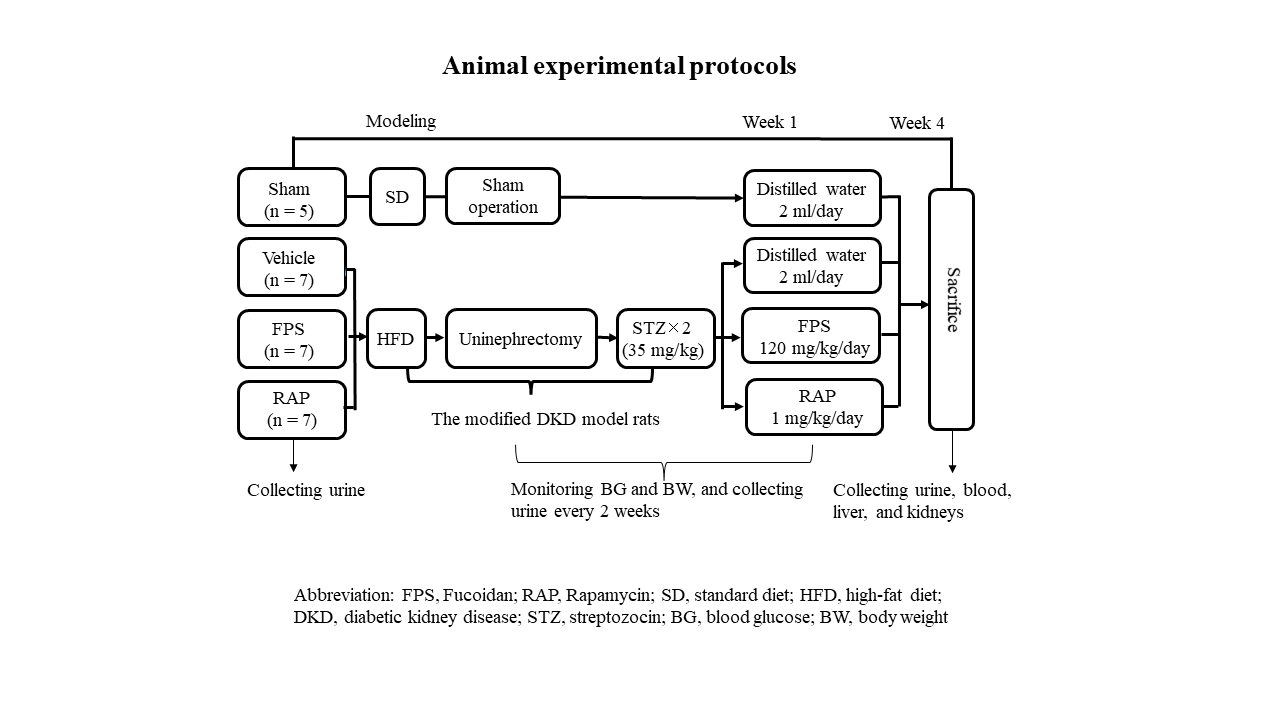

Supplement: Supplementary file 11 [file Image1.TIF]

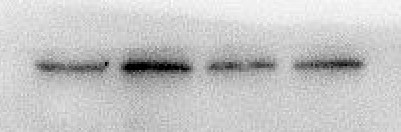

Supplement: Supplementary file 12 [file DataSheet10.ZIP › Original data of Figure 10/Figure 10A-ASC-1.jpg]

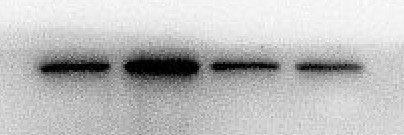

Supplement: Supplementary file 12 [file DataSheet10.ZIP › Original data of Figure 10/Figure 10A-ASC-2.jpg]

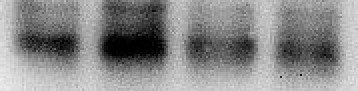

Supplement: Supplementary file 12 [file DataSheet10.ZIP › Original data of Figure 10/Figure 10A-ASC-3.jpg]

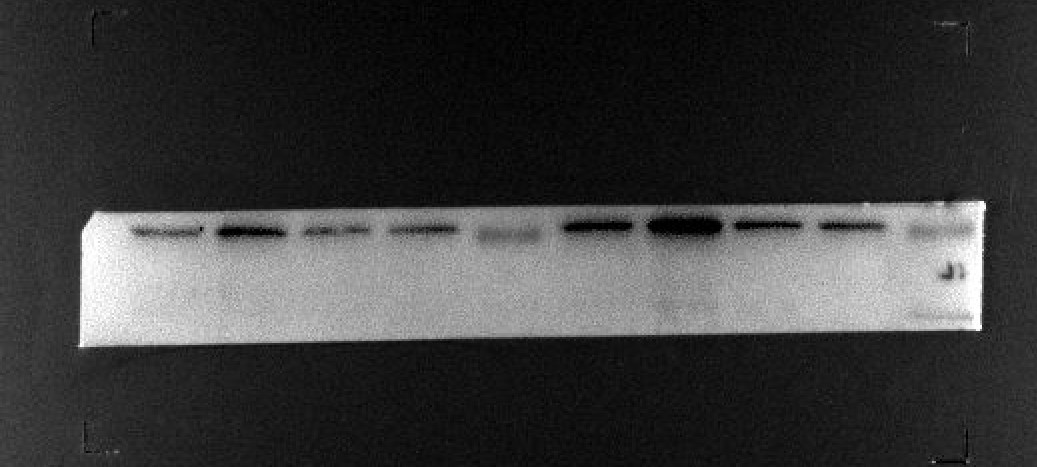

Supplement: Supplementary file 12 [file DataSheet10.ZIP › Original data of Figure 10/Figure 10A-ASC-original image-1.jpg]

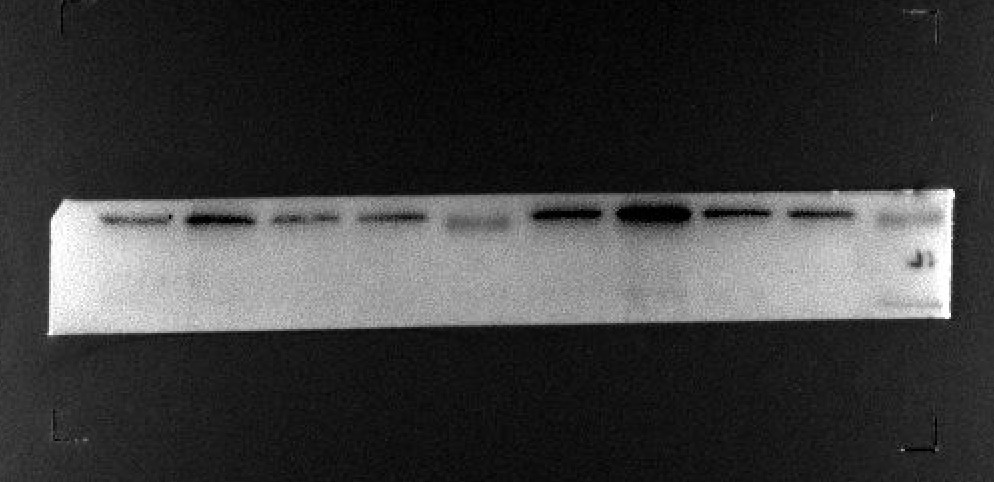

Supplement: Supplementary file 12 [file DataSheet10.ZIP › Original data of Figure 10/Figure 10A-ASC-original image-2.jpg]

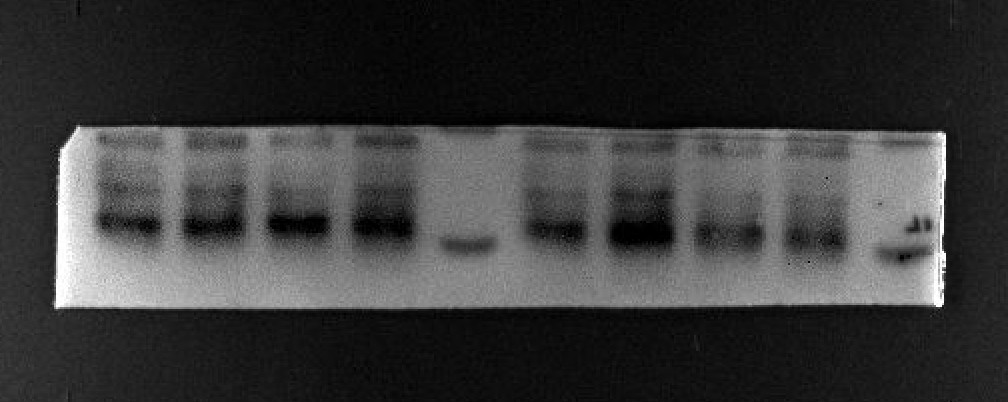

Supplement: Supplementary file 12 [file DataSheet10.ZIP › Original data of Figure 10/Figure 10A-ASC-original image-3.jpg]

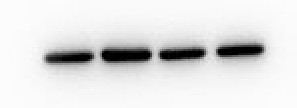

Supplement: Supplementary file 12 [file DataSheet10.ZIP › Original data of Figure 10/Figure 10A-GAPDH-1.jpg]

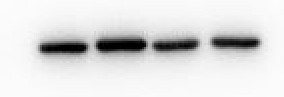

Supplement: Supplementary file 12 [file DataSheet10.ZIP › Original data of Figure 10/Figure 10A-GAPDH-2.jpg]

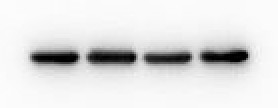

Supplement: Supplementary file 12 [file DataSheet10.ZIP › Original data of Figure 10/Figure 10A-GAPDH-3.jpg]

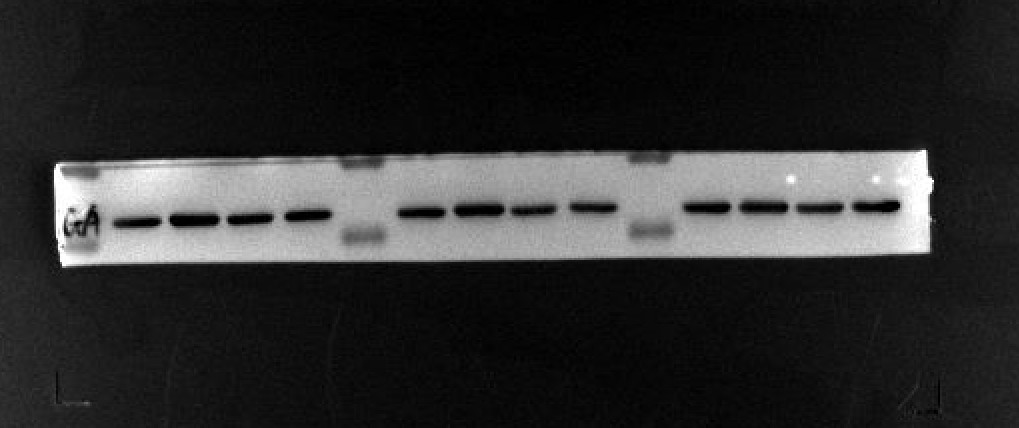

Supplement: Supplementary file 12 [file DataSheet10.ZIP › Original data of Figure 10/Figure 10A-GAPDH-original image-1.jpg]

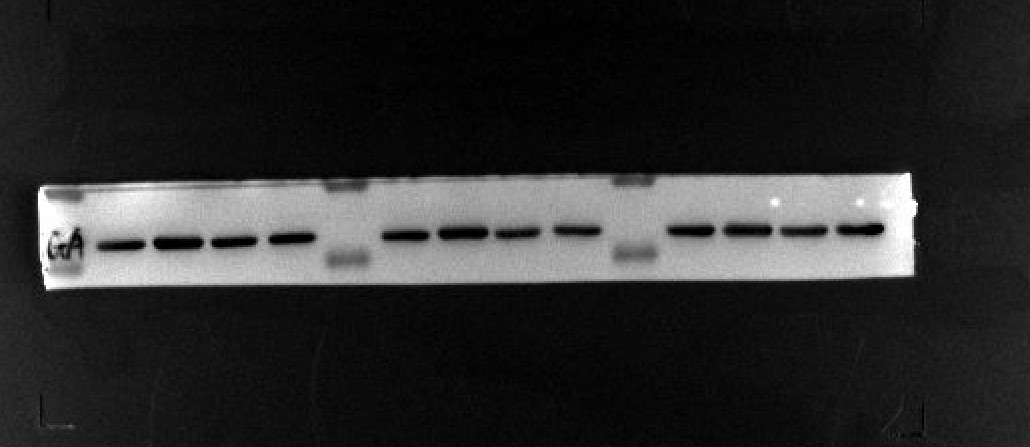

Supplement: Supplementary file 12 [file DataSheet10.ZIP › Original data of Figure 10/Figure 10A-GAPDH-original image-2.jpg]

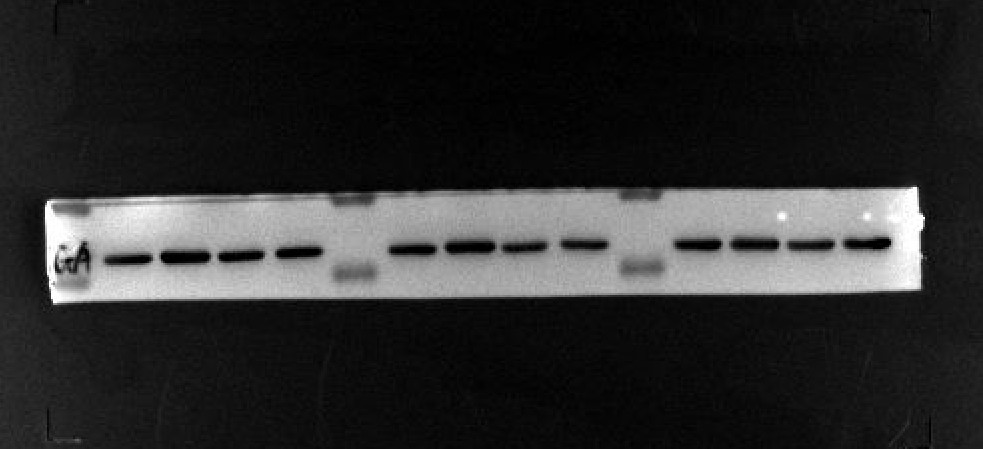

Supplement: Supplementary file 12 [file DataSheet10.ZIP › Original data of Figure 10/Figure 10A-GAPDH-original image-3.jpg]

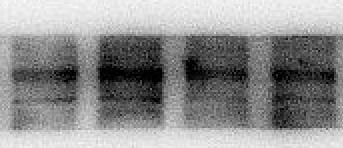

Supplement: Supplementary file 12 [file DataSheet10.ZIP › Original data of Figure 10/Figure 10A-NLRP3-1.jpg]

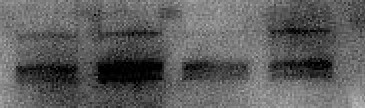

Supplement: Supplementary file 12 [file DataSheet10.ZIP › Original data of Figure 10/Figure 10A-NLRP3-2.jpg]

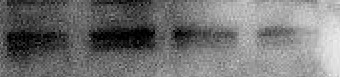

Supplement: Supplementary file 12 [file DataSheet10.ZIP › Original data of Figure 10/Figure 10A-NLRP3-3.jpg]

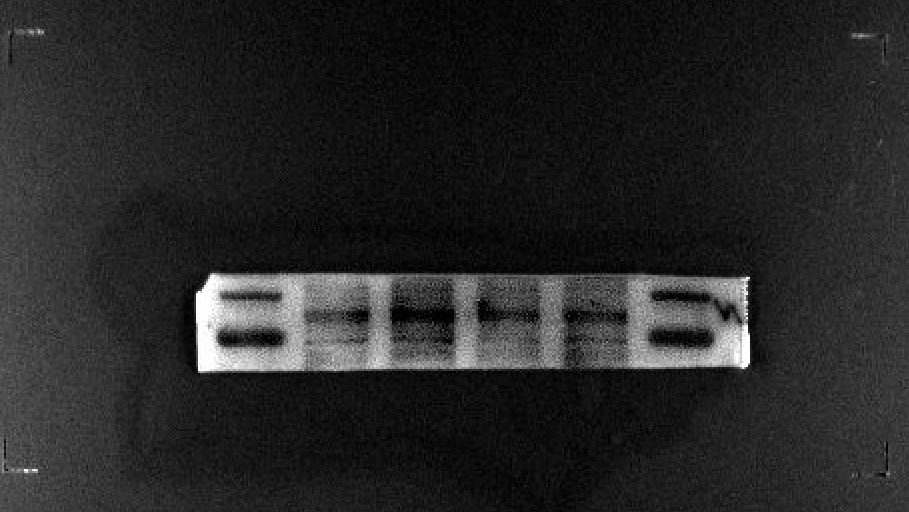

Supplement: Supplementary file 12 [file DataSheet10.ZIP › Original data of Figure 10/Figure 10A-NLRP3-original image-1.jpg]

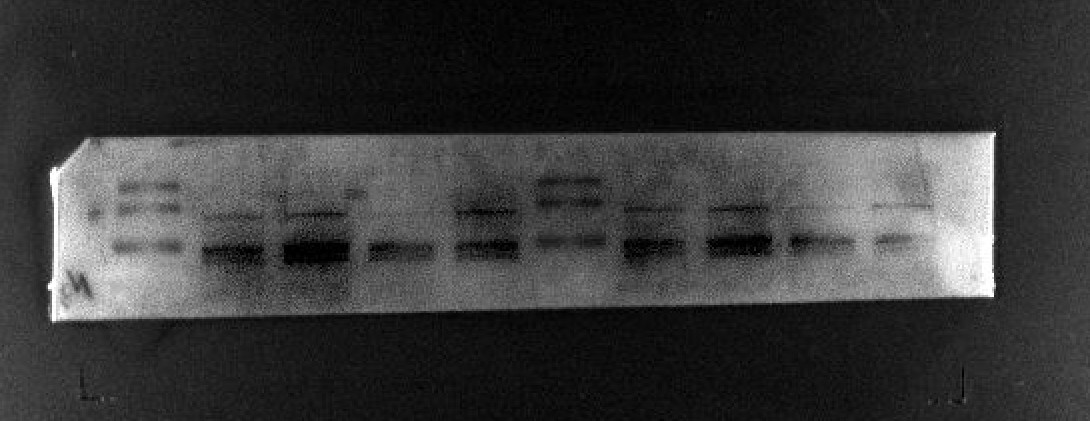

Supplement: Supplementary file 12 [file DataSheet10.ZIP › Original data of Figure 10/Figure 10A-NLRP3-original image-2.jpg]

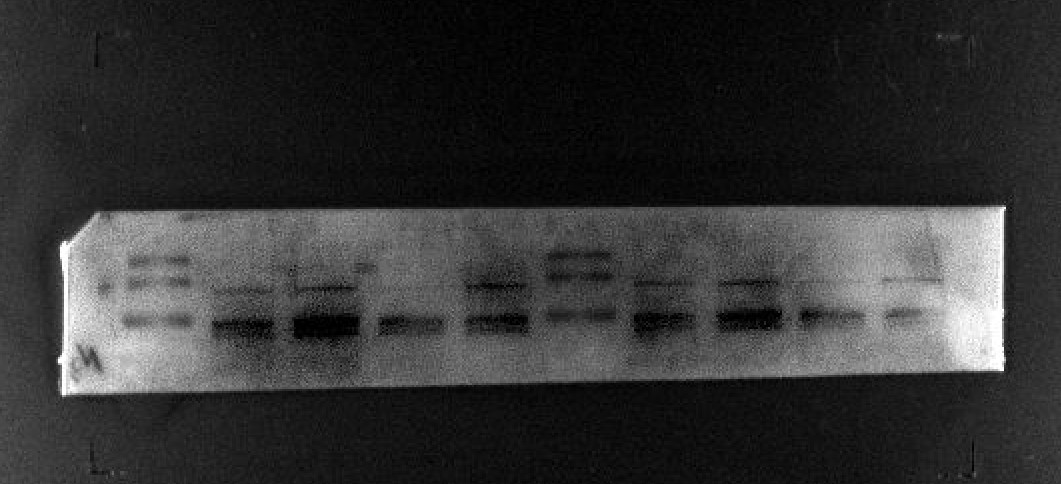

Supplement: Supplementary file 12 [file DataSheet10.ZIP › Original data of Figure 10/Figure 10A-NLRP3-original image-3.jpg]

Caspase-1

Original  
image 1

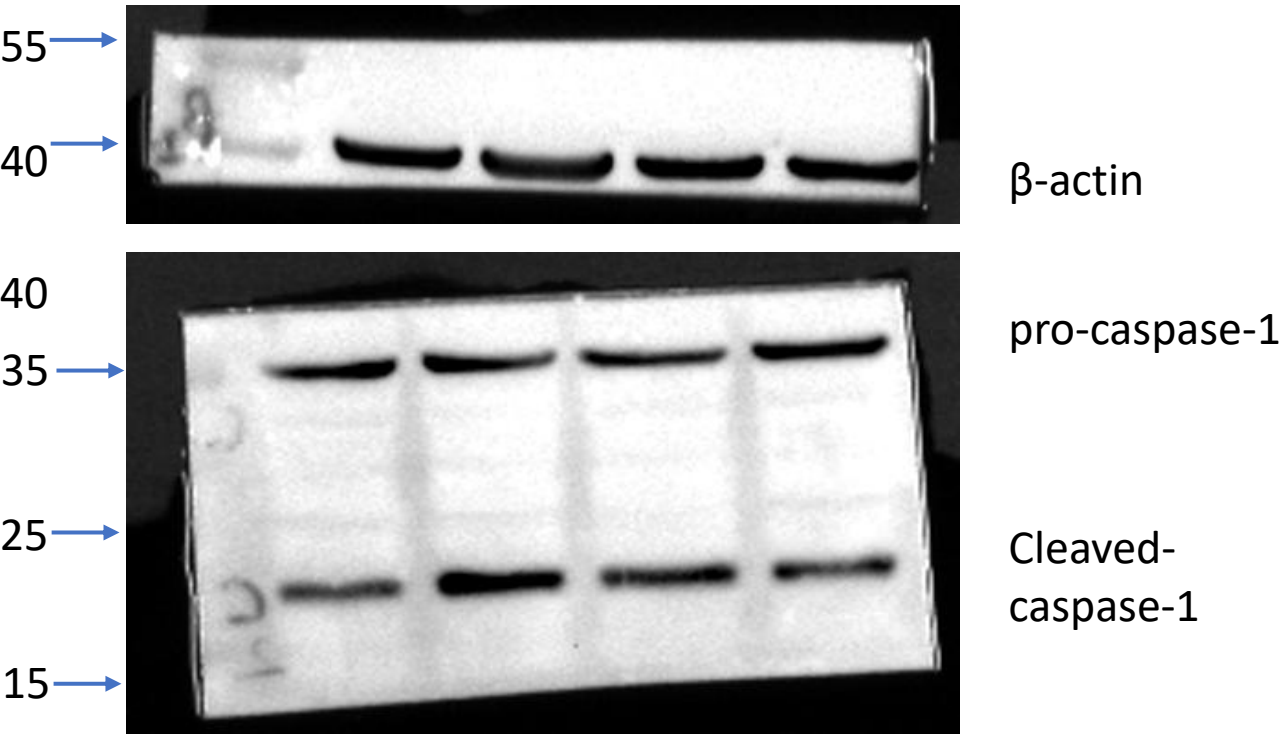

Original  
image 2

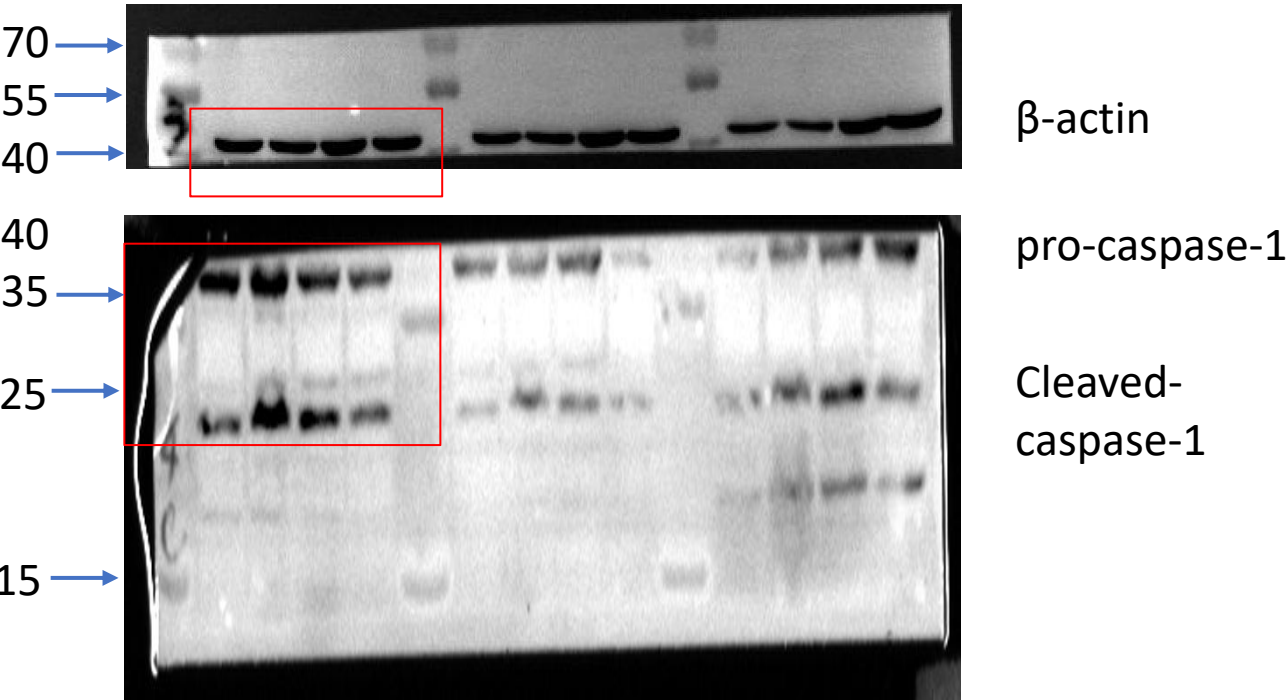

Original  
image 3

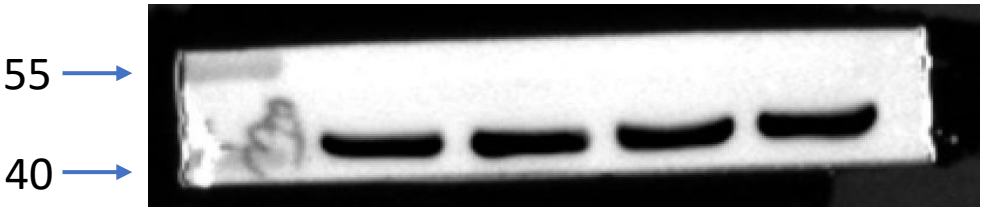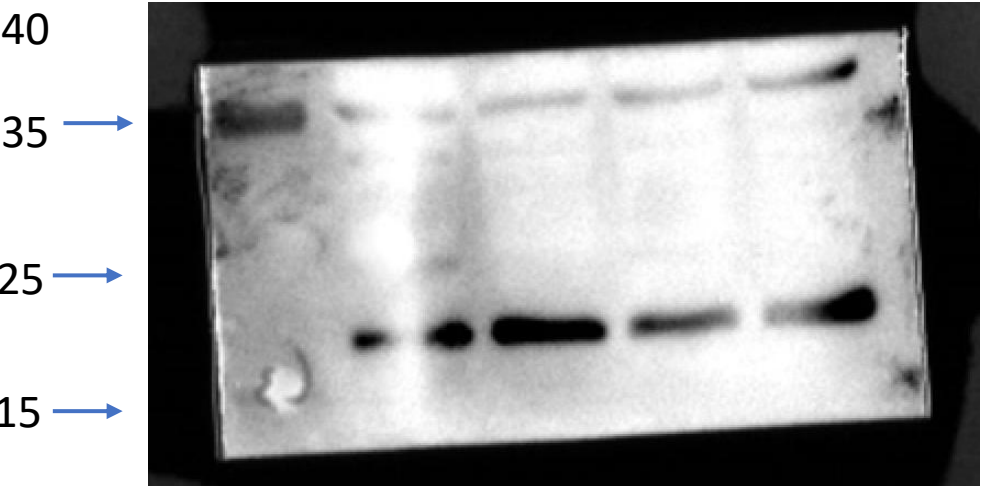

Supplement: Supplementary file 12 [file DataSheet10.ZIP › Original data of Figure 10/Figure 10A-pro-Caspase-1, cleaved-Caspase-1 and β-actin-original image-1-3.pdf]

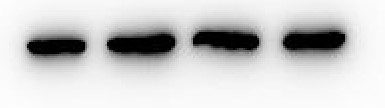

Supplement: Supplementary file 13 [file DataSheet6.ZIP › Original data of Figure 6/Figure 6A-GAPDH-1.jpg]

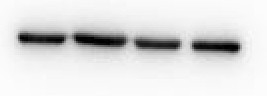

Supplement: Supplementary file 13 [file DataSheet6.ZIP › Original data of Figure 6/Figure 6A-GAPDH-2.jpg]

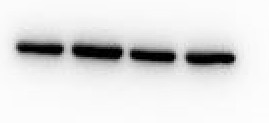

Supplement: Supplementary file 13 [file DataSheet6.ZIP › Original data of Figure 6/Figure 6A-GAPDH-3.jpg]

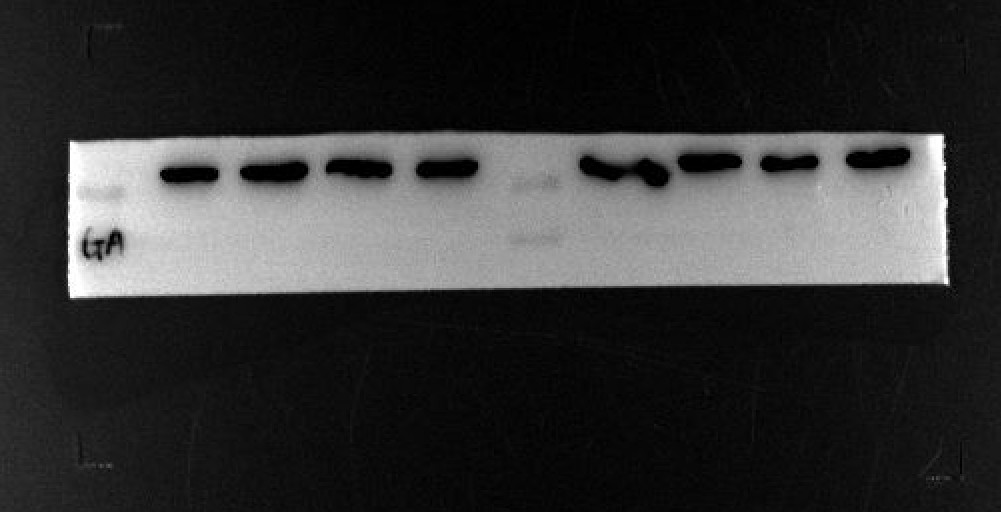

Supplement: Supplementary file 13 [file DataSheet6.ZIP › Original data of Figure 6/Figure 6A-GAPDH-original image-1.jpg]

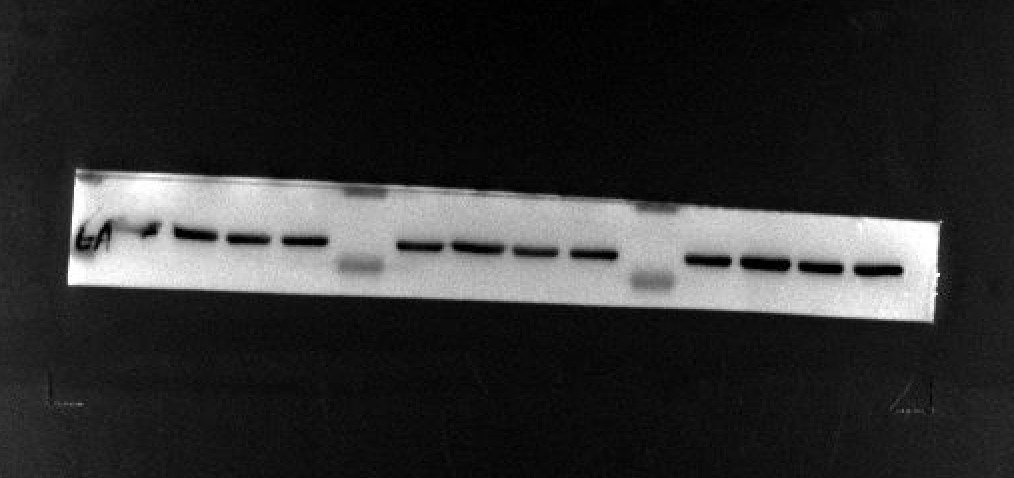

Supplement: Supplementary file 13 [file DataSheet6.ZIP › Original data of Figure 6/Figure 6A-GAPDH-original image-2.jpg]

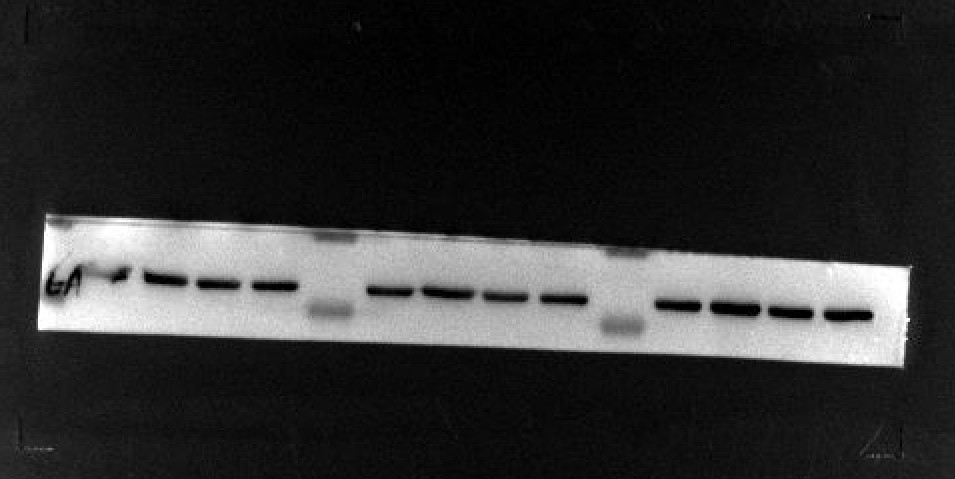

Supplement: Supplementary file 13 [file DataSheet6.ZIP › Original data of Figure 6/Figure 6A-GAPDH-original image-3.jpg]

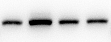

Supplement: Supplementary file 13 [file DataSheet6.ZIP › Original data of Figure 6/Figure 6A-IL-6-1.tif]

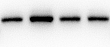

Supplement: Supplementary file 13 [file DataSheet6.ZIP › Original data of Figure 6/Figure 6A-IL-6-2.tif]

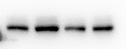

Supplement: Supplementary file 13 [file DataSheet6.ZIP › Original data of Figure 6/Figure 6A-IL-6-3.tif]

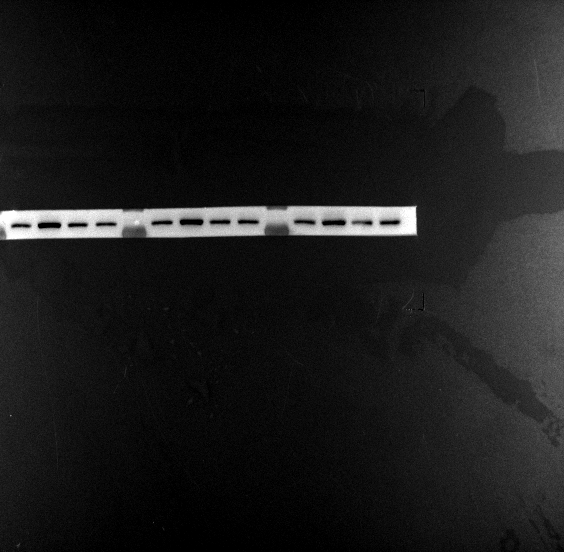

Supplement: Supplementary file 13 [file DataSheet6.ZIP › Original data of Figure 6/Figure 6A-IL-6-original image-1.tif]

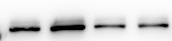

Supplement: Supplementary file 13 [file DataSheet6.ZIP › Original data of Figure 6/Figure 6A-TLR4-1.tif]

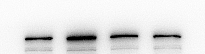

Supplement: Supplementary file 13 [file DataSheet6.ZIP › Original data of Figure 6/Figure 6A-TLR4-2.tif]

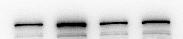

Supplement: Supplementary file 13 [file DataSheet6.ZIP › Original data of Figure 6/Figure 6A-TLR4-3.tif]

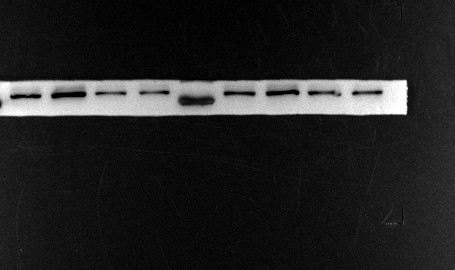

Supplement: Supplementary file 13 [file DataSheet6.ZIP › Original data of Figure 6/Figure 6A-TLR4-original image-1.jpg]

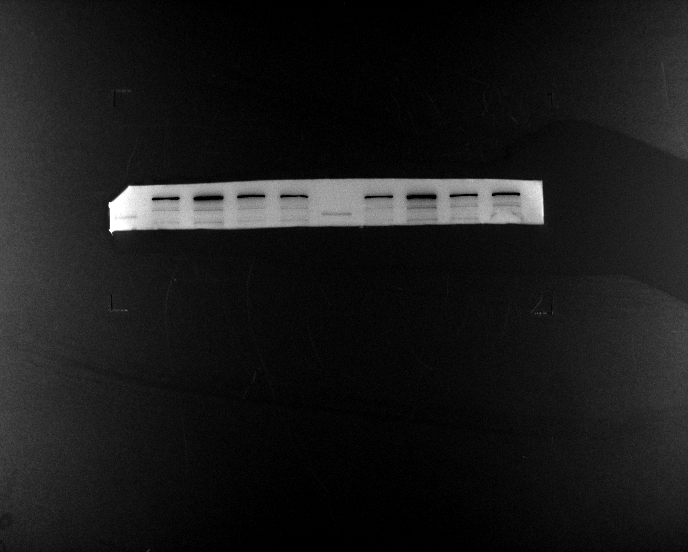

Supplement: Supplementary file 13 [file DataSheet6.ZIP › Original data of Figure 6/Figure 6A-TLR4-original image-2.tif]

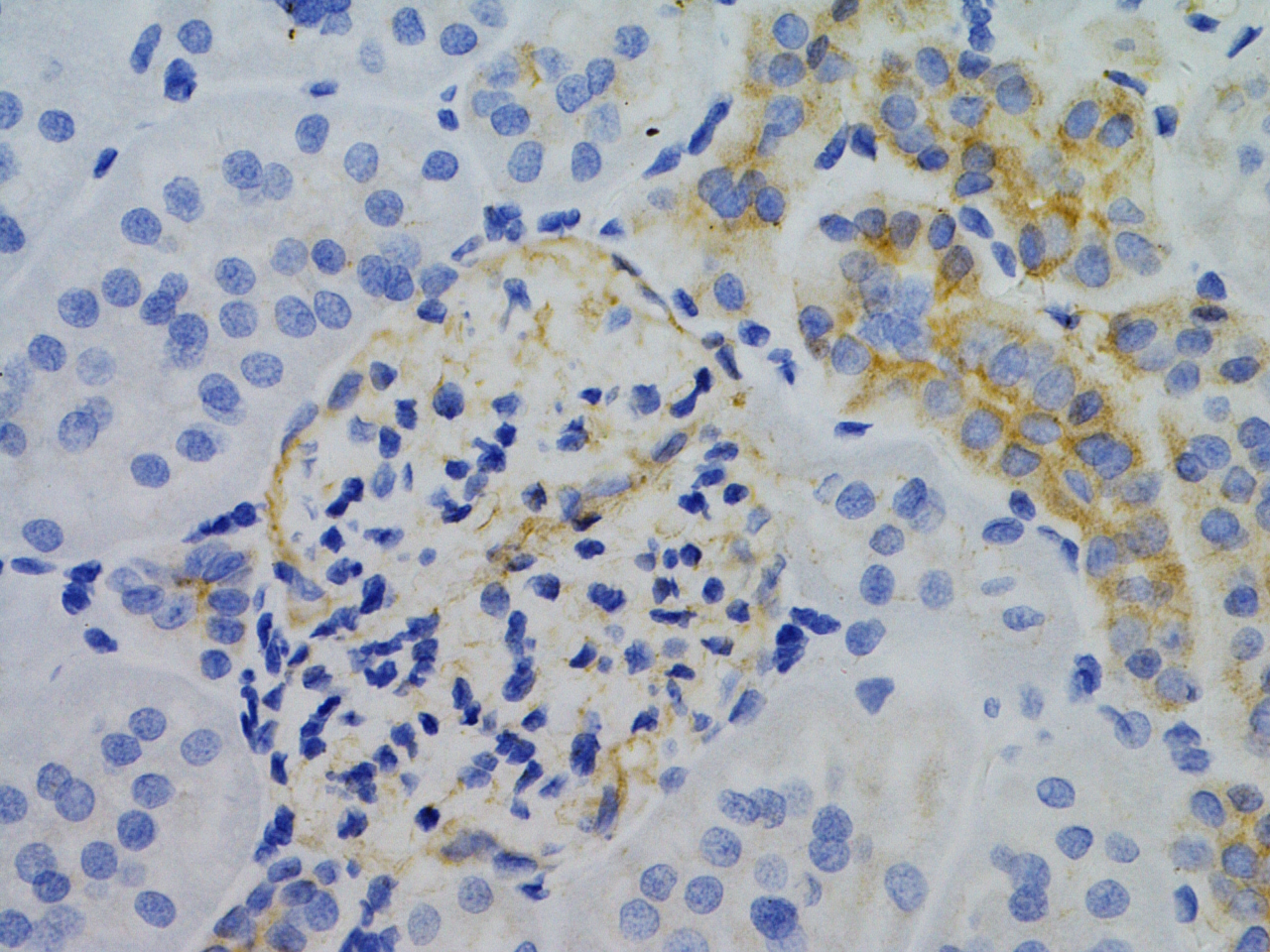

Supplement: Supplementary file 15 [file DataSheet2.ZIP › Original data of Figure 2/Figure 2A-Collagen I (FPS).jpg]

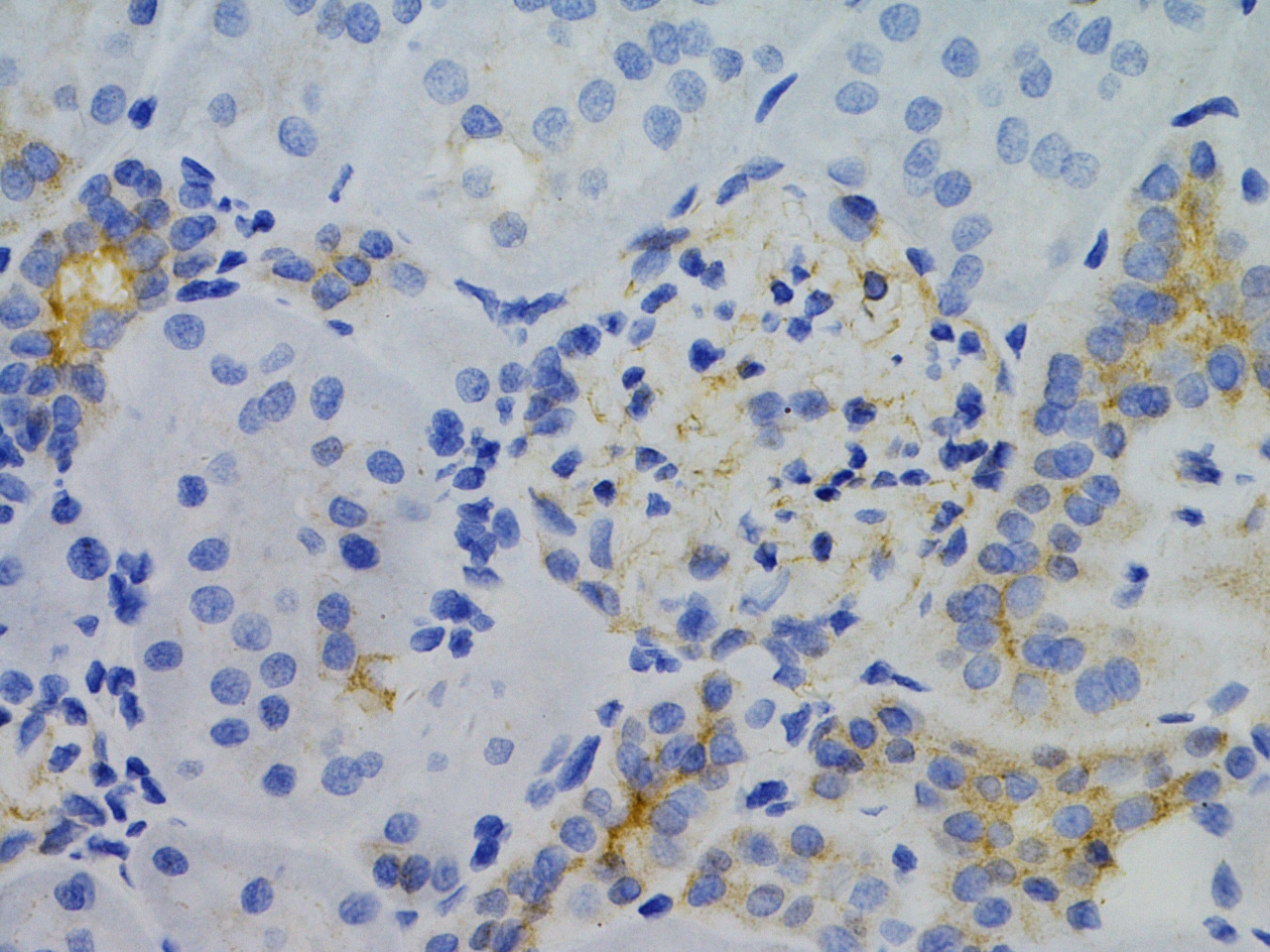

Supplement: Supplementary file 15 [file DataSheet2.ZIP › Original data of Figure 2/Figure 2A-Collagen I (RAP).jpg]

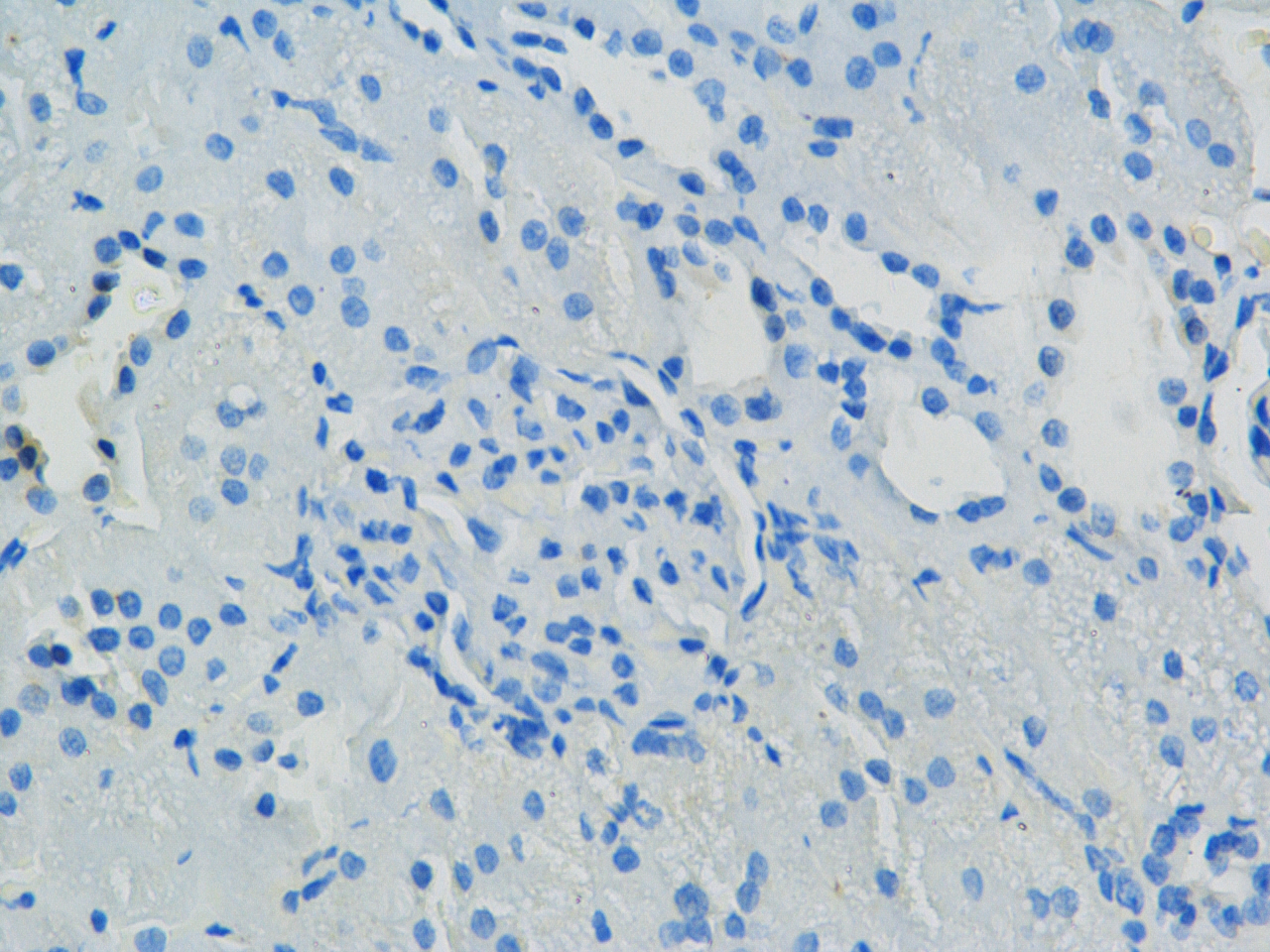

Supplement: Supplementary file 15 [file DataSheet2.ZIP › Original data of Figure 2/Figure 2A-Collagen I (Sham).jpg]

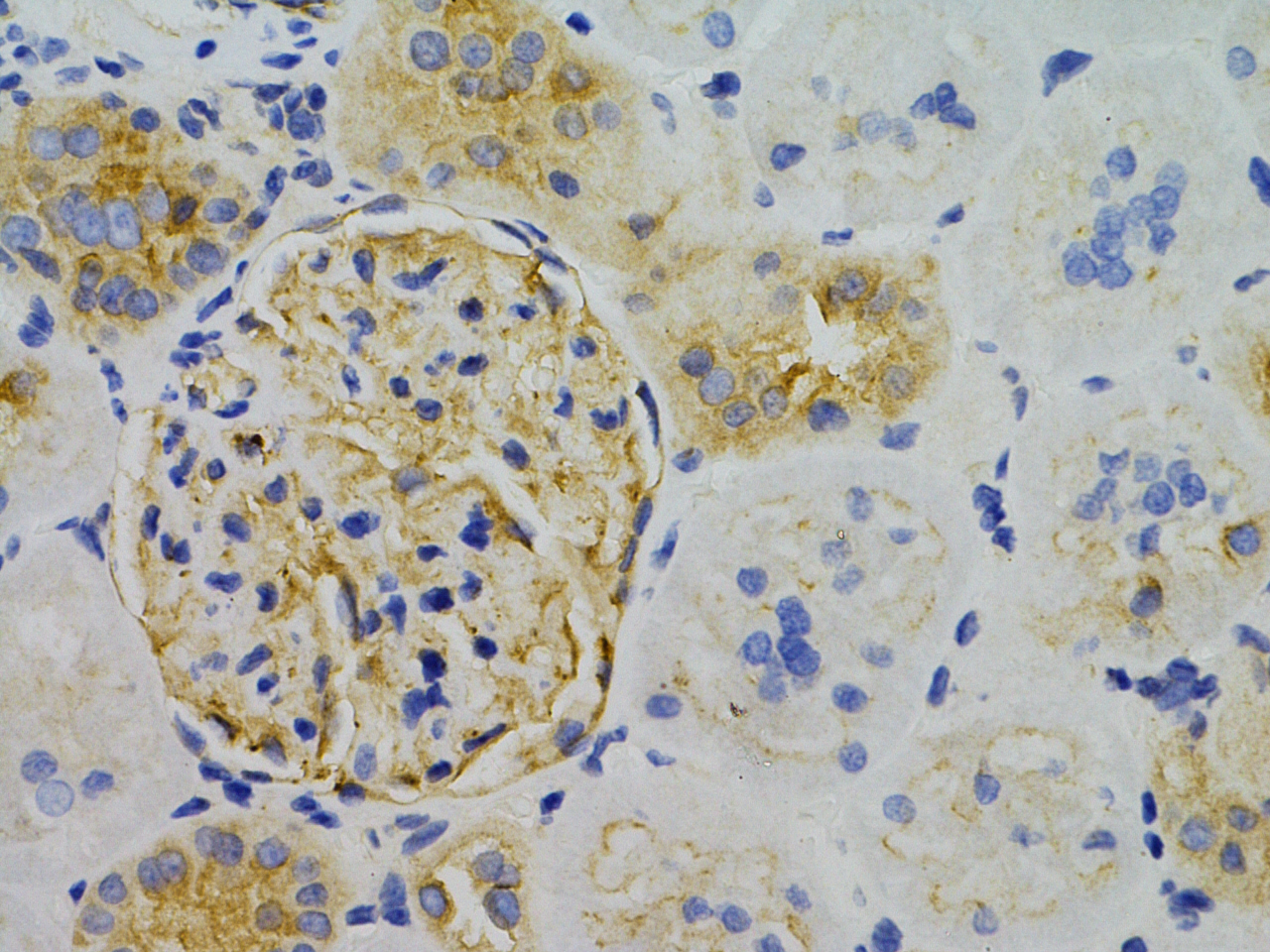

Supplement: Supplementary file 15 [file DataSheet2.ZIP › Original data of Figure 2/Figure 2A-Collagen I (Vehicle).jpg]

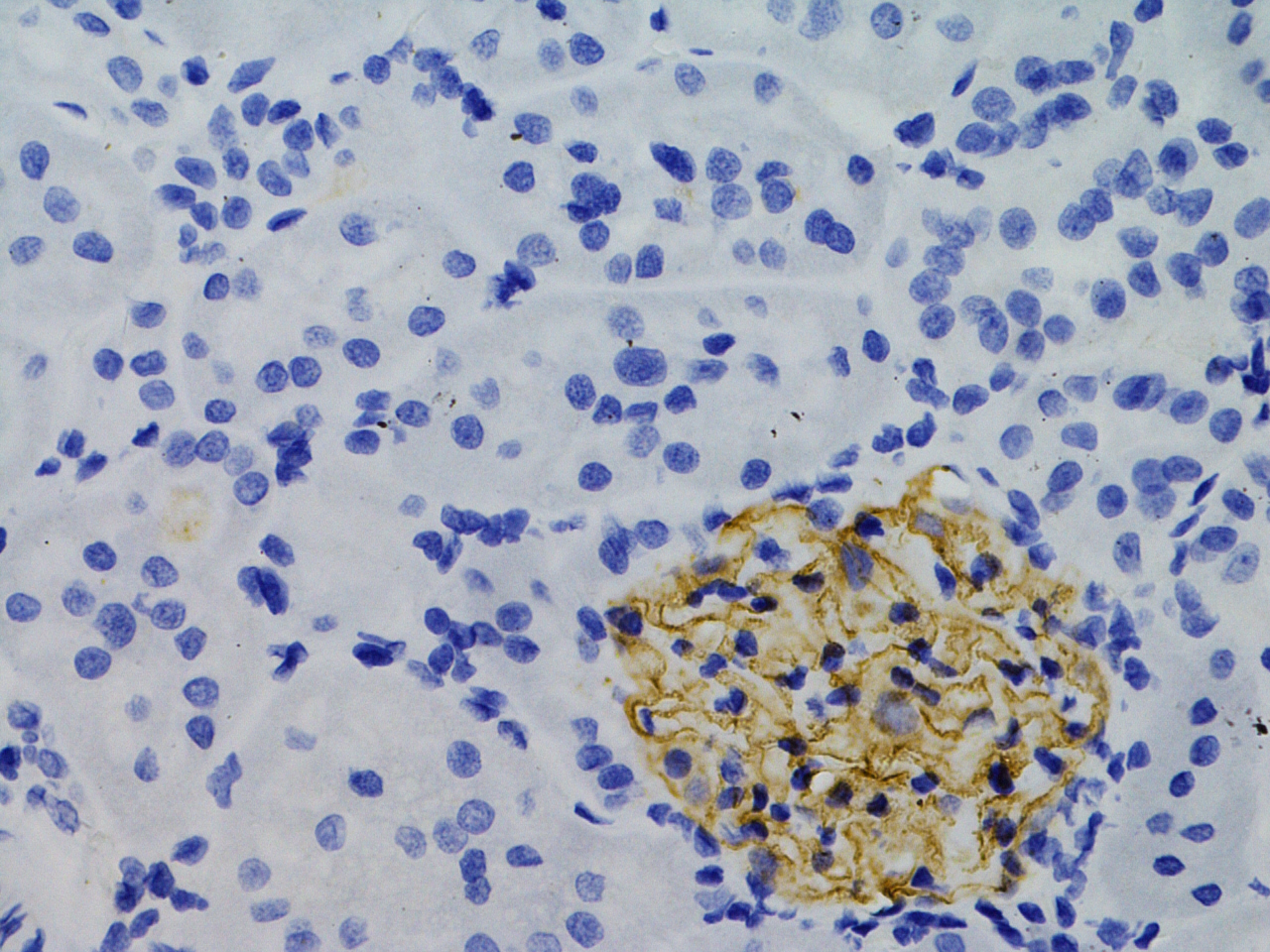

Supplement: Supplementary file 15 [file DataSheet2.ZIP › Original data of Figure 2/Figure 2A-FN (FPS).jpg]

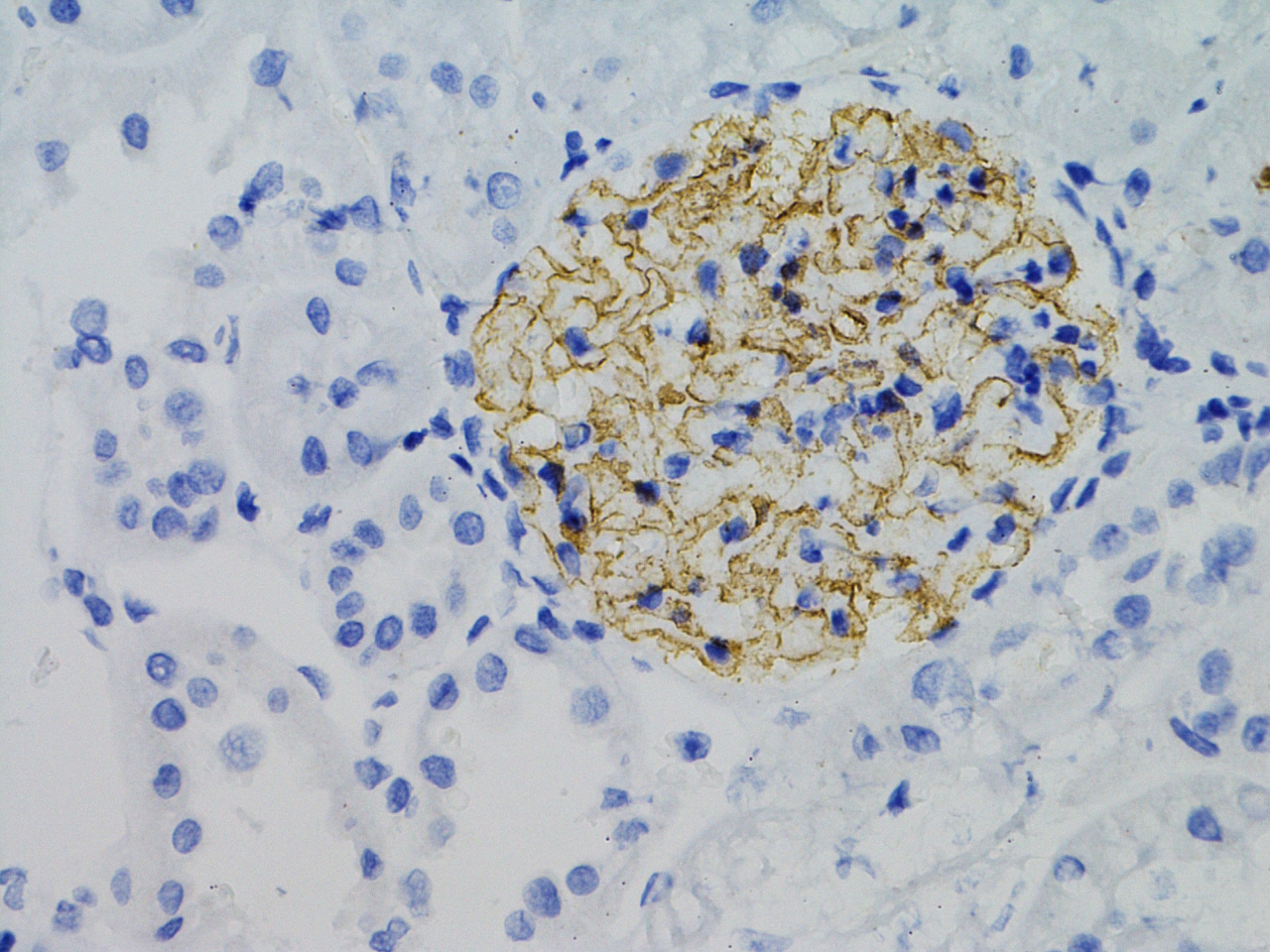

Supplement: Supplementary file 15 [file DataSheet2.ZIP › Original data of Figure 2/Figure 2A-FN (RAP).jpg]

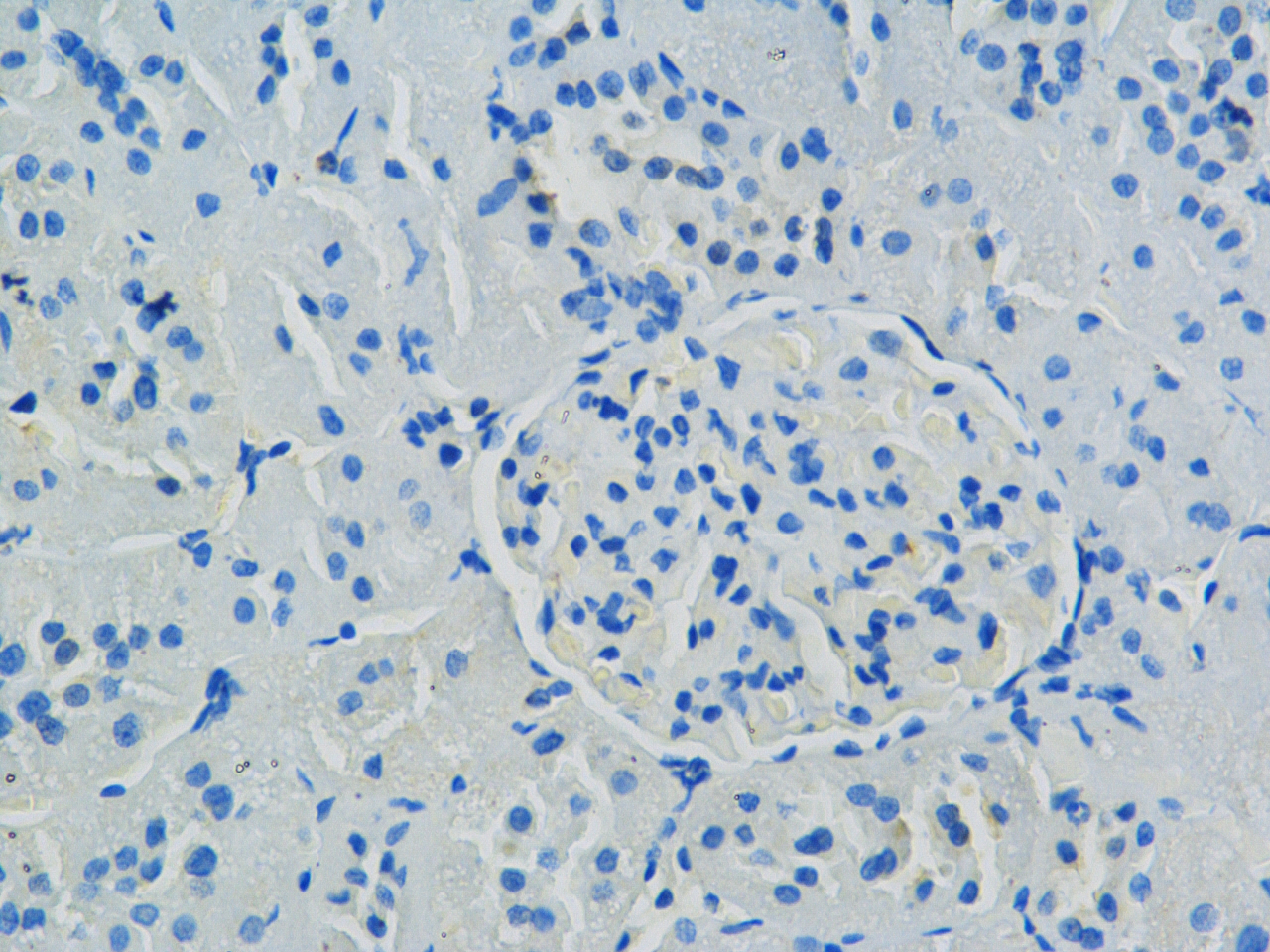

Supplement: Supplementary file 15 [file DataSheet2.ZIP › Original data of Figure 2/Figure 2A-FN (Sham).jpg]

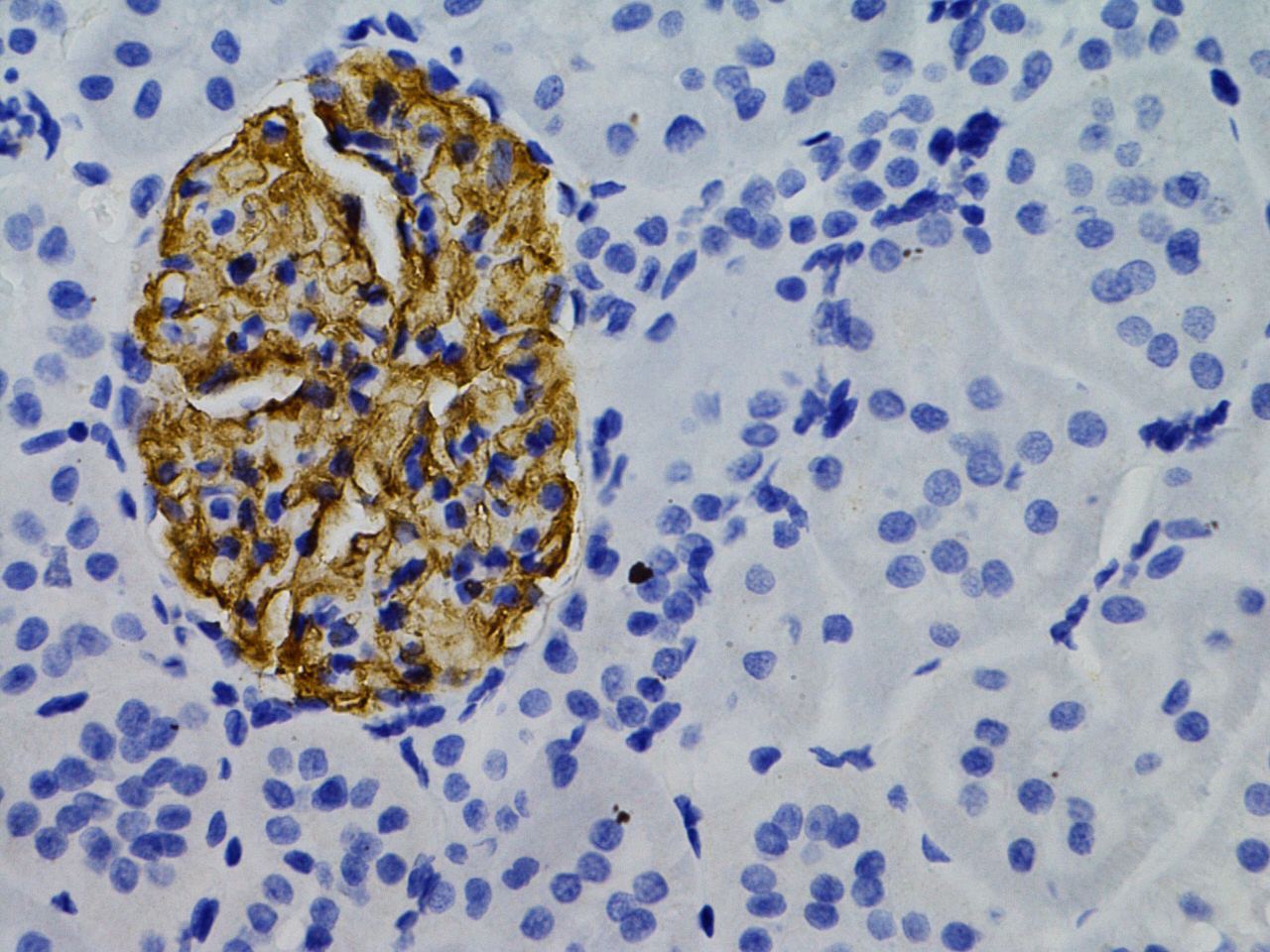

Supplement: Supplementary file 15 [file DataSheet2.ZIP › Original data of Figure 2/Figure 2A-FN (Vehicle).jpg]

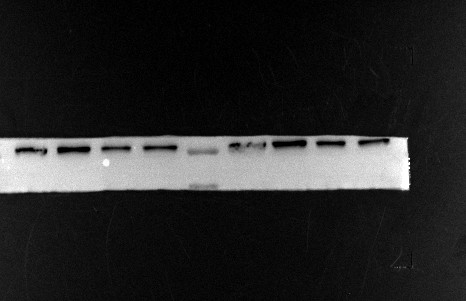

Supplement: Supplementary file 15 [file DataSheet2.ZIP › Original data of Figure 2/Figure 2D-Collagen I--2.jpg]

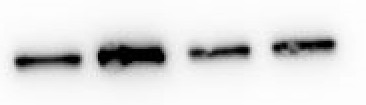

Supplement: Supplementary file 15 [file DataSheet2.ZIP › Original data of Figure 2/Figure 2D-Collagen I-1.jpg]

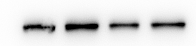

Supplement: Supplementary file 15 [file DataSheet2.ZIP › Original data of Figure 2/Figure 2D-Collagen I-2.tif]

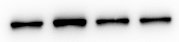

Supplement: Supplementary file 15 [file DataSheet2.ZIP › Original data of Figure 2/Figure 2D-Collagen I-3.tif]

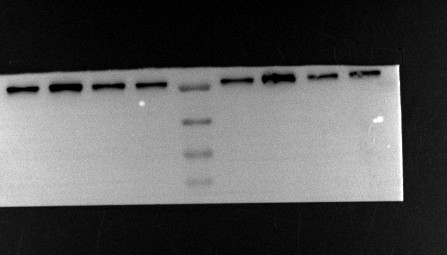

Supplement: Supplementary file 15 [file DataSheet2.ZIP › Original data of Figure 2/Figure 2D-Collagen I-original image-1.jpg]

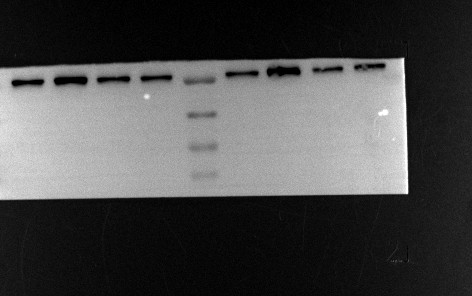

Supplement: Supplementary file 15 [file DataSheet2.ZIP › Original data of Figure 2/Figure 2D-Collagen I-original image-3.jpg]

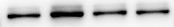

Supplement: Supplementary file 15 [file DataSheet2.ZIP › Original data of Figure 2/Figure 2D-FN-1.tif]

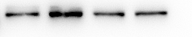

Supplement: Supplementary file 15 [file DataSheet2.ZIP › Original data of Figure 2/Figure 2D-FN-2.tif]

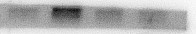

Supplement: Supplementary file 15 [file DataSheet2.ZIP › Original data of Figure 2/Figure 2D-FN-3.jpg]

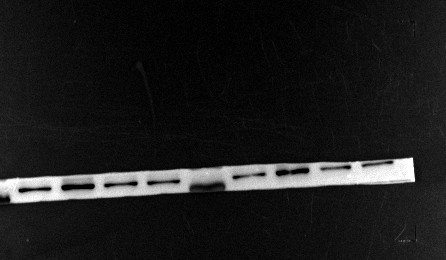

Supplement: Supplementary file 15 [file DataSheet2.ZIP › Original data of Figure 2/Figure 2D-FN-original image-1.jpg]

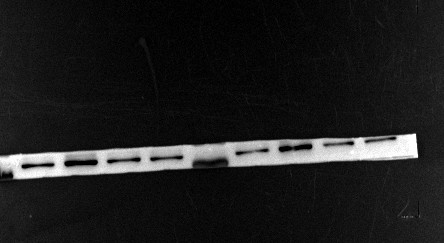

Supplement: Supplementary file 15 [file DataSheet2.ZIP › Original data of Figure 2/Figure 2D-FN-original image-2.jpg]

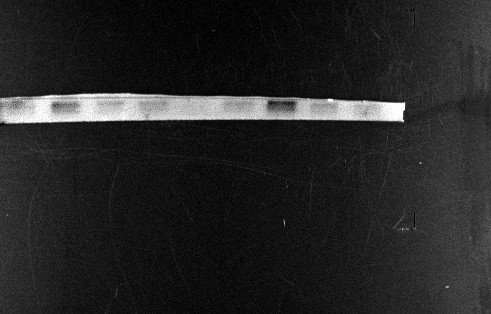

Supplement: Supplementary file 15 [file DataSheet2.ZIP › Original data of Figure 2/Figure 2D-FN-original image-3.jpg]

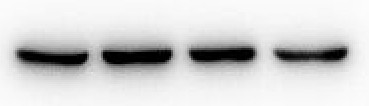

Supplement: Supplementary file 15 [file DataSheet2.ZIP › Original data of Figure 2/Figure 2D-GAPDH-1.jpg]

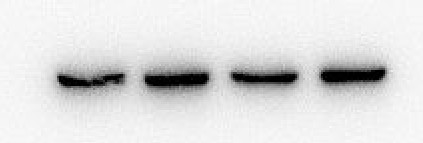

Supplement: Supplementary file 15 [file DataSheet2.ZIP › Original data of Figure 2/Figure 2D-GAPDH-2.jpg]

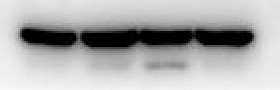

Supplement: Supplementary file 15 [file DataSheet2.ZIP › Original data of Figure 2/Figure 2D-GAPDH-3.jpg]

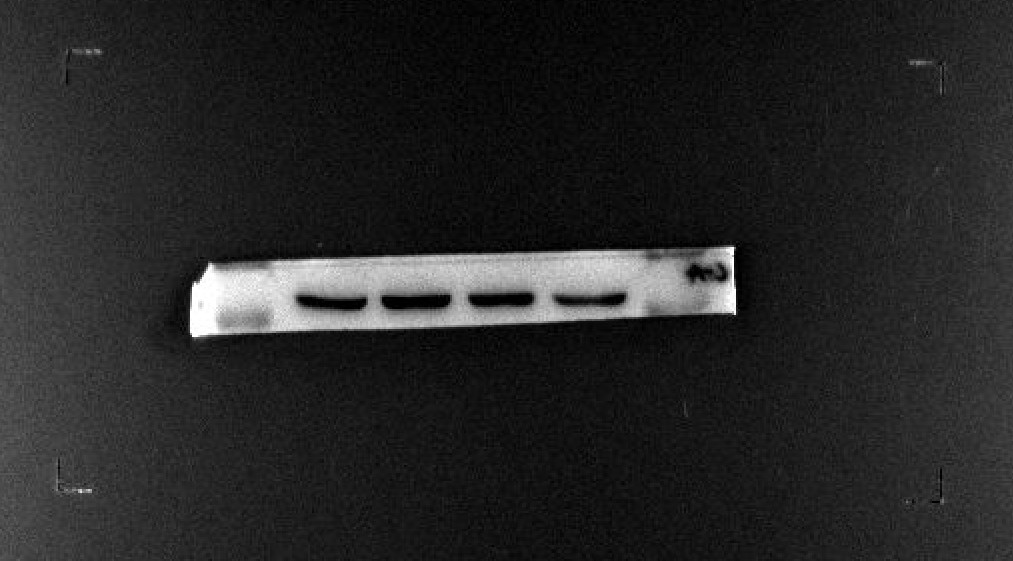

Supplement: Supplementary file 15 [file DataSheet2.ZIP › Original data of Figure 2/Figure 2D-GAPDH-original image-1.jpg]

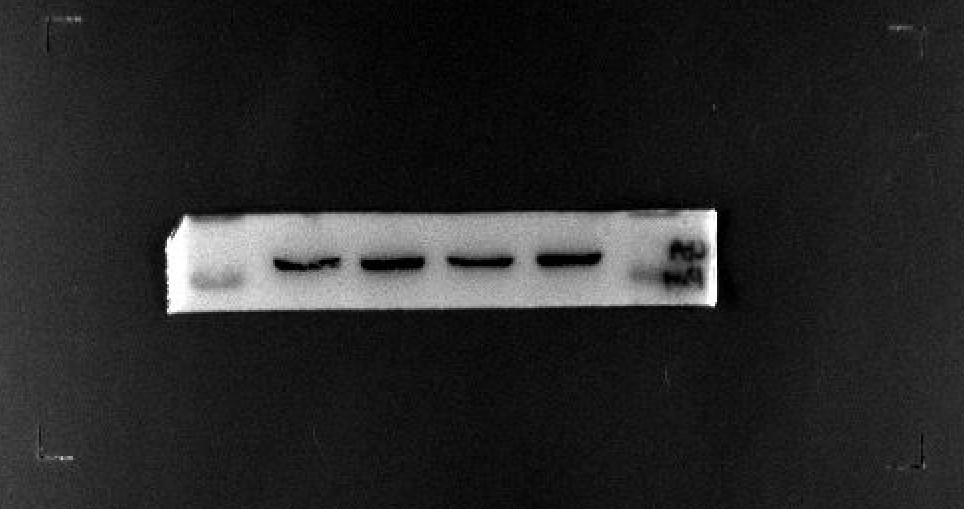

Supplement: Supplementary file 15 [file DataSheet2.ZIP › Original data of Figure 2/Figure 2D-GAPDH-original image-2.jpg]

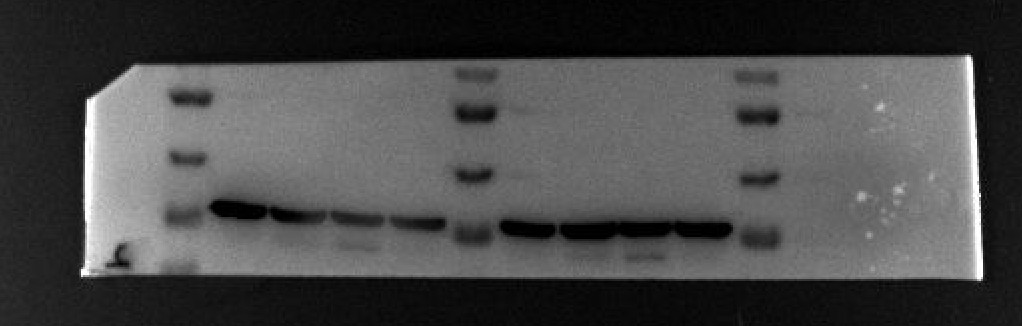

Supplement: Supplementary file 15 [file DataSheet2.ZIP › Original data of Figure 2/Figure 2D-GAPDH-original image-3.jpg]

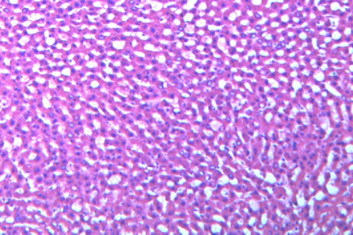

Supplement: Supplementary file 16 [file DataSheet15.ZIP › Original data of Supplementary Figure 2-3 and Figure Legends/Original data of Supplementary Figure 3/Supplementary Figure 3D (FPS).tif]

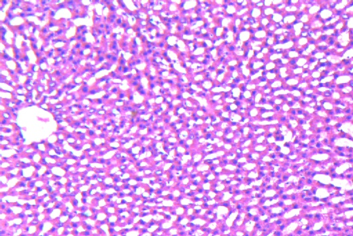

Supplement: Supplementary file 16 [file DataSheet15.ZIP › Original data of Supplementary Figure 2-3 and Figure Legends/Original data of Supplementary Figure 3/Supplementary Figure 3D (Sham).tif]

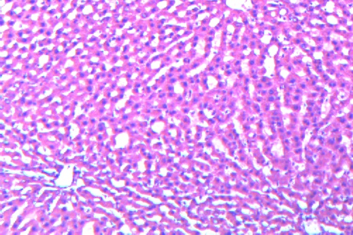

Supplement: Supplementary file 16 [file DataSheet15.ZIP › Original data of Supplementary Figure 2-3 and Figure Legends/Original data of Supplementary Figure 3/Supplementary Figure 3D (Vehicle).tif]

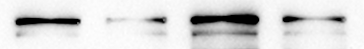

Supplement: Supplementary file 17 [file DataSheet5.ZIP › Original data of Figure 5/Figure 5A-CD2AP-1.tif]

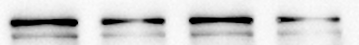

Supplement: Supplementary file 17 [file DataSheet5.ZIP › Original data of Figure 5/Figure 5A-CD2AP-2.tif]
