# Supplementary material for: Fucoidan Alleviates Renal Fibrosis in Diabetic Kidney Disease via Inhibition of NLRP3 Inflammasome-Mediated Podocyte Pyroptosis
Source: Front Pharmacol. 2022 Mar 18;13:790937. doi: 10.3389/fphar.2022.790937 (PMC8972405; doi:10.3389/fphar.2022.790937)

IL-18

Original image 1,2

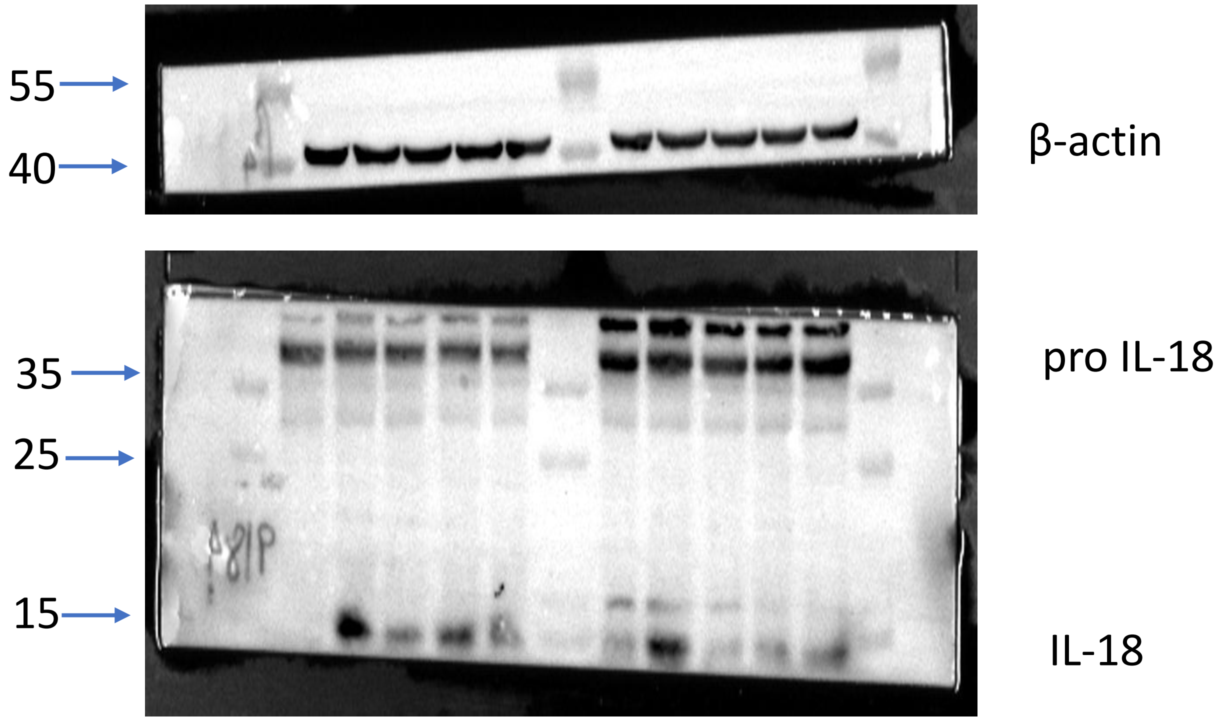

Original image 3

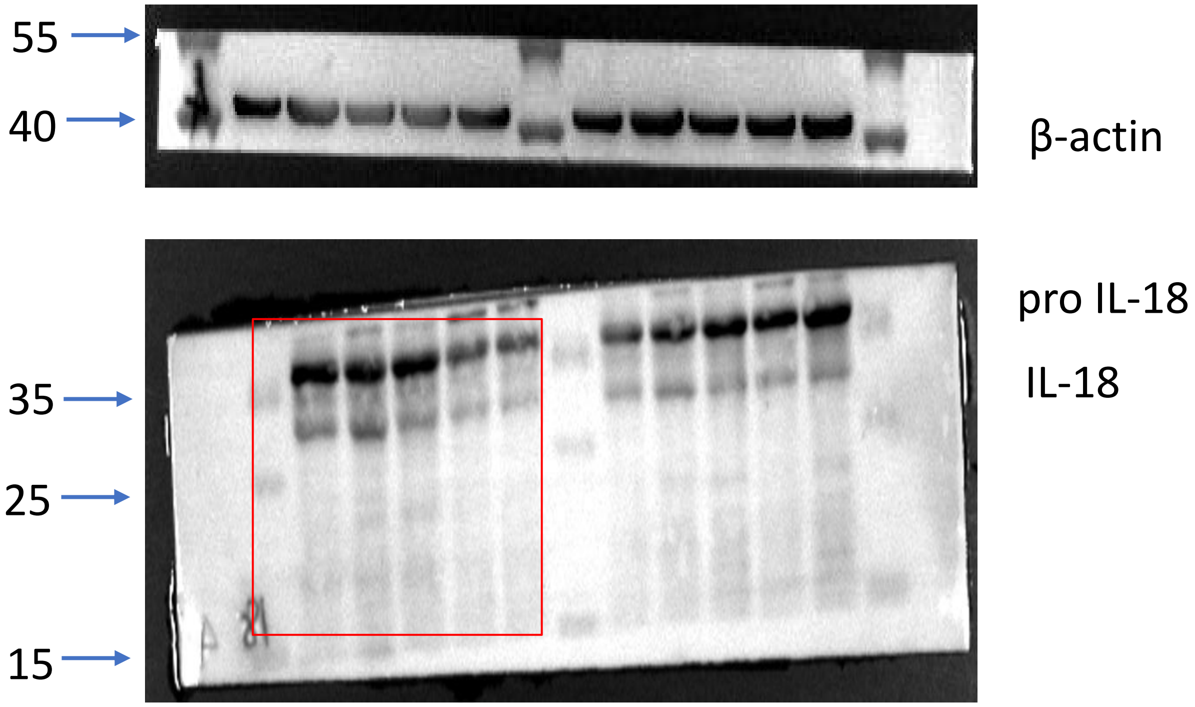

IL-1β

Original image 1

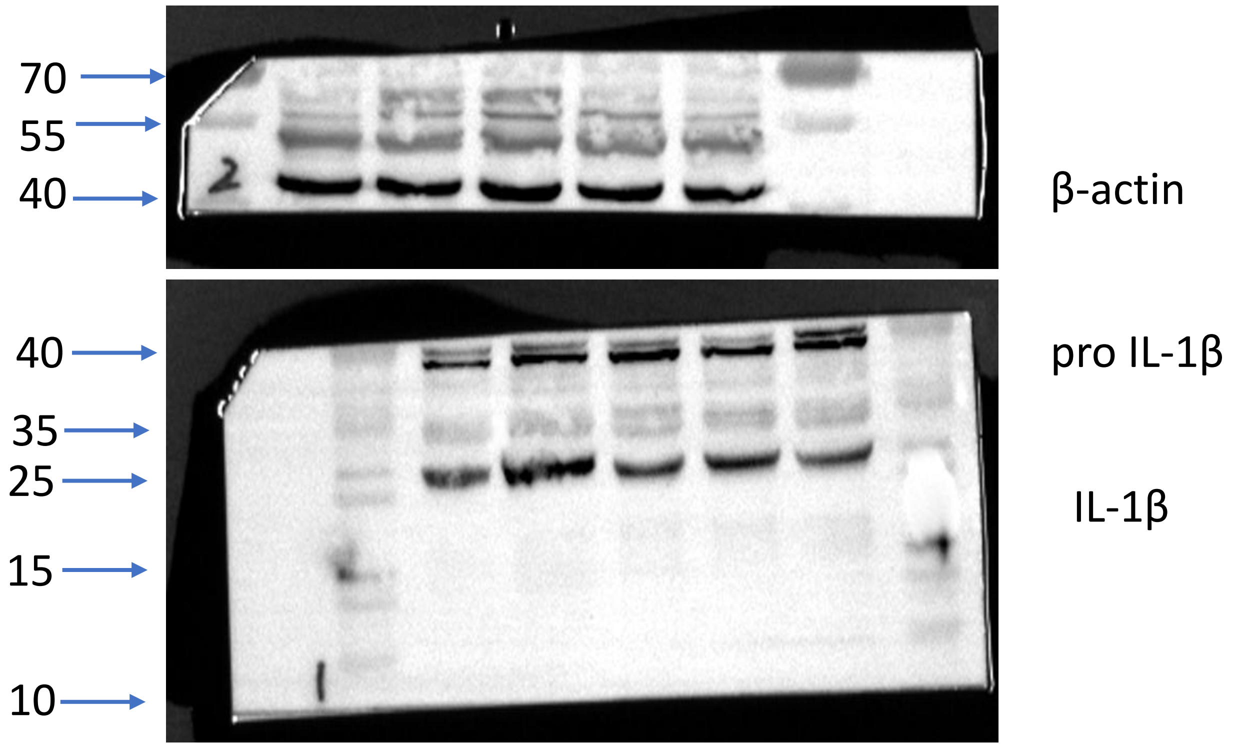

Original image 2

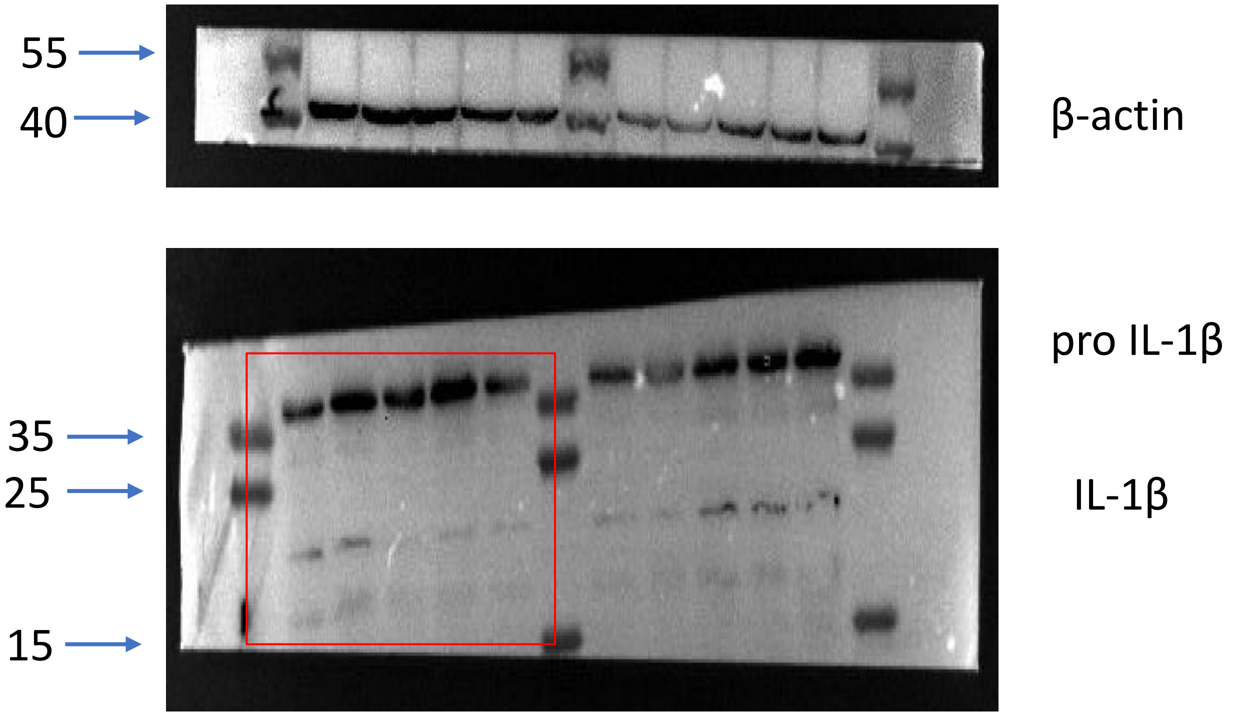

Original image 3

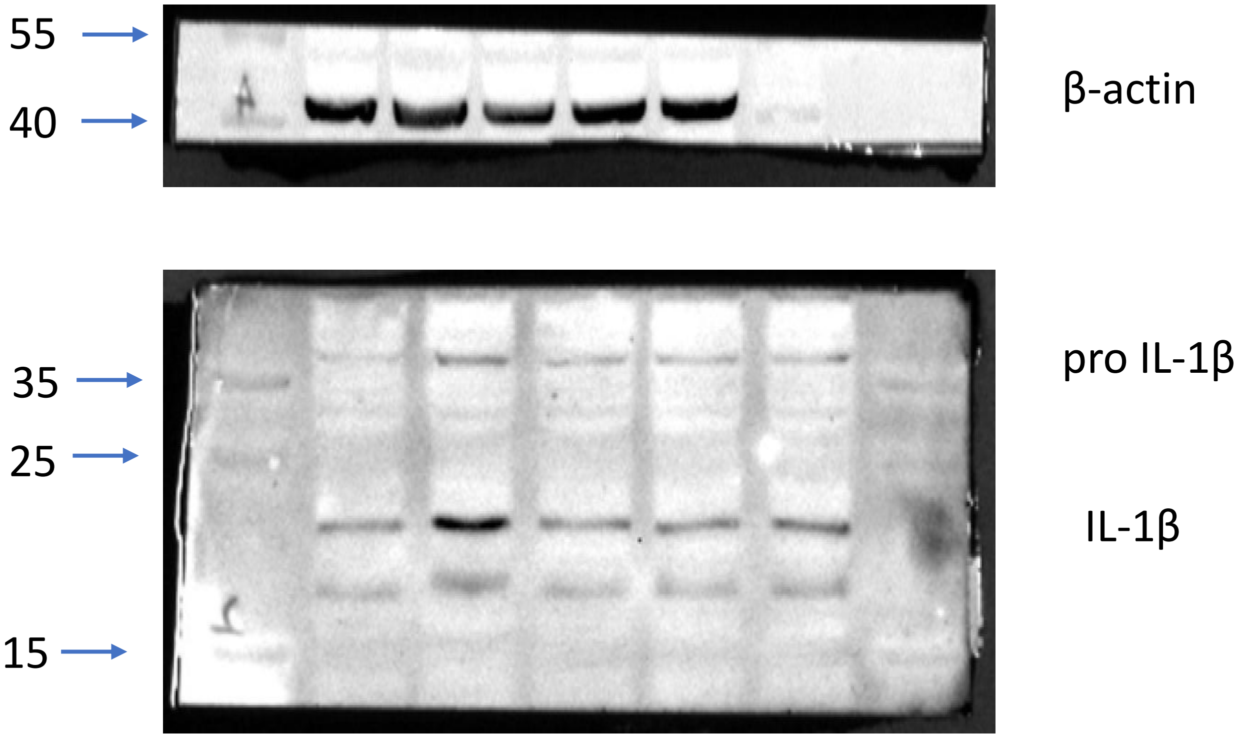

Supplement: Supplementary file 14 [file DataSheet12.ZIP › Original data of Figure 12/Figure 12A-pro-IL-18, IL-18 and pro-IL-1β, IL-1β-original image-1-3.pdf]
