# Supplementary material for: Fucoidan Alleviates Renal Fibrosis in Diabetic Kidney Disease via Inhibition of NLRP3 Inflammasome-Mediated Podocyte Pyroptosis
Source: Front Pharmacol. 2022 Mar 18;13:790937. doi: 10.3389/fphar.2022.790937 (PMC8972405; doi:10.3389/fphar.2022.790937)
Supplement: Supplementary file 16 [file DataSheet15.ZIP › Original data of Supplementary Figure 2-3 and Figure Legends/Revised 790937 (edition 3) Supplementary Figure Legends.docx]

Supplementary Material

## Supplementary Figure Legends

**Supplementary Figure 1.** Animal experimental protocols.

**Supplementary Figure 2.** Viability of the cultured MPC-5 cells. (A) The cultured MPC-5 cells were exposed to HG (30 mmol/L) with FPS at 15, 20, and 25 μg/ml for 24 h. (B) The cultured MPC-5 cells were exposed to HG (30 mmol/L) with RAP at 15, 20, and 25 nmol/L for 24 h.

**Supplementary Figure 3.** Effects of FPS and RAP on ALT, AST, and histological characteristics of liver tissues in the DKD model rats. (A) ALT level. (B) AST level. (D) H&E staining of the liver tissues (× 400). Scale bar = 50 μm.
