# Supplementary figures and images for: Fucoidan Alleviates Renal Fibrosis in Diabetic Kidney Disease via Inhibition of NLRP3 Inflammasome-Mediated Podocyte Pyroptosis (part 3 of 3)
Source: Front Pharmacol. 2022 Mar 18;13:790937. doi: 10.3389/fphar.2022.790937 (PMC8972405; doi:10.3389/fphar.2022.790937)

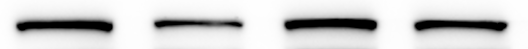

Supplement: Supplementary file 17 [file DataSheet5.ZIP › Original data of Figure 5/Figure 5A-CD2AP-3.tif]

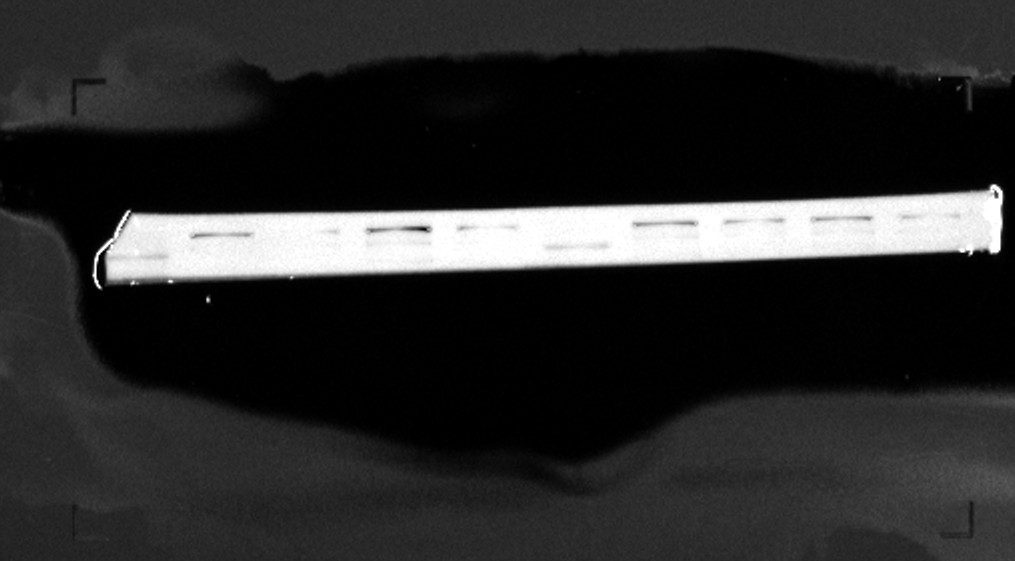

Supplement: Supplementary file 17 [file DataSheet5.ZIP › Original data of Figure 5/Figure 5A-CD2AP-original image-1.jpg]

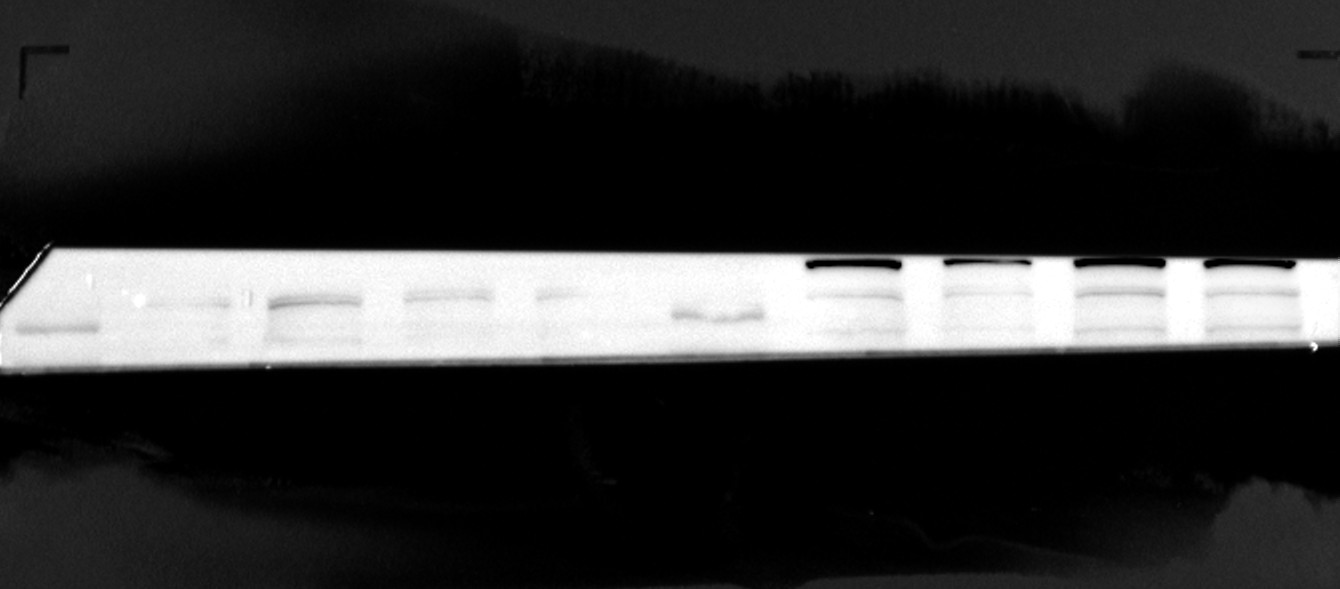

Supplement: Supplementary file 17 [file DataSheet5.ZIP › Original data of Figure 5/Figure 5A-CD2AP-original image-3.jpg]

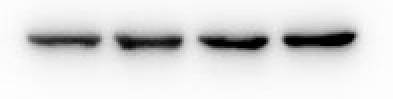

Supplement: Supplementary file 17 [file DataSheet5.ZIP › Original data of Figure 5/Figure 5A-GAPDH-1.jpg]

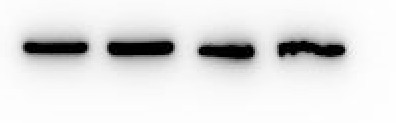

Supplement: Supplementary file 17 [file DataSheet5.ZIP › Original data of Figure 5/Figure 5A-GAPDH-2.jpg]

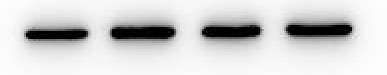

Supplement: Supplementary file 17 [file DataSheet5.ZIP › Original data of Figure 5/Figure 5A-GAPDH-3.jpg]

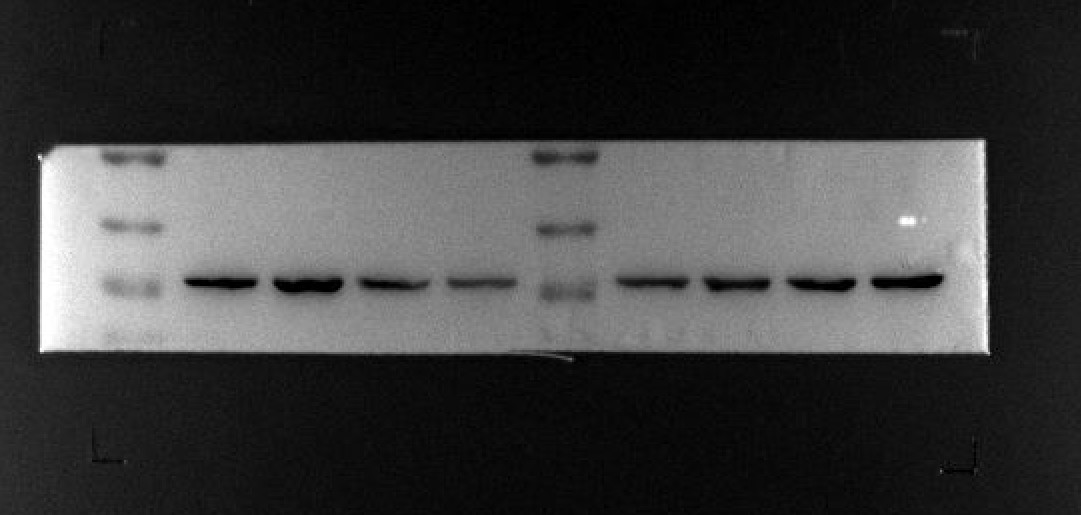

Supplement: Supplementary file 17 [file DataSheet5.ZIP › Original data of Figure 5/Figure 5A-GAPDH-original image-1.jpg]

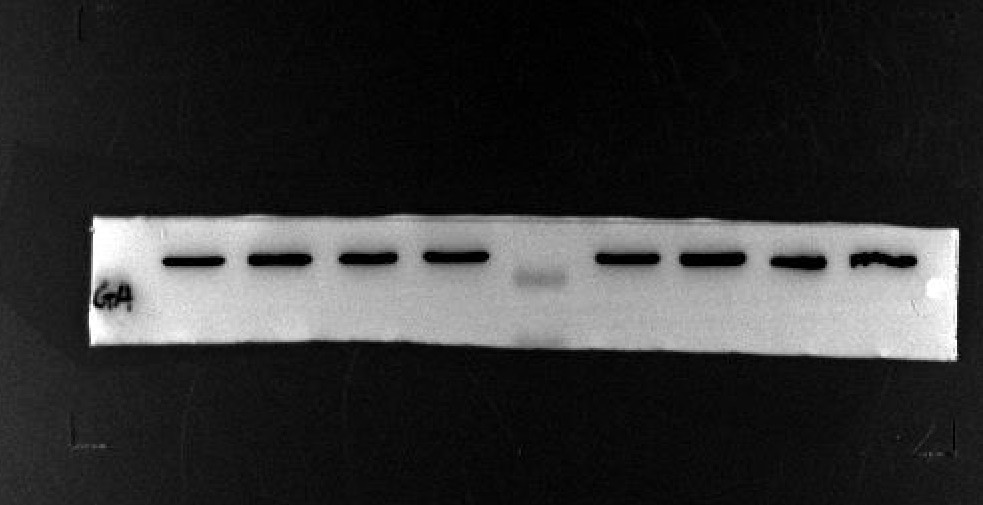

Supplement: Supplementary file 17 [file DataSheet5.ZIP › Original data of Figure 5/Figure 5A-GAPDH-original image-2.jpg]

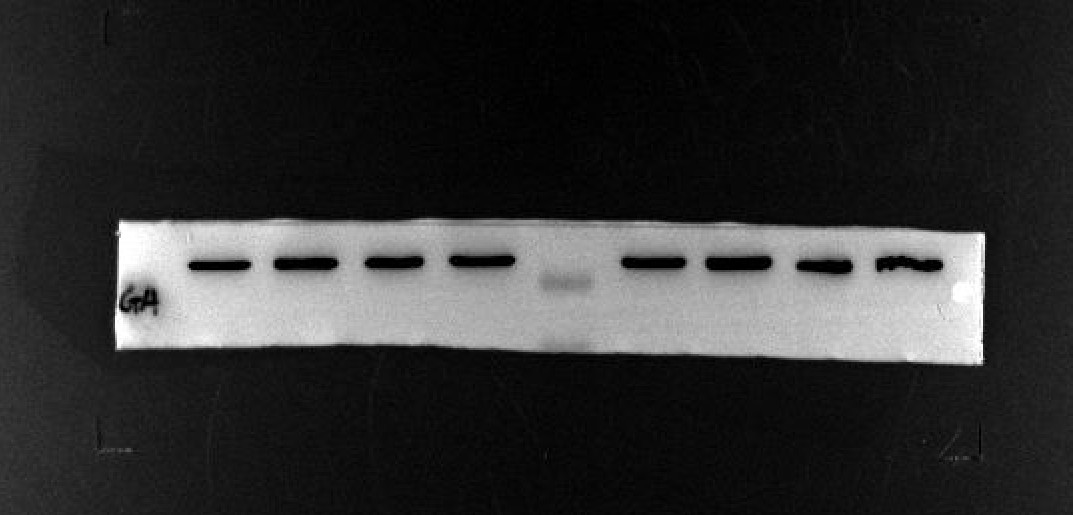

Supplement: Supplementary file 17 [file DataSheet5.ZIP › Original data of Figure 5/Figure 5A-GAPDH-original image-3.jpg]

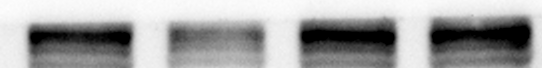

Supplement: Supplementary file 17 [file DataSheet5.ZIP › Original data of Figure 5/Figure 5A-neph1-1.tif]

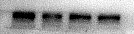

Supplement: Supplementary file 17 [file DataSheet5.ZIP › Original data of Figure 5/Figure 5A-neph1-2.tif]

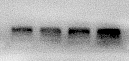

Supplement: Supplementary file 17 [file DataSheet5.ZIP › Original data of Figure 5/Figure 5A-neph1-3.tif]

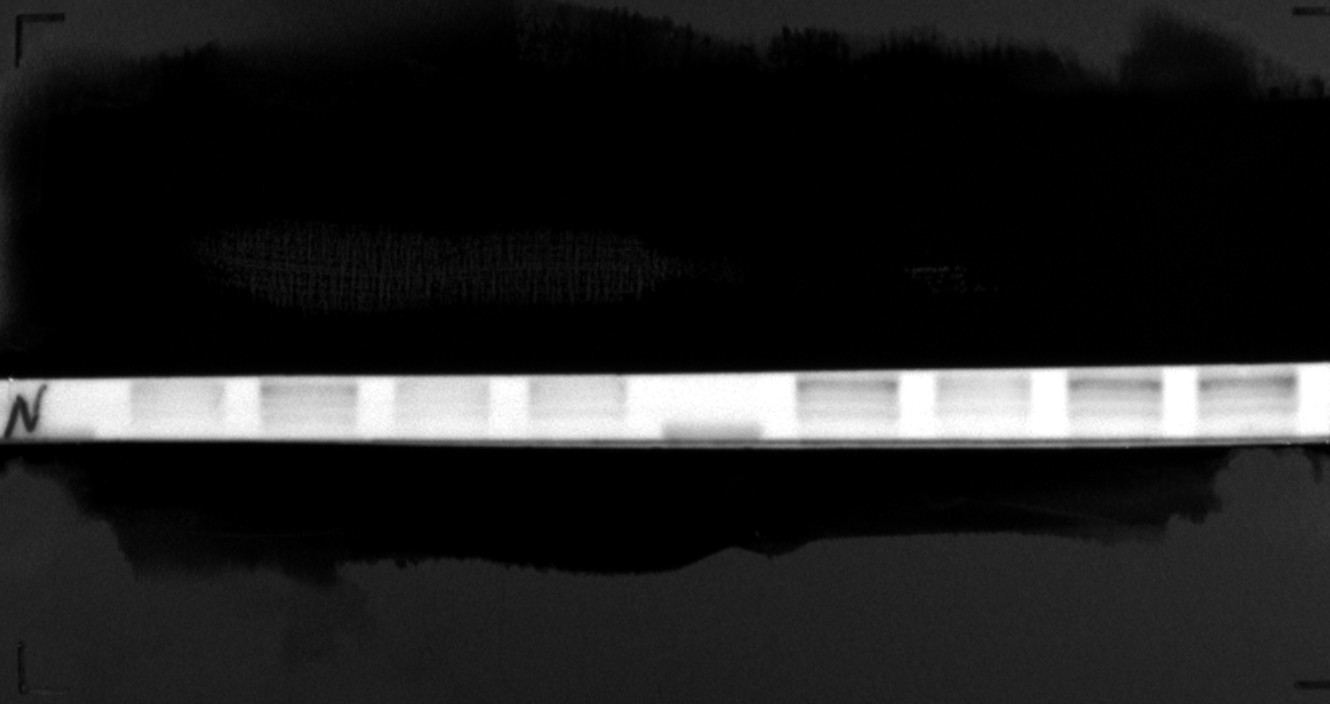

Supplement: Supplementary file 17 [file DataSheet5.ZIP › Original data of Figure 5/Figure 5A-neph1-original image-1.jpg]

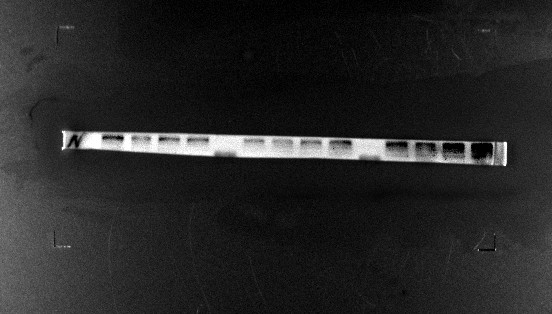

Supplement: Supplementary file 17 [file DataSheet5.ZIP › Original data of Figure 5/Figure 5A-neph1-original image-2.jpg]

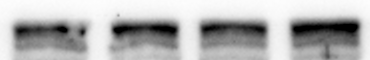

Supplement: Supplementary file 17 [file DataSheet5.ZIP › Original data of Figure 5/Figure 5A-nephrin-1.tif]

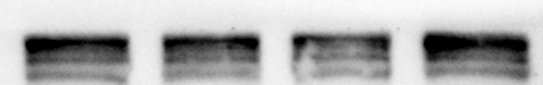

Supplement: Supplementary file 17 [file DataSheet5.ZIP › Original data of Figure 5/Figure 5A-nephrin-2.tif]

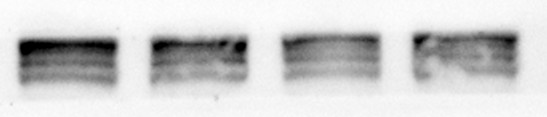

Supplement: Supplementary file 17 [file DataSheet5.ZIP › Original data of Figure 5/Figure 5A-nephrin-3.jpg]

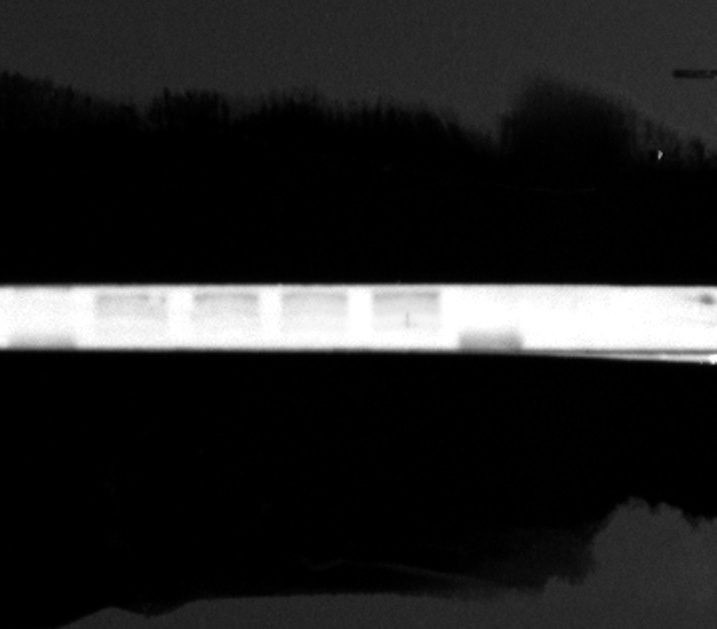

Supplement: Supplementary file 17 [file DataSheet5.ZIP › Original data of Figure 5/Figure 5A-nephrin-origianl image-1.jpg]

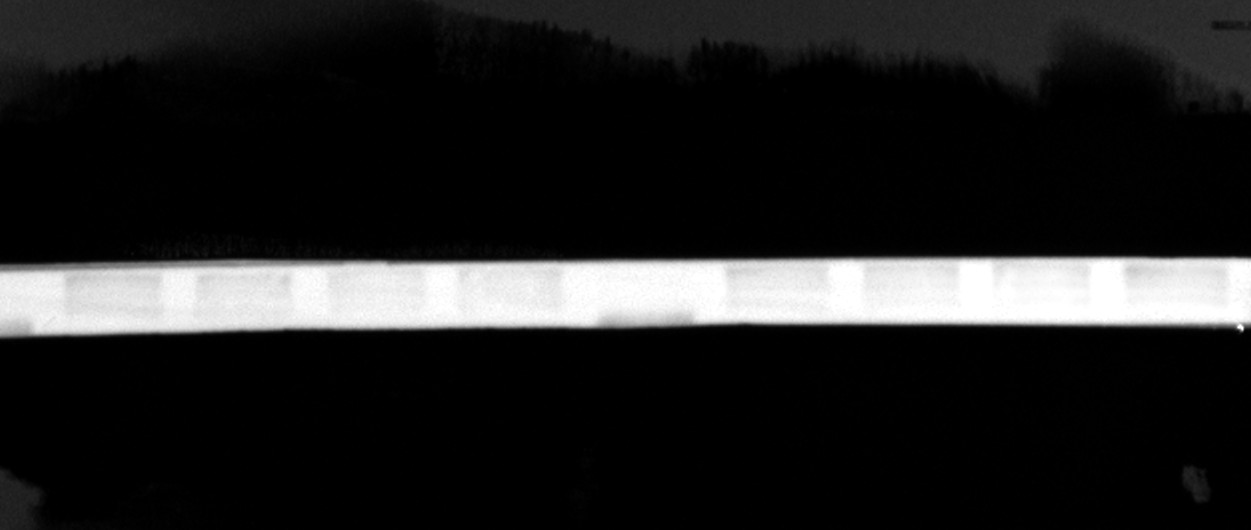

Supplement: Supplementary file 17 [file DataSheet5.ZIP › Original data of Figure 5/Figure 5A-nephrin-origianl image-2.jpg]

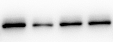

Supplement: Supplementary file 17 [file DataSheet5.ZIP › Original data of Figure 5/Figure 5A-podocin-1.tif]

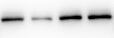

Supplement: Supplementary file 17 [file DataSheet5.ZIP › Original data of Figure 5/Figure 5A-podocin-2.tif]

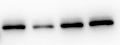

Supplement: Supplementary file 17 [file DataSheet5.ZIP › Original data of Figure 5/Figure 5A-podocin-3.tif]

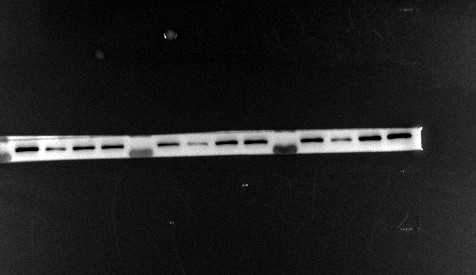

Supplement: Supplementary file 17 [file DataSheet5.ZIP › Original data of Figure 5/Figure 5A-podocin-original image-1.jpg]

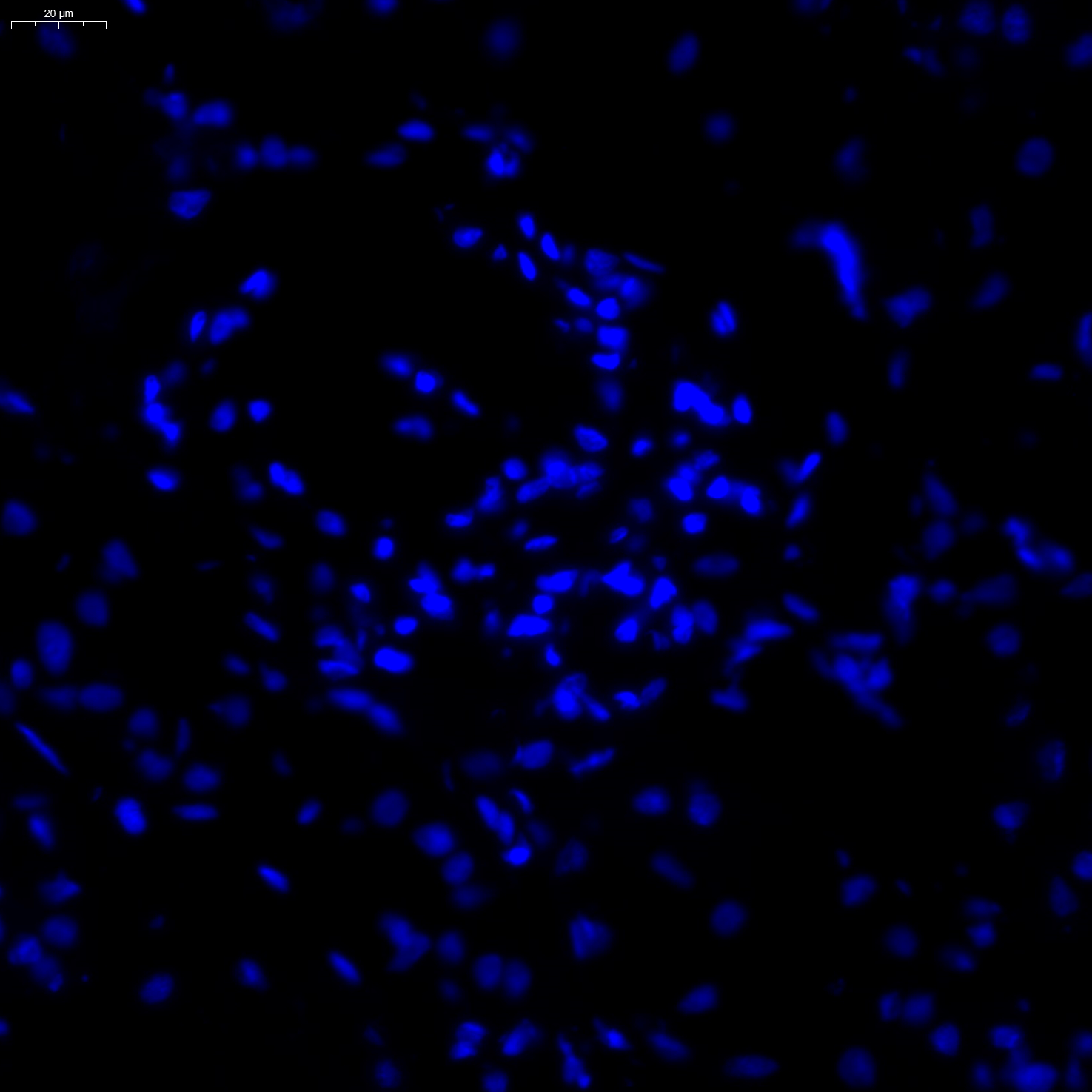

Supplement: Supplementary file 18 [file DataSheet7.ZIP › Original data of Figure 7/Figure 7A-DAPI (Sham).tif]

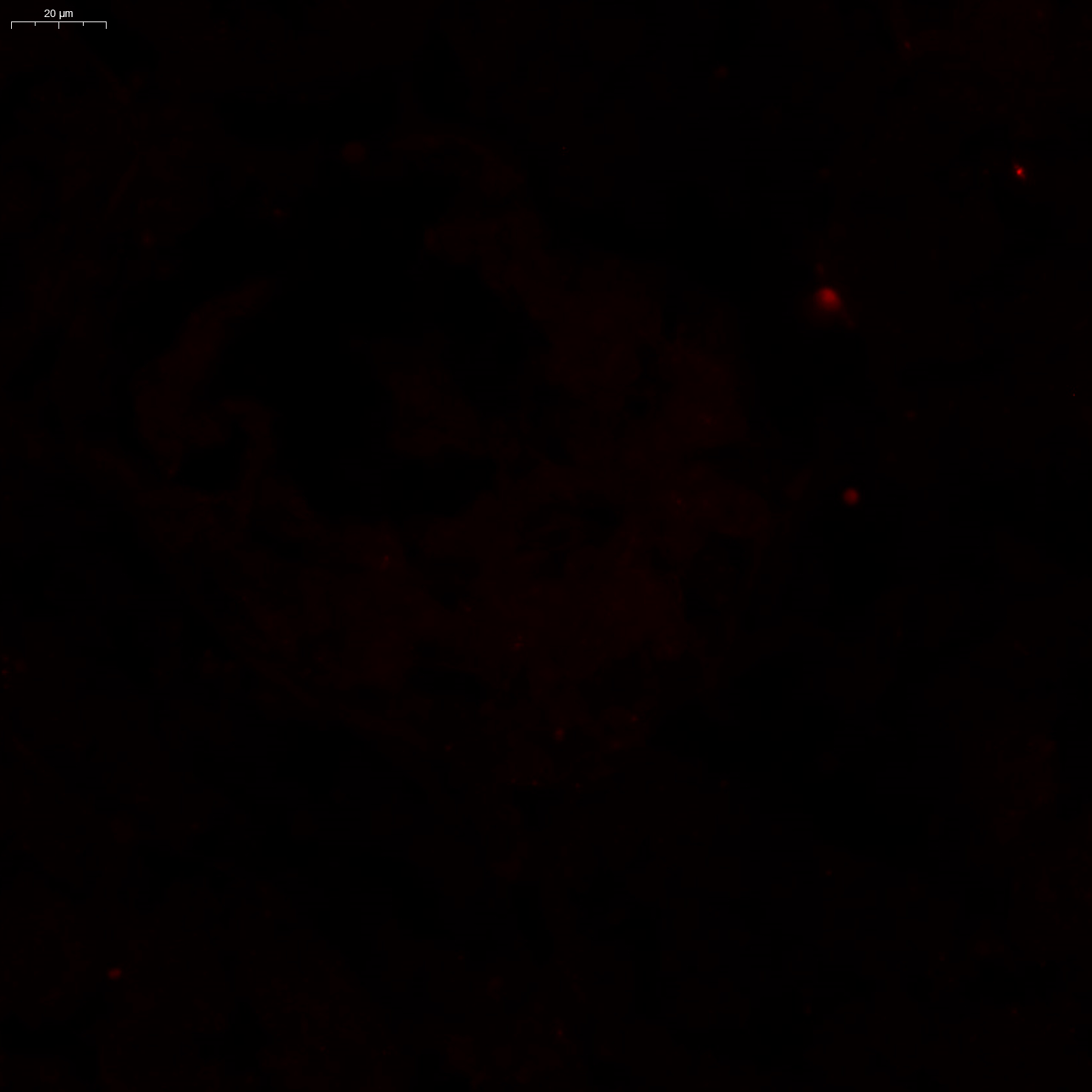

Supplement: Supplementary file 18 [file DataSheet7.ZIP › Original data of Figure 7/Figure 7A-GSDMD-N (Sham).tif]

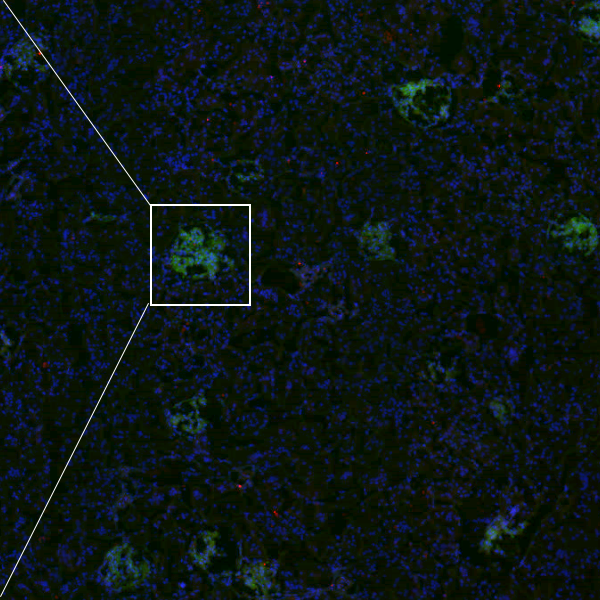

Supplement: Supplementary file 18 [file DataSheet7.ZIP › Original data of Figure 7/Figure 7A-Merge (FPS)× 100.tif]

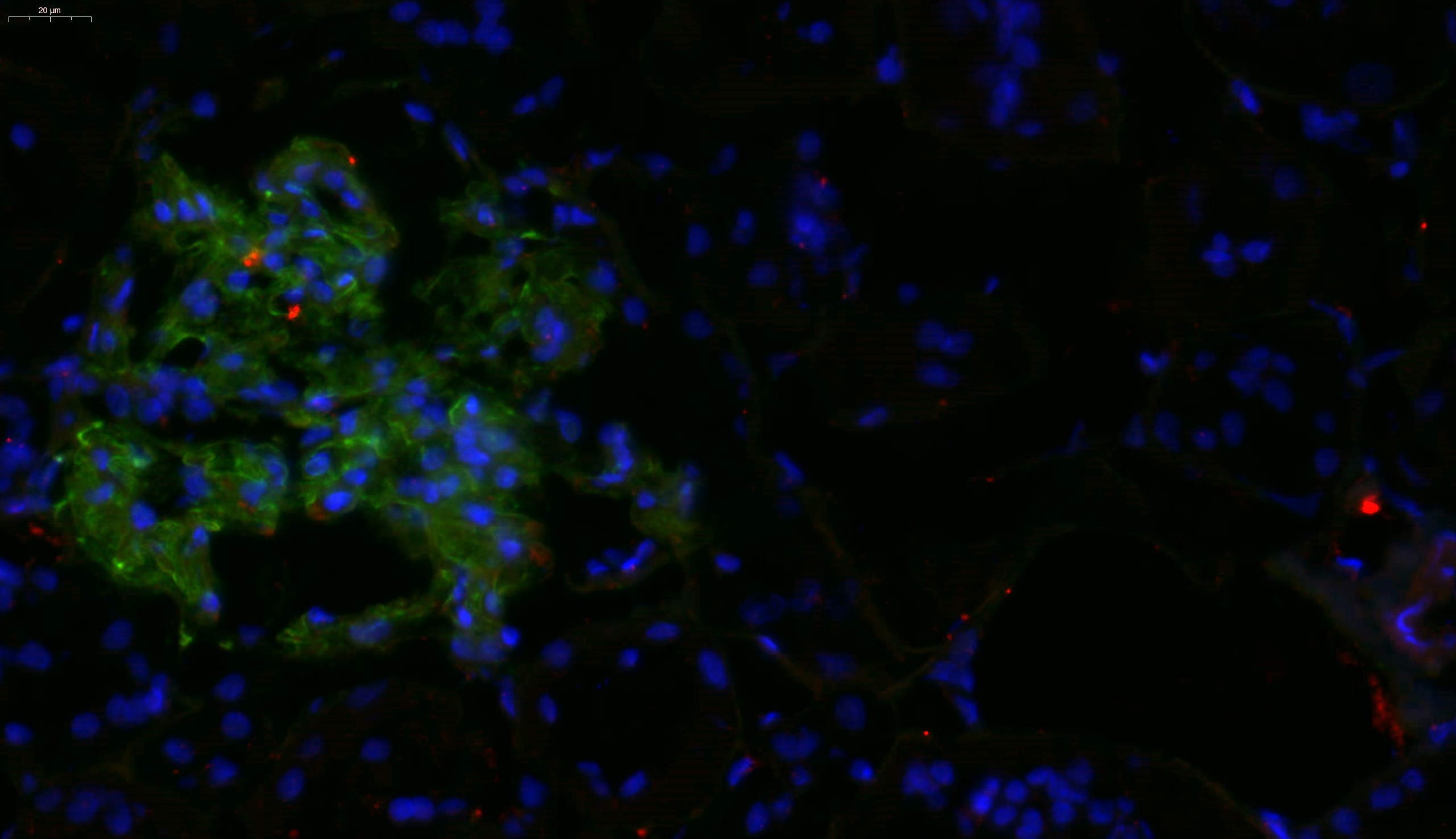

Supplement: Supplementary file 18 [file DataSheet7.ZIP › Original data of Figure 7/Figure 7A-Merge (FPS)× 400.tif]

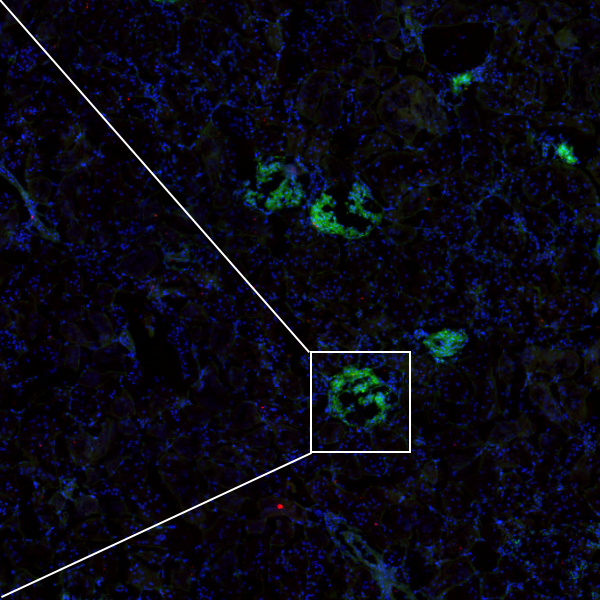

Supplement: Supplementary file 18 [file DataSheet7.ZIP › Original data of Figure 7/Figure 7A-Merge (RAP)× 100.tif]

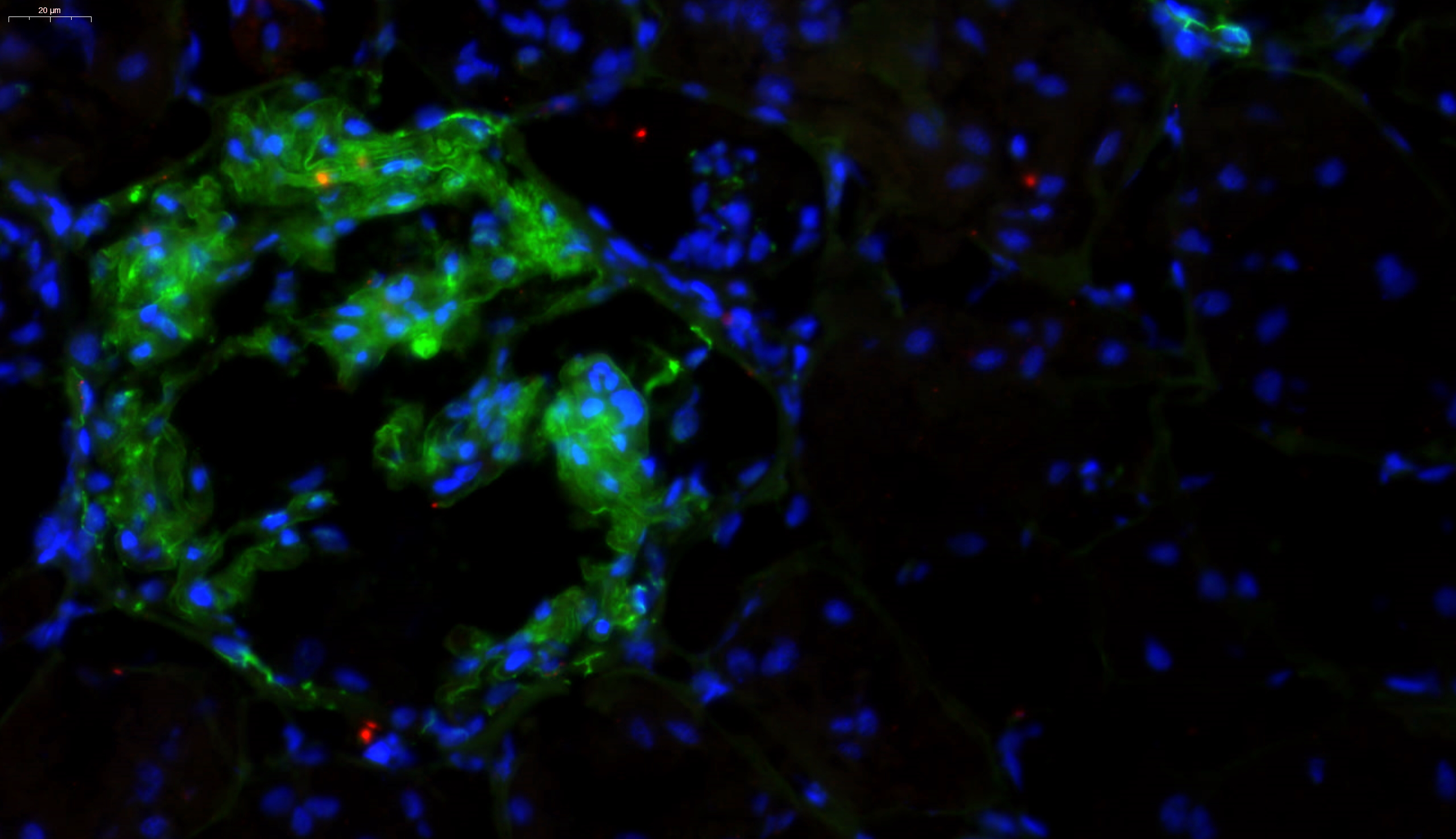

Supplement: Supplementary file 18 [file DataSheet7.ZIP › Original data of Figure 7/Figure 7A-Merge (RAP)× 400.tif]

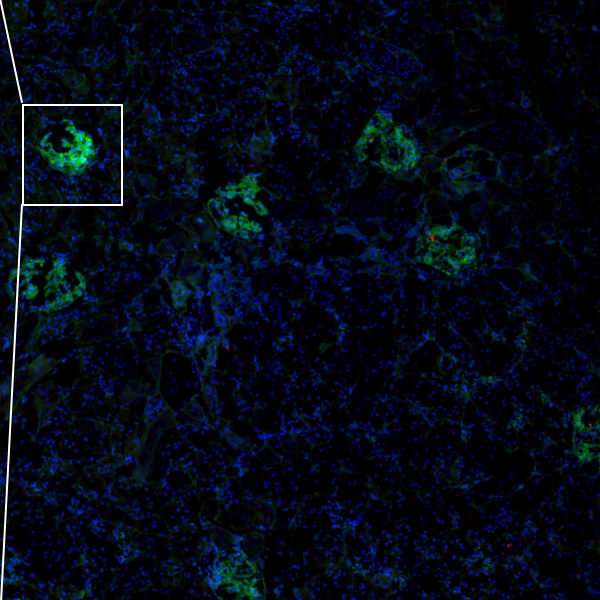

Supplement: Supplementary file 18 [file DataSheet7.ZIP › Original data of Figure 7/Figure 7A-Merge (Sham)× 100.tif]

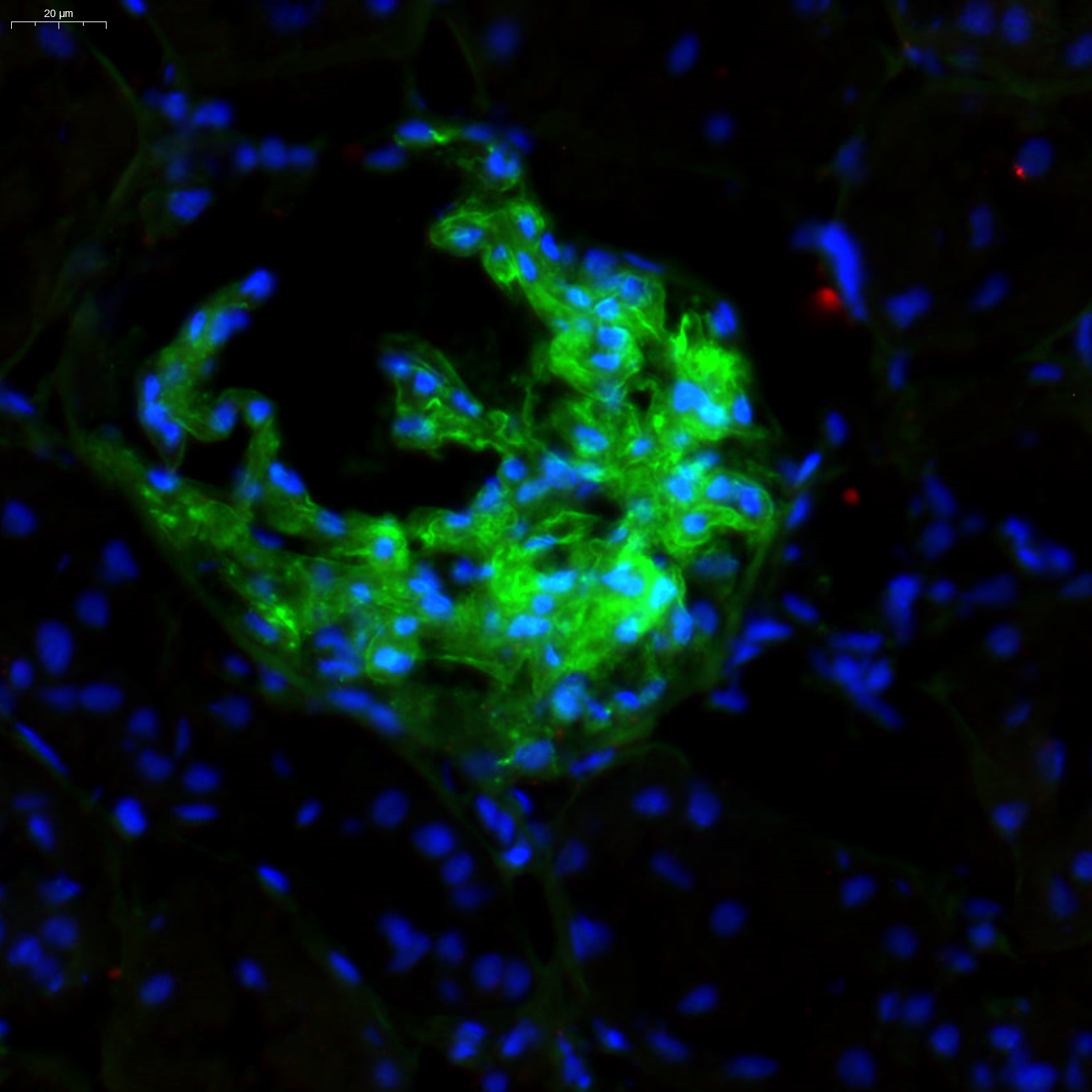

Supplement: Supplementary file 18 [file DataSheet7.ZIP › Original data of Figure 7/Figure 7A-Merge (Sham)× 400.tif]

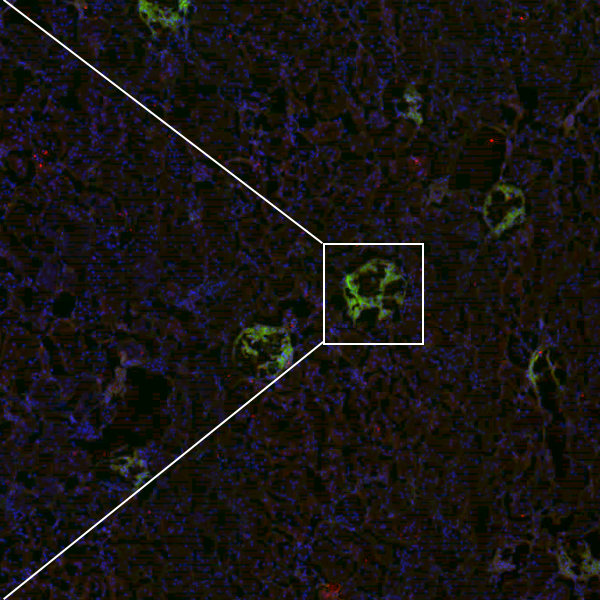

Supplement: Supplementary file 18 [file DataSheet7.ZIP › Original data of Figure 7/Figure 7A-Merge (Vehicel)× 100.tif]

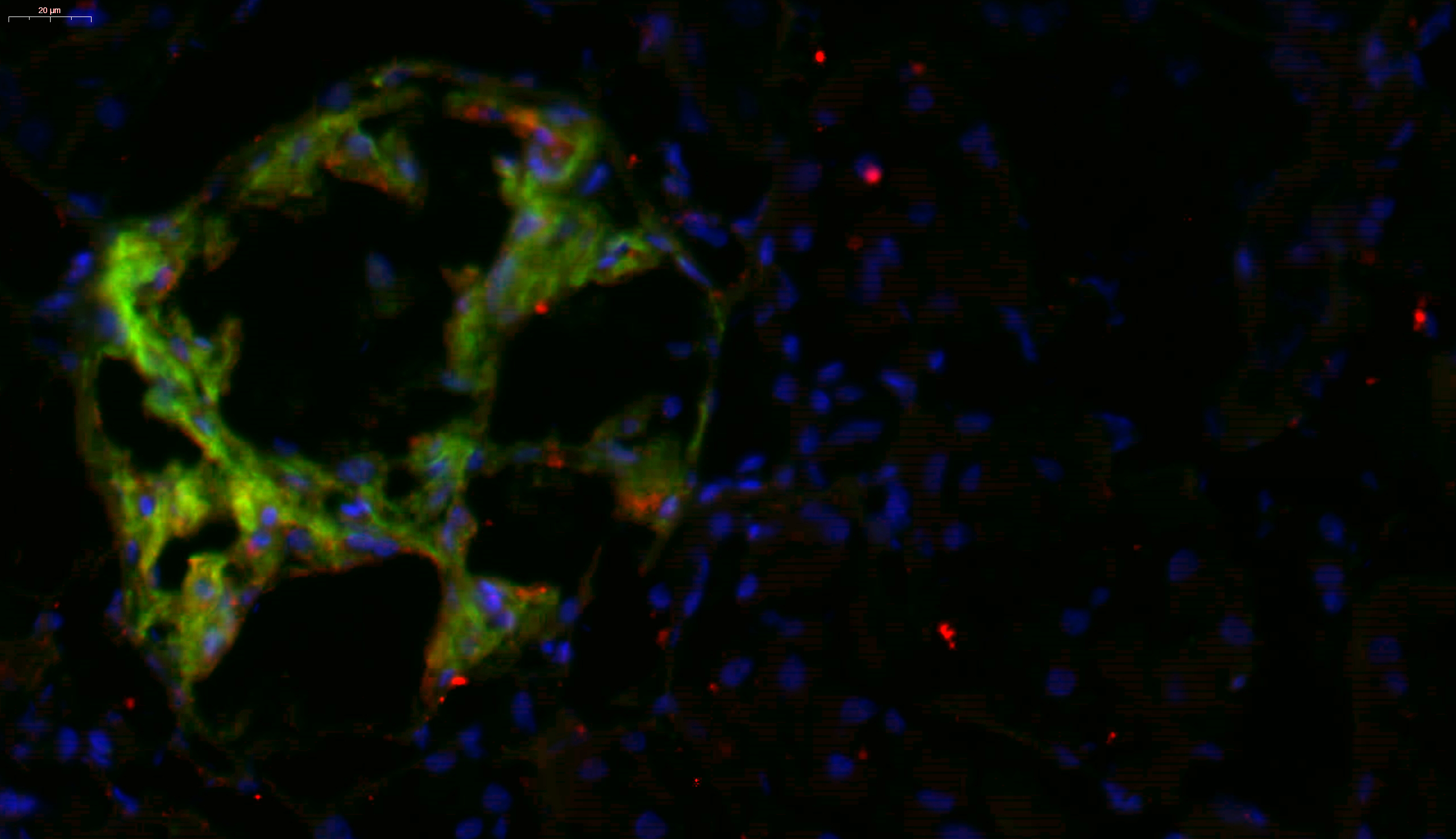

Supplement: Supplementary file 18 [file DataSheet7.ZIP › Original data of Figure 7/Figure 7A-Merge (Vehicel)× 400.tif]

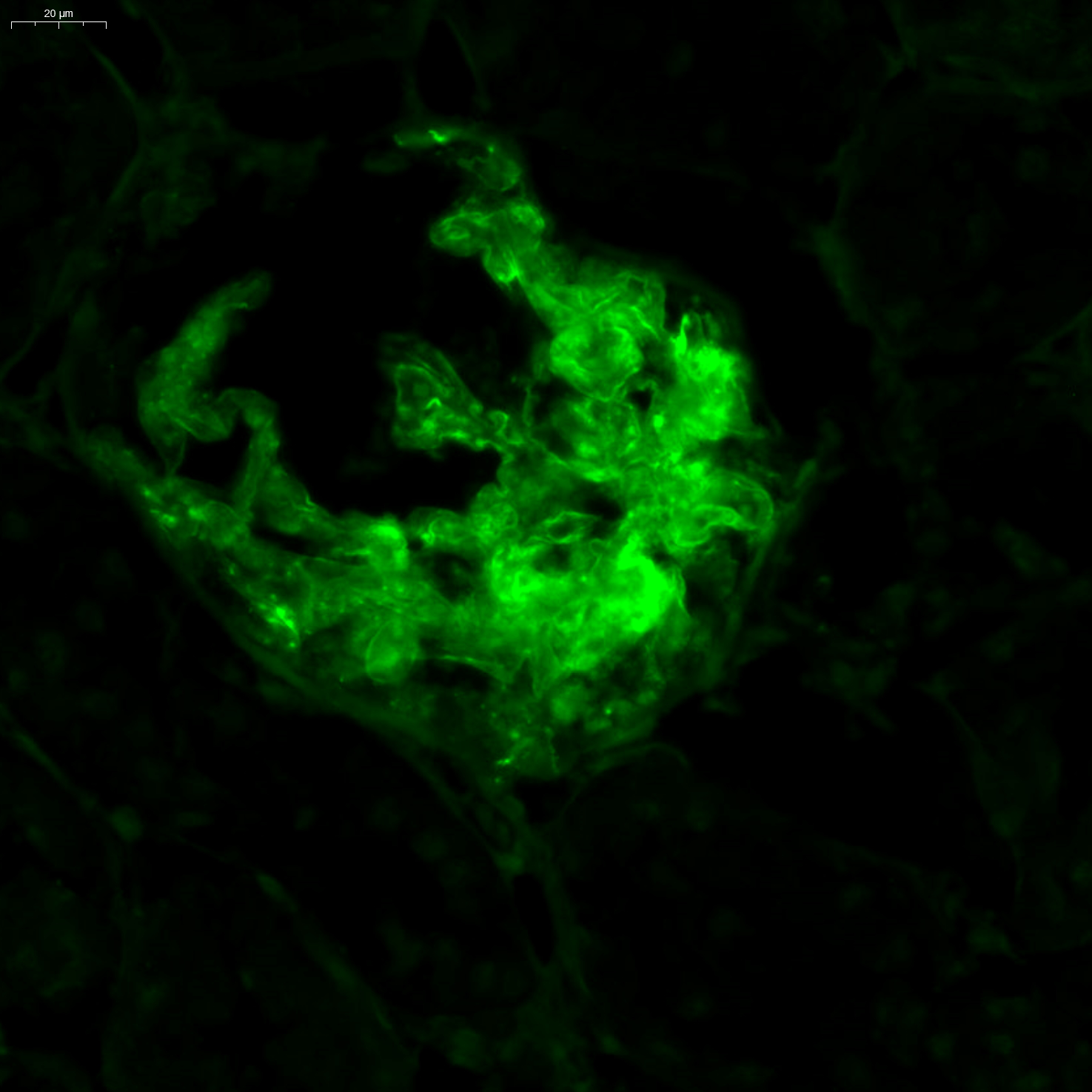

Supplement: Supplementary file 18 [file DataSheet7.ZIP › Original data of Figure 7/Figure 7A-ZO-1 (Sham).tif]

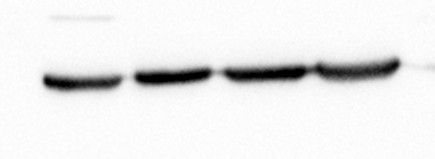

Supplement: Supplementary file 18 [file DataSheet7.ZIP › Original data of Figure 7/Figure 7C-GAPDH-1.jpg]

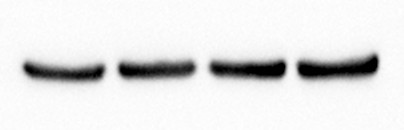

Supplement: Supplementary file 18 [file DataSheet7.ZIP › Original data of Figure 7/Figure 7C-GAPDH-2.jpg]

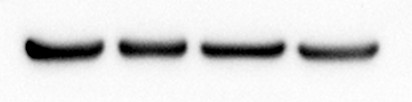

Supplement: Supplementary file 18 [file DataSheet7.ZIP › Original data of Figure 7/Figure 7C-GAPDH-3.jpg]

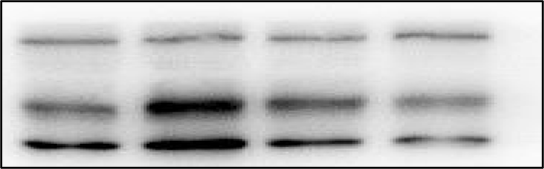

Supplement: Supplementary file 18 [file DataSheet7.ZIP › Original data of Figure 7/Figure 7C-GSDMD and GSDMD-N-1.tif]

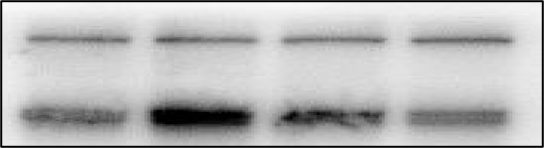

Supplement: Supplementary file 18 [file DataSheet7.ZIP › Original data of Figure 7/Figure 7C-GSDMD and GSDMD-N-2.tif]

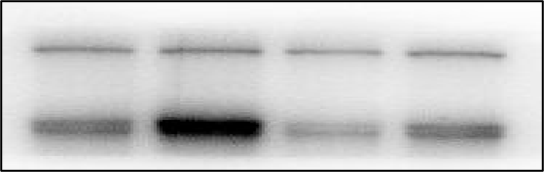

Supplement: Supplementary file 18 [file DataSheet7.ZIP › Original data of Figure 7/Figure 7C-GSDMD and GSDMD-N-3.tif]

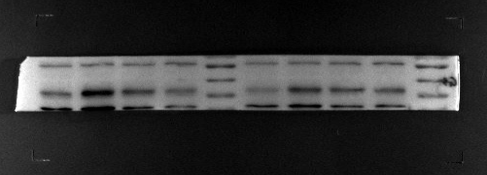

Supplement: Supplementary file 18 [file DataSheet7.ZIP › Original data of Figure 7/Figure 7C-GSDMD and GSDMD-N-original image-1.tif]

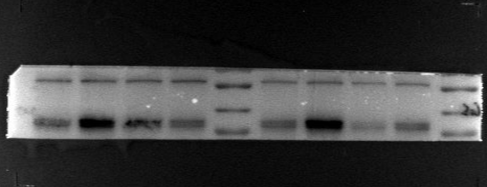

Supplement: Supplementary file 18 [file DataSheet7.ZIP › Original data of Figure 7/Figure 7C-GSDMD and GSDMD-N-original image-2.tif]

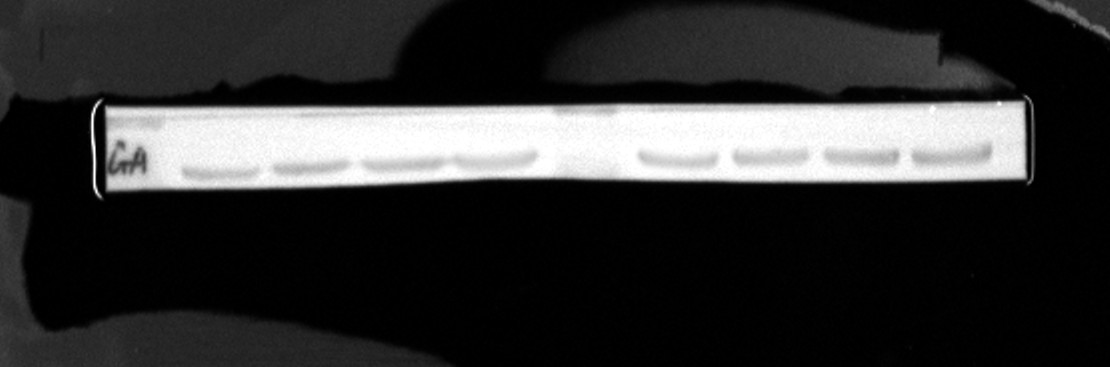

Supplement: Supplementary file 18 [file DataSheet7.ZIP › Original data of Figure 7/Figure 7C-original image-1.jpg]

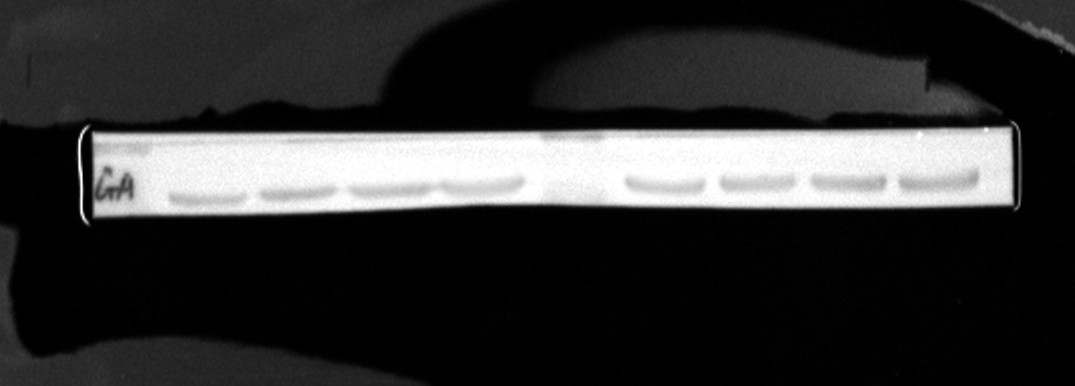

Supplement: Supplementary file 18 [file DataSheet7.ZIP › Original data of Figure 7/Figure 7C-original image-2.jpg]

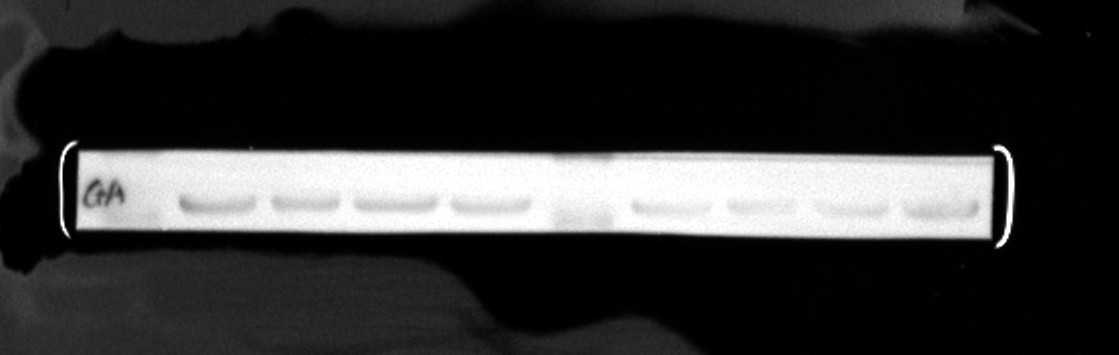

Supplement: Supplementary file 18 [file DataSheet7.ZIP › Original data of Figure 7/Figure 7C-original image-3.jpg]
